# Quantitative first principles calculations of protein circular dichroism in the near-ultraviolet†
†Electronic supplementary information (ESI) available. See DOI: 10.1039/c7sc00586e



**DOI:** 10.1039/c7sc00586e

**Published:** 2017-03-24

**Authors:** Zhuo Li, Jonathan D. Hirst

**Affiliations:** a School of Chemistry , University of Nottingham , University Park , Nottingham NG7 2RD , UK . Email: jonathan.hirst@nottingham.ac.uk

## Abstract

Vibrational structure in the near-UV circular dichroism (CD) spectra of proteins is an important source of information on protein conformation and can be exploited to study structure and folding. A fully quantitative theory of the relationship between protein conformation and optical spectroscopy would facilitate deeper interpretation of and insight into biophysical and simulation studies of protein dynamics and folding. We have developed new models of the aromatic side chain chromophores toluene, *p*-cresol and 3-methylindole, which incorporate *ab initio* calculations of the Franck–Condon effect into first principles calculations of CD using an exciton approach. The near-UV CD spectra of 40 proteins are calculated with the new parameter set and the correlation between the computed and the experimental intensity from 270 to 290 nm is much improved. The contribution of individual chromophores to the CD spectra has been calculated for several mutants and in many cases helps rationalize changes in their experimental spectra. Considering conformational flexibility by using families of NMR structures leads to further improvements for some proteins and illustrates an informative level of sensitivity to side chain conformation. In several cases, the near-UV CD calculations can distinguish the native protein structure from a set of computer-generated misfolded decoy structures.

## Abstract 2

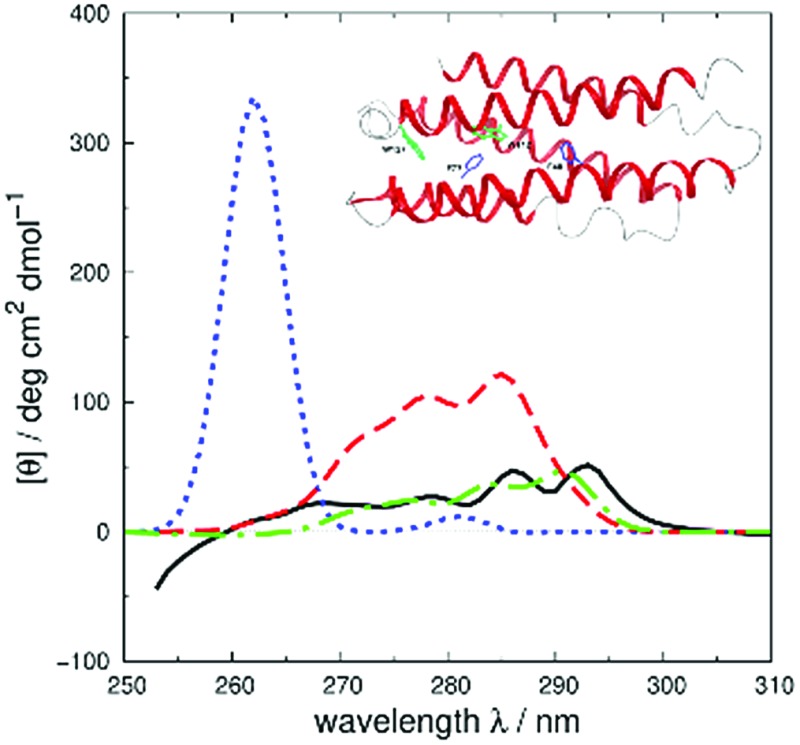
Including the vibrational structure of the electronic transitions of aromatic groups allows quantitative calculation of protein near-UV circular dichroism.

## Introduction

Electronic circular dichroism (CD) spectroscopy is a widely used technique which shows the differential absorption of left-handed and right-handed circularly polarized light by chiral molecules, for example, proteins.^1^ The side chains of aromatic amino acids play a key role, through hydrophobic packing, in the structure of proteins,^2^ hydrogels^3^ and many non-biological systems, such as conjugated polymers and supramolecular assemblies with aromatic side groups. They can also be integral to protein function, often mediating molecular recognition processes and catalysis. Thus, biophysical measurements to probe aromatic–aromatic interactions can furnish new insight into fundamental aspects of protein behaviour.

Bands in the electronic CD spectra of proteins in the near-ultraviolet (near-UV) region (240 to 320 nm) provide useful information on the environment and interactions of aromatic side chains. The measurement of the near-UV CD of proteins has been instrumental, for example, in advancing our understanding of compact non-native states of proteins.^4^ The ππ* electronic excitations on the aromatic rings of phenylalanine, tyrosine and tryptophan are sensitive to the local surroundings and can couple with one another. The separation and relative orientation of chromophores varies depending on the structure of the protein. Thus, the near-UV CD spectra of proteins reflect differences in conformation and can be used to probe the structure and dynamics of protein folding. Over many years, there have been applications of near-UV CD spectroscopy for biochemical and biophysical investigations. For example, site-directed mutagenesis has been used to investigate how aromatic residues contribute to the near-UV spectra.^5–8^ CD spectroscopy has been used to monitor and study binding processes,^1,9^ conformational changes induced by sequence modification,^8,10^ and folding and unfolding processes under various conditions,^11–13^ sometimes on ultrafast timescales.^14^ However, the relationship between structure and the near-UV CD spectrum is still not understood in a quantitative fashion, although some progress has been made.

The current state of the art and some of the background on the electronic structure and CD of proteins has been reviewed elsewhere.^15^ Fully *ab initio* calculations are feasible and routine for small molecules. However, more approximate approaches are necessary for computing the CD of proteins from first principles to cope with the size and the flexibility of the systems. The matrix method is a well-established formulation derived from the exciton model.^16–19^ Exciton and vibronic coupling theories have recently been reviewed in the context of molecular dimers.^20^ A key aspect in the matrix method is the set of parameters used to model the electronic excitations of the various chromophores in proteins and much effort has been devoted to developing parameter sets from *ab initio* quantum chemical methods.

The far-UV CD spectra of proteins have been extensively studied by theoretical approaches, partly because the intensity or the mean residue ellipticity at 222 nm correlates with the helical content of proteins. Parameter sets for the peptide bond, which contributes to the absorption in the far-UV region, have been calculated with various levels of theory on different model compounds.^21,22^ Other aspects which contribute to the far-UV CD spectrum have been studied, for example, the electronic transitions to high-lying B states of aromatic amino acids,^23,24^ the effect of hydrogen bonding^25^ and the vibrational structure of the ππ* transition of the amide group.^26^ Parameter sets of aromatic amino acids have been previously developed^27^ using *ab initio* complete active space multiconfigurational second-order perturbation theory^28^ (CASPT2) calculations on benzene, phenol and indole; these yielded more accurate computed near-UV CD spectra than earlier semi-empirical parameters.

Vibrational fine structure is commonly observed in high-resolution gas phase spectra and is a source of line-broadening in the solution phase. The Franck–Condon principle explains the appearance of the vibrational progression of an electronic excitation, which is governed by the extent of the overlap between the wave functions of each vibrational level in the electronic ground and excited states. The vibrational structure in the near-UV absorption and CD spectra of aromatic amino acids varies significantly depending on solvent.^29–33^ The sensitivity of the electronic transitions of aromatic amino acids to molecular environment leads to a richness of information in the near-UV CD spectra of proteins. The vibrational progressions of individual chromophores have been obtained by examining the difference spectra of wild-type proteins and their mutants.^5,34,35^ For various complexes between calmodulin and target peptides, deconvolution of CD spectra into a well-resolved ^1^L_b_ component and a broad ^1^L_a_ component has been used to analyse the characteristic contributions of these transitions in other complexes.^36^

The importance of taking vibrational structure into account in CD calculations has been established for various molecules. The gas phase CD spectrum of dimethyloxirane was better reproduced once the vibrational structure was incorporated in time-dependent DFT calculations.^37^ A cancellation of a strong vibrational progression by a series of positive bands rationalized the disappearance of a negative band in the experimental spectrum. In calculations of the CD spectra of naphthalenediimide (NDI) dimers and oligomers, consideration of the Franck–Condon effect was also essential.^38,39^ Incorporation of the vibrational progression of two electronic transitions of NDI can resolve two additional bands with relative intensities close to the experimental spectrum.

In our previous work, the vibrational structure of π_nb_π* transitions of the backbone chromophore has been incorporated in the calculation of the far-UV CD spectra of a set of 49 proteins.^26^ The features in the experimental spectra of α-helical proteins are better resolved with parameters considering the vibrational structure. For more irregular β structure proteins, there is a better correlation between the calculated and the experimental intensity in the far-UV.

In this study, we consider the Franck–Condon effect in calculations of the near-UV CD of proteins. The vibrational structure of the electronic transitions of aromatic amino acids is obtained from *ab initio* calculations on toluene, *p*-cresol and 3-methylindole, which have been widely studied both experimentally^40–48^ and theoretically^49–53^ as representing the chromophoric part of phenylalanine, tyrosine and tryptophan. The effectiveness of the parameter sets accounting for vibrational structure are assessed by calculations on a set of 40 proteins. We assess the contribution of an individual aromatic amino acid by considering changes in spectra due to mutation. Calculations with the NMR-determined structures are used to investigate the influence of the conformational diversity and turn out to further improve the accuracy of the calculations in the majority of the cases studied. Calculations on native structures of proteins are compared with computer-generated misfolded structures to assess the utility our new parameters in contributing to structure prediction and determination.

## Methods

### The matrix method

The rotational strength *R* ^0*k*^, which is the counterpart of absorbance in the absorption spectrum, can be calculated as the dot product of the electric and magnetic transition moments using the Rosenfeld equation^54^ (eqn (1)):

1 *R*^0*k*^ = Im(*ψ*^0^|*μ⃑*|*ψ*^*k*^*ψ*^*k*^|*m*|*ψ*^0^)

where *ψ* ^0^ and *ψ* ^*k*^ represent the wave functions of the ground and the excited states while *μ⃑* and *m* are the electric and the magnetic transition dipole moments of the excitation from state 0 to *k*. Im means the imaginary part of the product.

Due to the size and the complexity of proteins, it is impractical to obtain the wave functions from *ab initio* calculations. Instead, we calculate protein CD spectra using the matrix method^16,18,19^ as implemented in DichroCalc code.^55^ In the matrix method, the proteins are considered as being composed of *M* chromophores. The wave function of the protein in its *k* ^th^ excited state, *ψ* ^*k*^, is expressed as a linear combination of electronic configurations, *Φ*_*i*a_ (eqn (2)). The first summation is over the *M* chromophores while the second one is over the *n*_*i*_ electronic transitions of individual chromophore *i*. The interactions of the states are reflected by the expansion coefficients, *c*_*i*a_^*k*^. In each electronic configuration, only one chromophore *i* is in its excited state *a* while the rest of the chromophores are in their ground state 0, expressed by the wave function of an individual chromophore, *φ*_*i*a_ (eqn (3)). No charge transfer transitions are considered in the current calculations.2
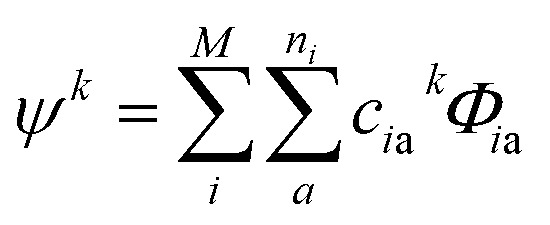

3 *Φ*_*i*a_ = *φ*_10_…*φ*_*i*a_…*φ*_*j*0_…*φ*_*M*0_

The wave functions *ψ* ^*k*^ of the protein and their corresponding energies can be calculated by solving the Schrödinger equation (eqn (4)) under the matrix formalism. The total Hamiltonian operator *Ĥ* of the protein is constructed with the local Hamiltonian *Ĥ*_*i*_ of chromophore *i* and the intergroup potential *V*_*ij*_, between two transitions (eqn (5)). The dimension, *M*, of the Hamiltonian matrix will be extended if multiple transitions are taken into account for chromophore *i*.

4 *Ĥψ*^*k*^ = *E*^*k*^*ψ*^*k*^

5
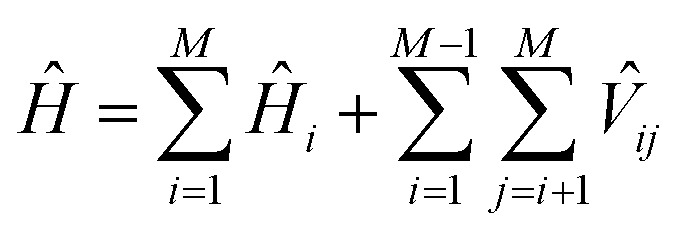



An illustrative Hamiltonian matrix is shown below (eqn (6)). Here we consider a two-chromophore (denoted 1 and 2) model with two transitions (denoted *a* and *b*) for each chromophore. Two vibrational levels (denoted v_1_ and v_2_) of the transition to the excited state *a* are shown.6
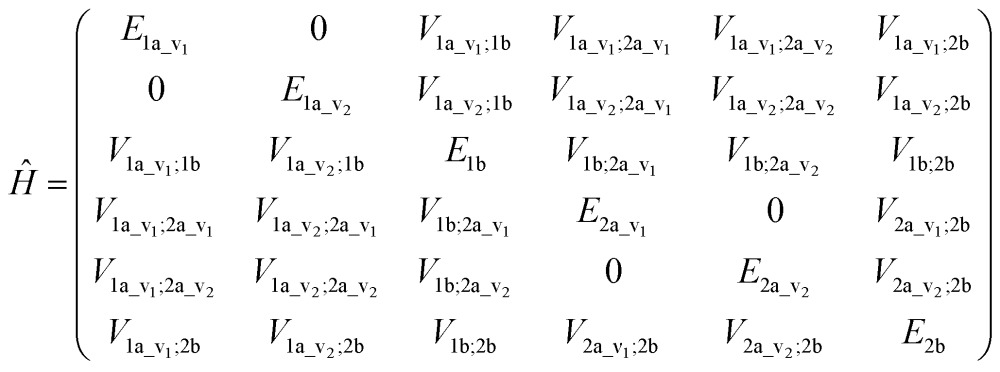
where the diagonal element *E* is the energy of a distinct transition of one chromophore, the off-diagonal element *V* is either the intergroup interaction or the static-field mixing of excitations of a single group, also known as one-electron mixing, as described by Condon, Alter and Eyring,^56^ and the interaction between different vibrational levels of a given electronic excited state in a particular chromophore is set to zero.

The interactions between chromophores are considered to be purely electrostatic. The interaction potential *V*_*ij*_ is then expressed with transition charge densities *ρ* and the separation *r* between chromophores. The transition charge densities are represented by a set of monopole charges so that the interactions between transitions can be calculated as the Coulomb interactions between these charges (eqn (7) and (8)).^15^
7
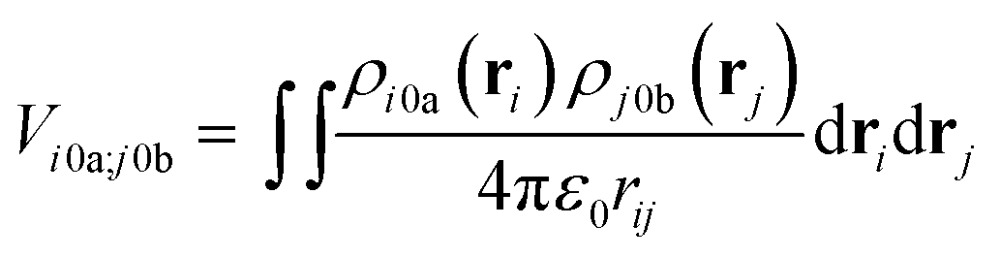
where *ρ* is the electronic transition density of either chromophore *i* or *j* from the ground state 0 to the excited state *a* or *b*, respectively; *ε*_0_ is the vacuum permittivity and *r*_*ij*_ is the separation of the chromophores.8
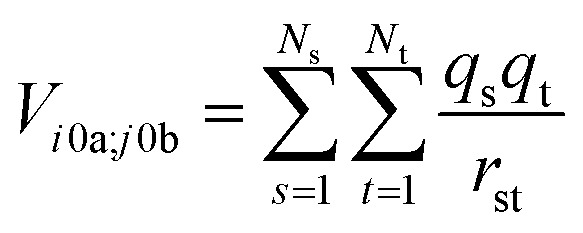
where *q* is the magnitude of the monopole charges on the chromophores, *N* is the number of charges and *r*_st_ is the distance between two charges.

By solving the Schrödinger equation, through diagonalizing the Hamiltonian matrix by a unitary transformation (eqn (9)), the energies of the transitions of the protein are the eigenvalues. The coefficients, *c*_*i*a_^*k*^, which show the contributions of each excitation of individual chromophore to the system, are the eigenvectors.

9 *U*^–1^*ĤU* = *H*_diag_

The electric and magnetic transition dipole moments of the transitions of the protein can be calculated from the transition moments of the individual chromophores with the unitary matrix *U*_a*i*_ (eqn (10) and (11)).10
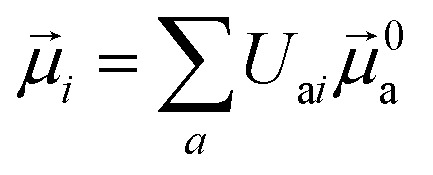

11
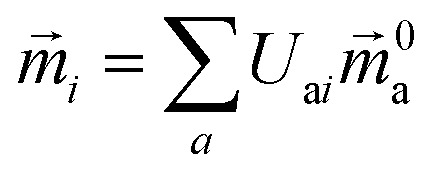



A convolution of the calculated line spectrum with a Gaussian type function is needed to mimic the observed broadened spectrum.^15^ A 4 nm bandwidth is used in the near-UV for all the calculations in this study.

### Franck–Condon calculations

The electronic ground and ^1^L_b_ excited states of toluene, *p*-cresol and 3-methylindole and also the ^1^L_a_ state in the case of 3-methylindole (the ^1^L_a_ states of toluene and *p*-cresol occur in the far-UV at ∼208 nm and ∼216 nm, respectively) were calculated using the MOLPRO package^57^ version 2010.1 employing the state-averaged complete active space self-consistent field (CASSCF) method^58^ with the 6-31G* basis set for various active spaces: six electrons in seven orbitals for toluene (six valence π orbitals and one Rydberg orbital), six electrons in six orbitals for *p*-cresol and ten electrons in ten active orbitals (nine valence π orbitals and one Rydberg orbital^59^) for 3-methylindole. The calculations of the Franck–Condon factors require the equilibrium geometries of the ground and the excited states, the vibrational mode vectors and the related frequencies; these were all taken from the *ab initio* calculations.

The Franck–Condon factors, which are the square of the overlap integrals of the vibrational wave functions between the ground and the excited states, govern the intensity distribution along the progression in the absorption spectrum. The freely available software, ezSpectrum, was used to calculate the Franck–Condon overlap integrals, *f*_v_, within the double-harmonic approximation.^60^ There is no severe distortion of the vibrational modes in the electronic ground and excited states which dominate the progressions for toluene, *p*-cresol or 3-methylindole. The parallel approximation, which assumes that the normal coordinates of the electronic ground and excited states are the same, was adopted so that multidimensional Franck-Condon integrals are the products of one dimensional Franck–Condon integrals which are computed analytically. The selection of the vibrational modes (shown later) was based on the symmetry and guided by previous studies^29,30,32^ of the vibrational features in the absorption and CD spectra. The transitions were considered to be excited from the lowest vibrational level of the ground electronic state, *i.e.*, no hot bands were taken into account, but combination bands were allowed. Six vibrational levels were calculated for individual modes in the excited state. Franck–Condon integrals with a magnitude greater than 0.1 were considered to be sufficiently significant to incorporate into the CD calculations. The Franck–Condon integrals were normalized using eqn (12).12
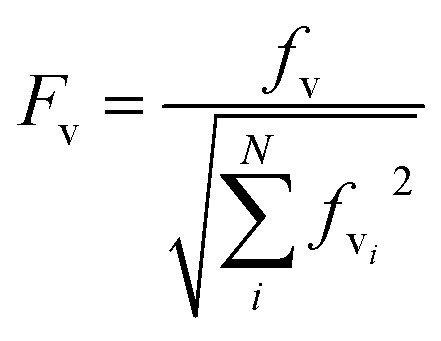



The vibrational transitions are incorporated by modifying the electric transition dipole moments and the monopole charges representing the transition charge densities by scaling by the normalized Franck–Condon overlap integral. No interaction between the vibrational levels of the same chromophore is considered in the CD calculation. We consider the first six to nine transitions in a vibrational progression, with the precise number determined based on a threshold of 0.1 for the Franck–Condon integral (below which transitions are taken to make a negligible contribution). The new parameters are provided as ESI† and are also accessible *via* the DichroCalc web interface at: ; http://comp.chem.nottingham.ac.uk/dichrocalc.

### Protein dataset, mutants and decoys

Experimental CD spectra of 40 proteins (Table 1) were assembled from the literature. The units of the experimental spectra were all standardized to be mean residue ellipticity to facilitate comparison of calculated and experimental spectra. The crystal structures of these proteins and the solution NMR structures of 13 proteins are available from the Protein Data Bank (PDB).^61^ The selection of a certain PDB entry was made to match as closely as possible factors such as species, bound ligands and other experimental conditions under which the CD spectra had been measured. All chains of multimeric proteins are used in our calculations. The calculated spectra of NMR structures are obtained by averaging the spectra of each individual structure in the PDB file.

**Table 1:** The 40 proteins studied: name, PDB codes, length (*i.e.* number of residues in the X-ray structure) and the number of aromatic residues and disulfide bonds. The four proteins whose mutants are studied are indicated by an asterisk. References to the experimental spectra of individual proteins are given in the ESI

Protein	PDB entry		Length	No. aromatic amino acids			
	X-ray	NMR		Phe	Tyr	Trp	S–S
Acetylcholinesterase	2ACE		537	34	17	14	3
Adenylate kinase	2ECK		428	10	14	0	0
α-Lactalbumin	1A4V		123	3	5	3	4
α-Toxin	1QM6		740	30	50	20	0
Apolipophorin III	1AEP	1LS4	161	2	0	2	0
Barnase*	1A2P	1FW7	330	12	21	9	0
β-2 microglobulin (human)	1LDS	1JNJ	100	5	6	2	1
β-Lactamase	1BTL		263	5	4	4	1
P.69 pertactin	1DAB		539	10	5	7	0
Bovine pancreatic trypsin inhibitor*	5PTI	1PIT	58	4	4	0	3
Calmodulin	4CLN		148	9	1	0	0
Cardiotoxin	4OM4	1CRF	300	10	15	0	20
Chymotrypsinogen A	2CGA		490	6	4	8	10
Dehydroquinase II	2BT4		1884	36	48	12	0
Dihydrofolate reductase	4P3Q		159	6	4	5	0
Glucose oxidase	1CF3		583	18	27	10	1
Hen egg white lysozyme	1HF4	1E8L	258	6	6	12	8
Human carbonic anhydrase II*	2CBA		260	12	8	7	0
Human serum albumin	1AO6		1170	62	35	2	34
Insulin	5ENA		51	3	4	0	3
Interleukin 4 (cytokine)	2B8U	1CYL	129	6	2	1	3
Interleukin 6	1ALU	2IL6	186	7	3	1	2
Monellin	1IV9	1MNL	192	12	14	2	0
Myoglobin (whale)	1UFP		154	6	3	2	0
Neocarzinostatin	1NOA		113	5	1	2	2
Odorant binding protein	1A3Y		298	17	10	2	1
Papain	3LFY		424	8	38	10	6
Pectate lyase C	2PEC		353	8	17	7	2
Phosphatidylethanolamine-binding protein	1A44		185	3	10	5	0
Phospholipase A2 (Ca^2+^)	1PSJ		124	6	7	3	7
Relaxin	6RLX		104	2	2	3	6
Rhodanese	1DP2		293	15	11	8	0
Ribonuclease T1	1RN1	1YGW	104	4	9	1	2
Ribonuclease A*	1AFU	2AAS	248	6	12	0	8
Staphylococcal nuclease	1STN	2KQ3	149	3	7	1	0
Extracellular domain of human tissue factor	2HFT		218	9	11	4	2
Sticholysin II	1O72		350	12	24	10	0
Subtilisin BPN′	1ST2		275	3	10	3	0
Thioredoxin	2TRX	1XOA	216	8	4	4	2
Tryptophan synthase α-subunit	1WQ5		536	24	14	0	0

CD spectra of mutants of four proteins were also calculated. Mutations from tyrosine to phenylalanine were modelled by deleting the hydroxyl group without any subsequent energy minimization. For mutations involving tryptophan, the residue is first substituted by the corresponding amino acid by the mutation prediction function on the WHAT IF server^62^ followed by a steric clashes refinement.^63^

The 3DRobot server^64^ was used to construct a set of calculated non-native structures (decoys) for 34 of the 40 proteins in our protein dataset. The other six proteins were not considered, because 3DRobot was unable to generate sufficient decoys in these cases. 1000 decoys were generated for each protein with an all-atom structural RMSD cut-off set to 12 Å. For multimeric proteins where the chains have identical sequences, only a single chain was used to generate decoys. For cases where the chains have distinct sequences, *e.g*., relaxin (; 6RLX) and insulin (; 5ENA), chain identifiers were unified so that 3DRobot generated appropriate decoys.

## Result and discussion

### Toluene

Hameka and Jensen^53^ reported calculated energies, geometries and vibrational frequencies of the ground and first excited states of toluene using the CASSCF method with four electrons in an active space of four orbitals. Our calculation employed a larger active space: six electrons in seven orbitals, which comprise three occupied π orbitals, three virtual π orbitals and one Rydberg orbital. The latter is essential for the calculation to converge. The transition to the first singlet excited state of toluene is localized in the π orbitals of the ring. In Table S1,† the geometries of the ground and first excited states are compared with those determined by electron diffraction.^48^ Although the barriers to internal rotation are very small, both states adopt staggered configurations, which is consistent with the predominant conformation of the β-carbon of phenylalanine. For the ground state, our calculations agree well with the experimental and previously calculated results. For the excited state, our calculations predict the C–C bonds of the ring to be extended by 0.04 Å compared to the ground state geometry. This is qualitatively similar to the calculations with the smaller active space, but there were some quantitative differences.

The assignment and the calculated frequencies of the selected vibrational normal modes are in good agreement with the experimentally identified IR frequencies of toluene^44^ (Tables 2 and S2†). There are cases where multiple internal coordinates contribute to the same mode while some vibrational motions distribute in different fundamentals in the excited state (Table S2†).

**Table 2:** Calculated vibrational frequencies of selected normal modes of the ground (S_0_), the first (^1^L_b_) and the second (^1^L_a_) excited states of toluene, *p*-cresol and 3-methylindole compared to their experimental counterparts

	Assignment	S_0_ frequency/cm^–1^		^1^L_b_, ^1^L_a_ frequency/cm^–1^		
				^1^L_b_		^1^L_a_
		Calc.	Expt.	Calc.	Expt.	Calc.
Toluene	C–C bend	838	788	787	736	
	C–H bend	1107	1030	978	935	
*p*-Cresol	C–C bend	890	844	845		
	C–H bend	1280	1174	1242		
3-Methylindole	C–C stretch	806	758	775	740	784
	Benzene C–H bend	1049	1009	957	919	1047

Experimentally, the vibrational structure in the absorption and the CD spectra in the near-UV of phenylalanine and derivatives shows two progressions, both with a spacing of 930 cm^–1^.^30^ One arises from excitations from the ground state to the lowest vibrational level of the first electronic excited state (denoted 0–0), while the other corresponds to the 0 + 520 cm^–1^ vibrational state of the excited state. The progression of the transition to the 0 + 520 cm^–1^ vibrational level has bands of the opposite sign to the 0–0 progression in the CD spectra of phenylalanine and derivatives.

**Fig. 1:**
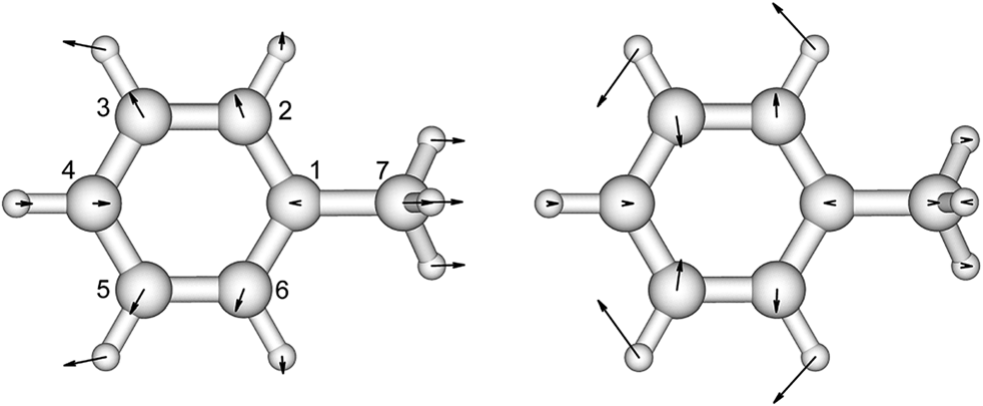
The vibrational modes which are Franck–Condon active in the excitation process of toluene. The direction and the magnitude are illustrated by the arrows located on the atoms.

There is no severe torsion between the equilibrium geometries of the ground and the first excited state of toluene. The overlap of the vibrational wave functions between the ground and the excited states is described well by one-dimensional Franck–Condon factors in the double-harmonic approximation. Two vibrational normal modes (Fig. 1) are Franck–Condon active. The normalized Franck–Condon integrals and the transition energies are given in Table 3. The CASSCF calculation gives a 0–0 transition energy of 37 890 cm^–1^ (264 nm) to the first excited state. This is close to the experimental peak position of 268 nm observed in the near-UV CD spectra of phenylalanine and its derivatives.^30^ In our parameters for CD calculations, we place the origin of toluene at 268 nm. The spacing in each progression used the excited state frequency of C–H bend mode. The frequency of C–C bend mode was taken to be 520 cm^–1^ (instead of the calculated 787 cm^–1^) to match the experimental spectra of phenylalanine.^30^ The discrepancy comes from the influence of the amine and the carboxylic acid groups in phenylalanine, which elongate the C1–C7 bond. At higher energies, the vibrational structure of the spectra becomes more complicated and overlapped, due to combination modes and anharmonicity. Calculation of the CD spectra considering only the predominant modes represents a step-wise approach to increasing the complexity of the computational methodology.

**Table 3:** Transition energies and Franck–Condon integrals of transitions to different vibrational levels (‘vib’ level) of the ^1^L_b_ states of toluene, *p*-cresol and 3-methylindole and the ^1^L_a_ state of 3-methylindole. The vibrational level is denoted as MvN, where N is the assignment of the vibrational mode and M is the vibrational level of that mode. The C–C bend, C–H bend and C–C stretch mode are denoted as B_CC_, B_CH_ and S_CC_, respectively

‘vib’ level	Energy/cm^–1^	FC integral
**Toluene**		
0	37 310	0.59
1vB_CC_	37 830	–0.43
1vB_CH_	38 288	0.49
1vB_CC_/1vB_CH_	38 808	–0.35
2vB_CH_	39 266	0.26
1vB_CC_/2vB_CH_	39 786	–0.18

***p*-Cresol**		
0	34 965	0.52
1vB_CC_	35 810	0.58
1vB_CH_	36 221	0.17
2vB_CC_	36 655	0.45
1vB_CH_/1vB_CC_	37 066	0.18
3 B_CC_	37 500	0.28
1vB_CH_/2vB_CC_	37 911	0.14
4vB_CC_	38 345	0.14

**3-Methylindole** ^**1**^ **L**_**b**_		
0	34 424	0.62
1vS_CC_	35 199	0.47
1vB_CH_	35 381	0.44
2vS_CC_	35 974	0.23
1vB_CH_/1vS_CC_	36 156	0.33
3vS_CC_	36 749	0.09
1vB_CH_/2vS_CC_	36 931	0.17

**3-Methylindole** ^**1**^ **L**_**a**_		
0	35 088	0.57
1vS_CC_	35 872	0.53
1vB_CH_	36 135	0.32
2vS_CC_	36 656	0.34
1vS_CC_/1vB_CH_	36 919	0.30
3vS_CC_	37 440	0.18
1vS_CC_/2vB_CH_	37 966	0.19
4vS_CC_	38 224	0.08
1vS_CC_/3vB_CH_	39 013	0.10

### *p*-Cresol

**Fig. 2:**
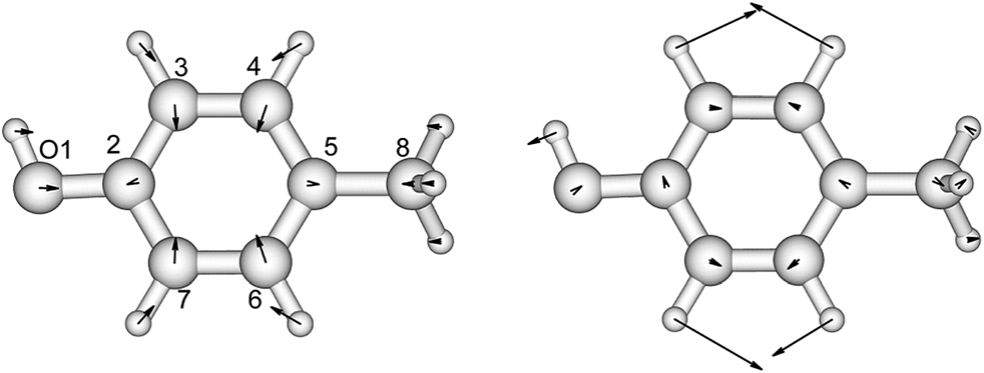
The vibrational modes which are Franck–Condon active in the excitation process of *p*-cresol. The direction and the magnitude are illustrated by the arrows located on the atoms.

*p*-Cresol is used instead of phenol to represent tyrosine in this study to take into account the influence of the methyl group on the geometries and vibrational frequencies of the ring. Its electronic ground and first singlet excited state have been studied experimentally^40–42,47^ and theoretically.^49,51–53^ Our calculated CASSCF geometries of these states agree well with the X-ray determined geometry^65^ and calculations using other levels of theory (Fig. 2 and Table S3†).^47,51^ The hydroxyl group is in the plane of the benzene ring while the methyl group is in a staggered configuration with one hydrogen perpendicular to the benzene ring. This is consistent with calculations at the B3LYP/cc-pVDZ level.^49^ In the excited state, the bond lengths of the C–C bond of the ring are extended by about 0.04 Å compared to the ground state, while the C–O bond length barely changes. In Table 2 (and Table S4†) the predominant normal modes that contribute to the vibrational features in the CD spectra are assigned and the calculated frequencies are compared to reported IR and Raman measurements^41^ of *p*-cresol.

The assignments and the normalized Franck–Condon integrals of *p*-cresol are shown in Table 3. The CASSCF calculation gave an overestimated 0–0 transition energy of 37 908 cm^–1^. We located the 0–0 transition at 34 965 cm^–1^ (286 nm) based on the CD spectrum of l-tyrosine ethyl ester in EPA (ethyl ether–isopentane–ethanol) at low temperature.^30^ The calculated excited state frequencies 890 cm^–1^ and 1280 cm^–1^ (Table 2) are used as the vibrational level interval in the excited state. This has been based on the transitions resolved and assigned in the resonance Raman spectrum^51^ of *p*-cresol and in the absorption and CD spectra of tyrosine derivatives.^28^ Two progressions, 0–0 and 0 + 1250 cm^–1^, were identified. The interval between transitions is 800 cm^–1^ for both progressions. Horwitz *et al.* ^30^ suggested that a 0 + 420 cm^–1^ transition is required to fill the gap between the first two vibrational transitions of the 0–0 progression. This is not taken into account in our CD calculations.

### 3-Methylindole

**Fig. 3:**
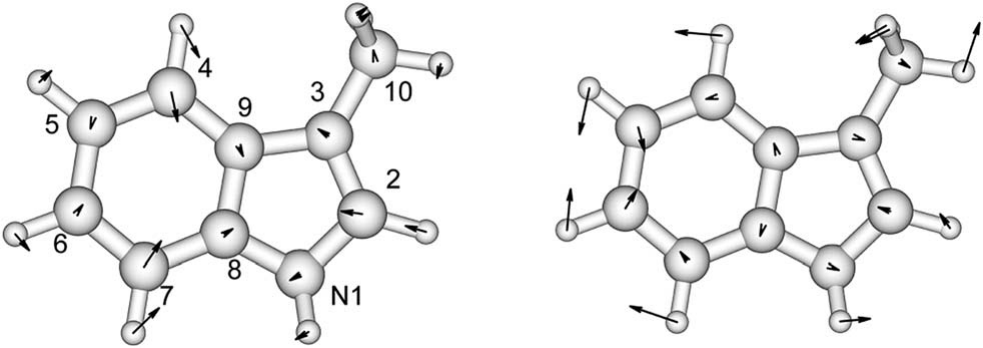
C–C stretch mode (left panel) and benzene C–H bend mode (right panel) of 3-methylindole which are Franck–Condon active in the excitation process. The direction and the magnitude of each normal mode are illustrated by the arrows located on the atoms.

Indole, as a model compound representing tryptophan, has been studied extensively using experimental^42,66,67^ and theoretical^68–74^ methods. The geometries and vibrational frequencies of the ground and two excited states, ^1^L_b_ and ^1^L_a_, of 3-methylindole have been compared to those of indole, to investigate the influence of the methyl group on the pyrrole ring.^42,46,50,75^ Our CASSCF calculations of these three states of 3-methylindole are compared with the experimental ground state geometry^76^ and the calculated ^1^L_b_ and ^1^L_a_ excited state geometries at various levels of theory (Fig. 3 and Table S5†). The calculated geometries of the ^1^L_b_ and ^1^L_a_ states are in good agreement with those computed for indole^75^ and for 3-methylindole^59^ using CASSCF and an atomic natural orbital (ANO-s) type basis set. The C4–C5 and C8–C9 bonds show the greatest change in the ^1^L_b_ state, while the N1–C2, C2–C3 and C6–C7 bonds change most in the ^1^L_a_ state.

We have compared the calculated vibrational frequencies of the ground and the two excited states with the experimental data measured with IR^77^ and one-colour resonant two-photon ionization (1C-R2PI) spectroscopy (Table S6†).^78^ The frequencies are in good agreement with experiment for both the ground and the ^1^L_b_ excited states. The contribution of each vibrational normal mode to the absorption spectrum of the ^1^L_b_ transition has previously been studied^75^ using TD-DFT and different functionals with the 6-311+G** basis set. The Franck–Condon, the Herzberg–Teller and Duschinsky effects were considered. The two predominant modes identified by those calculations, which contribute to the absorption spectrum, are with frequencies 806 cm^–1^ and 1049 cm^–1^; we also found this using ezSpectrum.^60^ The consistency of the description of the ^1^L_b_ state by several independent sources provides confidence in the ezSpectrum calculations and their application to the less studied ^1^L_a_ state.

The experimental absorption spectra and vibronic transitions of indole, 3-methylindole, *N*-acetyl-l-tryptophanamide and several proteins have been reported.^46^ Different solvents were used to perturb the spectra in order to help to assign bands, which is otherwise difficult due to the overlap of the ^1^L_b_ and ^1^L_a_ bands. Transitions to the ^1^L_a_ state are more sensitive to the environment than those to the ^1^L_b_ state.^79^ The 0–0 ^1^L_a_ transition energy may shift by approximately 5 to 10 nm due to interactions with the environment. However, it is difficult to distinguish all the bands from various electronic transitions. For the purposes of our protein CD calculations, the origins of the ^1^L_b_ and ^1^L_a_ bands are placed at 290.5 and 285 nm, respectively, in accordance with the experimental measurements of tryptophan derivatives^45^ and account for the energy shift induced by the surrounding residues or solvent. The Franck–Condon integrals for both states are shown in Table 3. The two progressions of the ^1^L_b_ transition are 0–0 and 0 + 775 cm^–1^ with a spacing of 957 cm^–1^, while the progressions of the ^1^L_a_ transition are 0–0 and 0 + 1047 cm^–1^ with a spacing of 784 cm^–1^.

### Calculated CD spectra

**Fig. 4:**
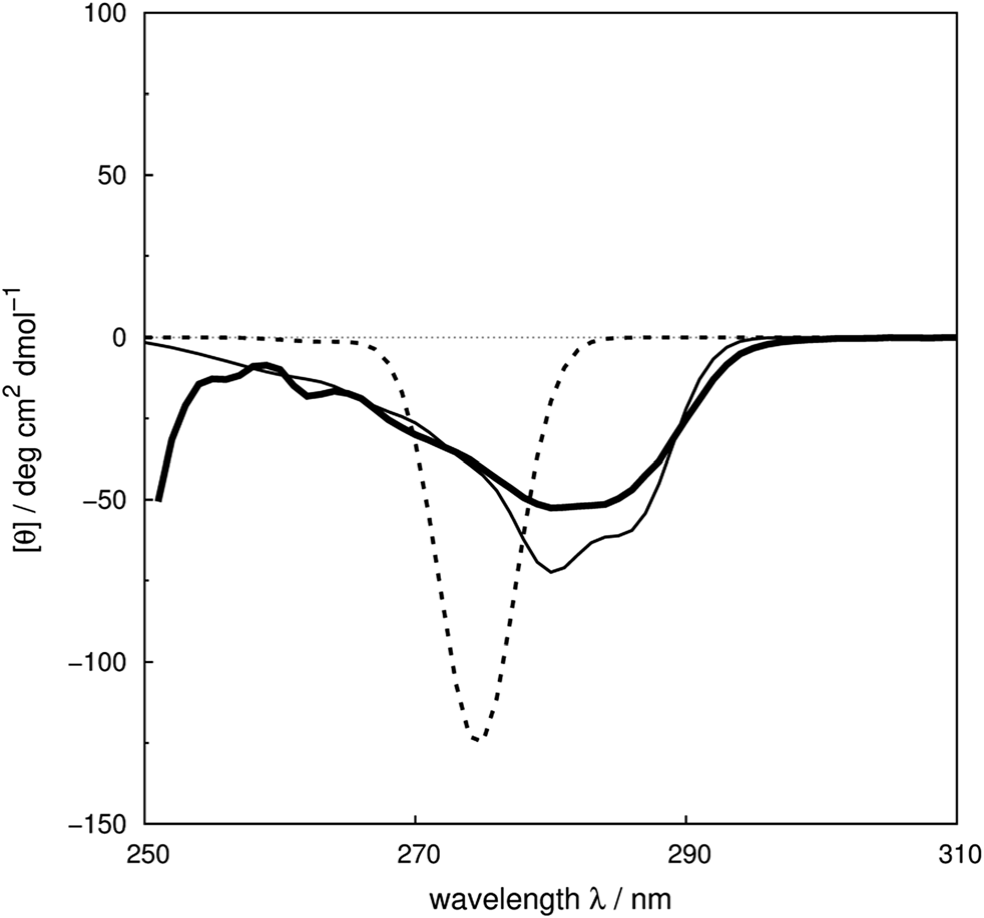
Experimental^80^ near-UV CD spectrum (bold solid line) and calculated spectra of adenylate kinase (PDB code: ; 2ECK) with ‘non-vib’ (dotted line) or ‘vib’ (thin solid line) parameter sets. The RMSE of the spectrum calculated with the ‘vib’ parameters is 11 deg cm^2^ dmol^–1^ and with the ‘non-vib’ parameters is 34 deg cm^2^ dmol^–1^.

**Fig. 5:**
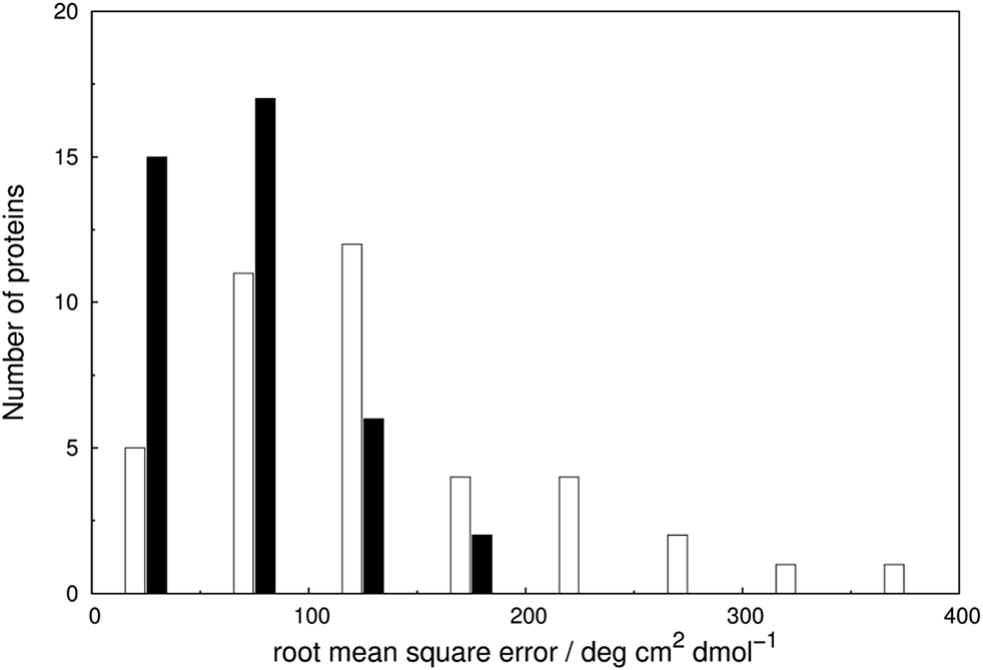
Root mean square error (RMSE) of the intensities between the experimental and calculated near-UV CD spectra, computed with ‘non-vib’ (open bar) and ‘vib’ (solid bar) parameters for the 40 proteins.

We assess the influence of the vibrational structure of the aromatic electronic transitions on the calculated CD spectra. Two parameter sets are considered corresponding to the inclusion or neglect of vibrational structure, respectively: ‘vib’ and ‘non-vib’. The calculated spectra are compared with the experimental spectra for each protein, as shown in Fig. 4 for adenylate kinase.^80^ Spectra of other proteins are given in Fig. S4 to S36.† We use the root mean square error (RMSE, Fig. 5) and the mean relative error (MRE) to assess the accuracy (Table S7†). The Spearman rank correlation between the calculated and the experimental intensities of all 40 proteins for each integer wavelength provides another useful statistic.

The RMSE between the two parameter sets are summarised in Fig. 5 and compared in detail in Table S7.† The wavelength range over which the RMSE is calculated depends on the associated published spectra. The ‘vib’ calculation gives a smaller RMSE for all the proteins except staphylococcal nuclease. For the ‘vib’ parameters, all 40 proteins have an RMSE less than 175 deg cm^2^ dmol^–1^, in contrast to a broader distribution of RMSE for the ‘non-vib’ calculations. The analogous distributions of mean absolute error (data not shown) are quantitatively similar to the RMSE. Half of the proteins show an improvement in RMSE of over 40% with the ‘vib’ parameters. The RMSE is reduced by over 150 deg cm^2^ dmol^–1^ in the case of five proteins: chymotrypsinogen A (Fig. S13†), human carbonic anhydrase II (Fig. S18†), papain (Fig. S25†), phospholipase A2 (Fig. S28†) and rhodanese (Fig. S30†).

**Fig. 6:**
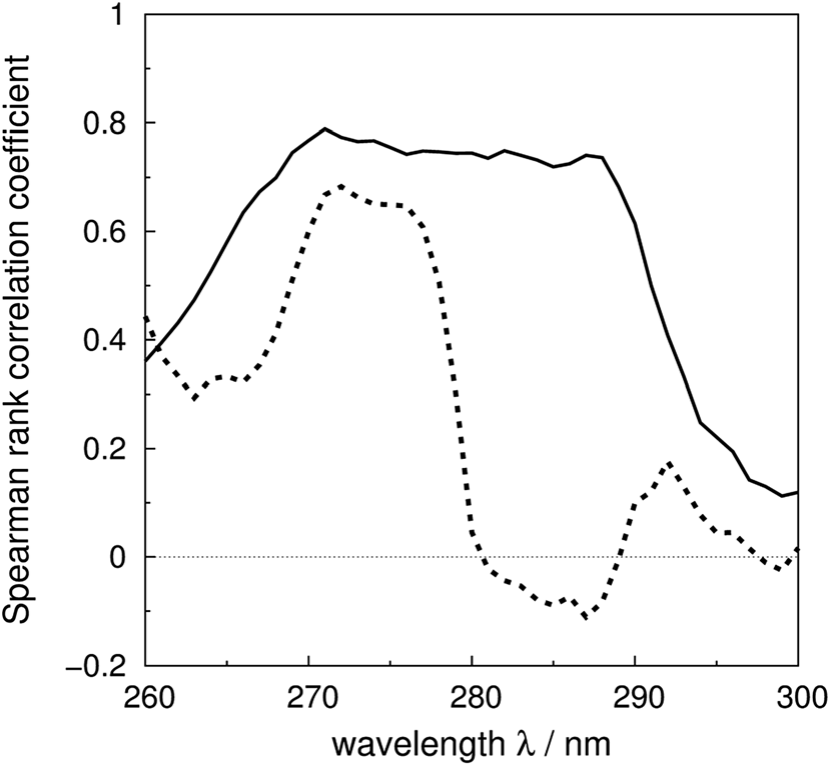
Spearman rank correlation coefficients between the calculated and experimental intensity for the ‘vib’ (solid line) and the ‘non-vib’ (dotted line) parameter sets, based on 40 proteins.

The Spearman rank correlation coefficients between the calculated and the experimental mean residue ellipticities are compared in Fig. 6 for the ‘non-vib’ and ‘vib’ parameters. The wavelengths covered are from 260 to 300 nm. There are limited experimental spectra beyond this range; the correlation coefficients between 260 and 264 nm are calculated without myoglobin and bovine pancreatic trypsin inhibitor, as experimental data were not available from the literature. The current wavelength range encompasses the excitation energies of the origins and the transitions with the largest Franck–Condon integrals of all three aromatic amino acids. The ‘vib’ parameter set gives greater correlation across nearly the whole wavelength range. The correlation coefficients increase by over 0.7 between 281 and 288 nm. The lack of correlation above 290 nm may be due to neglect of the influence of the environment on the excitation energy of tryptophan. An energy shift was calculated with a model considering the electrostatic interaction between the chromophore and the surrounding atoms.^81^ However, inclusion of this effect did not noticeably improve the accuracy of the calculations (data not shown).

### Illustrative spectra

**Fig. 7:**
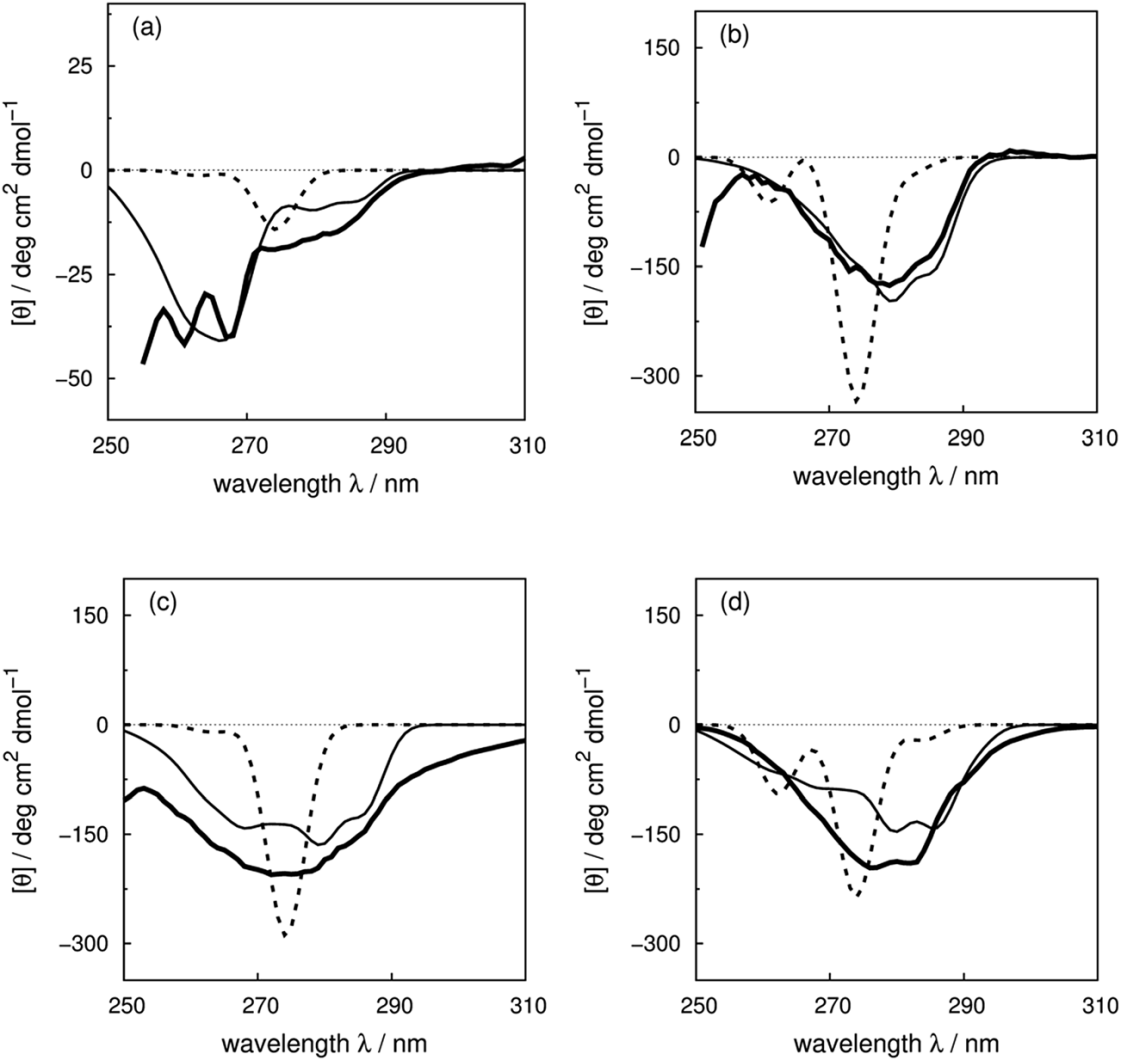
Experimental near-UV CD spectra (bold solid line) and calculated spectra with ‘non-vib’ (dotted line) or ‘vib’ (thin solid line) parameter sets: (a) calmodulin^9^ (PDB code: ; 4CLN), (b) subtilisin BPN′^35^ (PDB code: ; 1ST2), (c) insulin^82^ (PDB code: ; 5ENA), and (d) odorant binding protein^83^ (PDB code: ; 1A3Y).

We use four proteins from the set of 40 with various numbers of tryptophan residues and disulfide bonds to illustrate the effectiveness of the ‘vib’ parameter set in the CD calculations. *Drosophila melanogaster* calmodulin with calcium bound (Fig. 7a) has no tryptophans or disulfide bonds.^9^ The negative bands at 262 and 268 nm are from the nine phenylalanine residues in the protein, while its single tyrosine leads to a less intense broad band at a longer wavelength. Compared to the ‘non-vib’ parameters, which predicted a signal at 274 nm from the tyrosine, the calculation considering the vibrational structure locates the bands for the tyrosine and the phenylalanines at the same wavelengths as the experimental spectrum, with an average error in the intensity of less than 10 deg cm^2^ dmol^–1^ from 255 to 300 nm. *E. coli* adenylate kinase (Fig. 4) and *E. coli* tryptophan synthase α-subunit (Fig. S36†) are also proteins without tryptophans or disulfide bonds. The experimental spectrum of adenylate kinase,^80^ which shows little vibrational fine structure, is reproduced quantitatively with the ‘vib’ parameters. Tryptophan synthase α-subunit has a distinctive near-UV CD spectrum with both positive and negative bands,^34^ while our calculation predicts an intense negative broad band with shifted peak positions. This protein has 20 pairs of aromatic rings in close proximity (less than 8 Å) which lead to strong couplings.

*Bacillus amyloliquefaciens* subtilisin BPN′ (Fig. 7b) consists of 275 residues including three phenylalanines, ten tyrosines, three tryptophans and no disulfide bonds.^35^ The broad negative band at 278 nm in the experimental spectrum has no distinguishable fine structure and is calculated quantitatively at 279 nm with the ‘vib’ parameters, but less accurately at 274 nm with the ‘non-vib’ parameters. Both tyrosine and tryptophan contribute to this band. The well-reproduced rising edge of the experimental spectrum above 286 nm is solely from tryptophan, since there are no disulfide bonds. This level of accuracy reflects the effectiveness of the new parameters in describing tryptophans. Dehydroquinase II (Fig. S14†) and α-lactalbumin (Fig. S5†) are further similar examples. Some proteins, including barnase (Fig. S7†), human carbonic anhydrase II (Fig. S18†), rhodanese (Fig. S30†) and phosphatidylethanolamine-binding protein (Fig. S27†), have distinct peaks or shoulders of moderate intensity above 290 nm. These features must arise from tryptophans, since there are no other chromophores that have CD signals in this region.

Disulfide bonds forming between the side chains of two cysteines are another contributor to the near-UV protein CD spectra, which is currently omitted in our calculations. The CD bands arising from disulfide bonds are much broader than those of the aromatic amino acids appearing between 240 and 350 nm.^82^ Human insulin^82^ (Fig. 7c) and porcine odorant binding protein^83^ (Fig. 7d) illustrate the contribution of the disulfide moieties. Human insulin has three disulfide bonds and no tryptophans. Tyrosine is the only aromatic amino acid that contributes to the spectrum above 270 nm in the calculation. Insulin exists mostly as a dimer when there are no stabilizing moieties, such as Zn^2+^, phenol or glycerol, which is the case in our calculations; otherwise insulin is in an inactive hexamer form. The experimental spectrum of insulin is an intense broad negative band without clear fine structure. The two calculated extrema at 280 and 286 nm quantitatively match the rising edge at the same wavelengths which arises from a combination of individual bands of the four tyrosines. However, the broad band above 290 nm is not generated, due to the absence of the transitions of the disulfide bond in the calculation. The experimental and calculated spectra of bovine pancreatic trypsin inhibitor^5^ (Fig. S11†) and bovine ribonuclease A^7^ (Fig. S32†) are very similar to those of insulin. The overall shapes of the experimental spectra of these two proteins are reproduced well, especially for the region where only tyrosine contributes.

The assessment of the calculations becomes more complicated when all three aromatic chromophores and disulfide bonds are present. Fig. 7d displays the experimental and calculated CD spectra of porcine odorant binding protein.^83^ There are two negative peaks with comparable intensities at 276 and 282 nm and a shoulder at 290 nm in the experimental spectrum. Our calculation reproduced peaks at 276 nm and 282 nm, which arise from both tyrosines and tryptophans, although approximately 15 deg cm^2^ dmol^–1^ less intense and red-shifted by about 4 nm compared to the experiment. The shoulder at 290 nm is predicted by the calculation with a 17 deg cm^2^ dmol^–1^ discrepancy at the correct wavelength. Other examples in the dataset where the vibrational fine structure in the experimental spectra is well reproduced with the ‘vib’ parameters include papain (Fig. S25†), thioredoxin (Fig. S35†), phospholipase A2 (Fig. S28†), ribonuclease T1 (Fig. S31†) and pectate lyase C (Fig. S26†). However, the calculated intensities are not well estimated for some of these proteins. One reason may be the omission of the disulfide bond in our calculation. The band shapes and intensities vary widely depending on conformation^84–86^ and vicinal interactions.^87^ However, there must be other explanations for the band shift and the intensity difference for proteins without disulfide bonds; such cases include dihydrofolate reductase (Fig. S15†), rhodanese (Fig. S30†) and phosphatidylethanolamine-binding protein (Fig. S27†).

Solvent is a universal factor that affects both the excitation of the aromatic chromophores and the structure of the protein. The influence of solvent on the ground and the excited state orbitals is evident in the difference in the absorption and CD spectra measured in gas phase and in various solutions.^46^ Another consequence is broadened spectra, caused by interactions of varying degrees between the chromophore and surrounding solvent molecules. There are many environments in proteins for aromatic residues, spanning a range from buried to solvent exposed. These are currently omitted in our calculations; as noted earlier, exploratory calculations to model these effects did not yield more accurate calculated CD spectra and this will be addressed in future work.

### CD spectra of mutants

Comparison of the CD spectra of a wild-type protein and its mutants can reveal the contributions of individual aromatic residues to the spectrum. This kind of data can be difficult to extract directly from experimental spectra of wild-type proteins due to the complexity caused by a combination of multiple contributors. Tryptophan and tyrosine are often mutated to phenylalanine, while phenylalanine is usually mutated to leucine as the similar volume of the side chain minimizes the perturbation of the overall structure of the protein and no other ambiguous features should appear in the CD spectra of the mutants. The availability of CD spectra of mutants allows us to assess the effectiveness of our new parameters.

### Barnase

**Fig. 8:**
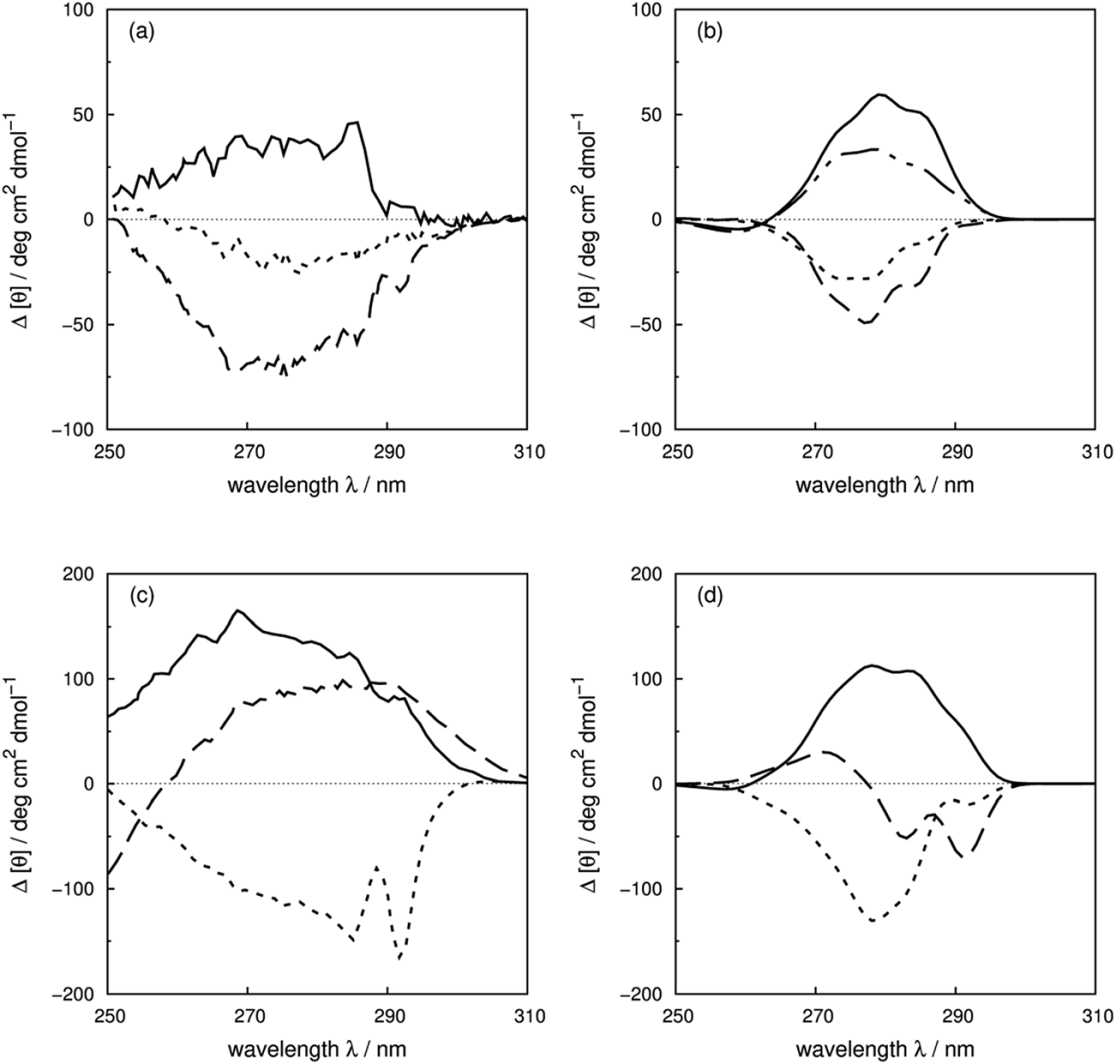
Difference spectra (wild-type minus the mutants) of barnase (experimental: (a) and (c); calculated: (b) and (d)). (a) and (b) ΔY78F (solid line), ΔY90F (dotted line), ΔY97F (dashed line); (c) and (d) ΔW35F (solid line), ΔW71F (dotted line), and ΔW94F (dashed line). The dotted-dash line in (b) is the difference spectrum calculated with the X-ray structure of Y78F barnase (PDB: 1BAO).

Vuilleumier *et al.* ^7^ have reported CD spectra of three tyrosine and three tryptophan mutants of barnase, which is a homo-trimer. The monomer consists of 110 residues including four phenylalanines, seven tyrosines and three tryptophans. The three chains in the crystal structure (PDB: ; 1A2P) adopt essentially identical core conformations.^88^ However, the residues on surface show some flexibility, which is consistent with the NMR structure (PDB: ; 1FW7).^89^ Comparable calculated spectra (Fig. S7†) confirm the similarity of the crystal and NMR structures. The ^17^Tyr residue adopts distinct conformations in the three chains of the crystal structure. The multimeric structure is used in the calculations of both wild-type and mutants. The predominant changes in the experimental spectra arising from the tyrosine mutations appear between 260 and 290 nm, the region in which tyrosine chromophore absorbs. The trend in intensities is predicted well by calculations (Fig. S37a and b†) across the mutants, although the intensities are underestimated for all the calculated spectra compared to their experimental counterparts. The intensity changes are reproduced quantitatively for all three tyrosine mutants above 270 nm in this protein (Fig. 8b). Both ^1^L_b_ states of tyrosine and phenylalanine contribute to the region below 270 nm where the calculated spectra did not predict the changes of Y78F and Y97F observed in the experimental spectra.

Our theoretical model is sensitive to subtle ring flips. There is an X-ray structure of the Y78F mutant of barnase (PDB: 1BAO) whose calculated spectrum is compared in Fig. 8b with the results calculated using a structure generated by deleting the OH group from the wild-type structure. Both structures give a positive band. The X-ray structure gives a difference spectrum slightly closer to the experimental one. Table 4 compares the differences in key residue–residue interactions (distances and transition dipole moment orientations) of two Y78F mutants (the X-ray determined structure and the one derived from deleting the OH group directly) for residues less than 8 Å apart, which might be considered to be strongly interacting. The nearest aromatic residue to ^78^Tyr is ^35^Trp at 8.8 Å, which means that the intensity changes between the wild-type and mutated barnase are predominantly from the tyrosine. Whilst the various pairwise distances are very similar, the relative orientations of ^13^Tyr–^17^Tyr, ^56^Phe–^103^Tyr and ^103^Tyr–^106^Phe appear to be better represented by the X-ray structure of the mutant than the mutant structure modelled from the wild-type.

**Table 4:** Comparison of distances and angles between transition dipole moments (TDM) of residue pairs with strong interactions. The distance is measured between the centroids of the aromatic side chains. The structures used in the calculations were generated by directly deleting the hydroxyl group from ^78^Tyr of wild-type barnase (; 1A2P–Y78F) or taken from the structure of the X-ray structure mutant (; 1BAO)

Residues	Distance/Å		Angle between TDMs/degree			
	1A2P–Y78F	1BAO	1A2P–Y78F		1BAO	
			^1^L_b_	^1^L_a_	^1^L_b_	^1^L_a_
^13^Tyr–^17^Tyr	4.2	4.4	115		83	
^56^Phe–^71^Trp	7.5	7.7	45	53	43	61
^56^Phe–^103^Tyr	5.6	5.3	44		151	
^56^Phe–^106^Phe	6.2	6.2	68		74	
^71^Trp–^97^Tyr	6.6	6.5	108	86	114	82
^90^Tyr–^94^Trp	7.0	7.0	86	173	87	168
^97^Tyr–^106^Phe	7.4	7.4	118		113	
^103^Tyr–^106^Phe	7.3	7.1	111		80	

For the tryptophan mutants of barnase, the trend of intensity changes is predicted well by the calculations (Fig. S38a and b†), although the calculated contribution of ^94^Trp agrees less well with experiment above 270 nm. ^35^Trp and ^71^Trp are more buried, with solvent accessible areas of 18 and 8 Å^2^, compared to 80 Å^2^ for ^94^Trp. This explains the more obvious vibrational features of ^35^Trp and ^71^Trp in the difference spectra (Fig. 8c). The contributions of ^35^Trp and ^71^Trp are quantitatively reproduced around 285 nm, the origin of the ^1^L_a_ state of tryptophan in our parameters. The ^1^L_b_ transition of ^71^Trp, which is the intense transition for this residue,^7^ is underestimated by our calculation. The calculated spectrum of W94F gave a band with the opposite sign compared to the experimental spectrum above 270 nm. This may be related to the larger solvent accessible area; also ^94^Trp is not in a well-defined secondary structure and the surrounding local structure may be sensitive to mutation.

### BPTI

**Fig. 9:**
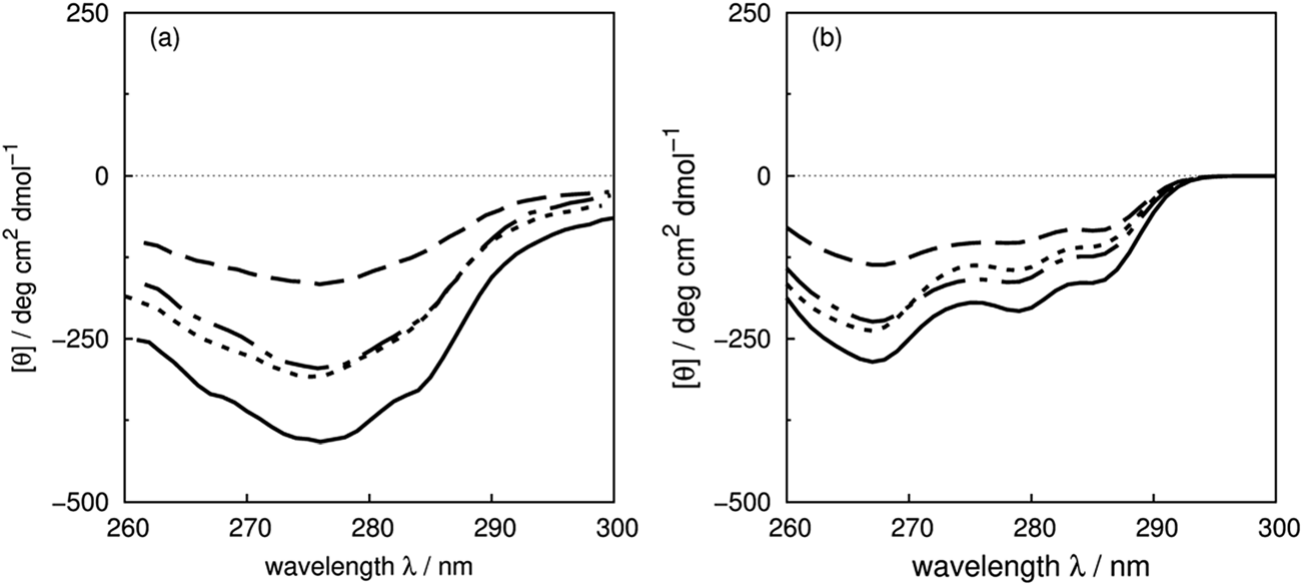
(a) Experimental CD spectra of wild-type bovine pancreatic trypsin inhibitor (solid line) and mutants F22L (dashed line), F45L (dot-dashed line) and Y23L (dotted line), and (b) calculated CD spectra from X-ray structures: wild-type (PDB: 5PTI, solid line), F22A (PDB: ; 1BTI, dashed line), F45A (PDB: ; 1FAN, dot-dashed line) and Y23A (PDB: ; 1BPT, dotted line).

Sreerama *et al.* ^6^ have systematically studied the contributions of four phenylalanines and four tyrosines in bovine pancreatic trypsin inhibitor (BPTI) in the far- and the near-UV region, by replacing them with leucine. The CD spectra of the BPTI mutants show distinct changes in intensities and peak positions in the far-UV, which may come from the contributions of the mutated residues or secondary structure changes associated with the mutations. There are indeed some conformational differences among the X-ray structures of BPTI mutants: F22A (PDB: ; 1BTI), F45A (PDB: ; 1FAN) and Y23A (PDB: ; 1BPT). The RMS distances are 0.92, 0.59 and 0.25 Å between the wild-type and the mutated structures, respectively. These three aromatic residues are buried; the solvent accessible surface areas are 22, 16 and 15 Å^2^, respectively.^6^ ^22^Phe and ^23^Tyr are in a β-strand, while ^45^Phe is in a β-bridge. Calculations with the crystal structures of the mutants are compared with experimental spectra of the wild-type and mutants of F22L, F45L and Y23L in Fig. 9, which shows that the trend of the intensity changes is reproduced well.

### RNase A

**Fig. 10:**
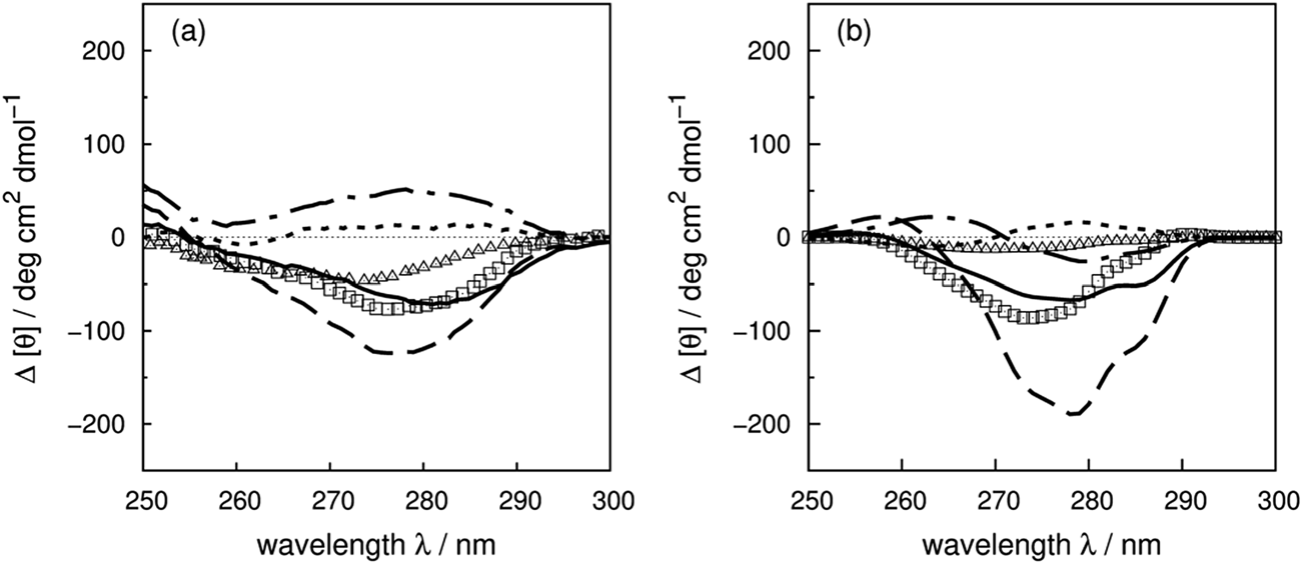
Difference spectra (wild-type minus the mutants) of RNase A ((a) experiment, (b) calculation). ΔY25F (solid line), ΔY73F (dashed line), ΔY76F (dotted line), ΔY92F (triangle), ΔY97F (dot-dashed line) and ΔY115F (square).

Woody and Woody^8^ have studied the contributions of tyrosines in the CD spectrum of ribonuclease A (RNase A) by replacing tyrosine with phenylalanine. There is no obvious secondary structure change, as shown in the far-UV spectra of the wild-type protein and the mutants.^7^ Fig. 10 compares the experimental and calculated changes arising from the mutations. The contributions of ^25^Tyr, ^76^Tyr and ^115^Tyr are reproduced quantitatively. The calculated shift of 6 nm of the intense peaks of ^25^Tyr and ^115^Tyr agrees well with the 5 nm shift observed experimentally. The contribution of ^73^Tyr is overestimated, while that of ^92^Tyr is underestimated but has the right sign. There are two pairs of interactions, ^73^Tyr–^115^Tyr and ^25^Tyr–^46^Tyr, with respective separations of 5.2 and 5.7 Å, in RNase A, which explains the intense contributions of ^25^Tyr, ^73^Tyr and ^115^Tyr. The calculated contribution due to ^97^Tyr has the wrong sign between 270 and 290 nm. The hydroxyl group of ^97^Tyr forms a hydrogen bond with the carbonyl oxygen of ^41^Lys which is in the binding pocket. The activity of this protein is affected by replacing ^97^Tyr with phenylalanine.^8^ This may indicate a conformational change of local structure of the binding site, which may be missing from our modelled structure of the mutant and may explain the discrepancy between the calculated and experimental spectra of mutant Y97F.^90^

### Human carbonic anhydrase II

**Fig. 11:**
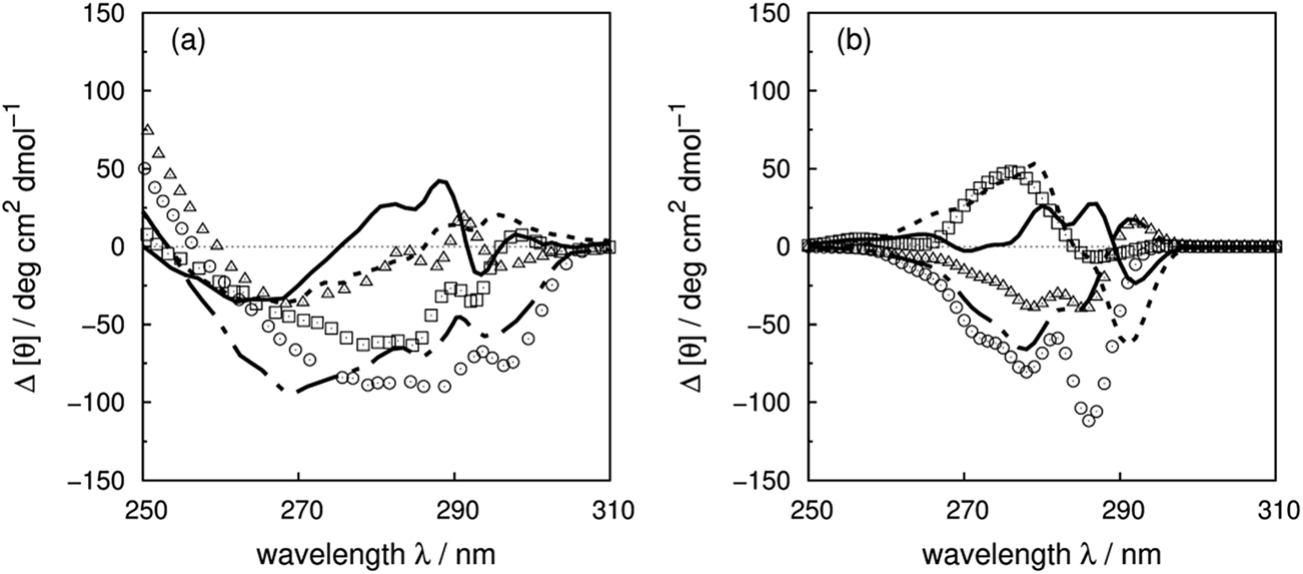
Difference spectra (wild-type minus the mutants) of human carbonic anhydrase II ((a) experiment, (b) calculation); ΔW5F (solid line), ΔW16F (square), ΔW97C (circle), ΔW123C (triangle), ΔW209F (dotted line) and ΔW245C (dot-dashed line).

The contributions of tryptophan to the CD spectrum of human carbonic anhydrase II (HCAII) have been studied by Freskgård *et al.* ^5^ through site-directed mutagenesis. The mutants are generated from a pseudo-wild-type HCAII, in which ^206^Cys has been mutated to ^206^Ser to eliminate the potential disulfide bond formation between this residue and the cysteine used to replace tryptophan. All tryptophans in HCAII have at least one other aromatic residue within 8 Å which leads to complex spectra. Fig. 11 shows the experimental and calculated difference spectra for the wild-type HCAII and the tryptophan mutants (experimental and calculated spectra of the wild-type and the mutants are shown in Fig. S39†). ^97^Trp and ^245^Trp have the largest contributions to the near-UV CD spectrum which is reproduced well by the calculation. The extrema at 287 nm in the experimental spectra are slightly overestimated by the calculation. The extrema at 296 nm, which indicate a significant shift in the transition energy due to the environment, are not reproduced.

The 0–0 transitions are at 34 424 and 35 088 cm^–1^ (290.5 and 285.0 nm) for the ^1^L_b_ and ^1^L_a_ states of 3-methylindole, respectively. The contribution of ^97^Trp, which is reproduced well, shows clear vibrational fine structure due to the buried environment. However, the shifts of ^97^Trp and ^245^Trp spectra due to the various local environments are not reproduced by the current calculations. ^5^Trp and ^16^Trp at the N-terminus are separated from most of the aromatic residues, except ^7^Tyr and ^20^Phe. The contribution of ^5^Trp is reproduced well both for the intensity and the peak position. However, the calculation of ^16^Trp gives the opposite sign to the experimental spectrum. This may be caused by local conformational changes of ^16^Trp mutants due to the loss of the hydrogen bonds between the indole ring and the carbonyl oxygen on ^7^Tyr and ^11^Asn. W209F is the other mutant where the calculation does not reproduce the experiment well. ^209^Trp is part of the active site where Zn^2+^ is bound to HCAII. Mutation of ^209^Trp decreases the activity by 66% and may indicate a less tight binding mode of Zn^2+^ and a weaker interaction between ^209^Trp and the ion.^91^ This may cause a conformational change in the protein.

### Influence of conformational dynamics

**Fig. 12:**
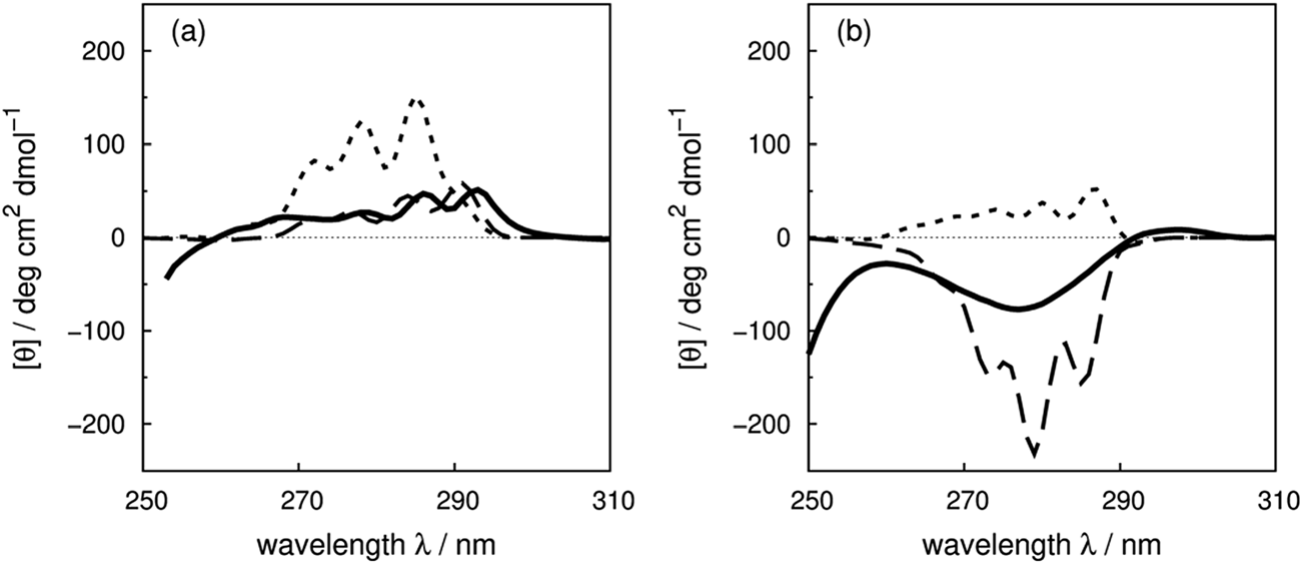
Comparison between the experimental spectrum (solid line) and calculated spectrum with ‘vib’ parameters from the X-ray structure (dotted line) and the NMR structures (dashed line) of (a) apolipophorin III^92^ and (b) staphylococcal nuclease.^93^

Compared to the crystal structure determined by X-ray diffraction, protein structures obtained by solution NMR contain information about the conformational dynamics. Calculations with an ensemble of NMR structures can account for some of the conformational flexibility in solution. The NMR structures for 13 of the proteins in our dataset (Table 1) are available in the PDB.^61^ The influence of the structures used in the calculation (X-ray *vs.* NMR) to the calculated spectra is similar for both ‘non-vib’ and ‘vib’ parameter sets. Combination of the NMR structures with the ‘vib’ parameters better reproduced the experimental spectra than with the ‘non-vib’ parameters. Comparison between calculations with X-ray and NMR structures of apolipophorin III^92^ (Fig. 12a) and staphylococcal nuclease^93^ (SNase, Fig. 12b) illustrates the influence of taking multiple conformations into account on the calculated intensity. Calculations considering a family of conformations also better predict the intensities of BPTI (Fig. S11†) and thioredoxin (Fig. S35†). Compared to the calculations with a crystal structure, the average intensity calculated over multiple frames can be stronger or weaker. For BPTI and thioredoxin, the spectra calculated from individual frames vary only in intensity, without significant changes in the sign of bands or the overall shape compared to calculations on crystal structures. This reflects the minor differences in conformations between NMR and X-ray structures. Calculations on NMR ensembles of other proteins show greater variation in band shapes due to distinct conformations.

The spectrum of apolipophorin III^92^ (Fig. 12a) calculated from the NMR structures compared to the spectrum from the X-ray structure is less intense and red-shifted by approximately 6 nm and is much closer to the experimental spectrum. For SNase^93^ (Fig. 12b), spectra calculated using individual frames from the NMR structures all show a broad negative band at 279 nm with an intensity ranging from –310 to –50 deg cm^2^ dmol^–1^; the opposite sign is obtained using the X-ray structure. The X-ray structure of SNase is five residues shorter than the NMR structure at the N-terminus due to a lack of electron density in the X-ray structure determination;^94^ ^141^Ser is the last residue at the C-terminus in the X-ray structure, whereas it is ^140^Trp in the NMR structure (denoted as SNase140). However, there are no missing aromatic amino acids in the truncated sequence. A study of SNase folding has highlighted the role of ^141^Ser in maintaining the native structure and fluorescence spectra of SNase truncated at ^140^Trp and ^141^Ser show variation in the conformation of ^140^Trp.^95^ Calculations based on NMR structures do not always reproduce the experimental spectrum better than the X-ray structure. Such cases include lysozyme (Fig. S17†), interleukin 6 (Fig. S21†), monellin (Fig. S22†) and ribonuclease T1 (Fig. S31†). The influence of conformational dynamics could also be explored using molecular dynamics simulations. Some individual cases have been studied^96–98^ and we are planning a more comprehensive investigation.

### CD calculation of native proteins and their decoys

In the final part of our study, we investigate to what extent a comparison between the computed and experimental near-UV CD spectra can be used to identify a native structure from a large number of plausible but incorrect model structures. CD spectra of decoys were calculated with the vibrational parameters and compared with the experimental spectra of the native proteins. The evenness and the diversity scores of the generated decoys show the quality of the decoy sets (Table S9†). To assess the similarity of two spectra, we use the Spearman rank correlation coefficient between the computed and experimental intensities at integer wavelengths (in nm) across the near-UV range (Table 5). This measure gives a useful indication of the overall shape and qualitative features of the spectra. For 17 out of the 34 proteins, the Spearman rank correlation between the computed and experimental spectra of the native structure is in the top 15% of all the evaluated models. The native structure of ribonuclease T1, papain and adenylate kinase are in the top 1%. The rank of the native structure is related to its actual value of the Spearman rank correlation and the distribution patterns of the Spearman rank correlation of the decoys which are different for various proteins. Four histograms are shown in Fig. S40 to S43† to represent the distinctive behaviour of the Spearman rank correlation distribution of decoys.

**Table 5:** The 34 proteins studied: name, PDB codes, Spearman rank correlation of the native structure (experimental *vs.* calculated intensities, in integer intervals between 250 nm or the lowest available signals and 300 nm) and its location (percentile) in the distribution generated by the decoy sets

		Spearman rank correlation	Rank in decoys/%
Adenylate kinase	2ECK	0.89	1
Calmodulin	4CLN	0.93	1
Papain	3LFY	0.91	1
Ribonuclease T1	1RN1	0.75	1
Sticholysin II	1O72	0.81	5
Apolipophorin III	1AEP	0.71	6
Human carbonic anhydrase II	2CBA	0.77	6
Phospholipase A2 (Ca^2+^)	1PSJ	0.87	6
Ribonuclease A	1AFU	0.96	6
β-Lactamase	1BTL	0.93	8
Insulin	5ENA	0.93	8
Hen egg white lysozyme	1HF4	0.76	9
Subtilisin BPN′	1ST2	0.89	9
Neocarzinostatin	1NOA	0.81	10
Phosphatidylethanolamine-binding protein	1A44	0.78	10
Dihydrofolate reductase	4P3Q	0.63	13
Relaxin	6RLX	0.82	15
Monellin	1IV9	0.59	21
Extracellular domain of human tissue factor	2HFT	0.66	25
Bovine pancreatic trypsin inhibitor	5PTI	0.66	29
Thioredoxin	2TRX	0.67	29
Odorant binding protein	1A3Y	0.76	34
α-Lactalbumin	1A4V	0.2	37
Rhodanese	1DP2	–0.1	41
β-2 microglobulin (human)	1LDS	0.14	45
Barnase	1A2P	0.07	46
Interleukin 6	1ALU	–0.38	48
Chymotrypsinogen A	2CGA	–0.08	53
Dehydroquinase II	2BT4	0.01	54
Cardiotoxin	4OM4	–0.47	71
Myoglobin (whale)	1UFP	–0.8	73
Staphylococcal nuclease	1STN	–0.39	81
Interleukin 4 (cytokine)	2B8U	–0.43	90
Tryptophan synthase α-subunit	1WQ5	–0.74	100

## Conclusions

In this work, the vibrational structure of electronic transitions of aromatic amino acids is incorporated into the calculations of the near-UV CD spectra of proteins by extending the matrix method approach to include new parameterizations of chromophores based on the Franck–Condon integrals of toluene, *p*-cresol and 3-methylindole. Qualitative and quantitative improvements in the computed near-UV CD spectra of proteins have been achieved through the first systematic inclusion of vibrational structure. Calculations on site-directed mutants give good predictions of the contribution of individual aromatic residues when the conformation of the protein is not severely affected by the mutation. Further improvements are obtained for some proteins by computing with the NMR determined structure which emphasizes the important role of conformation diversity. Calculations with our new vibrational parameters predict in half of the cases (34 proteins in total) that the native structures of those proteins are in the top 15% Spearman rank correlation among 1000 computer-generated misfolded structures, which shows the potentially useful ability to distinguish between native and incorrect protein structures. We will be developing the work further, for example, by generating parameters for disulfide bonds and by further consideration of the influence of solvent and conformational dynamics.
