# Supplementary material for: Quantitative first principles calculations of protein circular dichroism in the near-ultraviolet
Source: Chem Sci. 2017 Mar 24;8(6):4318–33. doi: 10.1039/c7sc00586e (PMC5637123; doi:10.1039/c7sc00586e)
Supplement: Supplementary file 1 [file SC-008-C7SC00586E-s001.pdf]

Electronic Supplementary Material (ESI) for Chemical Science.
This journal is © The Royal Society of Chemistry 2017

Supporting Information: Vibrational structure in the near-ultraviolet electronic circular dichroism spectra of proteins

Zhuo Li and Jonathan D. Hirst*

School of Chemistry, University of Nottingham, University Park, Nottingham NG7 2RD, United Kingdom

*jonathan.hirst@nottingham.ac.uk

Calculated geometries and vibrational frequencies assignment.

Toluene. Our calculated geometry (Table S1) and vibrational frequencies (Table S2) of the ground and the first excited states of toluene are in good agreement with the experimental[1-4] and previous calculated results.[5] The assignment of some vibrational modes differs from the previous study.[3,6] The C-H bending mode (1163 cm^{-1}) in our calculation was assigned to the band at 342 cm^{-1} in the experiment by Varsanyi[6] and to the band at 1080 cm^{-1} by Pitzer and Scott[4]. There are some discrepancies in Varsanyi's and Pitzer's assignments. Our assignments match better with Varsanyi's. The vibrational motion at band 1328 cm^{-1} in the ground state appears at both bands 1893 cm^{-1} and 1262 cm^{-1} in the excited state, which refer to the C-C and C-H stretches, respectively. The vibrational modes assigned to the bands at 1561 cm^{-1} and 1566 cm^{-1} in the excited state appear at 1569 cm^{-1} and 1648 cm^{-1} in the ground state.

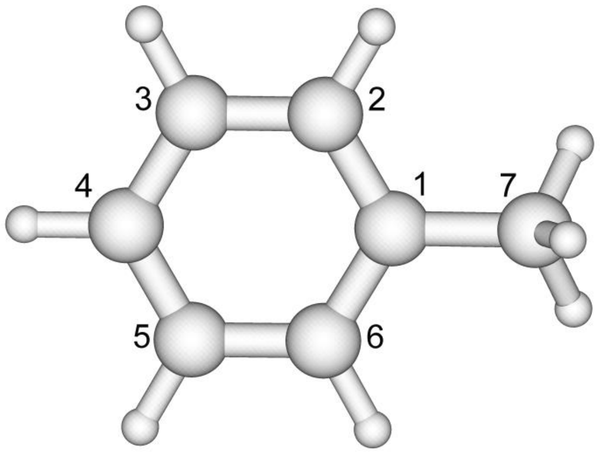

Figure S1. Structure of toluene and numbering convention of the non-hydrogen atoms.

Table S1. Geometries from CASSCF calculations of the ground (S_0) and the first (^1L_b) excited state of toluene compared to their experimental counterpart and previous CASSCF calculations with a smaller active space.

bonds	S_0 geometry / Å			^1L_b geometry / Å	
	Calc.	Expt.[1]	Lit. Calc.[5]	Calc.	Lit. Calc.[5]
C1-C2	1.401	1.399	1.396	1.439	1.472
C2-C3	1.396	1.399	1.398	1.433	1.474
C3-C4	1.396	1.399	1.378	1.432	1.428
C4-C5	1.396	1.399	1.398	1.432	1.359
C5-C6	1.396	1.399	1.394	1.433	1.430
C1-C6	1.401	1.399	1.405	1.439	1.478
C1-C7	1.513	1.511	1.511	1.504	1.497
C2-H	1.076	1.098	1.074	1.074	1.072
C3-H	1.076	1.098	1.074	1.073	1.071
C4-H	1.075	1.098	1.073	1.073	1.074
C5-H	1.076	1.098	1.073	1.073	1.074
C6-H	1.076	1.098	1.075	1.074	1.072
C7-H	1.085	1.12	1.083	1.085	1.084
C7-H	1.085	1.12	1.085	1.085	1.085
C7-H	1.087	1.12	1.085	1.089	1.090
angles	S_0 geometry / degree			^1L_b geometry / degree	
C1-C7-H	111.3	-	111.3	111.3	111.3

Table S2. Calculated (unscaled CASSCF) vibrational frequencies of the ground (S_0) and the first (^1L_b) excited state of toluene compared to their experimental counterpart and previous scaled CASSCF calculations with a smaller active space.

symmetry	assignment	S_0 frequency / cm^{-1}				^1L_b frequency / cm^{-1}		
		Calc.	Expt.		Lit.	Calc.	Expt.[3]	Lit.
			Expt.[2]	Expt.[4]	Calc.[a,5]			Calc.[a,5]
In Plane								
a_1	C-C stretch	1755	1606	1605	1601	1695	-	1636
	C-C stretch	1648	1497	1494	1506	1561/1566	-	-
	C-H bend (CH_3)	1637	1497	-	1513	1632	-	1504
	C-H bend (CH_3)	1569	-	-	-	1561/1566	-	-
	C-CH_3 stretch	1312	1211	-	1214	1286	-	1178
	C-H bend	1281	1182	1175	1197	1250	1021	1203
	C-H bend	1107	-	1030	-	978	935	-
	C-C bend	1069	1005	1003	1030	1035	966	977
	C-C bend	838	788	785	779	787	736	555
	C-C bend	555	522	521	522	503	457/462	459
b_2	C-C stretch	1724	1587	1586	1587	1667	-	1520
	C-H bend (CH_3)	1642	1497	-	1513	1625	-	1504
	C-C stretch	1586	1445	1445	1455	1519	-	1451
	C-H bend	1469	-	-	-	1435	-	-
	C-H bend	1328	-	1312	-	1893/1262	-	-
	C-H bend	1225	1157	1155	1153	1139	-	1126
	C-H bend	1163	1084	1080	1090	1139	-	1058
	C-CH_3 bend	1074	-	-	-	986	-	-
	C-C bend	668	622	623	637	588	532	721
	C-CH_3 wag	362	348	342	354	349	332	296
Out of plane								
b_1	C-H bend (CH_3)	1149	1041	-	1078	1110	-	1012
	C-C bend	1029	978	978	1032	711	-	1005
	C-H bend	931	898	895	904	647	-	519
	C-H bend	768	732	728	733	562	-	458
	C-C bend	728	698	695	698	495	423	312
	C-C bend	492	467	464	466	353	320	264
	C-CH_3 wag	221	220	216	225	158	157	85
a_2	C-C bend	998	967	964	966	653	687	790
	C-H bend	881	841	843	836	583	-	471
	C-C bend	436	407	407	409	283	228	161
Other modes								
	C-CH_3 rotation	18	-	-	-	39	-	-

a. The calculated vibrational frequencies are the scaled values reported in the literature.

***p*-cresol**. The calculated geometry (Table S3) and vibrational frequencies (Table S4) of the ground and the first excited states of *p*-cresol are compared with experiment[7,8] and previous reported calculations.[5,9] Both geometries and vibrational frequencies are in good agreement with experimental and theoretical results in the literature. DFT calculations with various sizes of basis set give similar results.[8,9] We compared our calculations with B3LYP/6-311++G** calculations (Table S4). Four vibrational modes, which were assigned to 842 cm^{-1}, 1374 cm^{-1}, 1470 cm^{-1} and 1568 cm^{-1} in the ground state, were assigned to more than one band in the excited state.

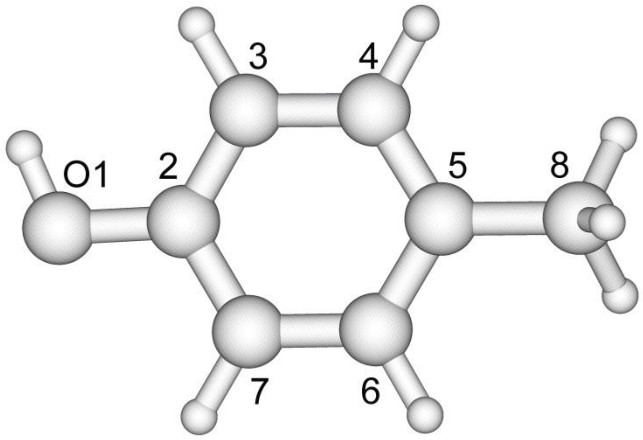

Figure S2. Structure of *p*-cresol and numbering convention of the non-hydrogen atoms.

Table S3. Geometries from CASSCF calculations of the ground (S_0) and the first (^1L_b) excited state of *p*-cresol compared to their experimental and calculated counterparts using CASSCF or DFT.

bonds	S_0 geometry / Å				^1L_b geometry / Å	
	Calc.	Expt.[7]	Calc. Lit.		Calc.	Calc. Lit.
			CASSCF[5]	B3LYP[9]		CASSCF[5]
O1-C2	1.361	1.387	1.380	1.368	1.354	1.377
C2-C3	1.392	1.397	1.371	1.399	1.429	1.358
C3-C4	1.398	1.401	1.402	1.396	1.431	1.426
C4-C5	1.397	1.391	1.392	1.403	1.439	1.476
C5-C6	1.403	1.406	1.408	1.403	1.438	1.470
C6-C7	1.393	1.397	1.387	1.396	1.434	1.473
C2-C7	1.396	1.388	1.398	1.402	1.425	1.427
C5-C8	1.513	1.520	1.511	1.511	1.504	1.497
O1-H	0.947	0.9	0.950	0.968	0.947	0.950
C3-H	1.077	1.0	1.075	1.092	0.947	1.075
C4-H	1.076	0.9	1.074	1.094	1.073	1.071
C6-H	1.076	1.0	1.074	1.094	1.073	1.071
C7-H	1.074	1.0	1.071	-	1.072	1.069
C8-H	1.085	0.9	1.085	1.103	1.085	1.084
C8-H	1.085	1.0	1.083	1.103	1.085	1.085
C8-H	1.087	1.1	1.085	1.100	1.087	1.090
angles	S_0 geometry / degree				^1L_b geometry / degree	
H-O1-C2	110.4	120.0	114.6	108.6	110.6	114.7
C5-C8-H	111.2	-	111.3	111.6	111.2	111.3
C7-C2-O1	117.6	118.7	116.3	117.8	116.8	114.0

Table S4. Vibrational frequencies from (unscaled) CASSCF calculations of the ground (S_0) and the first (^1L_b) excited state of *p*-cresol compared to their experimental counterpart and previous (scaled) calculations using CASSCF or DFT.

symmetry	assignment	S_0 frequency / cm^{-1}				^1L_b frequency / cm^{-1}	
		Calc.	Expt.[10]	Calc. Lit.		Calc.	Calc. Lit.[c,5]
				CASSCF[c,5]	B3LYP[8]		
	In Plane						
	C-C stretch	1778	1621	1624	1656	1720	1649
	C-C stretch	1677	1520	1537	1545	1590	1466
	CH_3 bend	1637	1385[b]	1436	1415	1632	1430
	C-O□ stretch	1387	1255	1245	1274	1379	1182
	C-CH_3 stretch	1310	1215[a]	1194	1229	1279	1153
a_1	C-H bend	1280	1178	1128	1195	1242	1105
	C-H bend	1099	1015	1008	1031	1014	1069
	C-C bend	890	840[b]	823	851	847	769
	C-H bend	790	739	727	745	751	576
	C-H bend	490	459	459	467	444	407
	C-C stretch	1738	-	1599	1632	1680	1525
	C-C stretch	1568	1431	1456	1456	1486/1566/1895	1450
	C-H bend	1470	1298[a]	1294	1332	1434/1486	1270
	C-H bend	1374	1337	1366	1359	1296/1895	1369
b_2	C-H bend	1194	1114	1077	1129	1156	-
	C-C bend	695	642	657	657	618	696
	C-O wag	453	420	430	426	433	414
	C-CH_3 wag	324	333	313	304	320	303
	Out of Plane						
	C-C bend	967	918[a]	936	919	634	454
	C-C bend	842	811	805	804	412/564	116
	C-C bend	721	698	698	693	488	576
b_1	C-C bend	535	503	510	513	564	244
	C-H bend	347	294	294	331	255	335
	CH wag, O-H wag	155	-	157	144	114	74
a_2	C-H bend	863	-	824	830	564	430
	C-C bend	442	-	416	419	273	225
	Other modes						
	CH_3 bend	1571	1472[a]	1513	1487	1566	1505
	O-H wag	1261	1187[a]	1214	1185	1895	1310
	CH_3 rock	1085	1105	1073	1060	1018	975
	O-H wag	259	-	-	296	247	-
	C-CH_3 rotation	25	-	55[d]	18	63	-

a. Data are from Raman vapor.[8] b. IR vapor data from different reference.[8] c. The calculated vibrational frequencies are scaled values as reported in the literature. d. This value has not been scaled.

3-methylindole. The calculated geometry (Table S5) and vibrational frequencies (Table S6) of 3-methylindole are compared with the experimental[11-14] and previous calculated results.[12,15] Our calculated geometries are in good agreement with earlier CASSCF/ANO-S calculations for all three states. The vibrational frequencies of the ground state are compared with the experimental IR frequencies and a B3LYP/TZ2P calculation. Our calculation shows good agreement. However, the frequencies are overestimated for some high energy modes. The N-H wag mode and C-CH_3 bend mode, which were assigned to bands 1420 $cm^{-1}$and 1387 cm^{-1} in the IR spectrum[13], appear together in bands 1554 cm^{-1} and 1575 cm^{-1} in the ground state as similar motions. Most of the vibrational modes in the $1L_b$ state can be matched to corresponding ground state vibrations. However, the in-plane stretch mode at 1477 cm^{-1} is assigned to two modes in the ^1L_b state (1425 cm^{-1} contains the skeletal motion while 1520 cm^{-1} contains the C-H bend motion on the benzene ring). The assignments of the ^1L_a state differ from the other two states. There are cases where a normal mode was assigned to a single band in the ground state while its vibrational character appears in two frequencies in the ^1L_a state or two or more vibrational motions are combined in a single band in the second excited state.

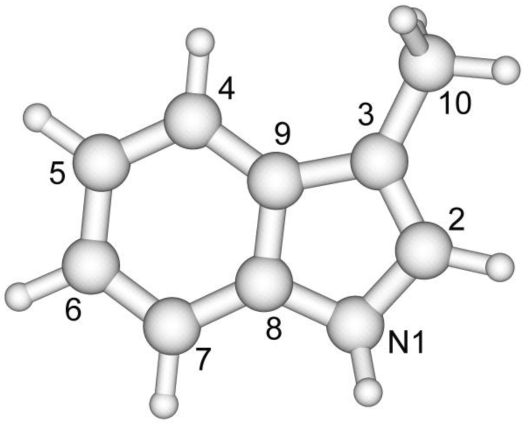

Figure S3. Structure of 3-methylindole and numbering convention of the non-hydrogen atoms.

Table S5. CASSCF calculated geometries of the ground (S_0), the first (^1L_b) and the second (^1L_a) excited state of 3-methylindole compared to experiment and previous CASSCF calculation with a smaller active space.

bonds	S_0 geometry / Å			^1L_b geometry / Å		^1L_a geometry / Å	
	Calc.	Expt.[14]	Lit. Calc.[15]	Calc.	Lit. Calc.[15]	Calc.	Lit. Calc.[15]
N1-C2	1.394	1.380	1.386	1.404	1.399	1.322	1.303
C2-C3	1.368	1.358	1.368	1.385	1.384	1.442	1.441
C3-C9	1.444	1.436	1.449	1.426	1.432	1.441	1.417
C4-C9	1.411	1.398	1.409	1.421	1.418	1.421	1.417
C4-C5	1.382	1.381	1.390	1.438	1.444	1.426	1.400
C5-C6	1.418	1.396	1.415	1.445	1.441	1.380	1.400
C6-C7	1.390	1.372	1.390	1.431	1.434	1.448	1.414
C7-C8	1.397	1.395	1.403	1.410	1.411	1.395	1.373
C8-C9	1.413	1.410	1.407	1.463	1.460	1.404	1.413
N1-C8	1.379	1.370	1.375	1.377	1.370	1.417	1.407
C3-C10	1.501	-	1.489	1.502	1.474	1.491	1.476
N1-H	0.994			0.994		0.998	
C2-H	1.071			1.069		1.068	
C4-H	1.076			1.073		1.072	
C5-H	1.075			1.074		1.077	
C6-H	1.075			1.073		1.076	
C7-H	1.076			1.074		1.073	
C10-H	1.085			1.085		1.084	
C10-H	1.086			1.086		1.086	
C10-H	1.086			1.086		1.086	
angles	S_0 geometry / degree			^1L_b geometry / degree		^1L_a geometry / degree	
C9-C8-N1-H	156.1	-	-	156.7	-	180.0	-

Table S6. Vibrational frequencies from unscaled CASSCF calculations the ground (S_0), the first (^1L_b) and the second (^1L_a) excited state of 3-methylindole compared to their experimental counterpart and scaled calculations using DFT.

assignment	S_0 frequency / cm^{-1}			Cal. frequency / cm^{-1}	
	Calc.	Expt.[13]	Calc. Lit.[13]	$^1L_{b.}$	^1L_a
In plane					
C-CH_3 wag	235	231	221	235	236
C-C bend	495	462	464	481	474
C-C bend	561	532	531	525	542
C-C bend	599	565	562	576	589
C-C bend	742	708	708	727	733
C-C stretch	806	758	762	775	784
benzene C-C bend	940	876	878	901	733
benzene C-H bend	1049	1009	1018	957	1047
C-CH_3 bend	1078	1070	1075	1055	1032
C-CH_3 stretch	1160	1070	1075	1142	1180
N1-C2 stretch	1174	1080	1090	1172	1215/1243
C-C stretch	1215	1249	1255	1255	1287
C-C stretch	1235	1249	1255	1312	1089
pyrrole stretch	1330	1229	1226	1395	1392
pyrrole stretch	1369	1302	1302	1351	1544
benzene C-H bend	1393	1334	1346	1395	1569
benzene C-H bend	1413	1334	1346	1626	1287
benzene C-H bend	1477	1345	1351	1425/1520	1392/1453
N1-H wag, C-CH_3 bend	1554	1420/1387	1424/1403	1589	1525
N1-H wag, C-CH_3 bend	1575	1420/1387	1424/1403	1565	1578
C-H bend	1610	1455	1463	1534	1569
benzene C-C stretch	1623	1493	1503	1626	1569
C-CH_3 bend	1646	1488	1481	1639	1638
C-C stretch	1688	1557	1570	1777	1764
benzene C-C stretch	1727	1577	1592	1670	1596
benzene C-C stretch	1800	1617	1633	2075	1720
C-C stretch, C-H wag	-	-	-	-	1282
C-C stretch, CH_3 bend	-	-	-	-	1638
Out of plane					
C-CH_3 rotation	142	177	135	111	89
C-C bend	160	177	151	135	123
C-C bend	234	-	219	191	173
C-C bend	306	231	244	286	321/1387
C-C bend	351	347	346	440	321/1387
benzene C-C bend	449	426	424	321	314
benzene C-C bend	591	573	581	412	554
C-C bend	610	601	609	524	586
C2-H□wag	750	780	784	551	624
benzene C-H bend	779	731	737	558	749
C-H bend	780	758	760	629	686

benzene C-H bend	887	-	842	608	963
benzene C-H bend	973	925	928	658	-
benzene C-H bend	1011	983	965	690	-
CH_3 bend	1151	-	1051	1144	1110
CH_3 bend	1630	-	-	1630	1616
C7-H wag	-	-	-	-	391
C4-H wag	-	-	-	-	454

Table S7. Comparison of the root-mean-square error (RMSE), mean relative error (MRE) and Spearman rank correlation between the near-UV CD intensity calculated with different parameters and the experimental intensity.

protein	PDB entry	RMSE / cm^{-1}		MRE		Spearman rank correlation	
		non-vib	*vib*	*non-vib*	*vib*	*non-vib*	*vib*
acetylcholinesterase[16]	2ACE	199	80	1.69	1.19	0.60	0.55
adenylate kinase[17]	2ECK	34	11	0.98	0.36	0.62	0.89
α-lactalbumin[18]	1A4V	195	144	3.00	1.39	0.27	0.20
α -toxin[19]	1QM6	197	92	5.36	6.98	-0.50	0.30
apolipophorin III[20]	1AEP	107	46	9.07	1.46	-0.59	0.71
barnase[21]	1A2P	173	121	1.30	1.07	-0.15	0.83
β-2 microglobulin (human)[22]	1LDS	147	96	4.54	6.74	0.09	0.14
β-lactamase[23]	1BTL	137	76	0.94	0.59	0.28	0.93
P.69 pertactin[24]	1DAB	45	23	9.68	2.50	-0.00	0.55
bovine pancreatic trypsin inhibitor[25]	5PTI	235	128	0.87	0.52	0.93	0.66
calmodulin[26]	4CLN	22	8	0.88	0.44	0.63	0.93
cardiotoxin[27]	4OM4	105	98	1.26	1.33	-0.71	-0.36
chymotrypsinogen A[28]	2CGA	352	169	3.06	9.54	-0.43	-0.03
dehydroquinase II[29]	2BT4	60	41	4.40	7.47	-0.74	0.12
dihydrofolate reductase[30]	4P3Q	62	40	14.28	6.40	0.67	0.63
glucose oxidase[31]	1CF3	68	65	0.79	0.67	0.75	0.65
hen egg white lysozyme[32]	1HF4	246	104	6.54	1.08	-0.04	0.90
human carbonic anhydrase II[33]	2CBA	307	75	2.40	0.63	0.17	0.77
human serum albumin[34]	1AO6	101	90	1.03	1.09	-0.60	0.44
insulin[35]	5ENA	114	56	0.84	0.49	0.97	0.94
interleukin 4 (cytokine)[36]	2B8U	64	46	4.51	1.50	-0.20	-0.43
interleukin 6[37]	1ALU	40	34	1.46	0.97	-0.66	-0.38
monellin[38]	1IV9	83	61	0.96	0.87	0.70	0.41
myoglobin (whale)[39]	1UFP	52	48	38.55	5.15	0.75	-0.80
neocarzinostatin[40]	1NOA	126	102	0.85	0.71	0.79	0.81
odorant binding protein[41]	1A3Y	74	40	0.68	0.49	0.70	0.91
papain[42]	3LFY	254	77	2.45	2.40	-0.06	0.89
pectate lyase C[43]	2PEC	111	26	5.74	1.20	-0.47	0.79
phosphatidylethanolamine-binding protein[44]	1A44	106	70	1.01	0.74	0.01	0.78
phospholipase A2 (Ca^{2+})[45]	1PSJ	279	102	52.18	4.44	0.78	0.87
relaxin[46]	6RLX	244	166	1.45	1.07	0.05	0.82
rhodanese[47]	1DP2	243	71	8.68	8.07	0.61	-0.10
ribonuclease T1[48]	1RN1	93	74	3.44	1.30	0.57	0.73
ribonuclease A[49]	1AFU	123	49	0.88	0.50	0.75	0.95
staphylococcal nuclease[50]	1STN	59	78	1.08	1.64	-0.14	-0.39
extracellular domain of human tissue factor[51]	2HFT	118	77	1.10	0.77	0.04	0.66
sticholysin II[52]	1O72	70	29	5.13	1.01	-0.18	0.83
subtilisin BPN’[53]	1ST2	78	28	0.77	0.55	0.71	0.89
thioredoxin[54]	2TRX	104	90	0.83	1.00	0.40	0.63
tryptophan synthase α-subunit[55]	1WQ5	31	27	5.89	10.68	-0.44	-0.37

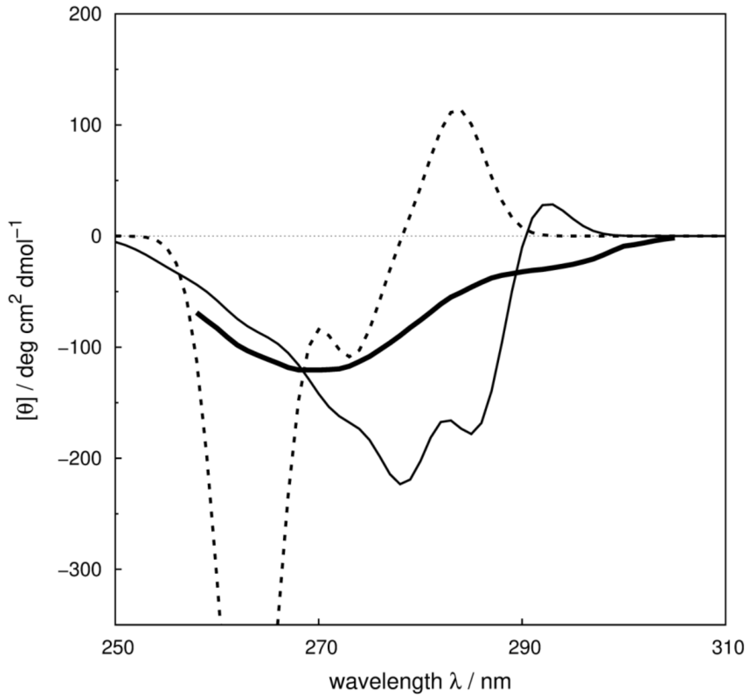

Figure S4. Experimental[16] near-UV CD spectrum (bold solid line) and calculated spectra of acetylcholinesterase (PDB code: 2ACE) with '*non-vib*' (dotted line) or '*vib*' (thin solid line) parameter sets. There is noticeable qualitative and quantitative (RMSE drops from 199 to 80 cm^{-1}) improvement with the new parameters.

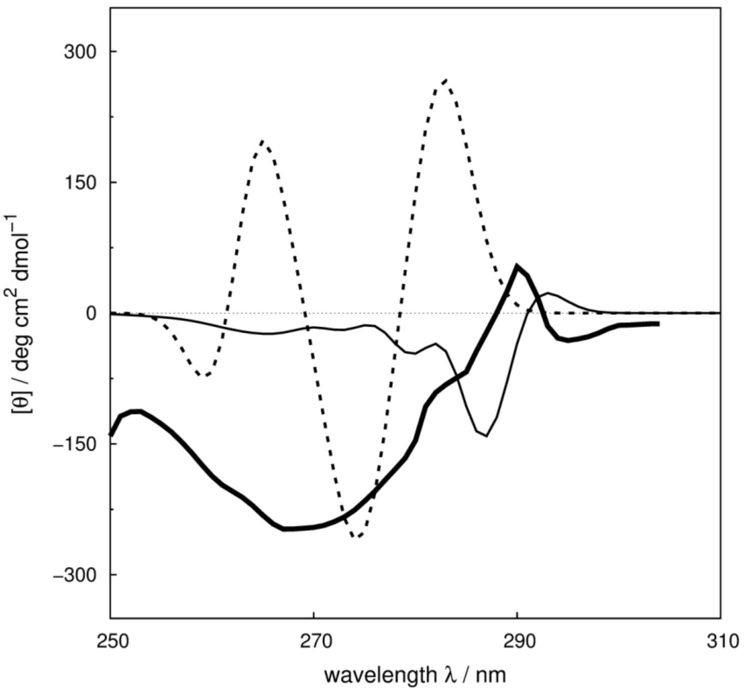

Figure S5. Experimental[18] near-UV CD spectrum (bold solid line) and calculated spectra of α-lactalbumin (PDB code: 1A4V) with '*non-vib*' (dotted line) or '*vib*' (thin solid line) parameter sets. There is modest improvement (RMSE drops from 195 to 144 cm^{-1}), but quantitative agreement is still lacking.

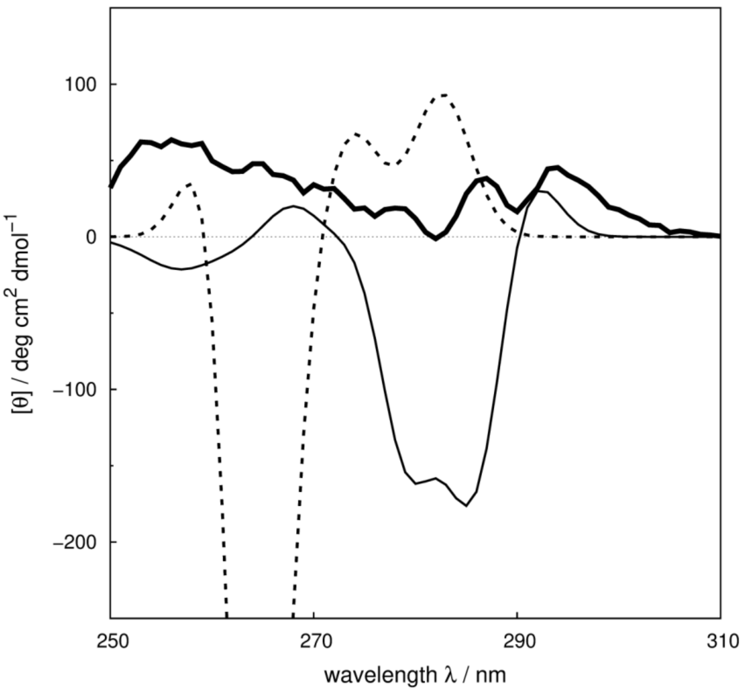

Figure S6. Experimental[19] near-UV CD spectrum (bold solid line) and calculated spectra of α-toxin (PDB code: 1QM6) with '*non-vib*' (dotted line) or '*vib*' (thin solid line) parameter sets. There is modest improvement (RMSE drops from 197 to 92 cm^{-1}), but quantitative agreement is still lacking and the calculations wrongly predict an intense negative peak between 275 and 285 nm.

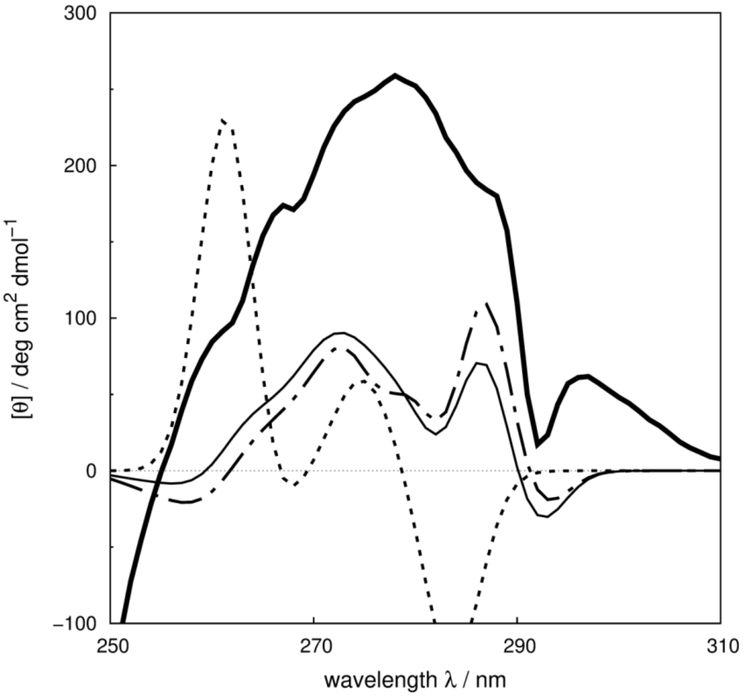

Figure S7. Experimental[21] near-UV CD spectrum (bold solid line) and calculated spectra of barnase (PDB code: 1A2P) with '*non-vib*' (dotted line) or '*vib*' (thin solid line) parameter sets with the X-ray structure. Calculated spectra from the NMR structures (PDB code: 1FW7) use the '*vib*' parameters (dotted-dash line). The new parameters give a qualitatively better computed spectrum, with either the X-ray or NMR structures. The band structure agrees well with experiment, but the intensity is under-estimated.

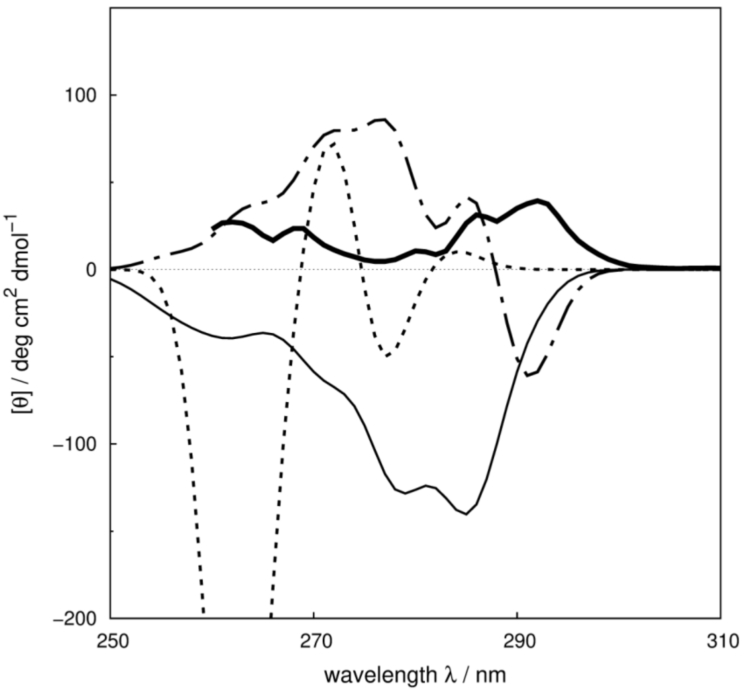

Figure S8. Experimental[22] near-UV CD spectrum (bold solid line) and calculated spectra of β-2 microglobulin (PDB code: 1LDS) with *'non-vib'* (dotted line) or *'vib'* (thin solid line) parameter sets. Calculated spectra from the NMR structures (PDB code: 1JNJ) use the *'vib'* parameters (dotted-dash line). The calculated spectrum using the new parameters and the NMR structures gives a noticeable improvement with experiment, but full quantitative agreement is still lacking.

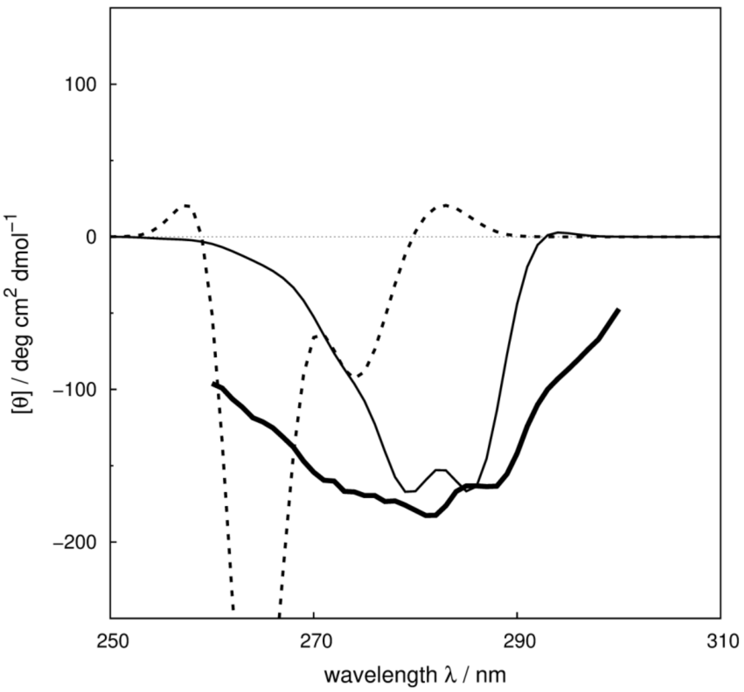

Figure S9. Experimental[23] near-UV CD spectrum (bold solid line) and calculated spectra of β-lactamase (PDB code: 1BTL) with '*non-vib*' (dotted line) or '*vib*' (thin solid line) parameter sets. There is noticeable qualitative (MRE drops from 0.94 to 0.59) and quantitative (RMSE drops from 137 to 76 cm^{-1}) improvement with the new parameters.

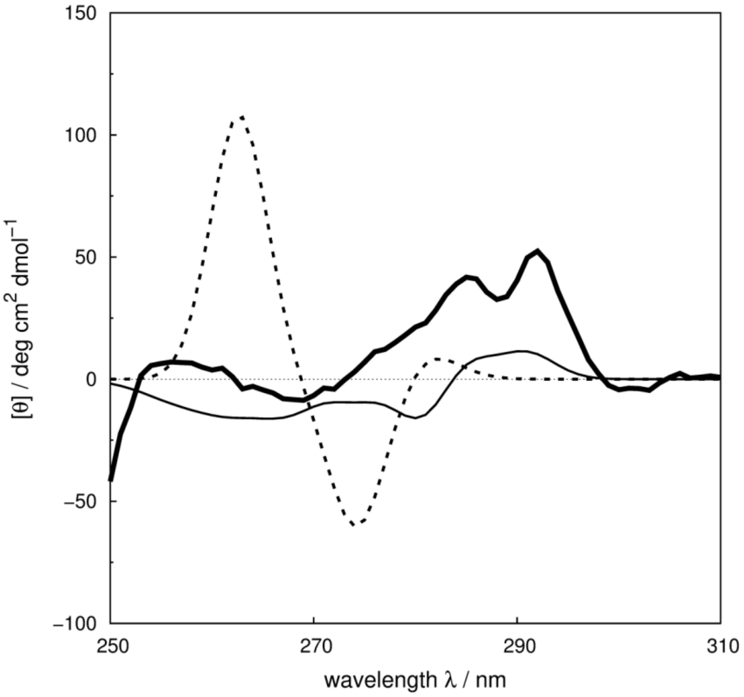

Figure S10. Experimental[24] near-UV CD spectrum (bold solid line) and calculated spectra of P. 69 pertactin (PDB code: 1DAB) with ‘*non-vib*’ (dotted line) or ‘*vib*’ (thin solid line) parameter sets. The new parameters improve the calculation of what is quite a weak near-UV CD spectrum.

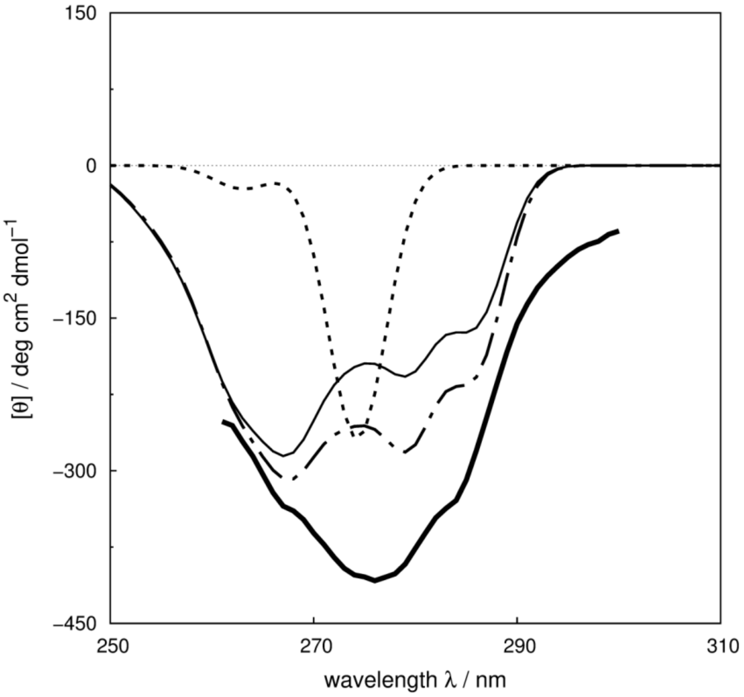

Figure S11. Experimental[25] near-UV CD spectrum (bold solid line) and calculated spectra of bovine pancreatic trypsin inhibitor (PDB code: 5PTI) with *'non-vib'* (dotted line) or *'vib'* (thin solid line) parameter sets. Calculated spectra from the NMR structures (PDB code: 1PIT) use the *'vib'* parameters (dotted-dash line). The new parameters improve the calculated spectrum (MRE drops from 0.87 to 0.52, and the RMSE drops from 235 to 128 cm^{-1}). The use of the NMR structures gives some further noticeable improvement. The band structure is reproduced well, although the intensity is under-estimated.

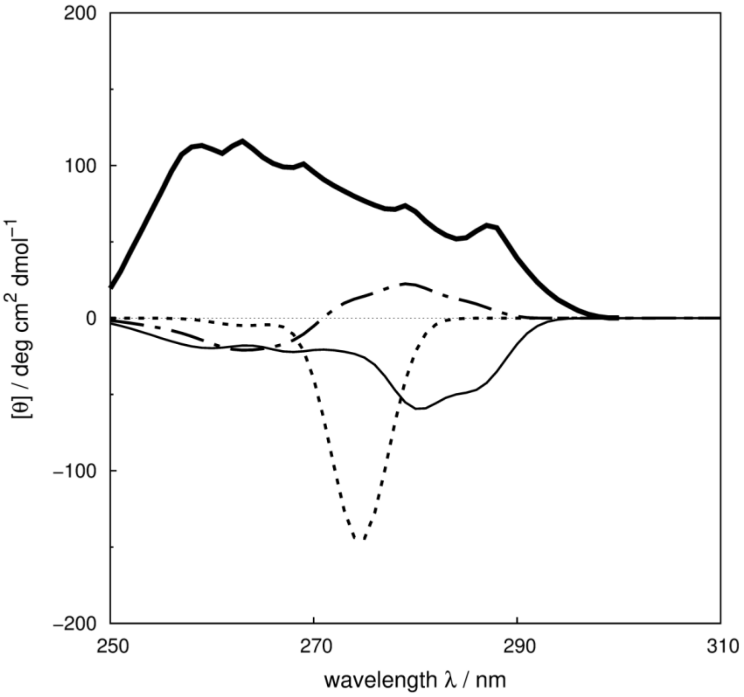

Figure S12. Experimental[27] near-UV CD spectrum (bold solid line) and calculated spectra of cardiotoxin (PDB code: 4OM4) with *'non-vib'* (dotted line) or '*vib*' (thin solid line) parameter sets. Calculated spectra from the NMR structures (PDB code: 1CRF) use the *'vib'* parameters (dotted-dash line). Calculation with the NMR structures predicts a positive band between 270 and 290 nm correctly which is predicted to be negative using the X-ray structure.

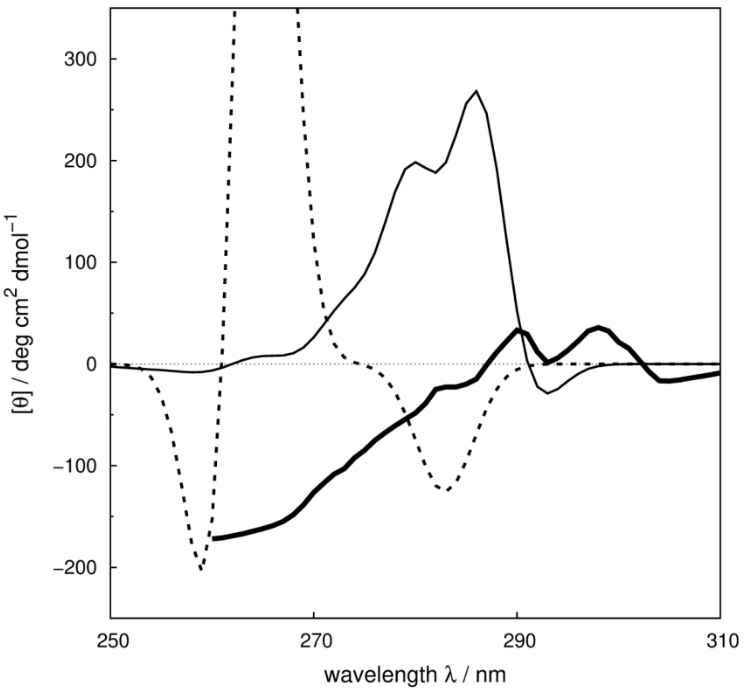

Figure S13. Experimental[28] near-UV CD spectrum (bold solid line) and calculated spectra of chymotrypsinogen A (PDB code: 2CGA) with '*non-vib*' (dotted line) or '*vib*' (thin solid line) parameter sets. The calculated spectrum using the new parameters shows noticeable quantitative (RMSE drops from 352 to 169 cm^{-1}) improvement. The band structure agrees with the experiment, but the calculated spectrum is blue-shifted and the intensity is over-estimated.

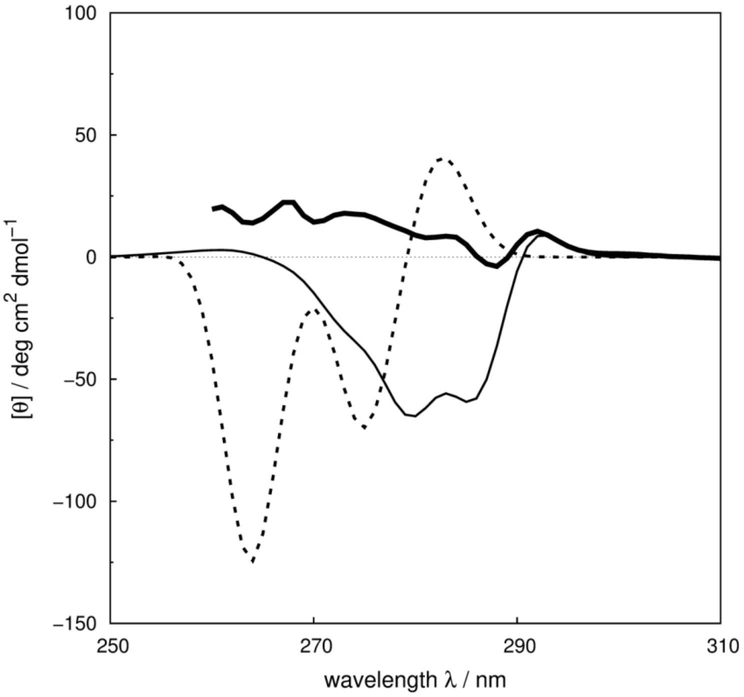

Figure S14. Experimental[29] near-UV CD spectrum (bold solid line) and calculated spectra of dehydroquinase II (PDB code: 2BT4) with ‘*non-vib*’ (dotted line) or ‘*vib*’ (thin solid line) parameter sets. The calculation predicts negative bands between 265 and 290 nm while the experiment shows weak positive bands. However, the prediction of the band structure shows noticeable improvement (Spearman rank correlation increases from -0.74 to 0.12) with the new parameters.

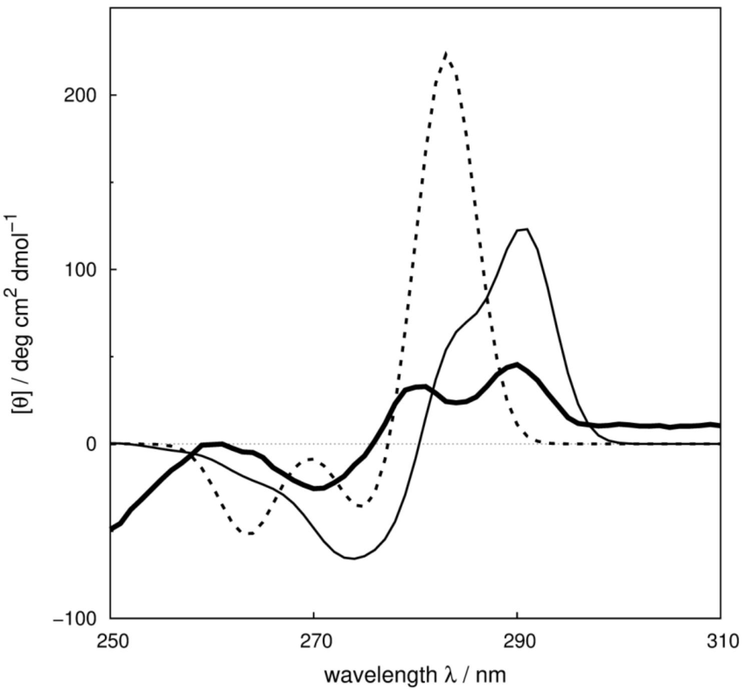

Figure S15. Experimental[30] near-UV CD spectrum (bold solid line) and calculated spectra of dihydrofolate reductase (PDB code: 4P3Q) with '*non-vib*' (dotted line) or '*vib*' (thin solid line) parameter sets. The calculated spectrum with the new parameters shows comparable Spearman rank correlation to the '*non-vib*'. The band structure is better reproduced with more details using the '*vib*' parameter sets.

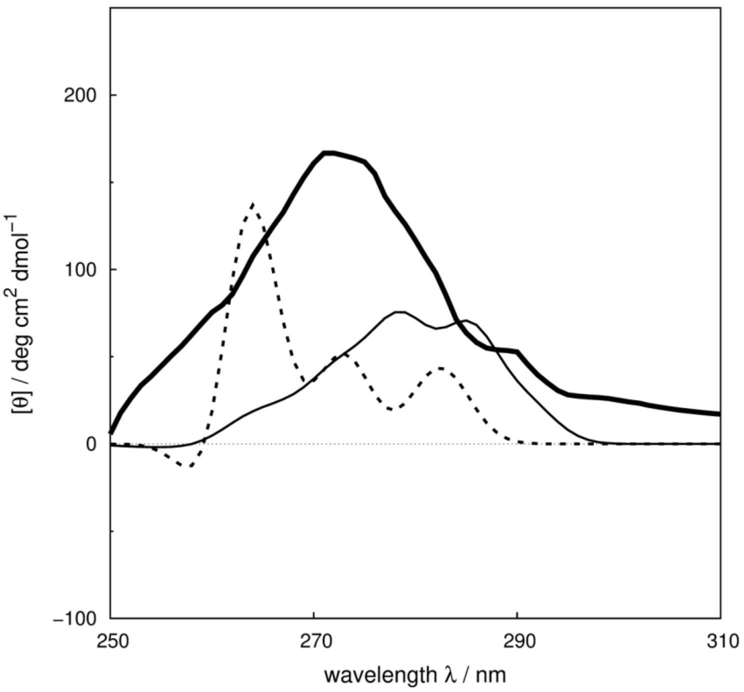

Figure S16. Experimental[31] near-UV CD spectrum (bold solid line) and calculated spectra of glucose oxidase (PDB code: 1CF3) with ‘*non-vib*’ (dotted line) or ‘*vib*’ (thin solid line) parameter sets. The calculated spectra with ‘*non-vib*’ and ‘*vib*’ parameters show comparable RMSE, MRE and Spearman rank correlation. Calculation with the new parameters gives better prediction above 270 nm while the ‘*non-vib*’ parameters show better results below 270 nm.

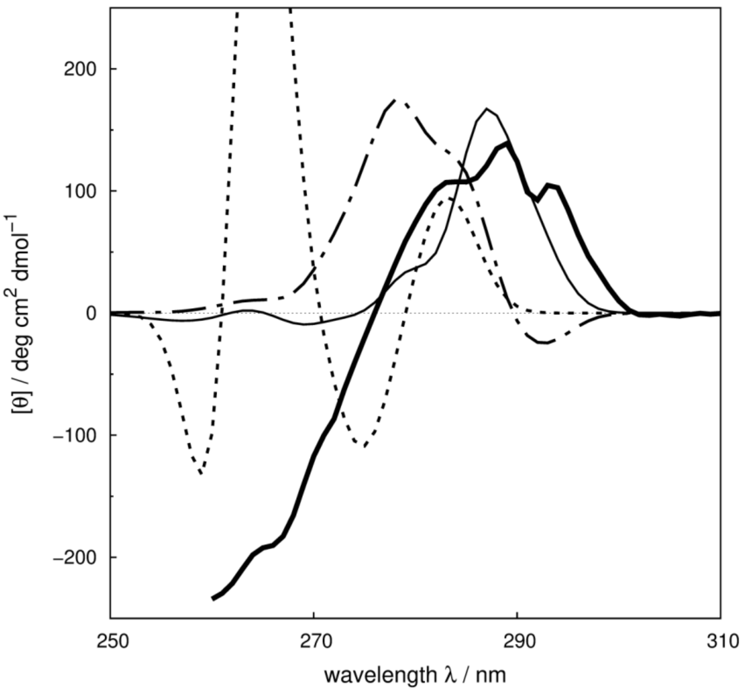

Figure S17. Experimental[32] near-UV CD spectrum (bold solid line) and calculated spectra of hen egg white lysozyme (PDB code: 1HF4) with *'non-vib'* (dotted line) or '*vib*' (thin solid line) parameter sets. Calculated spectra from the NMR structures (PDB code: 1E8L) use the '*vib*' parameters (dotted-dash line). The new parameters give a qualitatively better computed spectrum, with either the X-ray or NMR structures. The band structure agrees well with experiment with a comparable intensity.

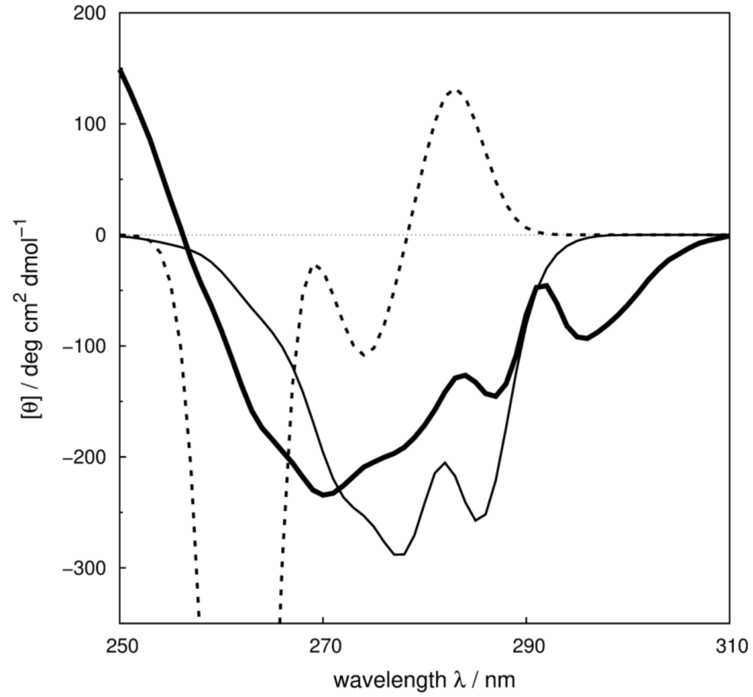

Figure S18. Experimental[33] near-UV CD spectrum (bold solid line) and calculated spectra of human carbonic anhydrase II (PDB code: 2CBA) with ‘*non-vib*’ (dotted line) or ‘*vib*’ (thin solid line) parameter sets. There is noticeable qualitative and quantitative (RMSE drops from 307 to 75 cm^{-1}) improvement with the new parameters.

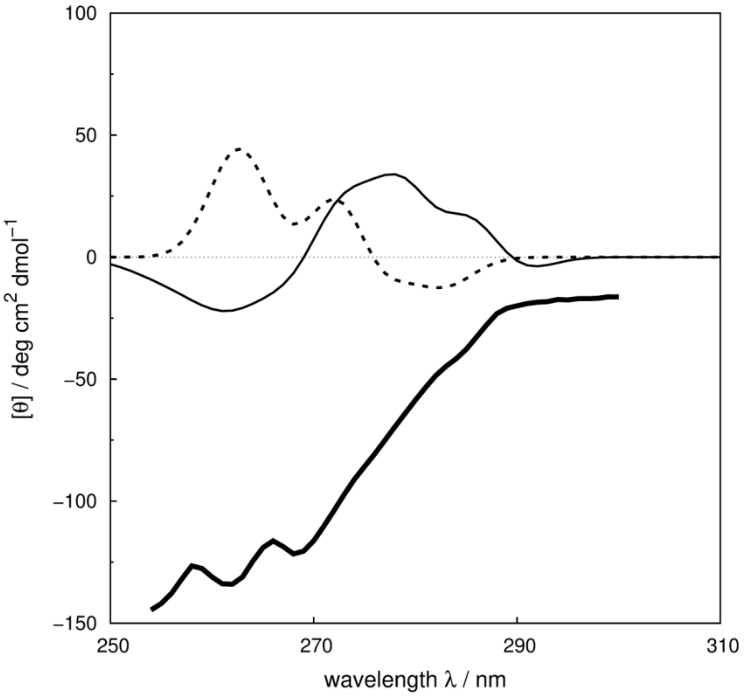

Figure S19. Experimental[34] near-UV CD spectrum (bold solid line) and calculated spectra of human serum albumin (PDB code: 1AO6) with '*non-vib*' (dotted line) or '*vib*' (thin solid line) parameter sets. Quantitative agreement for this protein is still lacking. The calculated spectrum with new parameters shows better Spearman rank correlation due to the prediction of the negative band below 270 nm.

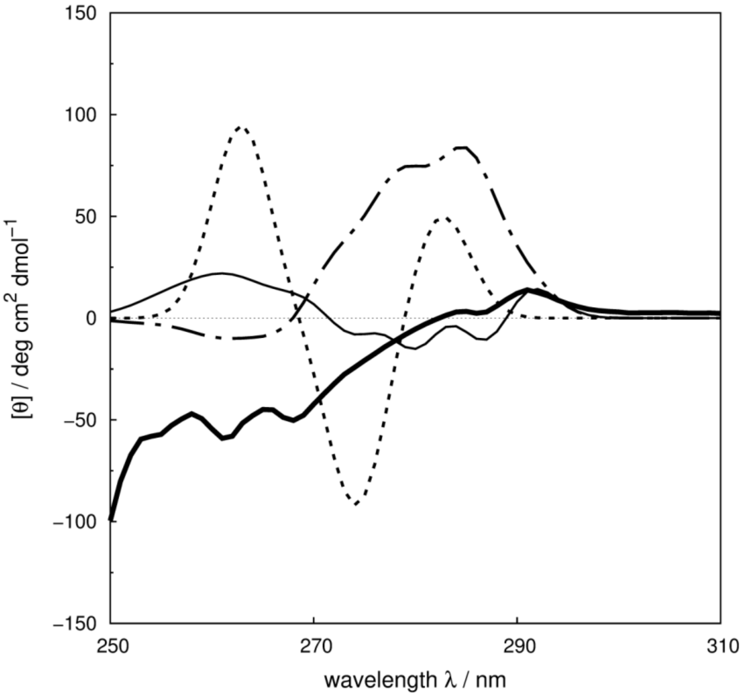

Figure S20. Experimental[36] near-UV CD spectrum (bold solid line) and calculated spectra of interleukin 4 (PDB code: 2B8U) with *'non-vib'* (dotted line) or *'vib'* (thin solid line) parameter sets. Calculated spectra from the NMR structures (PDB code: 1CYL) use the *'vib'* parameters (dotted-dash line). There is modest improvement (RMSE drops from 64 to 46 cm^{-1}), but quantitative agreement is still lacking and the calculations wrongly predict an positive peak between 250 and 275 nm. The calculated spectrum with the NMR structures shows better prediction of the sign of the bands with over-estimated intensities.

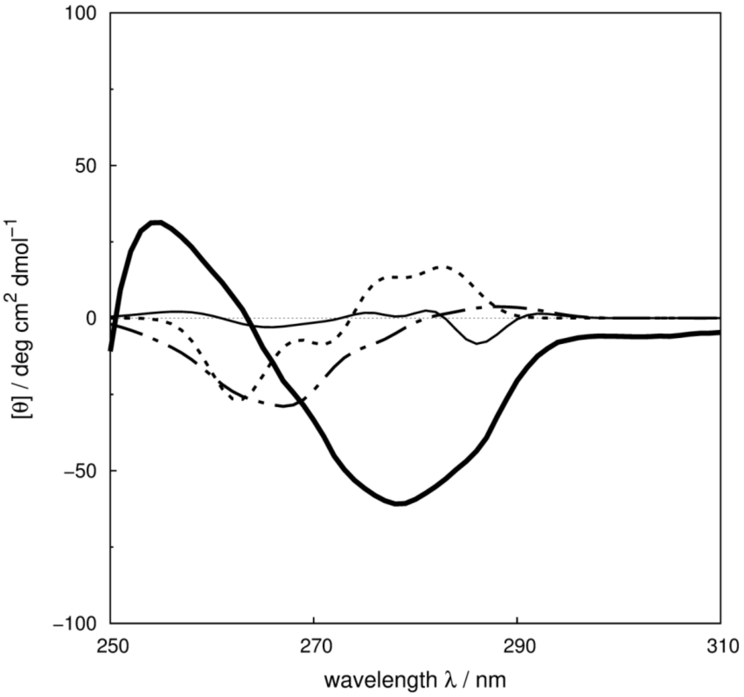

Figure S21. Experimental[37] near-UV CD spectrum (bold solid line) and calculated spectra of interleukin 6 (PDB code: 1ALU) with *'non-vib'* (dotted line) or *'vib'* (thin solid line) parameter sets. Calculated spectra from the NMR structures (PDB code 2IL6) use the *'vib'* parameters (dotted-dash line). Quantitative agreement is still lacking for this protein calculated with either X-ray or NMR structures.

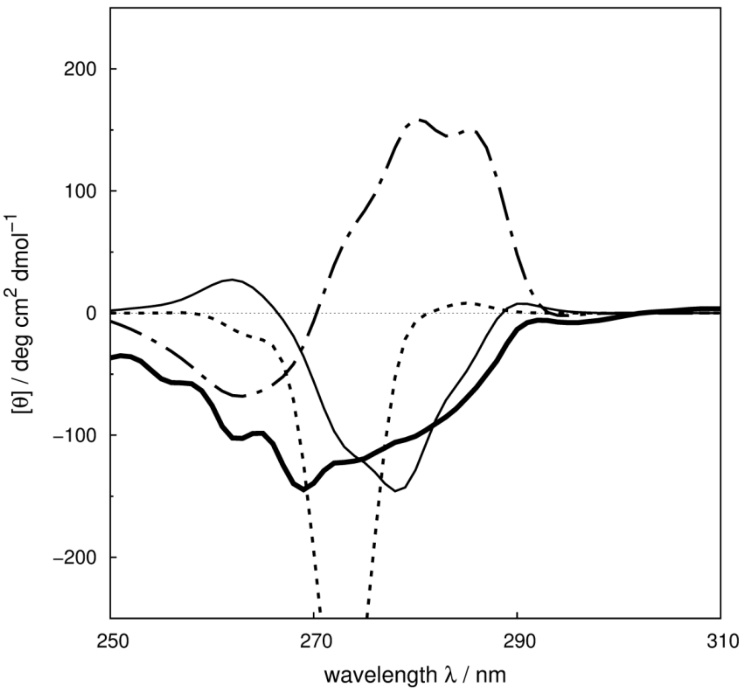

Figure S22. Experimental[38] near-UV CD spectrum (bold solid line) and calculated spectra of monellin (PDB code: 1IV9) with *'non-vib'* (dotted line) or *'vib'* (thin solid line) parameter sets. Calculated spectra from the NMR structures (PDB code: 1MNL) use the *'vib'* parameters (dotted-dash line). Calculation with the new parameters shows modest improvement between 265 and 290 nm, but quantitative agreement is still lacking. Calculations with the NMR structures show a well reproduced band below 270 nm but a wrongly predicted band over 270 nm.

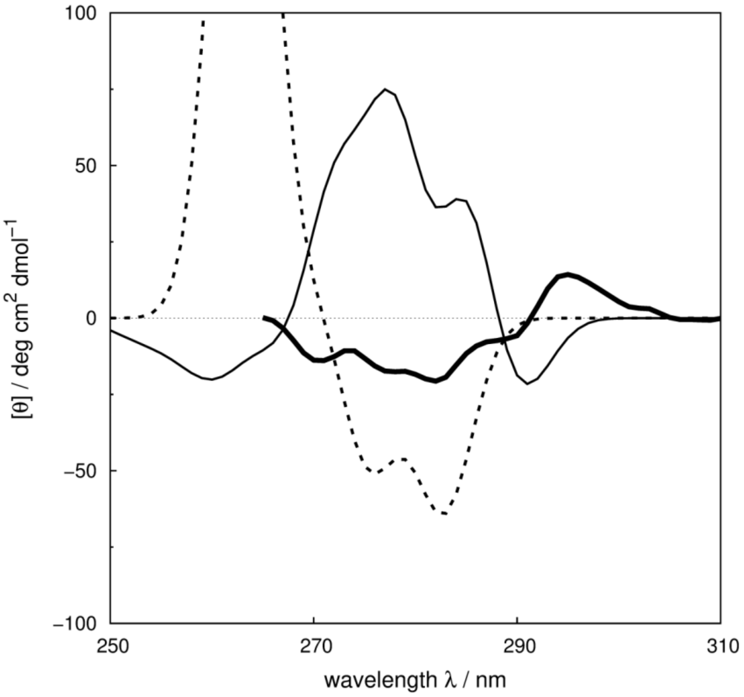

Figure S23. Experimental[39] near-UV CD spectrum (bold solid line) and calculated spectra of whale myoglobin (PDB code: 1UFP) with '*non-vib*' (dotted line) or '*vib*' (thin solid line) parameter sets. Quantitative agreement is still lacking for this weak near-UV CD spectrum.

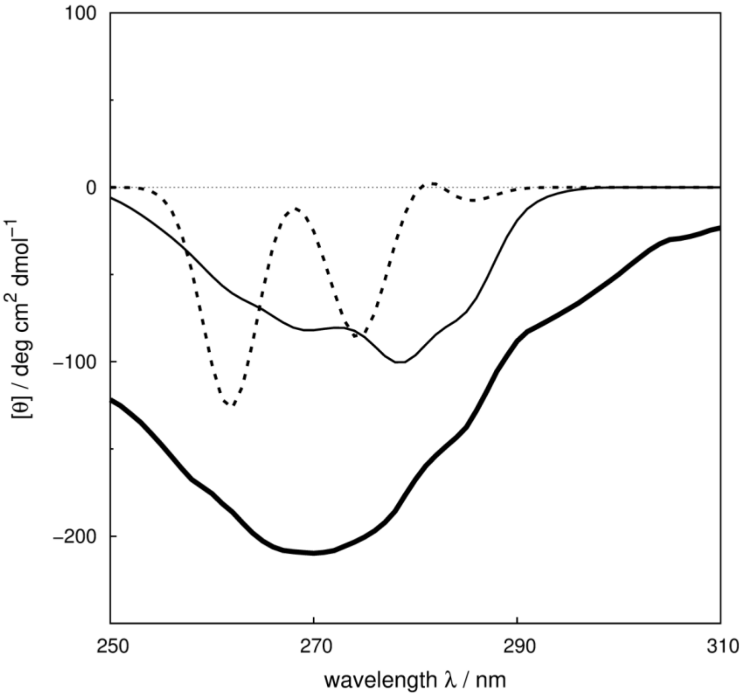

Figure S24. Experimental[40] near-UV CD spectrum (bold solid line) and calculated spectra of neocarzinostatin (PDB code: 1NOA) with ‘*non-vib*’ (dotted line) or ‘*vib*’ (thin solid line) parameter sets. The new parameters improve the calculated spectrum with a better band structure.

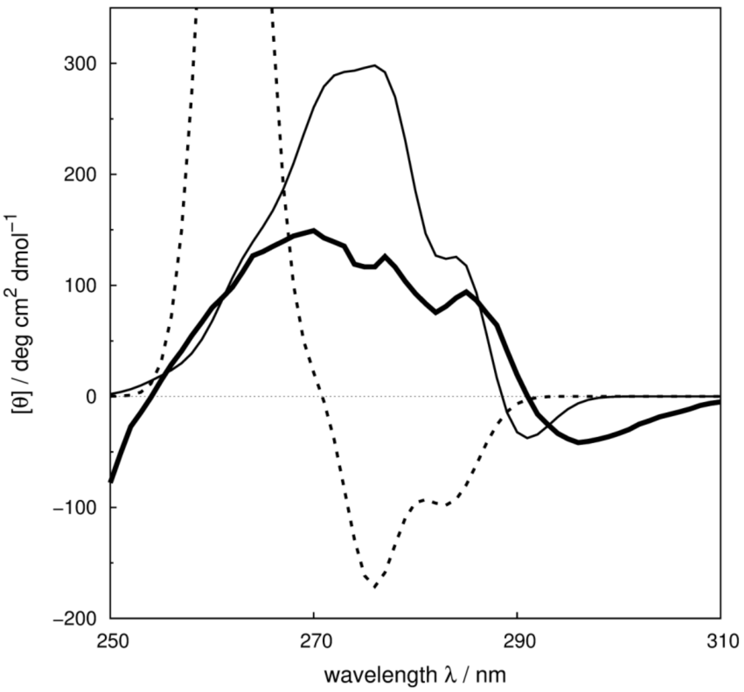

Figure S25. Experimental[42] near-UV CD spectrum (bold solid line) and calculated spectra of papain (PDB code: 3LFY) with '*non-vib*' (dotted line) or '*vib*' (thin solid line) parameter sets. There is noticeable qualitative and quantitative (RMSE drops from 254 to 77 cm^{-1}) improvement with the new parameters. The band structure is well reproduced with a Spearman rank correlation of 0.89.

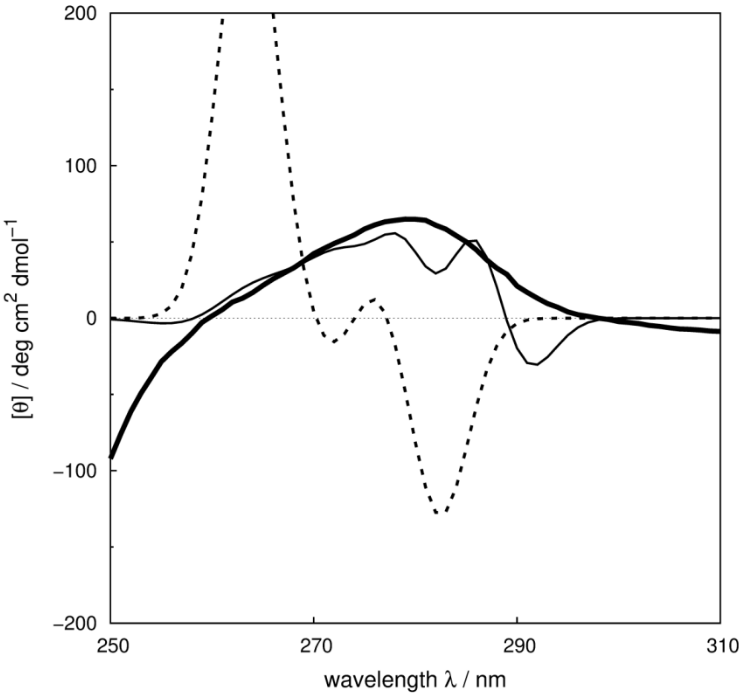

Figure S26. Experimental[43] near-UV CD spectrum (bold solid line) and calculated spectra of peptate lyase C (PDB code: 2PEC) with '*non-vib*' (dotted line) or '*vib*' (thin solid line) parameter sets. There is noticeable qualitative (MRE drops from 5.74 to 1.20) and quantitative (RMSE drops from 111 to 26 cm^{-1}) improvement with the new parameters.

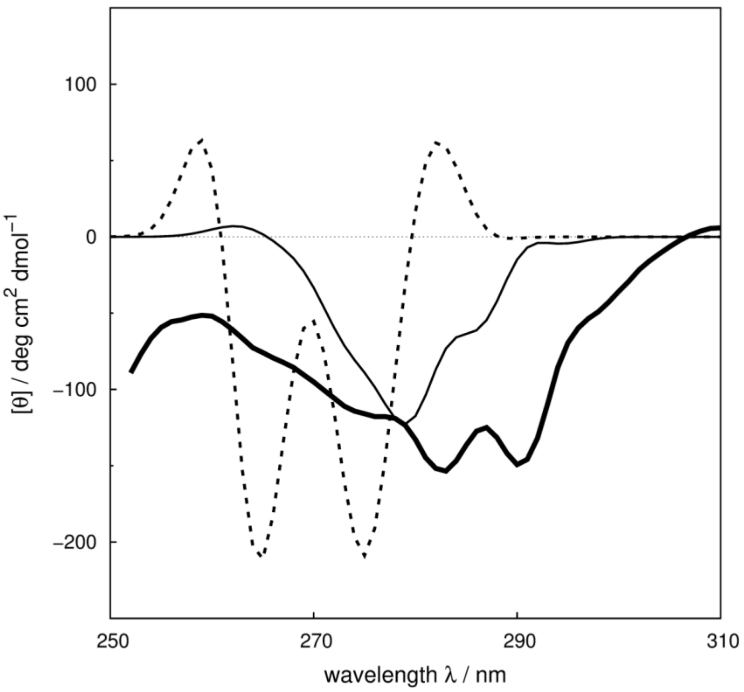

Figure S27. Experimental[44] near-UV CD spectrum (bold solid line) and calculated spectra of phosphatidylethanolamine-binding protein (PDB code: 1A44) with '*non-vib*' (dotted line) or '*vib*' (thin solid line) parameter sets. There is modest quantitative improvement (RMSE drops from 106 to 70 cm^{-1}) with a better reproduced band structure (Spearman rank correlation increases from 0.01 to 0.78).

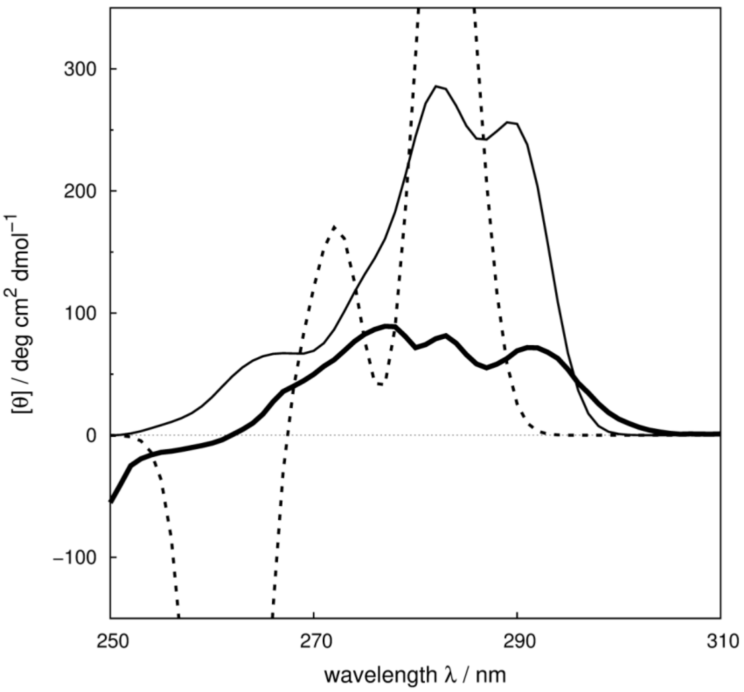

Figure S28. Experimental[45] near-UV CD spectrum (bold solid line) and calculated spectra of phospholipase A2 (Ca^{2+}) (PDB code: 1PSJ) with '*non-vib*' (dotted line) or '*vib*' (thin solid line) parameter sets. The new parameters give a qualitatively and quantitative better computed spectrum. The band structure agrees well with experiment, but the intensity is over-estimated.

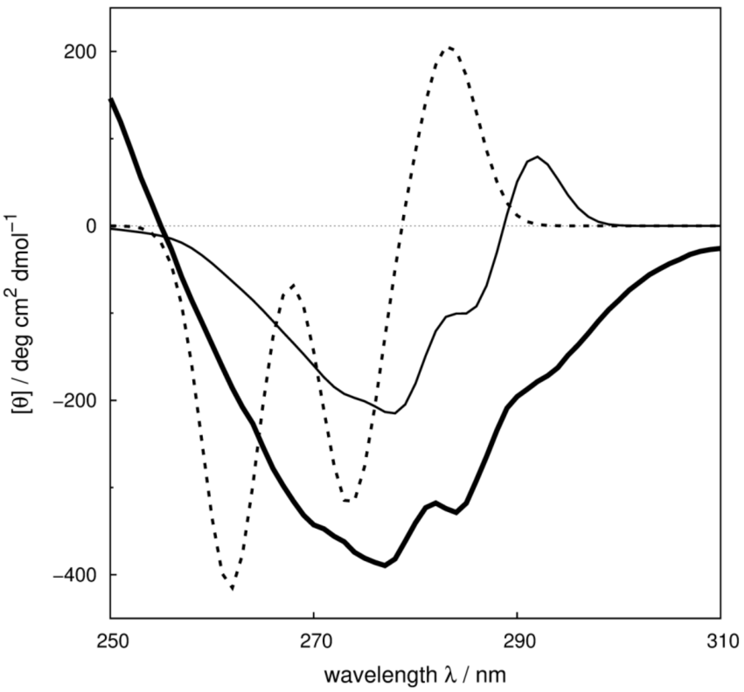

Figure S29. Experimental[46] near-UV CD spectrum (bold solid line) and calculated spectra of relaxin (PDB code: 6RLX) with '*non-vib*' (dotted line) or '*vib*' (thin solid line) parameter sets. There is noticeable quantitative (RMSE drops from 244 to 166 cm^{-1}) improvement with the new parameters. The band structure is well reproduced (Spearman rank correlation increases from 0.05 to 0.82) with the new parameters.

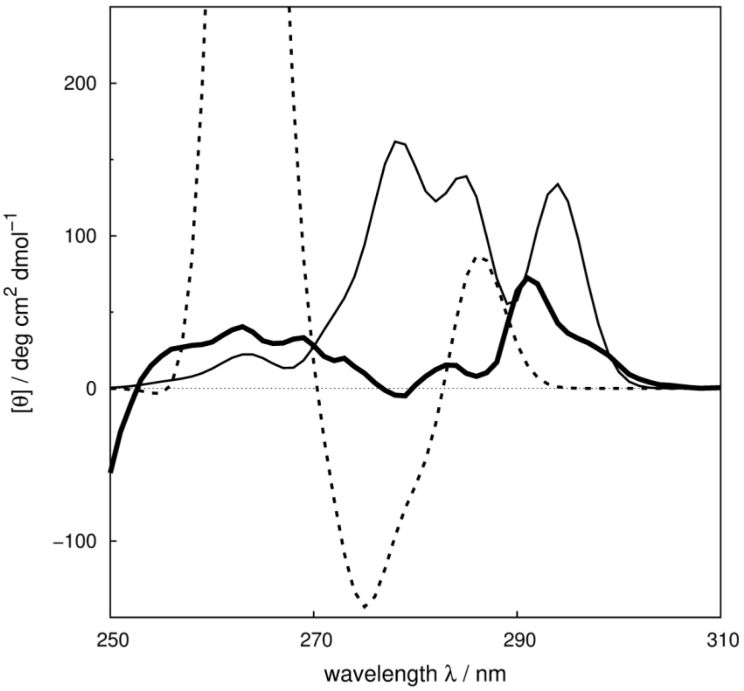

Figure S30. Experimental[47] near-UV CD spectrum (bold solid line) and calculated spectra of rhodanese (PDB code: 1DP2) with '*non-vib*' (dotted line) or '*vib*' (thin solid line) parameter sets. There is modest improvement (RMSE drops from 243 to 71 cm^{-1}), but quantitative agreement is still lacking.

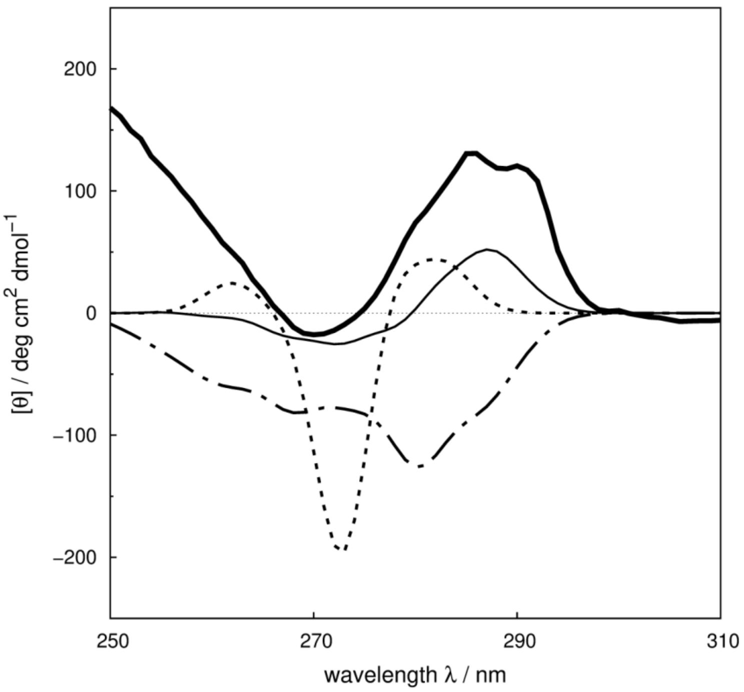

Figure S31. Experimental[48] near-UV CD spectrum (bold solid line) and calculated spectra of ribonuclease T1 (PDB code: 1RN1) with '*non-vib*' (dotted line) or '*vib*' (thin solid line) parameter sets. Calculated spectra from the NMR structures (PDB code: 1YGW) use the '*vib*' parameters (dotted-dash line). The calculated spectrum with the new parameters shows noticeable improvement between 270 and 300 nm using X-ray structure while the calculation with the NMR structures wrongly predicts a negative band.

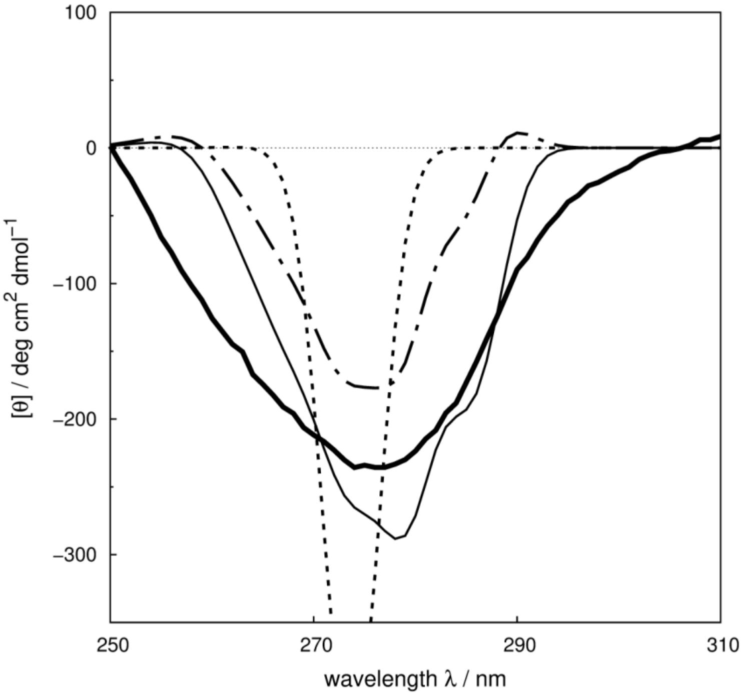

Figure S32. Experimental[49] near-UV CD spectrum (bold solid line) and calculated spectra of ribonuclease A (PDB code: 1AFU) with '*non-vib*' (dotted line) or '*vib*' (thin solid line) parameter sets. Calculated spectra from the NMR structures (PDB code: 2AAS) use the '*vib*' parameters (dotted-dash line). There is modest quantitative (RMSE drops from 123 to 49 cm^{-1}) improvement with the new parameters. Calculations with the NMR structures predict a less intense spectrum.

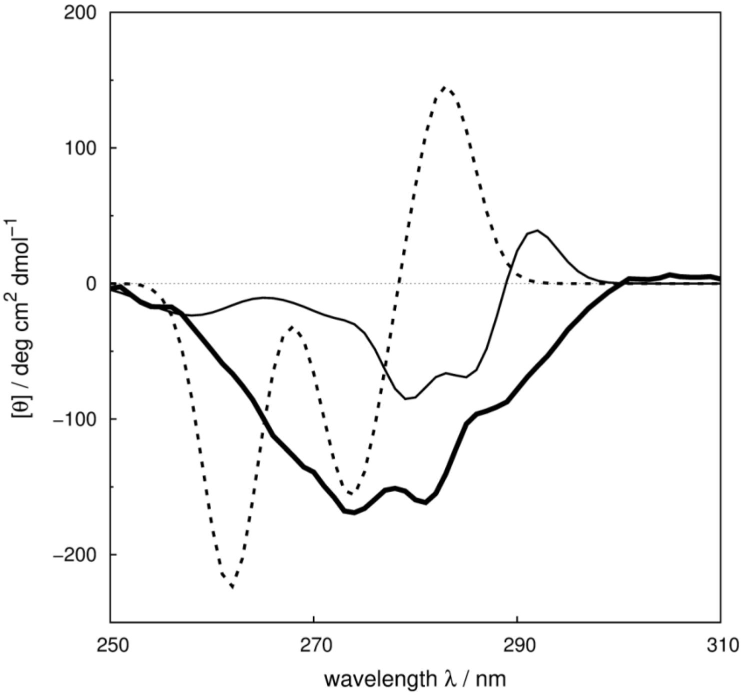

Figure S33. Experimental[51] near-UV CD spectrum (bold solid line) and calculated spectra of extracellular domain of human tissue factor (PDB code: 2HFT) with ‘*non-vib*’ (dotted line) or ‘*vib*’ (thin solid line) parameter sets. There is noticeable quantitative (RMSE drops from 118 to 77 cm^{-1}) improvement with the new parameters. The band structure is well reproduced (Spearman rank correlation increases from 0.04 to 0.66) with the new parameters.

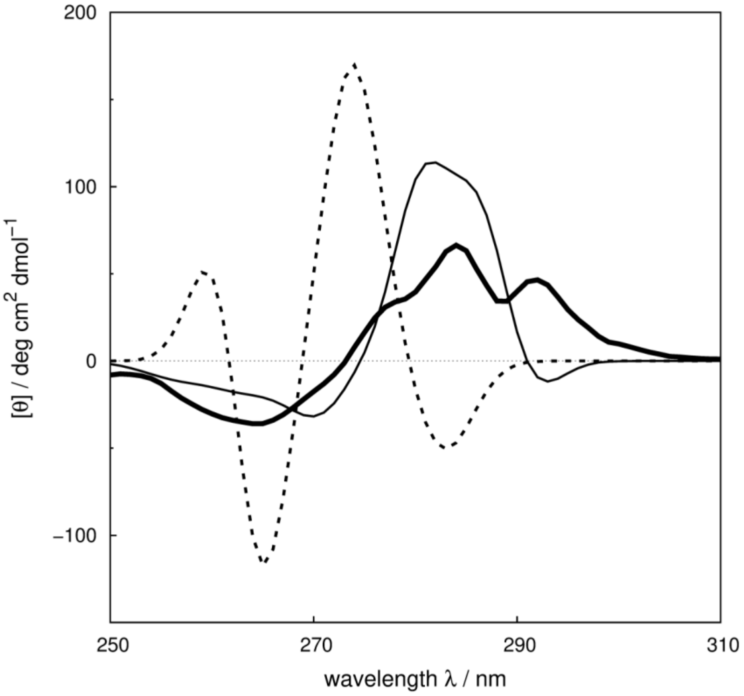

Figure S34. Experimental[52] near-UV CD spectrum (bold solid line) and calculated spectra of sticholysin II (PDB code: 1O72) with '*non-vib*' (dotted line) or '*vib*' (thin solid line) parameter sets. There is noticeable qualitative (MRE drops from 5.13 to 1.01) and quantitative (RMSE drops from 70 to 29 cm^{-1}) improvement with the new parameters. The band structure is also better reproduced (Spearman rank correlation increases from -0.18 to 0.83).

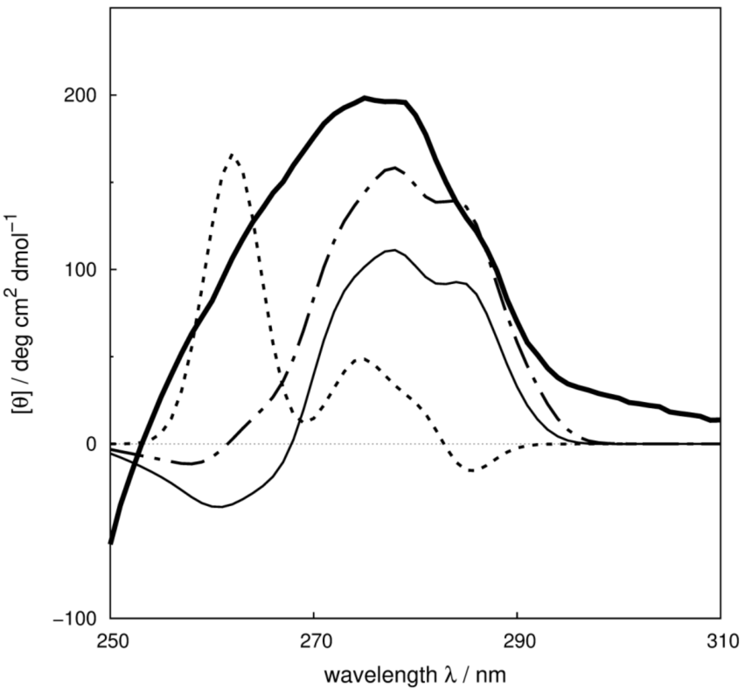

Figure S35. Experimental[54] near-UV CD spectrum (bold solid line) and calculated spectra of thioredoxin (PDB code: 2TRX) with ‘*non-vib*’ (dotted line) or *‘vib’* (thin solid line) parameter sets. Calculated spectra from the NMR structures (PDB code: 1XOA) use the *‘vib’* parameters (dotted-dash line). The new parameters improve the calculated spectrum (Spearman rank correlation increases from 0.40 to 0.63, and the RMSE drops from 104 to 90 cm^{-1}). The use of the NMR structures gives further noticeable improvement in intensity.

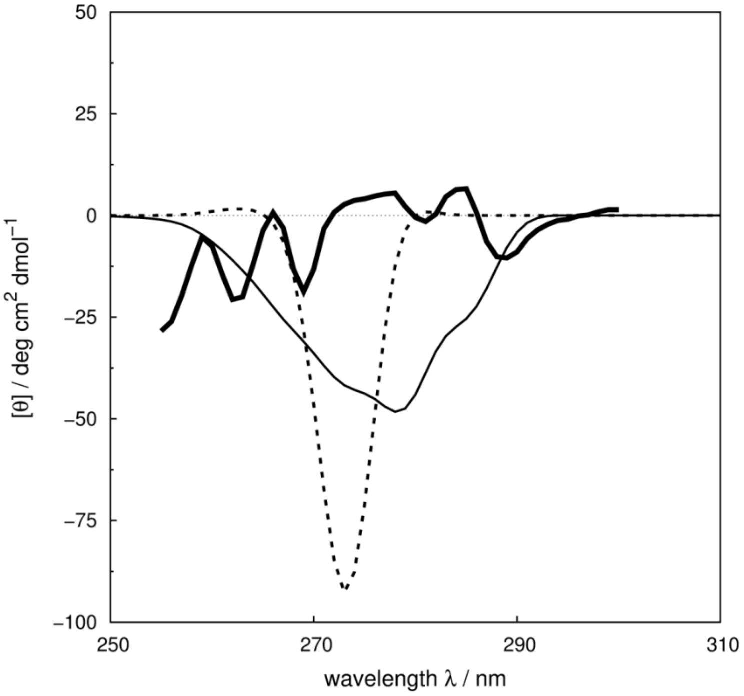

Figure S36. Experimental[55] near-UV CD spectrum (bold solid line) and calculated spectra of tryptophan synthase α-subunit (PDB code: 1WQ5) with '*non-vib*' (dotted line) or '*vib*' (thin solid line) parameter sets. Quantitative agreement is still lacking for this protein. Both parameters predict negative bands while the experiment shows both negative and positive bands.

The X-ray structures we used in our calculation include 16 proteins that contain multiple chains with identical sequences. All chains are considered in the calculation; this is necessary if the chains (despite identical sequence) do not share similar conformations or there are interactions between aromatic residues from different chains. Chains in the same protein with the same sequence may have distinguishable conformational differences arising from a specific residue or motif which may lead to various optical features in their near-UV CD spectra of individual chains. Calculation with one chain instead of the whole protein may not be sufficient to reproduce the experimental intensities which may be affected by cancellation or enhancement from multiple chains at certain wavelength. Proteins containing multiple chains can be grouped into four cases (Table S8). The first kind (Group 1, including adenylate kinase, human serum albumin, papain, ribonuclease A, thioredoxin and ribonuclease T1) has low all-atom RMSD between chains. There are no interactions between aromatic residues on different chains for these proteins and the Spearman rank correlations of calculations using all chains or considering only single chain are comparable. Two proteins (Group 2), α-toxin and dehydroquinase II, have similar chain conformations with aromatic rings adjacent to each other from different chains. There are no aromatic interactions between chains for proteins in Group 3. However, ^{146}Tyr in chymotrypsinogen A, ^{62}Trp in hen egg white lysozyme and ^{17}Tyr in barnase are not superimposable among chains. Another five proteins (Group 4) have both aromatic interactions between chains and residues or motifs that cannot superimpose among chains, such as ^{52}Phe in monellin, ^{10}Phe in odorant binding protein, ^{10}Trp and ^{62}Phe in sticholysin II, ^{72}Phe and ^{102}Tyr in tryptophan synthase α-subunit and ^{10}Phe in cardiotoxin. Comparison of the Spearman rank correlations (Table S8) between calculations with each single chain or the whole protein and their experimental spectra confirm the necessity of calculation with multiple chains of proteins.

Table S8. Spearman rank correlation coefficients between calculated intensity using vibrational parameters and the experiment spectra for all chain or each individual chain of multimeric proteins.

Protein		No. of chain	Spearman rank correlation				all-atom RMSD[a]			aromatic interaction between chains[b]	Group
			All	Chain A	Chain B	Chain C					
adenylate kinase	2ECK	2	0.89	0.89	0.89		0.05			0	1
α-toxin	1QM6	2	0.3	0.32	0.32		0.11			^{65}Tyr-^{65}Tyr	2
chymotrypsinogen A	2CGA	2	-0.03	-0.08	0.10		0.70			0	3
hen egg white lysozyme	1HF4	2	0.9	0.76	0.91		0.52			0	3
human serum albumin	1AO6	2	0.44	0.49	0.33		0.28			0	1
monellin	1IV9	2	0.41	0.59	0.44		0.63			^{3}Trp-^{163}Tyr	4
odorant binding protein	1A3Y	2	0.91	0.76	0.90		0.52			^{66}Phe-^{92}Tyr;^{56}Phe-^{10}Phe	4
papain	3LFY	2	0.89	0.91	0.90		0.26			0	1
ribonuclease A	1AFU	2	0.95	0.96	0.94		0.46			0	1
sticholysin II	1O72	2	0.83	0.81	0.63		0.46			^{91}Tyr-^{62}Phe	4
thioredoxin	2TRX	2	0.63	0.67	0.62		0.66			0	1
tryptophan synthase α-subunit	1WQ5	2	-0.37	-0.74	-0.57		0.77			^{72}Phe-^{102}Tyr;^{72}Phe-^{22}Phe	4
barnase	1A2P	3	0.83	0.15	0.86	0.80	0.23	0.31		0	3
ribonuclease T1	1RN1	3	0.73	0.75	0.65	0.70	0.41	0.41		0	1
cardiotoxin	4OM4	5	-0.36	Chain A	Chain B	Chain C				^{10}Phe(C)-^{10}Phe(E)	4
				-0.47	-0.72	-0.29	0.63	0.39			
					Chain D	Chain E					
					0.34	-0.49	0.76	0.70			
dehydroquinase II	2BT4	12	0.12	Chain A	Chain B	Chain C				^{116}Phe(A)-^{321}Tyr(B); ^{121}Phe(A)-^{516}Phe(C);	2
				0.01	-0.07	-0.03		0.15	0.12	^{140}Phe(A)-^{740}Phe(D); ^{316}Phe(B)-^{521}Tyr(C);	
				Chain D	Chain E	Chain F				^{340}Phe(B)-^{1340}Phe(G); ^{540}Phe(C)-^{1940}Phe(J);	
				-0.20	-0.10	-0.11	0.13	0.18	0.08	^{716}Phe(D)-^{921}Tyr(E); ^{721}Phe(D)-^{1116}Phe(F);	
				Chain G	Chain H	Chain I				^{916}Phe(E)-^{1121}Tyr(F); ^{940}Phe(E)-^{2340}Phe(L);	
				-0.15	-0.05	-0.19	0.09	0.12	0.13	^{1140}Phe(F)-^{1540}Phe(H); ^{1316}Phe(G)-^{1521}Tyr(H);	
				Chain J	Chain K	Chain L				^{1321}Tyr(G)-^{1716}Phe(I); ^{1516}Phe(H)-^{1721}Tyr(I);	

				-0.13	-0.18	0.01	0.10	0.08	0.08	^{1740}Phe(I)-^{2140}Phe(K); ^{1916}Phe(J)-^{2121}Tyr(K); ^{1921}Tyr(J)-^{2316}Phe(L); ^{2116}Phe(K)-^{2321}Tyr(L)	

a. all-atom RMSD compares chains with the first chain in the protein data bank file. b. Aromatic groups within 8 Å are considered to be interacted.

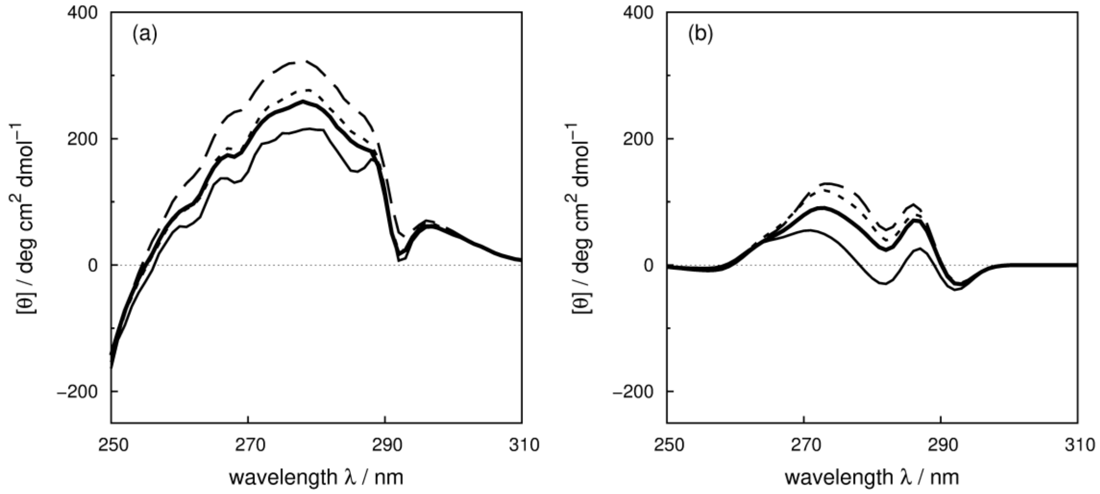

Figure S37. CD spectra of wild-type barnase (bold solid line) and mutants Y78F (solid line), Y90F (dotted line) and Y97F (dashed line); (a) experimental spectra and (b) calculated spectra.

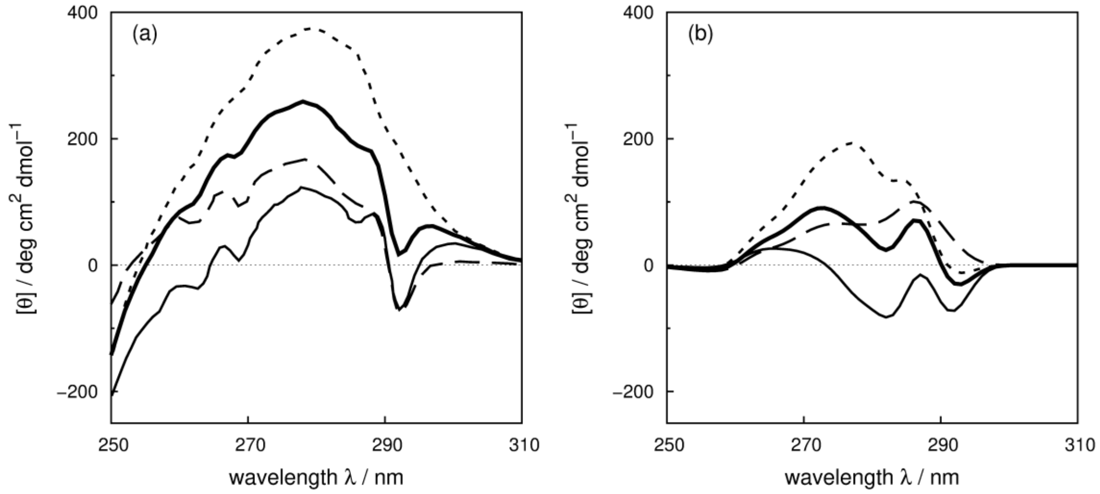

Figure S38. CD spectra of wild-type barnase (bold solid line) and mutants W35F (solid line), W71F (dotted line) and W94F (dashed line); (a) experimental spectra and (b) calculated spectra.

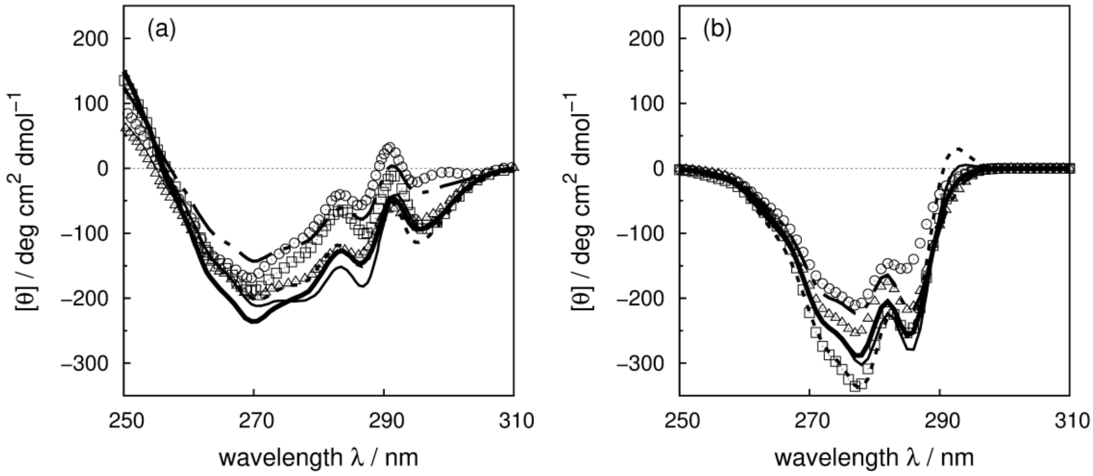

Figure S39. CD spectra of wild-type human carbonic anhydrase II (bold solid line) and mutants W5F (solid line), W16F (square), W97C (circle), W123C (triangle), W209F (dotted line) and W245C (dot-dashed line); (a) experiment and (b) calculation.

Decoy sets of 34 proteins and the Spearman rank correlation distribution.

3DRobot exported two scores ranging between zero and one to characterize and evaluate the evenness and the diversity of the generated decoys (Table S9).[56] An evenness score of unity corresponds to an even distribution of the decoys into bins with a 1 Å interval for the requested RMSD range. An evenness score of zero represents the extreme case that all decoys fall within a single bin. Decoy sets of 20 proteins have an evenness score greater than 0.9, i.e., the distribution of decoys is appropriate. The lowest evenness score is for rhodanese (1DP2, 0.551) with around 700 decoys accumulating in the 2 and 3 Å bins. The decoy sets of three proteins, relaxin (6RLX), tryptophan synthase α-subunit (1WQ5) and human carbonic anhydrase II (2CBA), with evenness scores less than 0.8 are either lacking decoys in the near-native state (RMSD < 3 Å) or in the distant-native end (RMSD above 7 Å). The other score, termed the normalized pair-wise RMSD (npwRMSD), reflects the diversity of the decoys. The npwRMSD is calculated with a function obtained using a set of random non-redundant proteins with low sequence identity as a reference pool to eliminate the dependence of pair-wise RMSD of decoys on their structural similarity with the native structure. The decoys which have low RMSD to the native structure tend to have low pair-wise RMSD due to structural similarity. The decoy sets of all the proteins have an npwRMSD over 0.85 corresponding to good diversity.

Table S9. The 34 proteins studied: name, PDB codes, evenness score, npwRMSD.

Protein	PDB entry	evenness	npwRMSD
adenylate kinase	2ECK	0.879	0.990
α-lactalbumin	1A4V	0.900	0.953
apolipophorin III	1AEP	0.943	0.990
barnase	1A2P	0.911	0.953
β-2 microglobulin (human)	1LDS	0.912	0.959
β-lactamase	1BTL	0.835	0.987
bovine pancreatic trypsin inhibitor	5PTI	0.916	0.895
calmodulin	4CLN	0.911	0.931
cardiotoxin	4OM4	0.911	0.949
chymotrypsinogen A	2CGA	0.797	0.946
dehydroquinase II	2BT4	0.918	0.980
dihydrofolate reductase	4P3Q	0.914	0.988
hen egg white lysozyme	1HF4	0.900	0.971
human carbonic anhydrase II	2CBA	0.719	0.980
insulin	5ENA	0.825	0.874
interleukin 4 (cytokine)	2B8U	0.911	0.974
interleukin 6	1ALU	0.874	0.955
monellin	1IV9	0.923	0.933
myoglobin (whale)	1UFP	0.895	0.978
neocarzinostatin	1NOA	0.933	0.977
odorant binding protein	1A3Y	0.911	0.979
papain	3LFY	0.902	0.982
phosphatidylethanolamine-binding protein	1A44	0.864	0.992
phospholipase A2 (Ca^{2+})	1PSJ	0.917	0.924
relaxin	6RLX	0.733	0.864
rhodanese	1DP2	0.551	0.998
ribonuclease T1	1RN1	0.909	0.971
ribonuclease A	1AFU	0.907	1.007
staphylococcal nuclease	1STN	0.909	0.996
extracellular domain of human tissue factor	2HFT	0.838	0.975
sticholysin II	1O72	0.815	0.991
subtilisin BPN’	1ST2	0.818	0.979
thioredoxin	2TRX	0.932	0.993
tryptophan synthase α-subunit	1WQ5	0.784	0.918

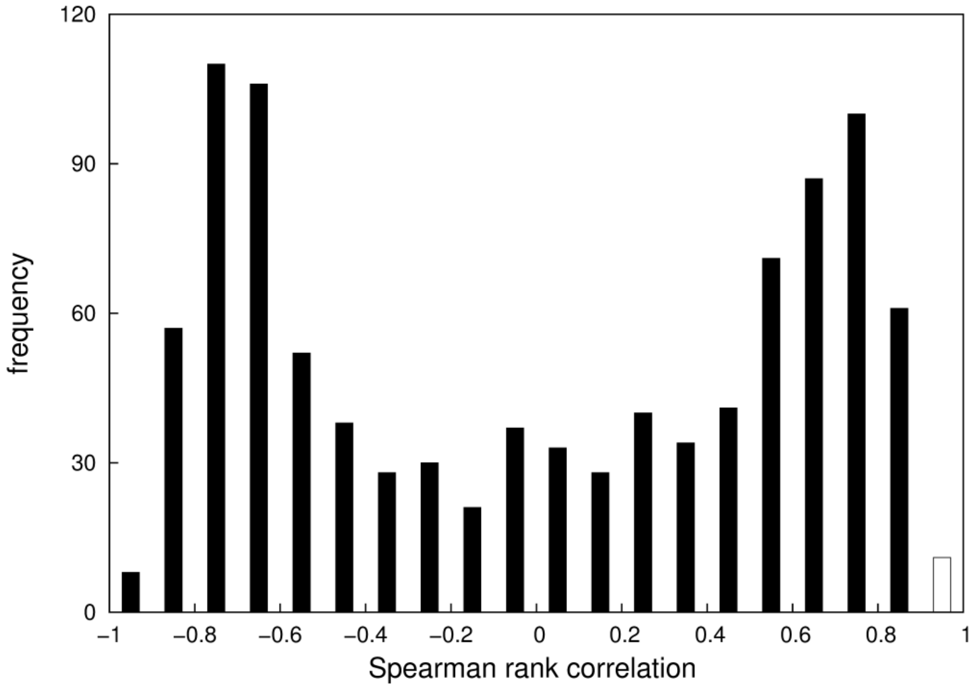

Figure S40. Papain (PDB code: 3LFY) represents the proteins whose Spearman rank correlation of the native structure in decoys set falls in the upper 10%. The open bar shows where the native structure appears in the Spearman rank correlation distribution of the native and the decoy structures. Another 14 proteins (42% of the proteins in decoy study) share a similar rank position as papain with distinct Spearman rank correlation distribution behavior of the decoy structures. The Spearman rank correlation of the native structure in this group ranges from 0.71 to 0.96.

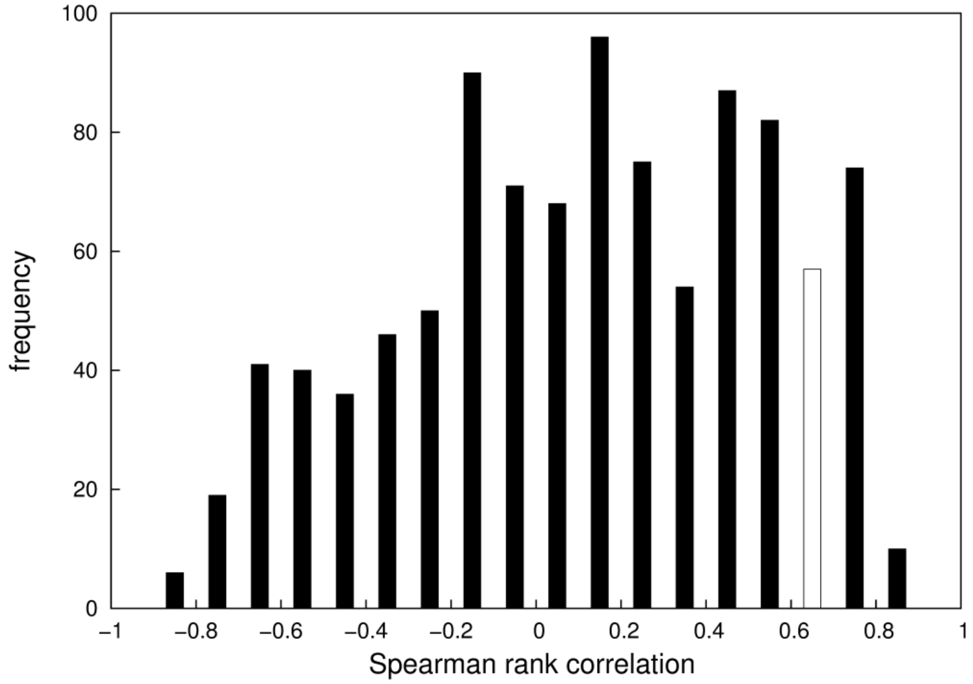

Figure S41. Dihydrofolate reductase (PDB code: 4P3Q) represents the proteins whose Spearman rank correlation of the native structure in decoys set falls in the upper 25%. The open bar shows where the native structure appears in the Spearman rank correlation distribution of the native and the decoy structures. Another three proteins share a similar rank position as dihydrofolate reductase with distinct Spearman rank correlation distribution behavior of the decoy structures. The Spearman rank correlation of the native structure in this group ranges from 0.59 to 0.82.

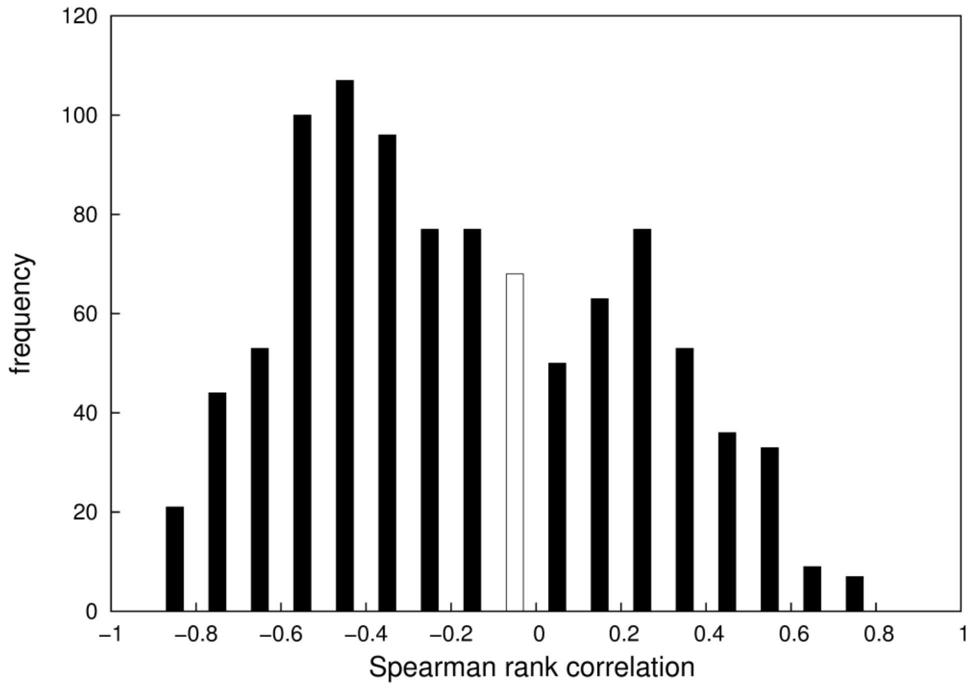

Figure S42. Rhodanese (PDB code: 1DP2) represents the proteins whose Spearman rank correlation of the native structure in decoys set falls in the upper 50%. The open bar shows where the native structure appears in the Spearman rank correlation distribution of the native and the decoy structures. Another seven proteins share a similar rank position as rhodanese with distinct Spearman rank correlation distribution behavior of the decoy structures. The Spearman rank correlation of the native structure in this group ranges from -0.38 to 0.76.

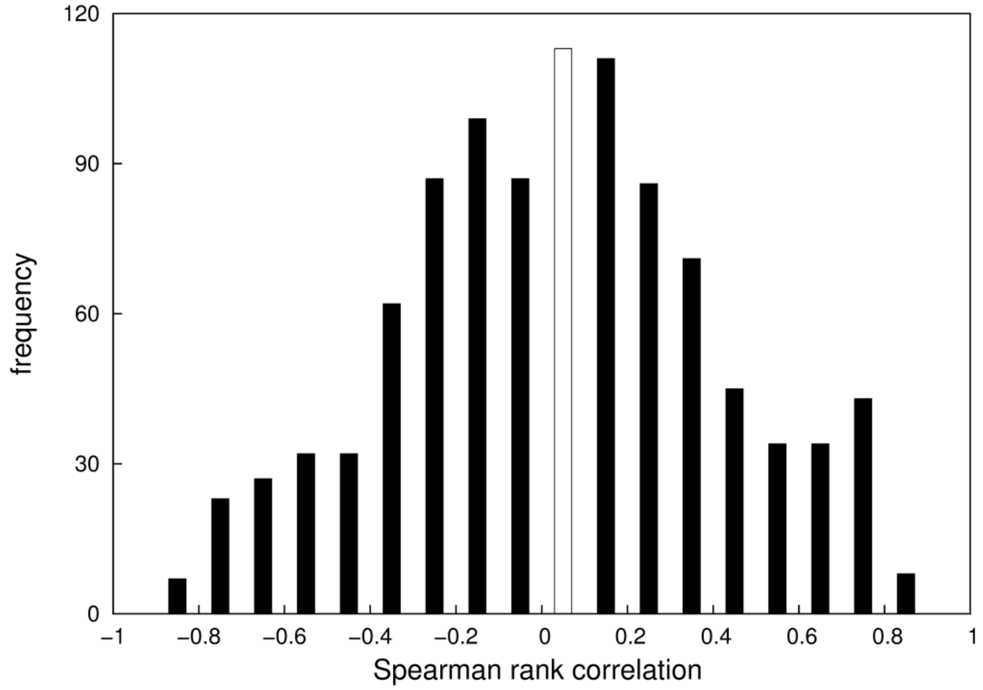

Figure S43. Dehydroquinase II (PDB code: 2BT4) represents the proteins whose Spearman rank correlation of the native structure in decoys set falls in the bottom 50%. The open bar shows where the native structure appears in the Spearman rank correlation distribution of the native and the decoy structures. Another six proteins share a similar rank position as dehydroquinase II with distinct Spearman rank correlation distribution behavior of the decoy structures. Three proteins (2B8U, 1STN and 1WQ5) fall in the bottom 25% of the Spearman rank correlation distribution. The Spearman rank correlation of the native structure in this group ranges from -0.80 to 0.01.

Reference

1. Seip, R.; Schultz, G.; Hargittai, I.; Szabo, Z. G. *Z. Naturforsch. A* **1977,** *32*, 1178.

2. Lau, C. L.; Snyder, R. G. *Spectrochim. Acta Mol. Biomol. Spectrosc.* **1971,** *A 27*, 2073.

3. Hickman, C. G.; Gascooke, J. R.; Lawrance, W. D. *J. Chem. Phys.* **1996,** *104*, 4887.

4. Pitzer, K. S.; Scott, D. W. *J. Am. Chem. Soc.* **1943,** *65*, 803.

5. Hameka, H. F.; Jensen, J. O. *THEOCHEM-J. Mol. Struc.* **1995,** *331*, 203.

6. Varsanyi, G. *Assignments for vibrational spectra of seven hundred benzene derivatives.* Lang, L.., Eds.; Wiley: New York, 1974.

7. Bois, C. *Acta Crystallogr., Sect. B: Struct. Crystallogr. Cryst. Chem* **1970,** *B 26*, 2086.

8. Arp, Z.; Autrey, D.; Laane, J.; Overman, S. A.; Thomas, G. J. *Biochemistry* **2001,** *40*, 2522.

9. Balachandran, V.; Murugan, M.; Nataraj, A.; Karnan, M.; Ilango, G. *Spectrochim. Acta Mol. Biomol. Spectrosc.* **2014,** *132*, 538.

10. Jakobsen, R. J. *Spectrochim. Acta* **1965,** *21*, 433.

11. Jensen, G. M.; Goodin, D. B.; Bunte, S. W. *J. Phys. Chem.* **1996,** *100*, 954.

12. Combs, A.; McCann, K.; Autrey, D.; Laane, J.; Overman, S. A.; Thomas, G. J. *J. Mol. Struct.* **2005,** *735*, 271.

13. Bunte, S. W.; Jensen, G. M.; McNesby, K. L.; Goodin, D. B.; Chabalowski, C. F.; Nieminen, R. M.; Suhai, S.; Jalkanen, K. J. *Chem. Phys.* **2001,** *265*, 13.

14. Chadwick, D. J. Pyrroles and their Benzo Derivatives: (i) Structure. In *Comprehensive Heterocyclic Chemistry: The Structure, Reactions, Synthesis, and Uses of Heterocyclic Compounds*. Bird, C. W. Cheeseman, G. W. H., Eds.; Pergammon: Oxford, 1984; Vol. 4, pp155.

15. Somers, K. R. F.; Ceulemans, A. *J. Phys. Chem. A* **2004,** *108*, 7577.

16. Jean, L.; Lee, C. F.; Shaw, M.; Vaux, D. J. *Plos One* **2008,** *3*, e1834.

17. Monnot, M.; Gilles, A. M.; Saintgirons, I.; Michelson, S.; Barzu, O.; Fermandjian, S. *J. Biol. Chem.* **1987,** *262*, 2502.

18. Permyakov, S. E.; Pershikova, I. V.; Zhadan, A. P.; Goers, J.; Bakunts, A. G.; Uversky, V. N.; Berliner, L. J.; Permyakov, E. A. *J. Proteome Res.* **2005,** *4*, 564.

19. Naylor, C. E.; Jepson, M.; Crane, D. T.; Titball, R. W.; Miller, J.; Basak, A. K.; Bolgiano, B. *J. Mol. Biol.* **1999,** *294*, 757.

20. Weers, P. M. M.; Kay, C. M.; Oikawa, K.; Wientzek, M.; Vanderhorst, D. J.; Ryan, R. O. *Biochemistry* **1994,** *33*, 3617.

21. Vuilleumier, S.; Sancho, J.; Loewenthal, R.; Fersht, A. R. *Biochemistry* **1993,** *32*, 10303.

22. Brown, E. M.; Groves, M. L. *Febs Lett.* **1985,** *184*, 36.

23. Vanhove, M.; Raquet, X.; Frere, J. M. *Proteins: Struct., Funct., Genet.* **1995,** *22*, 110.

24. Bolgiano, B.; Crane, D. T.; Xing, D.; Williams, L.; Jones, C.; Corbel, M. J. *Biologicals* **1999,** *27*, 155.

24. Sreerama, N.; Manning, M. C.; Powers, M. E.; Zhang, J. X.; Goldenberg, D. P.; Woody, R. W. *Biochemistry* **1999,** *38*, 10814.

25. Findlay, W. A.; Martin, S. R.; Beckingham, K.; Bayley, P. M. *Biochemistry* **1995,** *34*, 2087.

26. Galat, A.; Yang, C. C.; Blout, E. R. *Biochemistry* **1985,** *24*, 5678.

27. Khan, F.; Khan, R. H.; Muzammil, S. *BBA-Protein Struct. M* **2000,** *1481*, 229.

28. Krell, T.; Horsburgh, M. J.; Cooper, A.; Kelly, S. M.; Coggins, J. R. *J. Biol. Chem.* **1996,** *271*,

24492.
29. Svensson, A. K. E.; O'Neill, J. C.; Matthews, C. R. *J. Mol. Biol.* **2003,** *326*, 569.
30. Ranjbar, B.; Gill, P. *Chem. Biol. Drug Des.* **2009,** *74,* 101.
31. Buck, M.; Radford, S. E.; Dobson, C. M. *Biochemistry* **1993,** *32*, 669.
32. Freskgård, P.-O.; Martensson, L. G.; Jonasson, P.; Jonsson, B. H.; Carlsson, U. *Biochemistry* **1994,** *33*, 14281.
34. Sjoholm, I.; Ljungste, I. *J. Biol. Chem.* **1973,** *248*, 8434.
35. Hinds, K.; Koh, J. J.; Joss, L.; Liu, F.; Baudys, M.; Kim, S. W., *Bioconjugate Chem.* **2000,** *11*, 195.
36. Windsor, W. T.; Syto, R.; Le, H. V.; Trotta, P. P. *Biochemistry* **1991,** *30*, 1259.
37. Matthews, J. M.; Hammacher, A.; Howlett, G. J.; Simpson, R. J. *Biochemistry* **1998,** *37*, 10671.
38. Patra, A. K.; Udgaonkar, J. B. *Biochemistry* **2007,** *46*, 11727.
39. Sirangelo, I.; Bismuto, E.; Tavassi, S.; Irace, G. *Eur. Biophys. J. Biophy* **1998,** *27*, 27.
40. Chi, H.-W.; Chien, Y.-C.; Liu, C.-Y.; Tseng, C.-J.; Lee, Y.-J.; Chan, J.-L.; Chu, Y.-R.; Chin, D.-H. *Chem.-Eur. J.* **2011,** *17*, 1493.
41. Parisi, M.; Mazzini, A.; Sorbi, R. T.; Ramoni, R.; Grolli, S.; Favilla, R. *BBA-Proteins Proteom.* **2003,** *1652*, 115.
42. Edwin, F.; Jagannadham, M. V. *Biochem. Biophys. Res. Commun.* **1998,** *252*, 654.
43. Kamen, D. E.; Woody, R. W. *Prot. Sci.* **2001,** *10*, 2123.
44. Vallee, B.; Teyssier, C.; Maget-Dana, R.; Ramstein, J.; Bureaud, N.; Schoentgen, F. *Eur. J. Biochem.* **1999,** *266*, 40.
45. Ikeda, K.; Samejima, Y. *J. Biochem. (Tokyo, Jpn.)* **1981,** *89*, 1175.
46. Shire, S. J.; Holladay, L. A.; Rinderknecht, E. *Biochemistry* **1991,** *30*, 7703.
47. Cannella, C.; Costa, M.; Pensa, B.; Ricci, G.; Pecci, L.; Cavallini, D. *Eur. J. Biochem.* **1981,** *119*, 491.
48. Shastry, M. C. R.; Eftink, M. R. *Biochemistry* **1996,** *35*, 4094.
49. Woody, A. Y. M.; Woody, R. W. *Biopolymers* **2003,** *72*, 500.
50. Filfil, R.; Chalikian, T. V. *J. Mol. Biol.* **2000,** *299*, 827.
51. Andersson, D.; Carlsson, U.; Freskgård, P. O. *Eur. J. Biochem.* **2001,** *268*, 1118.
52. Mancheno, J. M.; De Los Rios, V.; Del Pozo, A. M.; Lanio, M. E.; Onaderra, M.; Gavilanes, J. G. *BBA-Protein Struct. M.* **2001,** *1545*, 122.
53. Eder, J.; Rheinnecker, M.; Fersht, A. R. *Biochemistry* **1993,** *32*, 18.
54. Georgescu, R. E.; Braswell, E. H.; Zhu, D.; Tasayco, M. L. *Biochemistry* **1999,** *38*, 13355.
55. Yutani, K.; Ogasahara, K.; Suzuki, M.; Sugino, Y. *J. Biochem. (Tokyo, Jpn.)* **1980,** *87*, 117.
56. Deng, H.; Jia, Y.; Zhang, Y. *Bioinformatics*. **2016,** *32*, 378.
