# Supplementary material for: Quantitative first principles calculations of protein circular dichroism in the near-ultraviolet
Source: Chem Sci. 2017 Mar 24;8(6):4318–33. doi: 10.1039/c7sc00586e (PMC5637123; doi:10.1039/c7sc00586e)
Supplement: Supplementary file 2 [file SC-008-C7SC00586E-s002.pdf]

Electronic Supplementary Material (ESI) for Chemical Science.
This journal is © The Royal Society of Chemistry 2017

```
 -PHEVIB00-
 6 # Phenylalanine_aromatic group / toluene
     1.39425895     0.00000067     0.00000000 1.0   # CG
     0.69713224     1.20746431     0.00000000 1.0   # CD1
    -0.69712835     1.20746137     0.00000000 1.0   # CE1
    -1.39426188    -0.00000070     0.00000000 1.0   # CZ
    -0.69713089    -1.20746326     0.00000000 1.0   # CE2
     0.69712932    -1.20746240     0.00000000 1.0   # CD2
&TRANSITION 1->...
 30    37310.0       # 268.0 nm,    (r2r1) 30 = 1Lb_v1 state
      0.00001159     0.23600000     0.00000000     1.0     # electr. mom.
      0.00000000     0.00000000     0.1066111229E-05      # magnet. mom.
      0.69712898    -1.20746181     0.00000000    -96.86030786
      1.39425828     0.00000067     0.00000000     -0.00222740
      0.69713190     1.20746372     0.00000000     96.85548927
     -0.69712801     1.20746079     0.00000000     96.85909911
     -1.39426121    -0.00000070     0.00000000     -0.01043734
     -0.69713055    -1.20746268     0.00000000    -96.84177463
      0.64421128    -1.20746181     0.00000000     93.27933698
      0.75004668    -1.20746181     0.00000000     85.24548231
      0.69712898    -1.26037951     0.00000000    -37.94013694
      0.69712898    -1.15454411     0.00000000    -43.76980229
      1.34134058     0.00000067     0.00000000      0.00067741
      1.44717598     0.00000067     0.00000000      0.00072899
      1.39425828    -0.05291703     0.00000000     -4.83905616
      1.39425828     0.05291837     0.00000000      4.83985668
      0.64421420     1.20746372     0.00000000    -93.27575739
      0.75004960     1.20746372     0.00000000    -85.24281091
      0.69713190     1.15454602     0.00000000     43.76924654
      0.69713190     1.26038142     0.00000000     37.93937250
     -0.75004571     1.20746079     0.00000000    -85.24580920
     -0.64421031     1.20746079     0.00000000    -93.27965303
     -0.69712801     1.15454309     0.00000000     43.77081240
     -0.69712801     1.26037849     0.00000000     37.94099971
     -1.44717891    -0.00000070     0.00000000      0.00199195
     -1.34134351    -0.00000070     0.00000000      0.00234255
     -1.39426121    -0.05291840     0.00000000     -4.83626168
     -1.39426121     0.05291700     0.00000000      4.84239554
     -0.75004826    -1.20746268     0.00000000     85.23912532
     -0.64421285    -1.20746268     0.00000000     93.27187296
     -0.69713055    -1.26038038     0.00000000    -37.94226491
     -0.69713055    -1.15454498     0.00000000    -43.77253092
 30    37879.0       # 264.0 nm,    (r2r1) 30 = 1Lb_v2 state
      0.00001159    -0.17200000     0.00000000     1.0     # electr. mom.
      0.00000000     0.00000000     0.1066111229E-05      # magnet. mom.
      0.69712898    -1.20746181     0.00000000     70.59310573
      1.39425828     0.00000067     0.00000000      0.00162336
      0.69713190     1.20746372     0.00000000    -70.58959387
     -0.69712801     1.20746079     0.00000000    -70.59222477
     -1.39426121    -0.00000070     0.00000000      0.00760687
     -0.69713055    -1.20746268     0.00000000     70.57959846
      0.64421128    -1.20746181     0.00000000    -67.98324559
      0.75004668    -1.20746181     0.00000000    -62.12806338
      0.69712898    -1.26037951     0.00000000     27.65128625
      0.69712898    -1.15454411     0.00000000     31.90002540
      1.34134058     0.00000067     0.00000000     -0.00049371
      1.44717598     0.00000067     0.00000000     -0.00053130
      1.39425828    -0.05291703     0.00000000      3.52676974
      1.39425828     0.05291837     0.00000000     -3.52735317
      0.64421420     1.20746372     0.00000000     67.98063674
      0.75004960     1.20746372     0.00000000     62.12611643
      0.69713190     1.15454602     0.00000000    -31.89962036
      0.69713190     1.26038142     0.00000000    -27.65072911
     -0.75004571     1.20746079     0.00000000     62.12830162
     -0.64421031     1.20746079     0.00000000     67.98347594
     -0.69712801     1.15454309     0.00000000    -31.90076158
     -0.69712801     1.26037849     0.00000000    -27.65191504
     -1.44717891    -0.00000070     0.00000000     -0.00145176
     -1.34134351    -0.00000070     0.00000000     -0.00170728
     -1.39426121    -0.05291840     0.00000000      3.52473309
     -1.39426121     0.05291700     0.00000000     -3.52920353
     -0.75004826    -1.20746268     0.00000000    -62.12343032
     -0.64421285    -1.20746268     0.00000000    -67.97780572
     -0.69713055    -1.26038038     0.00000000     27.65283714
     -0.69713055    -1.15454498     0.00000000     31.90201406
 30    38287.0       # 261.2 nm,    (r2r1) 30 = 1Lb_v3 state
      0.00001159     0.19600000     0.00000000     1.0     # electr. mom.
      0.0000000      0.00000000     0.1066111229E-05      # magnet. mom.
      0.69712898    -1.20746181     0.00000000    -80.44330653
      1.39425828     0.00000067     0.00000000     -0.00184987
      0.69713190     1.20746372     0.00000000     80.43930465
     -0.69712801     1.20746079     0.00000000     80.44230265
     -1.39426121    -0.00000070     0.00000000     -0.00866830
     -0.69713055    -1.20746268     0.00000000    -80.42791452
      0.64421128    -1.20746181     0.00000000     77.46927986
      0.75004668    -1.20746181     0.00000000     70.79709548
      0.69712898    -1.26037951     0.00000000    -31.50960526
```

	0.69712898	-1.15454411	0.00000000	-36.35119173	
	1.34134058	0.00000067	0.00000000	0.00056260	
	1.44717598	0.00000067	0.00000000	0.00060543	
	1.39425828	-0.05291703	0.00000000	-4.01887715	
	1.39425828	0.05291837	0.00000000	4.01954199	
	0.64421420	1.20746372	0.00000000	-77.46630698	
	0.75004960	1.20746372	0.00000000	-70.79487686	
	0.69713190	1.15454602	0.00000000	36.35073017	
	0.69713190	1.26038142	0.00000000	31.50897038	
	-0.75004571	1.20746079	0.00000000	-70.79736697	
	-0.64421031	1.20746079	0.00000000	-77.46954235	
	-0.69712801	1.15454309	0.00000000	36.35203064	
	-0.69712801	1.26037849	0.00000000	31.51032179	
	-1.44717891	-0.00000070	0.00000000	0.00165433	
	-1.34134351	-0.00000070	0.00000000	0.00194551	
	-1.39426121	-0.05291840	0.00000000	-4.01655631	
	-1.39426121	0.05291700	0.00000000	4.02165053	
	-0.75004826	-1.20746268	0.00000000	70.79181594	
	-0.64421285	-1.20746268	0.00000000	77.46308093	
	-0.69713055	-1.26038038	0.00000000	-31.51137255	
	-0.69713055	-1.15454498	0.00000000	-36.35345788	
30	38805.0	# 257.7 nm, (r2r1) 30 = 1Lb_v4 state			
	0.00001159	-0.14000000	0.00000000	1.0	# electr. mom.
	0.00000000	0.00000000	0.1066111229E-05		# magnet. mom.
	0.69712898	-1.20746181	0.00000000	57.45950467	
	1.39425828	0.00000067	0.00000000	0.00132134	
	0.69713190	1.20746372	0.00000000	-57.45664618	
	-0.69712801	1.20746079	0.00000000	-57.45878761	
	-1.39426121	-0.00000070	0.00000000	0.00619164	
	-0.69713055	-1.20746268	0.00000000	57.44851037	
	0.64421128	-1.20746181	0.00000000	-55.33519990	
	0.75004668	-1.20746181	0.00000000	-50.56935391	
	0.69712898	-1.26037951	0.00000000	22.50686090	
	0.69712898	-1.15454411	0.00000000	25.96513695	
	1.34134058	0.00000067	0.00000000	-0.00040186	
	1.44717598	0.00000067	0.00000000	-0.00043245	
	1.39425828	-0.05291703	0.00000000	2.87062654	
	1.39425828	0.05291837	0.00000000	-2.87110142	
	0.64421420	1.20746372	0.00000000	55.33307642	
	0.75004960	1.20746372	0.00000000	50.56776919	
	0.69713190	1.15454602	0.00000000	-25.96480727	
	0.69713190	1.26038142	0.00000000	-22.50640741	
	-0.75004571	1.20746079	0.00000000	50.56954783	
	-0.64421031	1.20746079	0.00000000	55.33538739	
	-0.69712801	1.15454309	0.00000000	-25.96573617	
	-0.69712801	1.26037849	0.00000000	-22.50737271	
	-1.44717891	-0.00000070	0.00000000	-0.00118166	
	-1.34134351	-0.00000070	0.00000000	-0.00138965	
	-1.39426121	-0.05291840	0.00000000	2.86896880	
	-1.39426121	0.05291700	0.00000000	-2.87260752	
	-0.75004826	-1.20746268	0.00000000	-50.56558282	
	-0.64421285	-1.20746268	0.00000000	-55.33077209	
	-0.69713055	-1.26038038	0.00000000	22.50812325	
	-0.69713055	-1.15454498	0.00000000	25.96675563	
30	39263.0	# 254.7 nm, (r2r1) 30 = 1Lb_v5 state			
	0.00001159	0.10400000	0.00000000	1.0	# electr. mom.
	0.00000000	0.00000000	0.1066111229E-05		# magnet. mom.
	0.69712898	-1.20746181	0.00000000	-42.68420347	
	1.39425828	0.00000067	0.00000000	-0.00098157	
	0.69713190	1.20746372	0.00000000	42.68208002	
	-0.69712801	1.20746079	0.00000000	42.68367079	
	-1.39426121	-0.00000070	0.00000000	-0.00459950	
	-0.69713055	-1.20746268	0.00000000	-42.67603628	
	0.64421128	-1.20746181	0.00000000	41.10614850	
	0.75004668	-1.20746181	0.00000000	37.56580576	
	0.69712898	-1.26037951	0.00000000	-16.71938238	
	0.69712898	-1.15454411	0.00000000	-19.28838745	
	1.34134058	0.00000067	0.00000000	0.00029852	
	1.44717598	0.00000067	0.00000000	0.00032125	
	1.39425828	-0.05291703	0.00000000	-2.13246543	
	1.39425828	0.05291837	0.00000000	2.13281820	
	0.64421420	1.20746372	0.00000000	-41.10457105	
	0.75004960	1.20746372	0.00000000	-37.56462854	
	0.69713190	1.15454602	0.00000000	19.28814254	
	0.69713190	1.26038142	0.00000000	16.71904551	
	-0.75004571	1.20746079	0.00000000	-37.56594982	
	-0.64421031	1.20746079	0.00000000	-41.10628778	
	-0.69712801	1.15454309	0.00000000	19.28883258	
	-0.69712801	1.26037849	0.00000000	16.71976258	
	-1.44717891	-0.00000070	0.00000000	0.00087781	
	-1.34134351	-0.00000070	0.00000000	0.00103231	
	-1.39426121	-0.05291840	0.00000000	-2.13123396	
	-1.39426121	0.05291700	0.00000000	2.13393702	
	-0.75004826	-1.20746268	0.00000000	37.56300438	
	-0.64421285	-1.20746268	0.00000000	41.10285927	
	-0.69713055	-1.26038038	0.00000000	-16.72032013	
	-0.69713055	-1.15454498	0.00000000	-19.28958990	

30 39714.0 # 251.8 nm, (r2r1) 30 = 1Lb_v6 state
0.00001159 -0.07200000 0.00000000 1.0 # electr. mom.
0.00000000 0.00000000 0.1066111229E-05 # magnet. mom.
0.69712898 -1.20746181 0.00000000 29.55060240
1.39425828 0.00000067 0.00000000 0.00067955
0.69713190 1.20746372 0.00000000 -29.54913232
-0.69712801 1.20746079 0.00000000 -29.55023363
-1.39426121 -0.00000070 0.00000000 0.00318427
-0.69713055 -1.20746268 0.00000000 29.54494819
0.64421128 -1.20746181 0.00000000 -28.45810281
0.75004668 -1.20746181 0.00000000 -26.00709630
0.69712898 -1.26037951 0.00000000 11.57495703
0.69712898 -1.15454411 0.00000000 13.35349900
1.34134058 0.00000067 0.00000000 -0.00020667
1.44717598 0.00000067 0.00000000 -0.00022240
1.39425828 -0.05291703 0.00000000 1.47632222
1.39425828 0.05291837 0.00000000 -1.47656644
0.64421420 1.20746372 0.00000000 28.45701073
0.75004960 1.20746372 0.00000000 26.00628130
0.69713190 1.15454602 0.00000000 -13.35332945
0.69713190 1.26038142 0.00000000 -11.57472381
-0.75004571 1.20746079 0.00000000 26.00719603
-0.64421031 1.20746079 0.00000000 28.45819923
-0.69712801 1.15454309 0.00000000 -13.35380717
-0.69712801 1.26037849 0.00000000 -11.57522050
-1.44717891 -0.00000070 0.00000000 -0.00060771
-1.34134351 -0.00000070 0.00000000 -0.00071468
-1.39426121 -0.05291840 0.00000000 1.47546967
-1.39426121 0.05291700 0.00000000 -1.47734101
-0.75004826 -1.20746268 0.00000000 -26.00515688
-0.64421285 -1.20746268 0.00000000 -28.45582565
-0.69713055 -1.26038038 0.00000000 11.57560624
-0.69713055 -1.15454498 0.00000000 13.35433147
30 47953.0 # 208.5 nm, (r3r1) 30 = 1La state
-0.00181497 0.00001159 0.00000000 1.0 # electr. mom.
0.00000000 0.00000000 -0.9176476175E-06 # magnet. mom.
0.69712898 -1.20746181 0.00000000 -720.10281331
1.39425828 0.00000067 0.00000000 676.46791718
0.69713190 1.20746372 0.00000000 -719.51123026
-0.69712801 1.20746079 0.00000000 719.55152597
-1.39426121 -0.00000070 0.00000000 -676.54491115
-0.69713055 -1.20746268 0.00000000 720.15377184
0.64421128 -1.20746181 0.00000000 189.43097314
0.75004668 -1.20746181 0.00000000 178.04942880
0.69712898 -1.26037951 0.00000000 169.00110437
0.69712898 -1.15454411 0.00000000 184.16175480
1.34134058 0.00000067 0.00000000 -183.99266875
1.44717598 0.00000067 0.00000000 -166.48118774
1.39425828 -0.05291703 0.00000000 -163.17095611
1.39425828 0.05291837 0.00000000 -163.16527032
0.64421420 1.20746372 0.00000000 189.00656837
0.75004960 1.20746372 0.00000000 177.69744029
0.69713190 1.15454602 0.00000000 184.26697454
0.69713190 1.26038142 0.00000000 169.07670418
-0.75004571 1.20746079 0.00000000 -177.76016923
-0.64421031 1.20746079 0.00000000 -189.07656043
-0.69712801 1.15454309 0.00000000 -184.21937510
-0.69712801 1.26037849 0.00000000 -169.03211760
-1.44717891 -0.00000070 0.00000000 166.44732014
-1.34134351 -0.00000070 0.00000000 183.96049147
-1.39426121 -0.05291840 0.00000000 163.24288157
-1.39426121 0.05291700 0.00000000 163.23688824
-0.75004826 -1.20746268 0.00000000 -178.11526142
-0.64421285 -1.20746268 0.00000000 -189.50389891
-0.69713055 -1.26038038 0.00000000 -168.95877480
-0.69713055 -1.15454498 0.00000000 -184.11655090
30 52322.0 # 191.1 nm, (r6r1) 30 = 1Ba state
-5.71534381 -0.00248626 0.00000000 1.0 # electr. mom.
0.0 0.0 -0.3115391048E-05 # magnet. mom.
0.69712898 -1.20746181 0.00000000 -392.69530416
1.39425828 0.00000067 0.00000000 -439.61855126
0.69713190 1.20746372 0.00000000 -370.02429158
-0.69712801 1.20746079 0.00000000 392.80345728
-1.39426121 -0.00000070 0.00000000 439.73613912
-0.69713055 -1.20746268 0.00000000 370.42051042
0.64421128 -1.20746181 0.00000000 245.95000790
0.75004668 -1.20746181 0.00000000 215.39198966
0.69712898 -1.26037951 0.00000000 -25.53486158
0.69712898 -1.15454411 0.00000000 -42.00365539
1.34134058 0.00000067 0.00000000 205.18413682
1.44717598 0.00000067 0.00000000 180.76575312
1.39425828 -0.05291703 0.00000000 22.57429123
1.39425828 0.05291837 0.00000000 31.71871666
0.64421420 1.20746372 0.00000000 230.77598078
0.75004960 1.20746372 0.00000000 193.55789659
0.69713190 1.15454602 0.00000000 -36.94518475
0.69713190 1.26038142 0.00000000 -16.81912488

```
      -0.75004571      1.20746079      0.00000000    -215.40469354
      -0.64421031      1.20746079      0.00000000    -245.95670050
      -0.69712801      1.15454309      0.00000000      41.96337346
      -0.69712801      1.26037849      0.00000000      25.48769650
      -1.44717891     -0.00000070      0.00000000    -180.82241486
      -1.34134351     -0.00000070      0.00000000    -205.24329826
      -1.39426121     -0.05291840      0.00000000     -31.71334325
      -1.39426121      0.05291700      0.00000000     -22.58131157
      -0.75004826     -1.20746268      0.00000000    -193.72087320
      -0.64421285     -1.20746268      0.00000000    -230.94680266
      -0.69713055     -1.26038038      0.00000000      16.78989144
      -0.69713055     -1.15454498      0.00000000      36.91056950
 30     52532.0   # 190.4 nm,   (r7r1) 30 = 1Bb state
      -0.00093506    5.73201442    0.00000000 1.0         # electr. mom.
       0.0            0.0           -0.2505031327E-03      # magnet. mom.
       0.69712898     -1.20746181      0.00000000    -309.50876290
       1.39425828      0.00000067      0.00000000     -29.17040098
       0.69713190      1.20746372      0.00000000     685.77686261
      -0.69712801      1.20746079      0.00000000     309.59074200
      -1.39426121     -0.00000070      0.00000000      29.30014138
      -0.69713055     -1.20746268      0.00000000    -685.91994019
       0.64421128     -1.20746181      0.00000000      10.97927957
       0.75004668     -1.20746181      0.00000000      37.38172162
       0.69712898     -1.26037951      0.00000000     123.03838987
       0.69712898     -1.15454411      0.00000000     136.18476466
       1.34134058      0.00000067      0.00000000      21.76816522
       1.44717598      0.00000067      0.00000000      19.54822925
       1.39425828     -0.05291703      0.00000000     -12.50673437
       1.39425828      0.05291837      0.00000000       0.19187083
       0.64421420      1.20746372      0.00000000    -201.76558306
       0.75004960      1.20746372      0.00000000    -182.09675491
       0.69713190      1.15454602      0.00000000    -155.85576653
       0.69713190      1.26038142      0.00000000    -147.12570941
      -0.75004571      1.20746079      0.00000000     -37.41335160
      -0.64421031      1.20746079      0.00000000     -11.02902158
      -0.69712801      1.15454309      0.00000000    -136.18006138
      -0.69712801      1.26037849      0.00000000    -123.04418227
      -1.44717891     -0.00000070      0.00000000     -19.58466312
      -1.34134351     -0.00000070      0.00000000     -21.80622221
      -1.39426121     -0.05291840      0.00000000      -0.21497419
      -1.39426121      0.05291700      0.00000000      12.47465251
      -0.75004826     -1.20746268      0.00000000     182.13462413
      -0.64421285     -1.20746268      0.00000000     201.82307318
      -0.69713055     -1.26038038      0.00000000     147.15209285
      -0.69713055     -1.15454498      0.00000000     155.87752099
&TRANSITION 2->...
 0     0.0   # (r2r2) 30
   0.0   0.0     0.0 1.0          # electr. mom.
                 0.0   0.0   0.0 # magnet. mom.
 0     0.0   # (r2r2) 30
   0.0   0.0     0.0 1.0          # electr. mom.
                 0.0   0.0   0.0 # magnet. mom.
 0     0.0   # (r2r2) 30
   0.0   0.0     0.0 1.0          # electr. mom.
                 0.0   0.0   0.0 # magnet. mom.
 0     0.0   # (r2r2) 30
   0.0   0.0     0.0 1.0          # electr. mom.
                 0.0   0.0   0.0 # magnet. mom.
 0     0.0   # (r2r2) 30
   0.0   0.0     0.0 1.0          # electr. mom.
                 0.0   0.0   0.0 # magnet. mom.
 0     0.0   # (r3r2) 30
   0.0     0.0     0.0 1.0        # electr. mom.
                 0.0   0.0   0.0 # magnet. mom.
 0     0.0   # (r6r2) 30
   0.0     0.0     0.0 1.0        # electr. mom.
                 0.0   0.0   0.0 # magnet. mom.
 0     0.0   # (r7r2) 30
   0.0     0.0     0.0 1.0        # electr. mom.
                 0.0   0.0   0.0 # magnet. mom.
&TRANSITION 3->...
 0     0.0   # (r2r2) 30
   0.0   0.0     0.0 1.0          # electr. mom.
                 0.0   0.0   0.0 # magnet. mom.
 0     0.0   # (r2r2) 30
   0.0   0.0     0.0 1.0          # electr. mom.
                 0.0   0.0   0.0 # magnet. mom.
 0     0.0   # (r2r2) 30
   0.0   0.0     0.0 1.0          # electr. mom.
                 0.0   0.0   0.0 # magnet. mom.
 0     0.0   # (r2r2) 30
   0.0   0.0     0.0 1.0          # electr. mom.
                 0.0   0.0   0.0 # magnet. mom.
 0     0.0   # (r3r2) 30
   0.0   0.0     0.0 1.0          # electr. mom.
                 0.0   0.0   0.0 # magnet. mom.
 0     0.0   # (r6r2) 30
```

```
    0.0      0.0      0.0 1.0              # electr. mom.
                         0.0    0.0    0.0 # magnet. mom.
 0      0.0    # (r7r2) 30
    0.0      0.0      0.0 1.0              # electr. mom.
                         0.0    0.0    0.0 # magnet. mom.
&TRANSITION 4->...
 0      0.0    # (r2r2) 30
    0.0    0.0      0.0 1.0                # electr. mom.
                         0.0    0.0    0.0 # magnet. mom.
 0      0.0    # (r2r2) 30
    0.0    0.0      0.0 1.0                # electr. mom.
                         0.0    0.0    0.0 # magnet. mom.
 0      0.0    # (r2r2) 30
    0.0    0.0      0.0 1.0                # electr. mom.
                         0.0    0.0    0.0 # magnet. mom.
 0      0.0    # (r3r2) 30
    0.0    0.0      0.0 1.0                # electr. mom.
                         0.0    0.0    0.0 # magnet. mom.
 0      0.0    # (r6r2) 30
      0.0    0.0      0.0 1.0              # electr. mom.
                         0.0    0.0    0.0 # magnet. mom.
 0      0.0    # (r7r2) 30
    0.0      0.0      0.0 1.0              # electr. mom.
                         0.0    0.0    0.0 # magnet. mom.
&TRANSITION 5->...
 0      0.0    # (r2r2) 30
    0.0    0.0      0.0 1.0                # electr. mom.
                         0.0    0.0    0.0 # magnet. mom.
 0      0.0    # (r2r2) 30
    0.0    0.0      0.0 1.0                # electr. mom.
                         0.0    0.0    0.0 # magnet. mom.
 0      0.0    # (r3r2) 30
    0.0    0.0      0.0 1.0                # electr. mom.
                         0.0    0.0    0.0 # magnet. mom.
 0      0.0    # (r6r2) 30
    0.0    0.0      0.0 1.0                # electr. mom.
                         0.0    0.0    0.0 # magnet. mom.
 0      0.0    # (r7r2) 30
    0.0    0.0      0.0 1.0              # electr. mom.
                         0.0    0.0 0.0 # magnet. mom.
&TRANSITION 6->...
 0      0.0    # (r2r2) 30
    0.0    0.0      0.0 1.0                # electr. mom.
                         0.0    0.0    0.0 # magnet. mom.
 0      0.0    # (r3r2) 30
    0.0    0.0      0.0 1.0                # electr. mom.
                         0.0    0.0    0.0 # magnet. mom.
 0      0.0    # (r6r2) 30
    0.0    0.0      0.0 1.0                # electr. mom.
                         0.0    0.0    0.0 # magnet. mom.
 0      0.0    # (r7r2) 30
    0.0    0.0      0.0 1.0                # electr. mom.
                         0.0    0.0    0.0 # magnet. mom.
&TRANSITION 7->...
 0      0.0    # (r3r2) 30
    0.0    0.0      0.0 1.0                # electr. mom.
                         0.0    0.0    0.0 # magnet. mom.
 0      0.0    # (r6r2) 30
    0.0    0.0      0.0 1.0                # electr. mom.
                         0.0    0.0    0.0 # magnet. mom.
 0      0.0    # (r7r2) 30
    0.0    0.0      0.0 1.0                # electr. mom.
                         0.0    0.0    0.0 # magnet. mom.
&TRANSITION 8->...
 0      0.0    # (r6r3) 30
    0.0    0.0      0.0 1.0                # electr. mom.
                         0.0    0.0    0.0 # magnet. mom.
 0      0.0    # (r7r3) 30
    0.0    0.0      0.0 1.0                # electr. mom.
                         0.0    0.0    0.0 # magnet. mom.
&TRANSITION 9->...
 0      0.0    # (r7r6) 30
    0.0    0.0      0.0 1.0                # electr. mom.
                         0.0    0.0    0.0 # magnet. mom.
&PERMANENT MOMENTS
 30        0.0                  # 0 nm, Grd. (r1r1) 30
         -0.00016991      0.00016902      0.00000000 1.0 # electr. mom.
           0.69712898         -1.20746181           0.00000000    -1072.58045587
           1.39425828           0.00000067           0.00000000    -2512.11222830
           0.69713190           1.20746372           0.00000000    -1191.59753688
          -0.69712801           1.20746079           0.00000000    -1073.00910576
          -1.39426121          -0.00000070           0.00000000    -2512.25643950
          -0.69713055          -1.20746268           0.00000000    -1191.95144218
           0.64421128          -1.20746181           0.00000000       421.14125304
           0.75004668          -1.20746181           0.00000000       396.28081896
           0.69712898          -1.26037951           0.00000000       136.54235846
           0.69712898          -1.15454411           0.00000000       116.16425837
```

1.34134058 0.00000067 0.00000000 340.56728323
1.44717598 0.00000067 0.00000000 297.21605093
1.39425828 -0.05291703 0.00000000 927.34693128
1.39425828 0.05291837 0.00000000 953.47234675
0.64421420 1.20746372 0.00000000 312.41237410
0.75004960 1.20746372 0.00000000 275.15783717
0.69713190 1.15454602 0.00000000 286.75977466
0.69713190 1.26038142 0.00000000 313.22575584
-0.75004571 1.20746079 0.00000000 396.53957155
-0.64421031 1.20746079 0.00000000 421.43850562
-0.69712801 1.15454309 0.00000000 116.09598274
-0.69712801 1.26037849 0.00000000 136.48603714
-1.44717891 -0.00000070 0.00000000 297.27514522
-1.34134351 -0.00000070 0.00000000 340.64352682
-1.39426121 -0.05291840 0.00000000 953.48065656
-1.39426121 0.05291700 0.00000000 927.34883222
-0.75004826 -1.20746268 0.00000000 275.37223637
-0.64421285 -1.20746268 0.00000000 312.66985104
-0.69713055 -1.26038038 0.00000000 313.17469018
-0.69713055 -1.15454498 0.00000000 286.69513022
30 0.0 # 0 nm, 1st Exc. (r2r2) 30
-0.00014141 0.00029094 0.00000000 1.0 # electr. mom.
0.69712898 -1.20746181 0.00000000 -994.42567205
1.39425828 0.00000067 0.00000000 70.45827883
0.69713190 1.20746372 0.00000000 -1027.25930923
-0.69712801 1.20746079 0.00000000 -994.26996144
-1.39426121 -0.00000070 0.00000000 70.25247806
-0.69713055 -1.20746268 0.00000000 -1027.09117330
0.64421128 -1.20746181 0.00000000 248.83619779
0.75004668 -1.20746181 0.00000000 267.78514637
0.69712898 -1.26037951 0.00000000 233.31463915
0.69712898 -1.15454411 0.00000000 246.28641547
1.34134058 0.00000067 0.00000000 264.39424315
1.44717598 0.00000067 0.00000000 277.70519323
1.39425828 -0.05291703 0.00000000 -307.89995021
1.39425828 0.05291837 0.00000000 -308.56462056
0.64421420 1.20746372 0.00000000 269.89306666
0.75004960 1.20746372 0.00000000 285.65241802
0.69713190 1.15454602 0.00000000 243.56387453
0.69713190 1.26038142 0.00000000 230.26174354
-0.75004571 1.20746079 0.00000000 267.67396085
-0.64421031 1.20746079 0.00000000 248.70106392
-0.69712801 1.15454309 0.00000000 246.32938694
-0.69712801 1.26037849 0.00000000 233.36048458
-1.44717891 -0.00000070 0.00000000 277.77610963
-1.34134351 -0.00000070 0.00000000 264.47018626
-1.39426121 -0.05291840 0.00000000 -308.53441090
-1.39426121 0.05291700 0.00000000 -307.87068562
-0.75004826 -1.20746268 0.00000000 285.56298317
-0.64421285 -1.20746268 0.00000000 269.78279482
-0.69713055 -1.26038038 0.00000000 230.27790874
-0.69713055 -1.15454498 0.00000000 243.57720965
30 0.0 # 0 nm, 1st Exc. (r2r2) 30
-0.00014141 0.00029094 0.00000000 1.0 # electr. mom.
0.69712898 -1.20746181 0.00000000 -994.42567205
1.39425828 0.00000067 0.00000000 70.45827883
0.69713190 1.20746372 0.00000000 -1027.25930923
-0.69712801 1.20746079 0.00000000 -994.26996144
-1.39426121 -0.00000070 0.00000000 70.25247806
-0.69713055 -1.20746268 0.00000000 -1027.09117330
0.64421128 -1.20746181 0.00000000 248.83619779
0.75004668 -1.20746181 0.00000000 267.78514637
0.69712898 -1.26037951 0.00000000 233.31463915
0.69712898 -1.15454411 0.00000000 246.28641547
1.34134058 0.00000067 0.00000000 264.39424315
1.44717598 0.00000067 0.00000000 277.70519323
1.39425828 -0.05291703 0.00000000 -307.89995021
1.39425828 0.05291837 0.00000000 -308.56462056
0.64421420 1.20746372 0.00000000 269.89306666
0.75004960 1.20746372 0.00000000 285.65241802
0.69713190 1.15454602 0.00000000 243.56387453
0.69713190 1.26038142 0.00000000 230.26174354
-0.75004571 1.20746079 0.00000000 267.67396085
-0.64421031 1.20746079 0.00000000 248.70106392
-0.69712801 1.15454309 0.00000000 246.32938694
-0.69712801 1.26037849 0.00000000 233.36048458
-1.44717891 -0.00000070 0.00000000 277.77610963
-1.34134351 -0.00000070 0.00000000 264.47018626
-1.39426121 -0.05291840 0.00000000 -308.53441090
-1.39426121 0.05291700 0.00000000 -307.87068562
-0.75004826 -1.20746268 0.00000000 285.56298317
-0.64421285 -1.20746268 0.00000000 269.78279482
-0.69713055 -1.26038038 0.00000000 230.27790874
-0.69713055 -1.15454498 0.00000000 243.57720965
30 0.0 # 0 nm, 1st Exc. (r2r2) 30
-0.00014141 0.00029094 0.00000000 1.0 # electr. mom.
0.69712898 -1.20746181 0.00000000 -994.42567205

	1.39425828	0.00000067	0.00000000	70.45827883
	0.69713190	1.20746372	0.00000000	-1027.25930923
	-0.69712801	1.20746079	0.00000000	-994.26996144
	-1.39426121	-0.00000070	0.00000000	70.25247806
	-0.69713055	-1.20746268	0.00000000	-1027.09117330
	0.64421128	-1.20746181	0.00000000	248.83619779
	0.75004668	-1.20746181	0.00000000	267.78514637
	0.69712898	-1.26037951	0.00000000	233.31463915
	0.69712898	-1.15454411	0.00000000	246.28641547
	1.34134058	0.00000067	0.00000000	264.39424315
	1.44717598	0.00000067	0.00000000	277.70519323
	1.39425828	-0.05291703	0.00000000	-307.89995021
	1.39425828	0.05291837	0.00000000	-308.56462056
	0.64421420	1.20746372	0.00000000	269.89306666
	0.75004960	1.20746372	0.00000000	285.65241802
	0.69713190	1.15454602	0.00000000	243.56387453
	0.69713190	1.26038142	0.00000000	230.26174354
	-0.75004571	1.20746079	0.00000000	267.67396085
	-0.64421031	1.20746079	0.00000000	248.70106392
	-0.69712801	1.15454309	0.00000000	246.32938694
	-0.69712801	1.26037849	0.00000000	233.36048458
	-1.44717891	-0.00000070	0.00000000	277.77610963
	-1.34134351	-0.00000070	0.00000000	264.47018626
	-1.39426121	-0.05291840	0.00000000	-308.53441090
	-1.39426121	0.05291700	0.00000000	-307.87068562
	-0.75004826	-1.20746268	0.00000000	285.56298317
	-0.64421285	-1.20746268	0.00000000	269.78279482
	-0.69713055	-1.26038038	0.00000000	230.27790874
	-0.69713055	-1.15454498	0.00000000	243.57720965
30	0.0 # 0 nm, 1st Exc. (r2r2) 30			
	-0.00014141	0.00029094	0.00000000 1.0 # electr. mom.	
	0.69712898	-1.20746181	0.00000000	-994.42567205
	1.39425828	0.00000067	0.00000000	70.45827883
	0.69713190	1.20746372	0.00000000	-1027.25930923
	-0.69712801	1.20746079	0.00000000	-994.26996144
	-1.39426121	-0.00000070	0.00000000	70.25247806
	-0.69713055	-1.20746268	0.00000000	-1027.09117330
	0.64421128	-1.20746181	0.00000000	248.83619779
	0.75004668	-1.20746181	0.00000000	267.78514637
	0.69712898	-1.26037951	0.00000000	233.31463915
	0.69712898	-1.15454411	0.00000000	246.28641547
	1.34134058	0.00000067	0.00000000	264.39424315
	1.44717598	0.00000067	0.00000000	277.70519323
	1.39425828	-0.05291703	0.00000000	-307.89995021
	1.39425828	0.05291837	0.00000000	-308.56462056
	0.64421420	1.20746372	0.00000000	269.89306666
	0.75004960	1.20746372	0.00000000	285.65241802
	0.69713190	1.15454602	0.00000000	243.56387453
	0.69713190	1.26038142	0.00000000	230.26174354
	-0.75004571	1.20746079	0.00000000	267.67396085
	-0.64421031	1.20746079	0.00000000	248.70106392
	-0.69712801	1.15454309	0.00000000	246.32938694
	-0.69712801	1.26037849	0.00000000	233.36048458
	-1.44717891	-0.00000070	0.00000000	277.77610963
	-1.34134351	-0.00000070	0.00000000	264.47018626
	-1.39426121	-0.05291840	0.00000000	-308.53441090
	-1.39426121	0.05291700	0.00000000	-307.87068562
	-0.75004826	-1.20746268	0.00000000	285.56298317
	-0.64421285	-1.20746268	0.00000000	269.78279482
	-0.69713055	-1.26038038	0.00000000	230.27790874
	-0.69713055	-1.15454498	0.00000000	243.57720965
30	0.0 # 0 nm, 1st Exc. (r2r2) 30			
	-0.00014141	0.00029094	0.00000000 1.0 # electr. mom.	
	0.69712898	-1.20746181	0.00000000	-994.42567205
	1.39425828	0.00000067	0.00000000	70.45827883
	0.69713190	1.20746372	0.00000000	-1027.25930923
	-0.69712801	1.20746079	0.00000000	-994.26996144
	-1.39426121	-0.00000070	0.00000000	70.25247806
	-0.69713055	-1.20746268	0.00000000	-1027.09117330
	0.64421128	-1.20746181	0.00000000	248.83619779
	0.75004668	-1.20746181	0.00000000	267.78514637
	0.69712898	-1.26037951	0.00000000	233.31463915
	0.69712898	-1.15454411	0.00000000	246.28641547
	1.34134058	0.00000067	0.00000000	264.39424315
	1.44717598	0.00000067	0.00000000	277.70519323
	1.39425828	-0.05291703	0.00000000	-307.89995021
	1.39425828	0.05291837	0.00000000	-308.56462056
	0.64421420	1.20746372	0.00000000	269.89306666
	0.75004960	1.20746372	0.00000000	285.65241802
	0.69713190	1.15454602	0.00000000	243.56387453
	0.69713190	1.26038142	0.00000000	230.26174354
	-0.75004571	1.20746079	0.00000000	267.67396085
	-0.64421031	1.20746079	0.00000000	248.70106392
	-0.69712801	1.15454309	0.00000000	246.32938694
	-0.69712801	1.26037849	0.00000000	233.36048458
	-1.44717891	-0.00000070	0.00000000	277.77610963
	-1.34134351	-0.00000070	0.00000000	264.47018626

	-1.39426121	-0.05291840	0.00000000	-308.53441090
	-1.39426121	0.05291700	0.00000000	-307.87068562
	-0.75004826	-1.20746268	0.00000000	285.56298317
	-0.64421285	-1.20746268	0.00000000	269.78279482
	-0.69713055	-1.26038038	0.00000000	230.27790874
	-0.69713055	-1.15454498	0.00000000	243.57720965
30	0.0 # 0 nm, 1st Exc. (r2r2) 30			
	-0.00014141	0.00029094	0.00000000 1.0 # electr. mom.	
	0.69712898	-1.20746181	0.00000000	-994.42567205
	1.39425828	0.00000067	0.00000000	70.45827883
	0.69713190	1.20746372	0.00000000	-1027.25930923
	-0.69712801	1.20746079	0.00000000	-994.26996144
	-1.39426121	-0.00000070	0.00000000	70.25247806
	-0.69713055	-1.20746268	0.00000000	-1027.09117330
	0.64421128	-1.20746181	0.00000000	248.83619779
	0.75004668	-1.20746181	0.00000000	267.78514637
	0.69712898	-1.26037951	0.00000000	233.31463915
	0.69712898	-1.15454411	0.00000000	246.28641547
	1.34134058	0.00000067	0.00000000	264.39424315
	1.44717598	0.00000067	0.00000000	277.70519323
	1.39425828	-0.05291703	0.00000000	-307.89995021
	1.39425828	0.05291837	0.00000000	-308.56462056
	0.64421420	1.20746372	0.00000000	269.89306666
	0.75004960	1.20746372	0.00000000	285.65241802
	0.69713190	1.15454602	0.00000000	243.56387453
	0.69713190	1.26038142	0.00000000	230.26174354
	-0.75004571	1.20746079	0.00000000	267.67396085
	-0.64421031	1.20746079	0.00000000	248.70106392
	-0.69712801	1.15454309	0.00000000	246.32938694
	-0.69712801	1.26037849	0.00000000	233.36048458
	-1.44717891	-0.00000070	0.00000000	277.77610963
	-1.34134351	-0.00000070	0.00000000	264.47018626
	-1.39426121	-0.05291840	0.00000000	-308.53441090
	-1.39426121	0.05291700	0.00000000	-307.87068562
	-0.75004826	-1.20746268	0.00000000	285.56298317
	-0.64421285	-1.20746268	0.00000000	269.78279482
	-0.69713055	-1.26038038	0.00000000	230.27790874
	-0.69713055	-1.15454498	0.00000000	243.57720965
30	0.0 # 0 nm, 2nd Exc. (r3r3) 30			
	-0.00291686	0.00618851	0.00000000 1.0 # electr. mom.	
	0.69712898	-1.20746181	0.00000000	-1160.35437240
	1.39425828	0.00000067	0.00000000	0.65288045
	0.69713190	1.20746372	0.00000000	-1183.39483694
	-0.69712801	1.20746079	0.00000000	-1159.22585184
	-1.39426121	-0.00000070	0.00000000	1.06361414
	-0.69713055	-1.20746268	0.00000000	-1183.55918016
	0.64421128	-1.20746181	0.00000000	302.23646904
	0.75004668	-1.20746181	0.00000000	320.88800918
	0.69712898	-1.26037951	0.00000000	263.22523834
	0.69712898	-1.15454411	0.00000000	275.89216536
	1.34134058	0.00000067	0.00000000	298.97769768
	1.44717598	0.00000067	0.00000000	311.91559786
	1.39425828	-0.05291703	0.00000000	-307.53548021
	1.39425828	0.05291837	0.00000000	-308.01692510
	0.64421420	1.20746372	0.00000000	317.24999784
	0.75004960	1.20746372	0.00000000	333.57399547
	0.69713190	1.15454602	0.00000000	273.78813000
	0.69713190	1.26038142	0.00000000	260.90269171
	-0.75004571	1.20746079	0.00000000	320.43349238
	-0.64421031	1.20746079	0.00000000	301.70304682
	-0.69712801	1.15454309	0.00000000	275.83288973
	-0.69712801	1.26037849	0.00000000	263.14759411
	-1.44717891	-0.00000070	0.00000000	311.97391877
	-1.34134351	-0.00000070	0.00000000	299.03338090
	-1.39426121	-0.05291840	0.00000000	-308.23744008
	-1.39426121	0.05291700	0.00000000	-307.83870389
	-0.75004826	-1.20746268	0.00000000	333.53316130
	-0.64421285	-1.20746268	0.00000000	317.21538720
	-0.69713055	-1.26038038	0.00000000	261.03370613
	-0.69713055	-1.15454498	0.00000000	273.88972622
30	0.0 # 0 nm, 3rd Exc. (r6r6) 30			
	-0.00209383	0.00210373	0.00000000 1.0 # electr. mom.	
	0.69712898	-1.20746181	0.00000000	-1625.45239752
	1.39425828	0.00000067	0.00000000	497.75810496
	0.69713190	1.20746372	0.00000000	-1069.73659243
	-0.69712801	1.20746079	0.00000000	-1625.19639250
	-1.39426121	-0.00000070	0.00000000	498.20869032
	-0.69713055	-1.20746268	0.00000000	-1069.44016906
	0.64421128	-1.20746181	0.00000000	662.90515440
	0.75004668	-1.20746181	0.00000000	621.40324646
	0.69712898	-1.26037951	0.00000000	166.05997075
	0.69712898	-1.15454411	0.00000000	183.33685344
	1.34134058	0.00000067	0.00000000	93.45928954
	1.44717598	0.00000067	0.00000000	151.60749418
	1.39425828	-0.05291703	0.00000000	-381.73751136
	1.39425828	0.05291837	0.00000000	-369.28409467
	0.64421420	1.20746372	0.00000000	-80.38651325

	0.75004960	1.20746372	0.00000000	2.85851866
	0.69713190	1.15454602	0.00000000	605.14385203
	0.69713190	1.26038142	0.00000000	542.06716320
	-0.75004571	1.20746079	0.00000000	621.20486338
	-0.64421031	1.20746079	0.00000000	662.62374528
	-0.69712801	1.15454309	0.00000000	183.46406256
	-0.69712801	1.26037849	0.00000000	166.15773706
	-1.44717891	-0.00000070	0.00000000	151.64625110
	-1.34134351	-0.00000070	0.00000000	93.47459890
	-1.39426121	-0.05291840	0.00000000	-369.51418541
	-1.39426121	0.05291700	0.00000000	-382.01476685
	-0.75004826	-1.20746268	0.00000000	2.66895854
	-0.64421285	-1.20746268	0.00000000	-80.61205546
	-0.69713055	-1.26038038	0.00000000	542.12078112
	-0.69713055	-1.15454498	0.00000000	605.20534267
30	0.0 # 0 nm, 4th Exc. (r7r7) 30			
	-0.00279529	0.00122626	0.00000000 1.0 # electr. mom.	
	0.69712898	-1.20746181	0.00000000	-1485.80130346
	1.39425828	0.00000067	0.00000000	562.67742797
	0.69713190	1.20746372	0.00000000	-1297.94006429
	-0.69712801	1.20746079	0.00000000	-1483.39510200
	-1.39426121	-0.00000070	0.00000000	563.36592624
	-0.69713055	-1.20746268	0.00000000	-1297.70400782
	0.64421128	-1.20746181	0.00000000	600.82047154
	0.75004668	-1.20746181	0.00000000	571.56760531
	0.69712898	-1.26037951	0.00000000	152.69549866
	0.69712898	-1.15454411	0.00000000	167.74937333
	1.34134058	0.00000067	0.00000000	110.78831779
	1.44717598	0.00000067	0.00000000	167.76991891
	1.39425828	-0.05291703	0.00000000	-428.33415221
	1.39425828	0.05291837	0.00000000	-421.18621190
	0.64421420	1.20746372	0.00000000	58.36522406
	0.75004960	1.20746372	0.00000000	125.06726664
	0.69713190	1.15454602	0.00000000	588.66257765
	0.69713190	1.26038142	0.00000000	527.10449424
	-0.75004571	1.20746079	0.00000000	570.70570570
	-0.64421031	1.20746079	0.00000000	599.83544770
	-0.69712801	1.15454309	0.00000000	167.45762002
	-0.69712801	1.26037849	0.00000000	152.42147006
	-1.44717891	-0.00000070	0.00000000	167.61254544
	-1.34134351	-0.00000070	0.00000000	110.61341242
	-1.39426121	-0.05291840	0.00000000	-421.35570106
	-1.39426121	0.05291700	0.00000000	-428.51997322
	-0.75004826	-1.20746268	0.00000000	124.83602256
	-0.64421285	-1.20746268	0.00000000	58.06349035
	-0.69713055	-1.26038038	0.00000000	527.24740550
	-0.69713055	-1.15454498	0.00000000	588.80929392

```
  -TYRVIB00-
 7 # Tyrosine_aromatic group / p-cresol
      1.23557695    -0.00671127     0.00000000 1.0   # CZ
      0.54866976     1.20505781     0.00000000 1.0   # CE1
      0.53489462    -1.20761830     0.00000000 1.0   # CE2
     -0.84197834     1.20827128     0.00000000 1.0   # CD1
     -0.86024633    -1.19659626     0.00000000 1.0   # CD2
     -1.55492874     0.00867527     0.00000000 1.0   # CG
      2.59151053     0.04298367     0.00000000 1.0   # O
&TRANSITION 1->...
 35     34965.0   # 286.0 nm,   (r2r1) = 1Lb_v1 state
     -0.0501        -0.6199         0.00000000 1.0       # electr. mom.
      0.0            0.0            0.2174085006         # magnet. mom.
      2.59150928     0.04298365     0.00000000    -70.70654815
      0.53489436    -1.20761772     0.00000000     12.07940493
      1.23557636    -0.00671127     0.00000000    257.62759180
      0.54866949     1.20505723     0.00000000    -21.75079997
     -0.84197793     1.20827070     0.00000000   -227.36804401
     -1.55492799     0.00867526     0.00000000     80.48459892
     -0.86024592    -1.19659568     0.00000000    133.88839299
      2.53859158     0.04298365     0.00000000     43.32020886
      2.64442698     0.04298365     0.00000000     32.24828912
      2.59150928    -0.00993405     0.00000000     -2.39473768
      2.59150928     0.09590135     0.00000000     -1.45341104
      0.48197666    -1.20761772     0.00000000     -4.77916543
      0.58781206    -1.20761772     0.00000000    -13.66943915
      0.53489436    -1.26053542     0.00000000      3.02588363
      0.53489436    -1.15470002     0.00000000      4.44985547
      1.18265866    -0.00671127     0.00000000   -103.42184606
      1.28849406    -0.00671127     0.00000000   -116.28261585
      1.23557636    -0.05962897     0.00000000    -12.57351941
      1.23557636     0.04620643     0.00000000    -26.56496369
      0.49575179     1.20505723     0.00000000     42.36432705
      0.60158719     1.20505723     0.00000000     44.49361363
      0.54866949     1.15213953     0.00000000    -34.36574382
      0.54866949     1.25797493     0.00000000    -31.18111072
     -0.89489563     1.20827070     0.00000000    115.62363257
     -0.78906023     1.20827070     0.00000000    129.70222188
     -0.84197793     1.15535300     0.00000000     -9.72251335
     -0.84197793     1.26118840     0.00000000     -7.58223017
     -1.60784569     0.00867526     0.00000000    -14.78677182
     -1.50201029     0.00867526     0.00000000    -18.42308662
     -1.55492799    -0.04424244     0.00000000    -20.04967361
     -1.55492799     0.06159296     0.00000000    -27.60031832
     -0.91316362    -1.19659568     0.00000000   -114.37803284
     -0.80732822    -1.19659568     0.00000000   -135.29183835
     -0.86024592    -1.24951338     0.00000000     52.69904780
     -0.86024592    -1.14367798     0.00000000     62.33934143
 35     35765.0   # 279.6 nm,   (r2r1) = 1Lb_v2 state
     -0.0559        -0.6915         0.00000000 1.0       # electr. mom.
      0.0            0.0            0.2174085006         # magnet. mom.
      2.59150928     0.04298365     0.00000000    -78.86499601
      0.53489436    -1.20761772     0.00000000     13.47318242
      1.23557636    -0.00671127     0.00000000    287.35385239
      0.54866949     1.20505723     0.00000000    -24.26050766
     -0.84197793     1.20827070     0.00000000   -253.60281832
     -1.55492799     0.00867526     0.00000000     89.77128341
     -0.86024592    -1.19659568     0.00000000    149.33705372
      2.53859158     0.04298365     0.00000000     48.31869450
      2.64442698     0.04298365     0.00000000     35.96924556
      2.59150928    -0.00993405     0.00000000     -2.67105357
      2.59150928     0.09590135     0.00000000     -1.62111231
      0.48197666    -1.20761772     0.00000000     -5.33060760
      0.58781206    -1.20761772     0.00000000    -15.24668213
      0.53489436    -1.26053542     0.00000000      3.37502405
      0.53489436    -1.15470002     0.00000000      4.96330033
      1.18265866    -0.00671127     0.00000000   -115.35513599
      1.28849406    -0.00671127     0.00000000   -129.69984076
      1.23557636    -0.05962897     0.00000000    -14.02431011
      1.23557636     0.04620643     0.00000000    -29.63015181
      0.49575179     1.20505723     0.00000000     47.25251863
      0.60158719     1.20505723     0.00000000     49.62749213
      0.54866949     1.15213953     0.00000000    -38.33102195
      0.54866949     1.25797493     0.00000000    -34.77893119
     -0.89489563     1.20827070     0.00000000    128.96482094
     -0.78906023     1.20827070     0.00000000    144.66786286
     -0.84197793     1.15535300     0.00000000    -10.84434181
     -0.84197793     1.26118840     0.00000000     -8.45710288
     -1.60784569     0.00867526     0.00000000    -16.49293780
     -1.50201029     0.00867526     0.00000000    -20.54882739
     -1.55492799    -0.04424244     0.00000000    -22.36309749
     -1.55492799     0.06159296     0.00000000    -30.78497043
     -0.91316362    -1.19659568     0.00000000   -127.57549817
     -0.80732822    -1.19659568     0.00000000   -150.90243509
     -0.86024592    -1.24951338     0.00000000     58.77970716
     -0.86024592    -1.14367798     0.00000000     69.53234237
 35     36215.0   # 276.1 nm,   (r2r1) = 1Lb_v3 state
```

	-0.0164	-0.2027	0.00000000 1.0	# electr. mom.
	0.0	0.0	0.2174085006	# magnet. mom.
	2.59150928	0.04298365	0.00000000	-23.11560228
	0.53489436	-1.20761772	0.00000000	3.94903623
	1.23557636	-0.00671127	0.00000000	84.22440501
	0.54866949	1.20505723	0.00000000	-7.11083845
	-0.84197793	1.20827070	0.00000000	-74.33186054
	-1.55492799	0.00867526	0.00000000	26.31227272
	-0.86024592	-1.19659568	0.00000000	43.77120540
	2.53859158	0.04298365	0.00000000	14.16237597
	2.64442698	0.04298365	0.00000000	10.54270991
	2.59150928	-0.00993405	0.00000000	-0.78289501
	2.59150928	0.09590135	0.00000000	-0.47515361
	0.48197666	-1.20761772	0.00000000	-1.56241947
	0.58781206	-1.20761772	0.00000000	-4.46885511
	0.53489436	-1.26053542	0.00000000	0.98923119
	0.53489436	-1.15470002	0.00000000	1.45476044
	1.18265866	-0.00671127	0.00000000	-33.81098814
	1.28849406	-0.00671127	0.00000000	-38.01547057
	1.23557636	-0.05962897	0.00000000	-4.11057365
	1.23557636	0.04620643	0.00000000	-8.68469967
	0.49575179	1.20505723	0.00000000	13.84987615
	0.60158719	1.20505723	0.00000000	14.54598907
	0.54866949	1.15213953	0.00000000	-11.23495471
	0.54866949	1.25797493	0.00000000	-10.19382466
	-0.89489563	1.20827070	0.00000000	37.80003372
	-0.78906023	1.20827070	0.00000000	42.40264946
	-0.84197793	1.15535300	0.00000000	-3.17851398
	-0.84197793	1.26118840	0.00000000	-2.47880602
	-1.60784569	0.00867526	0.00000000	-4.83413694
	-1.50201029	0.00867526	0.00000000	-6.02293217
	-1.55492799	-0.04424244	0.00000000	-6.55470099
	-1.55492799	0.06159296	0.00000000	-9.02318099
	-0.91316362	-1.19659568	0.00000000	-37.39281843
	-0.80732822	-1.19659568	0.00000000	-44.23002408
	-0.86024592	-1.24951338	0.00000000	17.22853486
	-0.86024592	-1.14367798	0.00000000	20.38016931
35	36565.0	# 273.5 nm, (r2r1) = 1Lb_v4 state		
	-0.0433	-0.5365	0.00000000 1.0	# electr. mom.
	0.0	0.0	0.2174085006	# magnet. mom.
	2.59150928	0.04298365	0.00000000	-61.18835898
	0.53489436	-1.20761772	0.00000000	10.45333119
	1.23557636	-0.00671127	0.00000000	222.94695444
	0.54866949	1.20505723	0.00000000	-18.82280767
	-0.84197793	1.20827070	0.00000000	-196.76080732
	-1.55492799	0.00867526	0.00000000	69.65013368
	-0.86024592	-1.19659568	0.00000000	115.86495547
	2.53859158	0.04298365	0.00000000	37.48864229
	2.64442698	0.04298365	0.00000000	27.90717328
	2.59150928	-0.00993405	0.00000000	-2.07236915
	2.59150928	0.09590135	0.00000000	-1.25775955
	0.48197666	-1.20761772	0.00000000	-4.13581624
	0.58781206	-1.20761772	0.00000000	-11.82932234
	0.53489436	-1.26053542	0.00000000	2.61855314
	0.53489436	-1.15470002	0.00000000	3.85083647
	1.18265866	-0.00671127	0.00000000	-89.49967448
	1.28849406	-0.00671127	0.00000000	-100.62918680
	1.23557636	-0.05962897	0.00000000	-10.88093025
	1.23557636	0.04620643	0.00000000	-22.98891089
	0.49575179	1.20505723	0.00000000	36.66143687
	0.60158719	1.20505723	0.00000000	38.50408872
	0.54866949	1.15213953	0.00000000	-29.73958600
	0.54866949	1.25797493	0.00000000	-26.98365351
	-0.89489563	1.20827070	0.00000000	100.05891280
	-0.78906023	1.20827070	0.00000000	112.24230739
	-0.84197793	1.15535300	0.00000000	-8.41371348
	-0.84197793	1.26118840	0.00000000	-6.56154534
	-1.60784569	0.00867526	0.00000000	-12.79624485
	-1.50201029	0.00867526	0.00000000	-15.94305573
	-1.55492799	-0.04424244	0.00000000	-17.35067909
	-1.55492799	0.06159296	0.00000000	-23.88489085
	-0.91316362	-1.19659568	0.00000000	-98.98098996
	-0.80732822	-1.19659568	0.00000000	-117.07947550
	-0.86024592	-1.24951338	0.00000000	45.60494521
	-0.86024592	-1.14367798	0.00000000	53.94750701
35	37015.0	# 270.2 nm, (r2r1) = 1Lb_v5 state		
	-0.0173	-0.2146	0.00000000 1.0	# electr. mom.
	0.0	0.0	0.2174085006	# magnet. mom.
	2.59150928	0.04298365	0.00000000	-24.47534359
	0.53489436	-1.20761772	0.00000000	4.18133247
	1.23557636	-0.00671127	0.00000000	89.17878178
	0.54866949	1.20505723	0.00000000	-7.52912307
	-0.84197793	1.20827070	0.00000000	-78.70432293
	-1.55492799	0.00867526	0.00000000	27.86005347
	-0.86024592	-1.19659568	0.00000000	46.34598219
	2.53859158	0.04298365	0.00000000	14.99545691
	2.64442698	0.04298365	0.00000000	11.16286931

	2.59150928	-0.00993405	0.00000000	-0.82894766	
	2.59150928	0.09590135	0.00000000	-0.50310382	
	0.48197666	-1.20761772	0.00000000	-1.65432650	
	0.58781206	-1.20761772	0.00000000	-4.73172894	
	0.53489436	-1.26053542	0.00000000	1.04742126	
	0.53489436	-1.15470002	0.00000000	1.54033459	
	1.18265866	-0.00671127	0.00000000	-35.79986979	
	1.28849406	-0.00671127	0.00000000	-40.25167472	
	1.23557636	-0.05962897	0.00000000	-4.35237210	
	1.23557636	0.04620643	0.00000000	-9.19556436	
	0.49575179	1.20505723	0.00000000	14.66457475	
	0.60158719	1.20505723	0.00000000	15.40163549	
	0.54866949	1.15213953	0.00000000	-11.89583440	
	0.54866949	1.25797493	0.00000000	-10.79346140	
	-0.89489563	1.20827070	0.00000000	40.02356512	
	-0.78906023	1.20827070	0.00000000	44.89692296	
	-0.84197793	1.15535300	0.00000000	-3.36548539	
	-0.84197793	1.26118840	0.00000000	-2.62461813	
	-1.60784569	0.00867526	0.00000000	-5.11849794	
	-1.50201029	0.00867526	0.00000000	-6.37722229	
	-1.55492799	-0.04424244	0.00000000	-6.94027164	
	-1.55492799	0.06159296	0.00000000	-9.55395634	
	-0.91316362	-1.19659568	0.00000000	-39.59239598	
	-0.80732822	-1.19659568	0.00000000	-46.83179020	
	-0.86024592	-1.24951338	0.00000000	18.24197808	
	-0.86024592	-1.14367798	0.00000000	21.57900280	
35	37365.0	# 267.6 nm, (r2r1) = 1Lb_v6 state			
	-0.0270	-0.3338	0.00000000	1.0	# electr. mom.
	0.0	0.0	0.2174085006		# magnet. mom.
	2.59150928	0.04298365	0.00000000	-38.07275670	
	0.53489436	-1.20761772	0.00000000	6.50429496	
	1.23557636	-0.00671127	0.00000000	138.72254943	
	0.54866949	1.20505723	0.00000000	-11.71196922	
	-0.84197793	1.20827070	0.00000000	-122.42894677	
	-1.55492799	0.00867526	0.00000000	43.33786096	
	-0.86024592	-1.19659568	0.00000000	72.09375007	
	2.53859158	0.04298365	0.00000000	23.32626631	
	2.64442698	0.04298365	0.00000000	17.36446337	
	2.59150928	-0.00993405	0.00000000	-1.28947414	
	2.59150928	0.09590135	0.00000000	-0.78260594	
	0.48197666	-1.20761772	0.00000000	-2.57339677	
	0.58781206	-1.20761772	0.00000000	-7.36046723	
	0.53489436	-1.26053542	0.00000000	1.62932195	
	0.53489436	-1.15470002	0.00000000	2.39607602	
	1.18265866	-0.00671127	0.00000000	-55.68868634	
	1.28849406	-0.00671127	0.00000000	-62.61371623	
	1.23557636	-0.05962897	0.00000000	-6.77035660	
	1.23557636	0.04620643	0.00000000	-14.30421122	
	0.49575179	1.20505723	0.00000000	22.81156072	
	0.60158719	1.20505723	0.00000000	23.95809965	
	0.54866949	1.15213953	0.00000000	-18.50463129	
	0.54866949	1.25797493	0.00000000	-16.78982885	
	-0.89489563	1.20827070	0.00000000	62.25887907	
	-0.78906023	1.20827070	0.00000000	69.83965793	
	-0.84197793	1.15535300	0.00000000	-5.23519950	
	-0.84197793	1.26118840	0.00000000	-4.08273932	
	-1.60784569	0.00867526	0.00000000	-7.96210790	
	-1.50201029	0.00867526	0.00000000	-9.92012357	
	-1.55492799	-0.04424244	0.00000000	-10.79597810	
	-1.55492799	0.06159296	0.00000000	-14.86170986	
	-0.91316362	-1.19659568	0.00000000	-61.58817153	
	-0.80732822	-1.19659568	0.00000000	-72.84945142	
	-0.86024592	-1.24951338	0.00000000	28.37641035	
	-0.86024592	-1.14367798	0.00000000	33.56733769	
35	37815.0	# 264.5 nm, (r2r1) = 1Lb_v7 state			
	-0.0135	-0.1669	0.00000000	1.0	# electr. mom.
	0.0	0.0	0.2174085006		# magnet. mom.
	2.59150928	0.04298365	0.00000000	-19.03637835	
	0.53489436	-1.20761772	0.00000000	3.25214748	
	1.23557636	-0.00671127	0.00000000	69.36127471	
	0.54866949	1.20505723	0.00000000	-5.85598461	
	-0.84197793	1.20827070	0.00000000	-61.21447339	
	-1.55492799	0.00867526	0.00000000	21.66893048	
	-0.86024592	-1.19659568	0.00000000	36.04687504	
	2.53859158	0.04298365	0.00000000	11.66313316	
	2.64442698	0.04298365	0.00000000	8.68223169	
	2.59150928	-0.00993405	0.00000000	-0.64473707	
	2.59150928	0.09590135	0.00000000	-0.39130297	
	0.48197666	-1.20761772	0.00000000	-1.28669839	
	0.58781206	-1.20761772	0.00000000	-3.68023362	
	0.53489436	-1.26053542	0.00000000	0.81466098	
	0.53489436	-1.15470002	0.00000000	1.19803801	
	1.18265866	-0.00671127	0.00000000	-27.84434317	
	1.28849406	-0.00671127	0.00000000	-31.30685811	
	1.23557636	-0.05962897	0.00000000	-3.38517830	
	1.23557636	0.04620643	0.00000000	-7.15210561	
	0.49575179	1.20505723	0.00000000	11.40578036	

```
      0.60158719        1.20505723        0.00000000       11.97904982
      0.54866949        1.15213953        0.00000000       -9.25231564
      0.54866949        1.25797493        0.00000000       -8.39491442
     -0.89489563        1.20827070        0.00000000       31.12943954
     -0.78906023        1.20827070        0.00000000       34.91982897
     -0.84197793        1.15535300        0.00000000       -2.61759975
     -0.84197793        1.26118840        0.00000000       -2.04136966
     -1.60784569        0.00867526        0.00000000       -3.98105395
     -1.50201029        0.00867526        0.00000000       -4.96006178
     -1.55492799       -0.04424244        0.00000000       -5.39798905
     -1.55492799        0.06159296        0.00000000       -7.43085493
     -0.91316362       -1.19659568        0.00000000      -30.79408577
     -0.80732822       -1.19659568        0.00000000      -36.42472571
     -0.86024592       -1.24951338        0.00000000       14.18820518
     -0.86024592       -1.14367798        0.00000000       16.78366885
35    38165.0   # 262.0 nm,   (r2r1) = 1Lb_v8 state
     -0.0135        -0.1669        0.00000000 1.0      # electr. mom.
      0.0            0.0           0.2174085006        # magnet. mom.
      2.59150928        0.04298365        0.00000000      -19.03637835
      0.53489436       -1.20761772        0.00000000        3.25214748
      1.23557636       -0.00671127        0.00000000       69.36127471
      0.54866949        1.20505723        0.00000000       -5.85598461
     -0.84197793        1.20827070        0.00000000      -61.21447339
     -1.55492799        0.00867526        0.00000000       21.66893048
     -0.86024592       -1.19659568        0.00000000       36.04687504
      2.53859158        0.04298365        0.00000000       11.66313316
      2.64442698        0.04298365        0.00000000        8.68223169
      2.59150928       -0.00993405        0.00000000       -0.64473707
      2.59150928        0.09590135        0.00000000       -0.39130297
      0.48197666       -1.20761772        0.00000000       -1.28669839
      0.58781206       -1.20761772        0.00000000       -3.68023362
      0.53489436       -1.26053542        0.00000000        0.81466098
      0.53489436       -1.15470002        0.00000000        1.19803801
      1.18265866       -0.00671127        0.00000000      -27.84434317
      1.28849406       -0.00671127        0.00000000      -31.30685811
      1.23557636       -0.05962897        0.00000000       -3.38517830
      1.23557636        0.04620643        0.00000000       -7.15210561
      0.49575179        1.20505723        0.00000000       11.40578036
      0.60158719        1.20505723        0.00000000       11.97904982
      0.54866949        1.15213953        0.00000000       -9.25231564
      0.54866949        1.25797493        0.00000000       -8.39491442
     -0.89489563        1.20827070        0.00000000       31.12943954
     -0.78906023        1.20827070        0.00000000       34.91982897
     -0.84197793        1.15535300        0.00000000       -2.61759975
     -0.84197793        1.26118840        0.00000000       -2.04136966
     -1.60784569        0.00867526        0.00000000       -3.98105395
     -1.50201029        0.00867526        0.00000000       -4.96006178
     -1.55492799       -0.04424244        0.00000000       -5.39798905
     -1.55492799        0.06159296        0.00000000       -7.43085493
     -0.91316362       -1.19659568        0.00000000      -30.79408577
     -0.80732822       -1.19659568        0.00000000      -36.42472571
     -0.86024592       -1.24951338        0.00000000       14.18820518
     -0.86024592       -1.14367798        0.00000000       16.78366885
35   46205.0   # 216.4 nm,   (r3r1) = 1La state
      1.38590213   -0.15821049    0.00000000 1.0      # electr. mom.
      0.0            0.0          -0.7184570540E-01    # magnet. mom.
      2.59150928        0.04298365        0.00000000      144.12823848
      0.53489436       -1.20761772        0.00000000     -475.87423699
      1.23557636       -0.00671127        0.00000000      252.26197435
      0.54866949        1.20505723        0.00000000     -595.67921246
     -0.84197793        1.20827070        0.00000000      602.25412118
     -1.55492799        0.00867526        0.00000000     -825.48364584
     -0.86024592       -1.19659568        0.00000000      617.81960045
      2.53859158        0.04298365        0.00000000      -43.97312472
      2.64442698        0.04298365        0.00000000      -40.55833805
      2.59150928       -0.00993405        0.00000000      -29.88132038
      2.59150928        0.09590135        0.00000000      -29.27540683
      0.48197666       -1.20761772        0.00000000      126.59486448
      0.58781206       -1.20761772        0.00000000      114.43397731
      0.53489436       -1.26053542        0.00000000      114.43433621
      0.53489436       -1.15470002        0.00000000      120.25713638
      1.18265866       -0.00671127        0.00000000     -149.60946451
      1.28849406       -0.00671127        0.00000000     -157.85386272
      1.23557636       -0.05962897        0.00000000       29.56507253
      1.23557636        0.04620643        0.00000000       26.87073240
      0.49575179        1.20505723        0.00000000      180.69741696
      0.60158719        1.20505723        0.00000000      163.62554093
      0.54866949        1.15213953        0.00000000      128.67488578
      0.54866949        1.25797493        0.00000000      122.86467917
     -0.89489563        1.20827070        0.00000000     -183.39273038
     -0.78906023        1.20827070        0.00000000     -199.82699741
     -0.84197793        1.15535300        0.00000000     -115.52322922
     -0.84197793        1.26118840        0.00000000     -104.78518138
     -1.60784569        0.00867526        0.00000000      168.15153672
     -1.50201029        0.00867526        0.00000000      187.45507296
     -1.55492799       -0.04424244        0.00000000      235.60880923
     -1.55492799        0.06159296        0.00000000      234.81516322
```

```
        -0.91316362         -1.19659568          0.00000000     -181.81291430
        -0.80732822         -1.19659568          0.00000000     -195.24548246
        -0.86024592         -1.24951338          0.00000000     -114.95294650
        -0.86024592         -1.14367798          0.00000000     -126.78506453
  35        50932.0    # 196.3 nm,    (r6r1) = 1Ba state
          5.79562640      0.00147495      0.00000000 1.0         # electr. mom.
          0.0                       0.0        -0.5751422283E-02      # magnet. mom.
          2.59150928           0.04298365          0.00000000        213.45391680
          0.53489436          -1.20761772          0.00000000        739.07449968
          1.23557636          -0.00671127          0.00000000       -549.20288266
          0.54866949           1.20505723          0.00000000        681.36019589
        -0.84197793           1.20827070          0.00000000       -726.31340002
        -1.55492799           0.00867526          0.00000000        281.58568114
        -0.86024592          -1.19659568          0.00000000       -632.39787811
          2.53859158           0.04298365          0.00000000       -105.32235994
          2.64442698           0.04298365          0.00000000        -79.49050320
          2.59150928          -0.00993405          0.00000000        -15.80597947
          2.59150928           0.09590135          0.00000000        -15.36451133
          0.48197666          -1.20761772          0.00000000       -292.48022333
          0.58781206          -1.20761772          0.00000000       -255.02304475
          0.53489436          -1.26053542          0.00000000        -93.94156522
          0.53489436          -1.15470002          0.00000000        -99.35106926
          1.18265866          -0.00671127          0.00000000        264.07677082
          1.28849406          -0.00671127          0.00000000        303.98844029
          1.23557636          -0.05962897          0.00000000         -8.66918616
          1.23557636           0.04620643          0.00000000         -6.16743475
          0.49575179           1.20505723          0.00000000       -271.05485486
          0.60158719           1.20505723          0.00000000       -229.72177104
          0.54866949           1.15213953          0.00000000        -93.60494434
          0.54866949           1.25797493          0.00000000        -89.10447394
        -0.89489563           1.20827070          0.00000000        210.34572360
        -0.78906023           1.20827070          0.00000000        243.18556618
        -0.84197793           1.15535300          0.00000000        143.23645426
        -0.84197793           1.26118840          0.00000000        132.11312222
        -1.60784569           0.00867526          0.00000000       -233.59730827
        -1.50201029           0.00867526          0.00000000       -253.94863267
        -1.55492799          -0.04424244          0.00000000        100.99148270
        -1.55492799           0.06159296          0.00000000        102.47223461
        -0.91316362          -1.19659568          0.00000000        157.41553368
        -0.80732822          -1.19659568          0.00000000        183.71561443
        -0.86024592          -1.24951338          0.00000000        140.43734698
        -0.86024592          -1.14367798          0.00000000        153.10944000
  35        51963.0    # 192.4 nm,    (r7r1) = 1Bb state
        -0.02200506    -5.32789864      0.00000000 1.0         # electr. mom.
          0.0                  0.0                 -0.3298715429            # magnet. mom.
          2.59150928           0.04298365          0.00000000          2.65625030
          0.53489436          -1.20761772          0.00000000       1182.00291389
          1.23557636          -0.00671127          0.00000000        -71.65960176
          0.54866949           1.20505723          0.00000000      -1100.14570603
        -0.84197793           1.20827070          0.00000000         -4.15231637
        -1.55492799           0.00867526          0.00000000         -0.77563368
        -0.86024592          -1.19659568          0.00000000        -28.92839026
          2.53859158           0.04298365          0.00000000         -6.07539317
          2.64442698           0.04298365          0.00000000         -4.36380470
          2.59150928          -0.00993405          0.00000000          3.81894864
          2.59150928           0.09590135          0.00000000          3.79502563
          0.48197666          -1.20761772          0.00000000       -434.07759302
          0.58781206          -1.20761772          0.00000000       -379.16869637
          0.53489436          -1.26053542          0.00000000       -179.08625645
          0.53489436          -1.15470002          0.00000000       -193.17141168
          1.18265866          -0.00671127          0.00000000         25.29542242
          1.28849406          -0.00671127          0.00000000         27.15391786
          1.23557636          -0.05962897          0.00000000         17.06864054
          1.23557636           0.04620643          0.00000000          2.45351774
          0.49575179           1.20505723          0.00000000        391.77335458
          0.60158719           1.20505723          0.00000000        341.08733506
          0.54866949           1.15213953          0.00000000        191.53128336
          0.54866949           1.25797493          0.00000000        178.72663325
        -0.89489563           1.20827070          0.00000000        -80.54966813
        -0.78906023           1.20827070          0.00000000       -126.77961840
        -0.84197793           1.15535300          0.00000000        107.70560539
        -0.84197793           1.26118840          0.00000000         99.77245718
        -1.60784569           0.00867526          0.00000000          0.72099302
        -1.50201029           0.00867526          0.00000000          1.63411162
        -1.55492799          -0.04424244          0.00000000         -0.48999677
        -1.55492799           0.06159296          0.00000000         -0.93804053
        -0.91316362          -1.19659568          0.00000000        104.61460656
        -0.80732822          -1.19659568          0.00000000        156.91243762
        -0.86024592          -1.24951338          0.00000000       -108.72151056
        -0.86024592          -1.14367798          0.00000000       -119.63981669
&TRANSITION 2->...
  0      0.0    # (r2r2) 30
     0.0    0.0      0.0 1.0              # electr. mom.
                         0.0    0.0    0.0 # magnet. mom.
  0      0.0    # (r2r2) 30
     0.0    0.0      0.0 1.0              # electr. mom.
                         0.0    0.0    0.0 # magnet. mom.
```

```
0      0.0    # (r2r2) 30
   0.0    0.0      0.0 1.0              # electr. mom.
                    0.0    0.0    0.0 # magnet. mom.
0      0.0    # (r2r2) 30
   0.0    0.0      0.0 1.0              # electr. mom.
                    0.0    0.0    0.0 # magnet. mom.
0      0.0    # (r2r2) 30
   0.0    0.0      0.0 1.0              # electr. mom.
                    0.0    0.0    0.0 # magnet. mom.
0      0.0    # (r2r2) 30
   0.0    0.0      0.0 1.0              # electr. mom.
                    0.0    0.0    0.0 # magnet. mom.
0      0.0    # (r2r2) 30
   0.0    0.0      0.0 1.0              # electr. mom.
                    0.0    0.0    0.0 # magnet. mom.
35        9713.0    # 1029.5 nm,    (r3r2)
        -0.01768          0.135252         0.00000000 1.0          # electr. mom.
         0.0              0.0              -1.983345155             # magnet. mom.
         2.59150928       0.04298365       0.00000000      -4.85360239
         0.53489436      -1.20761772       0.00000000     -11.00452971
         1.23557636      -0.00671127       0.00000000      77.09698564
         0.54866949       1.20505723       0.00000000     -20.09317347
        -0.84197793       1.20827070       0.00000000       9.66162962
        -1.55492799       0.00867526       0.00000000     -13.43125865
        -0.86024592      -1.19659568       0.00000000     -11.50863096
         2.53859158       0.04298365       0.00000000       4.05003468
         2.64442698       0.04298365       0.00000000       2.85195958
         2.59150928      -0.00993405       0.00000000      -0.94218716
         2.59150928       0.09590135       0.00000000      -0.99051102
         0.48197666      -1.20761772       0.00000000      12.24828699
         0.58781206      -1.20761772       0.00000000      12.02012705
         0.53489436      -1.26053542       0.00000000      -5.82234921
         0.53489436      -1.15470002       0.00000000      -7.09408108
         1.18265866      -0.00671127       0.00000000     -13.86051024
         1.28849406      -0.00671127       0.00000000     -14.89464972
         1.23557636      -0.05962897       0.00000000     -26.32939905
         1.23557636       0.04620643       0.00000000     -22.21766644
         0.49575179       1.20505723       0.00000000      11.24017590
         0.60158719       1.20505723       0.00000000       8.40399294
         0.54866949       1.15213953       0.00000000       0.42305151
         0.54866949       1.25797493       0.00000000      -0.00372026
        -0.89489563       1.20827070       0.00000000       8.21694874
        -0.78906023       1.20827070       0.00000000      10.05044362
        -0.84197793       1.15535300       0.00000000     -14.74029810
        -0.84197793       1.26118840       0.00000000     -13.21223370
        -1.60784569       0.00867526       0.00000000       5.44335505
        -1.50201029       0.00867526       0.00000000       5.97103838
        -1.55492799      -0.04424244       0.00000000       1.03368031
        -1.55492799       0.06159296       0.00000000       1.03511316
        -0.91316362      -1.19659568       0.00000000     -11.62328834
        -0.80732822      -1.19659568       0.00000000     -16.12072902
        -0.86024592      -1.24951338       0.00000000      18.56345619
        -0.86024592      -1.14367798       0.00000000      20.43253920
35    14440.0    # 692.5 nm,    (r6r2) 35
        -0.04534          0.108732         0.00000000 1.0          # electr. mom.
         0.0              0.0              0.3558985919             # magnet. mom.
         2.59150928       0.04298365       0.00000000     -18.23989383
         0.53489436      -1.20761772       0.00000000     -89.47644813
         1.23557636      -0.00671127       0.00000000       5.91956085
         0.54866949       1.20505723       0.00000000     134.09401849
        -0.84197793       1.20827070       0.00000000    -145.78611708
        -1.55492799       0.00867526       0.00000000      12.71572084
        -0.86024592      -1.19659568       0.00000000      98.98988402
         2.53859158       0.04298365       0.00000000       7.36736889
         2.64442698       0.04298365       0.00000000       6.24840171
         2.59150928      -0.00993405       0.00000000       2.11100171
         2.59150928       0.09590135       0.00000000       2.56319092
         0.48197666      -1.20761772       0.00000000      -9.89757917
         0.58781206      -1.20761772       0.00000000      -2.00164830
         0.53489436      -1.26053542       0.00000000      48.48890290
         0.53489436      -1.15470002       0.00000000      51.88274889
         1.18265866      -0.00671127       0.00000000      19.79442409
         1.28849406      -0.00671127       0.00000000      18.31380281
         1.23557636      -0.05962897       0.00000000     -19.98165547
         1.23557636       0.04620643       0.00000000     -24.03436933
         0.49575179       1.20505723       0.00000000      -5.63816505
         0.60158719       1.20505723       0.00000000      -8.42603551
         0.54866949       1.15213953       0.00000000     -62.57342777
         0.54866949       1.25797493       0.00000000     -57.18087468
        -0.89489563       1.20827070       0.00000000      48.66119225
        -0.78906023       1.20827070       0.00000000      51.83447259
        -0.84197793       1.15535300       0.00000000      22.41215614
        -0.84197793       1.26118840       0.00000000      22.51281490
        -1.60784569       0.00867526       0.00000000       8.42392831
        -1.50201029       0.00867526       0.00000000      11.44986119
        -1.55492799      -0.04424244       0.00000000     -16.15478140
        -1.55492799       0.06159296       0.00000000     -16.11074303
```

```
  -0.91316362    -1.19659568    0.00000000    9.91211792
  -0.80732822    -1.19659568    0.00000000    20.13056443
  -0.86024592    -1.24951338    0.00000000    -60.06332223
  -0.86024592    -1.14367798    0.00000000    -68.26107281
35   15471.0   # 646.4 nm,   (r7r2) 35
  -0.19729    0.04654    0.00000000 1.0    # electr. mom.
  0.0    0.0    0.2038478162E-01    # magnet. mom.
  2.59150928    0.04298365    0.00000000    -10.57594085
  0.53489436    -1.20761772    0.00000000    110.37822619
  1.23557636    -0.00671127    0.00000000    -58.62712949
  0.54866949    1.20505723    0.00000000    101.68250916
  -0.84197793    1.20827070    0.00000000    91.12932661
  -1.55492799    0.00867526    0.00000000    -133.02099900
  -0.86024592    -1.19659568    0.00000000    81.19281910
  2.53859158    0.04298365    0.00000000    6.70195282
  2.64442698    0.04298365    0.00000000    5.61736863
  2.59150928    -0.00993405    0.00000000    -1.32770197
  2.59150928    0.09590135    0.00000000    -0.50326167
  0.48197666    -1.20761772    0.00000000    -57.66990396
  0.58781206    -1.20761772    0.00000000    -52.96958964
  0.53489436    -1.26053542    0.00000000    -2.10295211
  0.53489436    -1.15470002    0.00000000    2.68688864
  1.18265866    -0.00671127    0.00000000    -8.97651938
  1.28849406    -0.00671127    0.00000000    -2.94341030
  1.23557636    -0.05962897    0.00000000    35.54947002
  1.23557636    0.04620643    0.00000000    34.01338889
  0.49575179    1.20505723    0.00000000    -58.47193480
  0.60158719    1.20505723    0.00000000    -52.93238838
  0.54866949    1.15213953    0.00000000    8.20033848
  0.54866949    1.25797493    0.00000000    1.94620753
  -0.89489563    1.20827070    0.00000000    -32.58403172
  -0.78906023    1.20827070    0.00000000    -32.16005825
  -0.84197793    1.15535300    0.00000000    -12.55852379
  -0.84197793    1.26118840    0.00000000    -13.37429489
  -1.60784569    0.00867526    0.00000000    17.72808249
  -1.50201029    0.00867526    0.00000000    15.32741910
  -1.55492799    -0.04424244    0.00000000    49.64481737
  -1.55492799    0.06159296    0.00000000    49.74115102
  -0.91316362    -1.19659568    0.00000000    -30.47929173
  -0.80732822    -1.19659568    0.00000000    -30.30296039
  -0.86024592    -1.24951338    0.00000000    -10.67265981
  -0.86024592    -1.14367798    0.00000000    -9.28641395
&TRANSITION 3->...
0    0.0   # (r2r2) 30
  0.0   0.0    0.0 1.0    # electr. mom.
    0.0   0.0   0.0 # magnet. mom.
0    0.0   # (r2r2) 30
  0.0   0.0    0.0 1.0    # electr. mom.
    0.0   0.0   0.0 # magnet. mom.
0    0.0   # (r2r2) 30
  0.0   0.0    0.0 1.0    # electr. mom.
    0.0   0.0   0.0 # magnet. mom.
0    0.0   # (r2r2) 30
  0.0   0.0    0.0 1.0    # electr. mom.
    0.0   0.0   0.0 # magnet. mom.
0    0.0   # (r2r2) 30
  0.0   0.0    0.0 1.0    # electr. mom.
    0.0   0.0   0.0 # magnet. mom.
0    0.0   # (r2r2) 30
  0.0   0.0    0.0 1.0    # electr. mom.
    0.0   0.0   0.0 # magnet. mom.
35    9713.0   # 1029.5 nm,   (r3r2)
  -0.01972    0.150858    0.00000000 1.0    # electr. mom.
  0.0    0.0    -1.983345155    # magnet. mom.
  2.59150928    0.04298365    0.00000000    -5.41363344
  0.53489436    -1.20761772    0.00000000    -12.27428314
  1.23557636    -0.00671127    0.00000000    85.99279167
  0.54866949    1.20505723    0.00000000    -22.41161657
  -0.84197793    1.20827070    0.00000000    10.77643304
  -1.55492799    0.00867526    0.00000000    -14.98101926
  -0.86024592    -1.19659568    0.00000000    -12.83654991
  2.53859158    0.04298365    0.00000000    4.51734638
  2.64442698    0.04298365    0.00000000    3.18103184
  2.59150928    -0.00993405    0.00000000    -1.05090106
  2.59150928    0.09590135    0.00000000    -1.10480075
  0.48197666    -1.20761772    0.00000000    13.66155087
  0.58781206    -1.20761772    0.00000000    13.40706479
  0.53489436    -1.26053542    0.00000000    -6.49415874
  0.53489436    -1.15470002    0.00000000    -7.91262890
  1.18265866    -0.00671127    0.00000000    -15.45979989
  1.28849406    -0.00671127    0.00000000    -16.61326315
  1.23557636    -0.05962897    0.00000000    -29.36740663
  1.23557636    0.04620643    0.00000000    -24.78124334
  0.49575179    1.20505723    0.00000000    12.53711927
  0.60158719    1.20505723    0.00000000    9.37368443
  0.54866949    1.15213953    0.00000000    0.47186514
  0.54866949    1.25797493    0.00000000    -0.00414952
```

```
-0.89489563    1.20827070    0.00000000    9.16505821
-0.78906023    1.20827070    0.00000000    11.21011019
-0.84197793    1.15535300    0.00000000    -16.44110173
-0.84197793    1.26118840    0.00000000    -14.73672220
-1.60784569    0.00867526    0.00000000    6.07143448
-1.50201029    0.00867526    0.00000000    6.66000434
-1.55492799    -0.04424244    0.00000000    1.15295112
-1.55492799    0.06159296    0.00000000    1.15454930
-0.91316362    -1.19659568    0.00000000    -12.96443699
-0.80732822    -1.19659568    0.00000000    -17.98081314
-0.86024592    -1.24951338    0.00000000    20.70539345
-0.86024592    -1.14367798    0.00000000    22.79013987
35    14440.0    # 692.5 nm,    (r6r2) 35
-0.05058    0.121278    0.00000000 1.0    # electr. mom.
0.0    0.0    0.3558985919    # magnet. mom.
2.59150928    0.04298365    0.00000000    -20.34449696
0.53489436    -1.20761772    0.00000000    -99.80065368
1.23557636    -0.00671127    0.00000000    6.60258710
0.54866949    1.20505723    0.00000000    149.56640524
-0.84197793    1.20827070    0.00000000    -162.60759213
-1.55492799    0.00867526    0.00000000    14.18291940
-0.86024592    -1.19659568    0.00000000    110.41179371
2.53859158    0.04298365    0.00000000    8.21744992
2.64442698    0.04298365    0.00000000    6.96937114
2.59150928    -0.00993405    0.00000000    2.35457883
2.59150928    0.09590135    0.00000000    2.85894372
0.48197666    -1.20761772    0.00000000    -11.03960754
0.58781206    -1.20761772    0.00000000    -2.23260772
0.53489436    -1.26053542    0.00000000    54.08377631
0.53489436    -1.15470002    0.00000000    57.86921992
1.18265866    -0.00671127    0.00000000    22.07839610
1.28849406    -0.00671127    0.00000000    20.42693390
1.23557636    -0.05962897    0.00000000    -22.28723110
1.23557636    0.04620643    0.00000000    -26.80756579
0.49575179    1.20505723    0.00000000    -6.28872256
0.60158719    1.20505723    0.00000000    -9.39827038
0.54866949    1.15213953    0.00000000    -69.79343867
0.54866949    1.25797493    0.00000000    -63.77866791
-0.89489563    1.20827070    0.00000000    54.27594520
-0.78906023    1.20827070    0.00000000    57.81537327
-0.84197793    1.15535300    0.00000000    24.99817415
-0.84197793    1.26118840    0.00000000    25.11044738
-1.60784569    0.00867526    0.00000000    9.39592004
-1.50201029    0.00867526    0.00000000    12.77099902
-1.55492799    -0.04424244    0.00000000    -18.01879464
-1.55492799    0.06159296    0.00000000    -17.96967492
-0.91316362    -1.19659568    0.00000000    11.05582383
-0.80732822    -1.19659568    0.00000000    22.45332186
-0.86024592    -1.24951338    0.00000000    -66.99370556
-0.86024592    -1.14367798    0.00000000    -76.13735044
35    15471.0    # 646.4 nm,    (r7r2) 35
-0.22005    0.05191    0.00000000 1.0    # electr. mom.
0.0    0.0    0.2038478162E-01    # magnet. mom.
2.59150928    0.04298365    0.00000000    -11.79624172
0.53489436    -1.20761772    0.00000000    123.11417537
1.23557636    -0.00671127    0.00000000    -65.39179828
0.54866949    1.20505723    0.00000000    113.41510637
-0.84197793    1.20827070    0.00000000    101.64424891
-1.55492799    0.00867526    0.00000000    -148.36957581
-0.86024592    -1.19659568    0.00000000    90.56122131
2.53859158    0.04298365    0.00000000    7.47525506
2.64442698    0.04298365    0.00000000    6.26552655
2.59150928    -0.00993405    0.00000000    -1.48089835
2.59150928    0.09590135    0.00000000    -0.56133032
0.48197666    -1.20761772    0.00000000    -64.32412365
0.58781206    -1.20761772    0.00000000    -59.08146537
0.53489436    -1.26053542    0.00000000    -2.34560043
0.53489436    -1.15470002    0.00000000    2.99691425
1.18265866    -0.00671127    0.00000000    -10.01227161
1.28849406    -0.00671127    0.00000000    -3.28303456
1.23557636    -0.05962897    0.00000000    39.65133194
1.23557636    0.04620643    0.00000000    37.93801069
0.49575179    1.20505723    0.00000000    -65.21869651
0.60158719    1.20505723    0.00000000    -59.03997166
0.54866949    1.15213953    0.00000000    9.14653139
0.54866949    1.25797493    0.00000000    2.17076993
-0.89489563    1.20827070    0.00000000    -36.34372769
-0.78906023    1.20827070    0.00000000    -35.87083420
-0.84197793    1.15535300    0.00000000    -14.00758422
-0.84197793    1.26118840    0.00000000    -14.91748276
-1.60784569    0.00867526    0.00000000    19.77363047
-1.50201029    0.00867526    0.00000000    17.09596746
-1.55492799    -0.04424244    0.00000000    55.37306553
-1.55492799    0.06159296    0.00000000    55.48051460
-0.91316362    -1.19659568    0.00000000    -33.99613308
-0.80732822    -1.19659568    0.00000000    -33.79945582
-0.86024592    -1.24951338    0.00000000    -11.90412055
```

```
-0.86024592 -1.14367798 0.00000000 -10.35792325
&TRANSITION 4->...
0 0.0 # (r2r2) 30
0.0 0.0 0.0 1.0 # electr. mom.
0.0 0.0 0.0 # magnet. mom.
0 0.0 # (r2r2) 30
0.0 0.0 0.0 1.0 # electr. mom.
0.0 0.0 0.0 # magnet. mom.
0 0.0 # (r2r2) 30
0.0 0.0 0.0 1.0 # electr. mom.
0.0 0.0 0.0 # magnet. mom.
0 0.0 # (r2r2) 30
0.0 0.0 0.0 1.0 # electr. mom.
0.0 0.0 0.0 # magnet. mom.
0 0.0 # (r2r2) 30
0.0 0.0 0.0 1.0 # electr. mom.
0.0 0.0 0.0 # magnet. mom.
35 9713.0 # 1029.5 nm, (r3r2)
-0.00578 0.044217 0.00000000 1.0 # electr. mom.
0.0 0.0 -1.983345155 # magnet. mom.
2.59150928 0.04298365 0.00000000 -1.58675463
0.53489436 -1.20761772 0.00000000 -3.59763471
1.23557636 -0.00671127 0.00000000 25.20478377
0.54866949 1.20505723 0.00000000 -6.56892210
-0.84197793 1.20827070 0.00000000 3.15860968
-1.55492799 0.00867526 0.00000000 -4.39098840
-0.86024592 -1.19659568 0.00000000 -3.76243704
2.53859158 0.04298365 0.00000000 1.32404980
2.64442698 0.04298365 0.00000000 0.93237140
2.59150928 -0.00993405 0.00000000 -0.30802272
2.59150928 0.09590135 0.00000000 -0.32382091
0.48197666 -1.20761772 0.00000000 4.00424767
0.58781206 -1.20761772 0.00000000 3.92965692
0.53489436 -1.26053542 0.00000000 -1.90346032
0.53489436 -1.15470002 0.00000000 -2.31921882
1.18265866 -0.00671127 0.00000000 -4.53132066
1.28849406 -0.00671127 0.00000000 -4.86940472
1.23557636 -0.05962897 0.00000000 -8.60768815
1.23557636 0.04620643 0.00000000 -7.26346788
0.49575179 1.20505723 0.00000000 3.67467289
0.60158719 1.20505723 0.00000000 2.74745923
0.54866949 1.15213953 0.00000000 0.13830530
0.54866949 1.25797493 0.00000000 -0.00121624
-0.89489563 1.20827070 0.00000000 2.68631017
-0.78906023 1.20827070 0.00000000 3.28572195
-0.84197793 1.15535300 0.00000000 -4.81894361
-0.84197793 1.26118840 0.00000000 -4.31938409
-1.60784569 0.00867526 0.00000000 1.77955838
-1.50201029 0.00867526 0.00000000 1.95207024
-1.55492799 -0.04424244 0.00000000 0.33793395
-1.55492799 0.06159296 0.00000000 0.33840238
-0.91316362 -1.19659568 0.00000000 -3.79992119
-0.80732822 -1.19659568 0.00000000 -5.27023833
-0.86024592 -1.24951338 0.00000000 6.06882222
-0.86024592 -1.14367798 0.00000000 6.67986858
35 14440.0 # 692.5 nm, (r6r2) 35
-0.01482 0.035547 0.00000000 1.0 # electr. mom.
0.0 0.0 0.3558985919 # magnet. mom.
2.59150928 0.04298365 0.00000000 -5.96304221
0.53489436 -1.20761772 0.00000000 -29.25191574
1.23557636 -0.00671127 0.00000000 1.93524105
0.54866949 1.20505723 0.00000000 43.83842912
-0.84197793 1.20827070 0.00000000 -47.66084597
-1.55492799 0.00867526 0.00000000 4.15706258
-0.86024592 -1.19659568 0.00000000 32.36207747
2.53859158 0.04298365 0.00000000 2.40856291
2.64442698 0.04298365 0.00000000 2.04274671
2.59150928 -0.00993405 0.00000000 0.69013517
2.59150928 0.09590135 0.00000000 0.83796626
0.48197666 -1.20761772 0.00000000 -3.23574704
0.58781206 -1.20761772 0.00000000 -0.65438502
0.53489436 -1.26053542 0.00000000 15.85214133
0.53489436 -1.15470002 0.00000000 16.96166791
1.18265866 -0.00671127 0.00000000 6.47125403
1.28849406 -0.00671127 0.00000000 5.98720476
1.23557636 -0.05962897 0.00000000 -6.53246429
1.23557636 0.04620643 0.00000000 -7.85738997
0.49575179 1.20505723 0.00000000 -1.84324627
0.60158719 1.20505723 0.00000000 -2.75466546
0.54866949 1.15213953 0.00000000 -20.45669754
0.54866949 1.25797493 0.00000000 -18.69374749
-0.89489563 1.20827070 0.00000000 15.90846670
-0.78906023 1.20827070 0.00000000 16.94588527
-0.84197793 1.15535300 0.00000000 7.32705104
-0.84197793 1.26118840 0.00000000 7.35995872
-1.60784569 0.00867526 0.00000000 2.75397656
-1.50201029 0.00867526 0.00000000 3.74322385
```

```
-1.55492799 -0.04424244 0.00000000 -5.28137084
-1.55492799 0.06159296 0.00000000 -5.26697368
-0.91316362 -1.19659568 0.00000000 3.24050009
-0.80732822 -1.19659568 0.00000000 6.58114606
-0.86024592 -1.24951338 0.00000000 -19.63608611
-0.86024592 -1.14367798 0.00000000 -22.31611996
35 15471.0 # 646.4 nm, (r7r2) 35
-0.0645 0.015215 0.00000000 1.0 # electr. mom.
0.0 0.0 0.2038478162E-01 # magnet. mom.
2.59150928 0.04298365 0.00000000 -3.45751912
0.53489436 -1.20761772 0.00000000 36.08518933
1.23557636 -0.00671127 0.00000000 -19.16656156
0.54866949 1.20505723 0.00000000 33.24235876
-0.84197793 1.20827070 0.00000000 29.79227985
-1.55492799 0.00867526 0.00000000 -43.48763429
-0.86024592 -1.19659568 0.00000000 26.54380625
2.53859158 0.04298365 0.00000000 2.19102304
2.64442698 0.04298365 0.00000000 1.83644744
2.59150928 -0.00993405 0.00000000 -0.43405641
2.59150928 0.09590135 0.00000000 -0.16452785
0.48197666 -1.20761772 0.00000000 -18.85362245
0.58781206 -1.20761772 0.00000000 -17.31698123
0.53489436 -1.26053542 0.00000000 -0.68750357
0.53489436 -1.15470002 0.00000000 0.87840590
1.18265866 -0.00671127 0.00000000 -2.93463133
1.28849406 -0.00671127 0.00000000 -0.96226875
1.23557636 -0.05962897 0.00000000 11.62194212
1.23557636 0.04620643 0.00000000 11.11976175
0.49575179 1.20505723 0.00000000 -19.11582484
0.60158719 1.20505723 0.00000000 -17.30481928
0.54866949 1.15213953 0.00000000 2.68087989
0.54866949 1.25797493 0.00000000 0.63626015
-0.89489563 1.20827070 0.00000000 -10.65247191
-0.78906023 1.20827070 0.00000000 -10.51386520
-0.84197793 1.15535300 0.00000000 -4.10567124
-0.84197793 1.26118840 0.00000000 -4.37236564
-1.60784569 0.00867526 0.00000000 5.79571927
-1.50201029 0.00867526 0.00000000 5.01088701
-1.55492799 -0.04424244 0.00000000 16.23003645
-1.55492799 0.06159296 0.00000000 16.26153014
-0.91316362 -1.19659568 0.00000000 -9.96438384
-0.80732822 -1.19659568 0.00000000 -9.90673705
-0.86024592 -1.24951338 0.00000000 -3.48913878
-0.86024592 -1.14367798 0.00000000 -3.03594302
&TRANSITION 5->...
0 0.0 # (r2r2) 30
0.0 0.0 0.0 1.0 # electr. mom.
0.0 0.0 0.0 # magnet. mom.
0 0.0 # (r2r2) 30
0.0 0.0 0.0 1.0 # electr. mom.
0.0 0.0 0.0 # magnet. mom.
0 0.0 # (r2r2) 30
0.0 0.0 0.0 1.0 # electr. mom.
0.0 0.0 0.0 # magnet. mom.
0 0.0 # (r2r2) 30
0.0 0.0 0.0 1.0 # electr. mom.
0.0 0.0 0.0 # magnet. mom.
35 9713.0 # 1029.5 nm, (r3r2)
-0.0153 0.117045 0.00000000 1.0 # electr. mom.
0.0 0.0 -1.983345155 # magnet. mom.
2.59150928 0.04298365 0.00000000 -4.20023284
0.53489436 -1.20761772 0.00000000 -9.52315071
1.23557636 -0.00671127 0.00000000 66.71854526
0.54866949 1.20505723 0.00000000 -17.38832320
-0.84197793 1.20827070 0.00000000 8.36102563
-1.55492799 0.00867526 0.00000000 -11.62320460
-0.86024592 -1.19659568 0.00000000 -9.95939217
2.53859158 0.04298365 0.00000000 3.50483771
2.64442698 0.04298365 0.00000000 2.46804194
2.59150928 -0.00993405 0.00000000 -0.81535427
2.59150928 0.09590135 0.00000000 -0.85717300
0.48197666 -1.20761772 0.00000000 10.59947912
0.58781206 -1.20761772 0.00000000 10.40203302
0.53489436 -1.26053542 0.00000000 -5.03857143
0.53489436 -1.15470002 0.00000000 -6.13910863
1.18265866 -0.00671127 0.00000000 -11.99467233
1.28849406 -0.00671127 0.00000000 -12.88960072
1.23557636 -0.05962897 0.00000000 -22.78505687
1.23557636 0.04620643 0.00000000 -19.22682673
0.49575179 1.20505723 0.00000000 9.72707530
0.60158719 1.20505723 0.00000000 7.27268620
0.54866949 1.15213953 0.00000000 0.36610227
0.54866949 1.25797493 0.00000000 -0.00321946
-0.89489563 1.20827070 0.00000000 7.11082103
-0.78906023 1.20827070 0.00000000 8.69749929
-0.84197793 1.15535300 0.00000000 -12.75602720
-0.84197793 1.26118840 0.00000000 -11.43366378
```

```
        -1.60784569      0.00867526      0.00000000       4.71059572
        -1.50201029      0.00867526      0.00000000       5.16724475
        -1.55492799     -0.04424244      0.00000000       0.89453104
        -1.55492799      0.06159296      0.00000000       0.89577101
        -0.91316362     -1.19659568      0.00000000     -10.05861491
        -0.80732822     -1.19659568      0.00000000     -13.95063089
        -0.86024592     -1.24951338      0.00000000      16.06452940
        -0.86024592     -1.14367798      0.00000000      17.68200507
35    14440.0    # 692.5 nm,    (r6r2) 35
        -0.03924         0.094095        0.00000000 1.0        # electr. mom.
         0.0             0.0             0.3558985919           # magnet. mom.
         2.59150928      0.04298365      0.00000000     -15.78452351
         0.53489436     -1.20761772      0.00000000     -77.43154165
         1.23557636     -0.00671127      0.00000000       5.12269689
         0.54866949      1.20505723      0.00000000     116.04290062
        -0.84197793      1.20827070      0.00000000    -126.16106286
        -1.55492799      0.00867526      0.00000000      11.00398919
        -0.86024592     -1.19659568      0.00000000      85.66432271
         2.53859158      0.04298365      0.00000000       6.37560770
         2.64442698      0.04298365      0.00000000       5.40727071
         2.59150928     -0.00993405      0.00000000       1.82682840
         2.59150928      0.09590135      0.00000000       2.21814599
         0.48197666     -1.20761772      0.00000000      -8.56521275
         0.58781206     -1.20761772      0.00000000      -1.73219565
         0.53489436     -1.26053542      0.00000000      41.96155059
         0.53489436     -1.15470002      0.00000000      44.89853270
         1.18265866     -0.00671127      0.00000000      17.12979008
         1.28849406     -0.00671127      0.00000000      15.84848320
         1.23557636     -0.05962897      0.00000000     -17.29181723
         1.23557636      0.04620643      0.00000000     -20.79897346
         0.49575179      1.20505723      0.00000000      -4.87918130
         0.60158719      1.20505723      0.00000000      -7.29176150
         0.54866949      1.15213953      0.00000000     -54.15008172
         0.54866949      1.25797493      0.00000000     -49.48344924
        -0.89489563      1.20827070      0.00000000      42.11064714
        -0.78906023      1.20827070      0.00000000      44.85675512
        -0.84197793      1.15535300      0.00000000      19.39513512
        -0.84197793      1.26118840      0.00000000      19.48224366
        -1.60784569      0.00867526      0.00000000       7.28993796
        -1.50201029      0.00867526      0.00000000       9.90853372
        -1.55492799     -0.04424244      0.00000000     -13.98009929
        -1.55492799      0.06159296      0.00000000     -13.94198916
        -0.91316362     -1.19659568      0.00000000       8.57779435
        -0.80732822     -1.19659568      0.00000000      17.42068076
        -0.86024592     -1.24951338      0.00000000     -51.97787501
        -0.86024592     -1.14367798      0.00000000     -59.07208224
35    15471.0    # 646.4 nm,    (r7r2) 35
        -0.17073         0.040275        0.00000000 1.0        # electr. mom.
         0.0             0.0             0.2038478162E-01       # magnet. mom.
         2.59150928      0.04298365      0.00000000      -9.15225651
         0.53489436     -1.20761772      0.00000000      95.51961882
         1.23557636     -0.00671127      0.00000000     -50.73501591
         0.54866949      1.20505723      0.00000000      87.99447908
        -0.84197793      1.20827070      0.00000000      78.86191726
        -1.55492799      0.00867526      0.00000000    -115.11432606
        -0.86024592     -1.19659568      0.00000000      70.26301653
         2.53859158      0.04298365      0.00000000       5.79976686
         2.64442698      0.04298365      0.00000000       4.86118439
         2.59150928     -0.00993405      0.00000000      -1.14897286
         2.59150928      0.09590135      0.00000000      -0.43551491
         0.48197666     -1.20761772      0.00000000     -49.90664766
         0.58781206     -1.20761772      0.00000000     -45.83906796
         0.53489436     -1.26053542      0.00000000      -1.81986240
         0.53489436     -1.15470002      0.00000000       2.32519209
         1.18265866     -0.00671127      0.00000000      -7.76814177
         1.28849406     -0.00671127      0.00000000      -2.54718199
         1.23557636     -0.05962897      0.00000000      30.76396444
         1.23557636      0.04620643      0.00000000      29.43466346
         0.49575179      1.20505723      0.00000000     -50.60071281
         0.60158719      1.20505723      0.00000000     -45.80687456
         0.54866949      1.15213953      0.00000000       7.09644677
         0.54866949      1.25797493      0.00000000       1.68421805
        -0.89489563      1.20827070      0.00000000     -28.19771976
        -0.78906023      1.20827070      0.00000000     -27.83081964
        -0.84197793      1.15535300      0.00000000     -10.86795328
        -0.84197793      1.26118840      0.00000000     -11.57390904
        -1.60784569      0.00867526      0.00000000      15.34160984
        -1.50201029      0.00867526      0.00000000      13.26411269
        -1.55492799     -0.04424244      0.00000000      42.96186119
        -1.55492799      0.06159296      0.00000000      43.04522684
        -0.91316362     -1.19659568      0.00000000     -26.37631015
        -0.80732822     -1.19659568      0.00000000     -26.22371572
        -0.86024592     -1.24951338      0.00000000      -9.23595560
        -0.86024592     -1.14367798      0.00000000      -8.03631977
&TRANSITION 6->...
  0      0.0    # (r2r2) 30
     0.0    0.0      0.0 1.0              # electr. mom.
```

```
0.0 0.0 0.0 # magnet. mom.
0 0.0 # (r2r2) 30
0.0 0.0 0.0 1.0 # electr. mom.
0.0 0.0 0.0 # magnet. mom.
0 0.0 # (r2r2) 30
0.0 0.0 0.0 1.0 # electr. mom.
0.0 0.0 0.0 # magnet. mom.
35 9713.0 # 1029.5 nm, (r3r2)
-0.00612 0.046818 0.00000000 1.0 # electr. mom.
0.0 0.0 -1.983345155 # magnet. mom.
2.59150928 0.04298365 0.00000000 -1.68009314
0.53489436 -1.20761772 0.00000000 -3.80926029
1.23557636 -0.00671127 0.00000000 26.68741811
0.54866949 1.20505723 0.00000000 -6.95532928
-0.84197793 1.20827070 0.00000000 3.34441025
-1.55492799 0.00867526 0.00000000 -4.64928184
-0.86024592 -1.19659568 0.00000000 -3.98375687
2.53859158 0.04298365 0.00000000 1.40193508
2.64442698 0.04298365 0.00000000 0.98721678
2.59150928 -0.00993405 0.00000000 -0.32614171
2.59150928 0.09590135 0.00000000 -0.34286920
0.48197666 -1.20761772 0.00000000 4.23979165
0.58781206 -1.20761772 0.00000000 4.16081321
0.53489436 -1.26053542 0.00000000 -2.01542857
0.53489436 -1.15470002 0.00000000 -2.45564345
1.18265866 -0.00671127 0.00000000 -4.79786893
1.28849406 -0.00671127 0.00000000 -5.15584029
1.23557636 -0.05962897 0.00000000 -9.11402275
1.23557636 0.04620643 0.00000000 -7.69073069
0.49575179 1.20505723 0.00000000 3.89083012
0.60158719 1.20505723 0.00000000 2.90907448
0.54866949 1.15213953 0.00000000 0.14644091
0.54866949 1.25797493 0.00000000 -0.00128778
-0.89489563 1.20827070 0.00000000 2.84432841
-0.78906023 1.20827070 0.00000000 3.47899971
-0.84197793 1.15535300 0.00000000 -5.10241088
-0.84197793 1.26118840 0.00000000 -4.57346551
-1.60784569 0.00867526 0.00000000 1.88423829
-1.50201029 0.00867526 0.00000000 2.06689790
-1.55492799 -0.04424244 0.00000000 0.35781242
-1.55492799 0.06159296 0.00000000 0.35830840
-0.91316362 -1.19659568 0.00000000 -4.02344596
-0.80732822 -1.19659568 0.00000000 -5.58025235
-0.86024592 -1.24951338 0.00000000 6.42581176
-0.86024592 -1.14367798 0.00000000 7.07280203
35 14440.0 # 692.5 nm, (r6r2) 35
-0.0157 0.037638 0.00000000 1.0 # electr. mom.
0.0 0.0 0.3558985919 # magnet. mom.
2.59150928 0.04298365 0.00000000 -6.31380940
0.53489436 -1.20761772 0.00000000 -30.97261666
1.23557636 -0.00671127 0.00000000 2.04907875
0.54866949 1.20505723 0.00000000 46.41716025
-0.84197793 1.20827070 0.00000000 -50.46442514
-1.55492799 0.00867526 0.00000000 4.40159568
-0.86024592 -1.19659568 0.00000000 34.26572908
2.53859158 0.04298365 0.00000000 2.55024308
2.64442698 0.04298365 0.00000000 2.16290829
2.59150928 -0.00993405 0.00000000 0.73073136
2.59150928 0.09590135 0.00000000 0.88725840
0.48197666 -1.20761772 0.00000000 -3.42608510
0.58781206 -1.20761772 0.00000000 -0.69287826
0.53489436 -1.26053542 0.00000000 16.78462023
0.53489436 -1.15470002 0.00000000 17.95941308
1.18265866 -0.00671127 0.00000000 6.85191603
1.28849406 -0.00671127 0.00000000 6.33939328
1.23557636 -0.05962897 0.00000000 -6.91672689
1.23557636 0.04620643 0.00000000 -8.31958938
0.49575179 1.20505723 0.00000000 -1.95167252
0.60158719 1.20505723 0.00000000 -2.91670460
0.54866949 1.15213953 0.00000000 -21.66003269
0.54866949 1.25797493 0.00000000 -19.79337970
-0.89489563 1.20827070 0.00000000 16.84425885
-0.78906023 1.20827070 0.00000000 17.94270205
-0.84197793 1.15535300 0.00000000 7.75805405
-0.84197793 1.26118840 0.00000000 7.79289746
-1.60784569 0.00867526 0.00000000 2.91597519
-1.50201029 0.00867526 0.00000000 3.96341349
-1.55492799 -0.04424244 0.00000000 -5.59203972
-1.55492799 0.06159296 0.00000000 -5.57679566
-0.91316362 -1.19659568 0.00000000 3.43111774
-0.80732822 -1.19659568 0.00000000 6.96827230
-0.86024592 -1.24951338 0.00000000 -20.79115000
-0.86024592 -1.14367798 0.00000000 -23.62883289
35 15471.0 # 646.4 nm, (r7r2) 35
-0.06829 0.01611 0.00000000 1.0 # electr. mom.
0.0 0.0 0.2038478162E-01 # magnet. mom.
2.59150928 0.04298365 0.00000000 -3.66090260
```

```
0.53489436 -1.20761772 0.00000000 38.20784753
1.23557636 -0.00671127 0.00000000 -20.29400636
0.54866949 1.20505723 0.00000000 35.19779163
-0.84197793 1.20827070 0.00000000 31.54476690
-1.55492799 0.00867526 0.00000000 -46.04573042
-0.86024592 -1.19659568 0.00000000 28.10520661
2.53859158 0.04298365 0.00000000 2.31990674
2.64442698 0.04298365 0.00000000 1.94447376
2.59150928 -0.00993405 0.00000000 -0.45958914
2.59150928 0.09590135 0.00000000 -0.17420596
0.48197666 -1.20761772 0.00000000 -19.96265906
0.58781206 -1.20761772 0.00000000 -18.33562718
0.53489436 -1.26053542 0.00000000 -0.72794496
0.53489436 -1.15470002 0.00000000 0.93007684
1.18265866 -0.00671127 0.00000000 -3.10725671
1.28849406 -0.00671127 0.00000000 -1.01887280
1.23557636 -0.05962897 0.00000000 12.30558578
1.23557636 0.04620643 0.00000000 11.77386539
0.49575179 1.20505723 0.00000000 -20.24028512
0.60158719 1.20505723 0.00000000 -18.32274982
0.54866949 1.15213953 0.00000000 2.83857871
0.54866949 1.25797493 0.00000000 0.67368722
-0.89489563 1.20827070 0.00000000 -11.27908790
-0.78906023 1.20827070 0.00000000 -11.13232785
-0.84197793 1.15535300 0.00000000 -4.34718131
-0.84197793 1.26118840 0.00000000 -4.62956361
-1.60784569 0.00867526 0.00000000 6.13664394
-1.50201029 0.00867526 0.00000000 5.30564507
-1.55492799 -0.04424244 0.00000000 17.18474448
-1.55492799 0.06159296 0.00000000 17.21809074
-0.91316362 -1.19659568 0.00000000 -10.55052406
-0.80732822 -1.19659568 0.00000000 -10.48948629
-0.86024592 -1.24951338 0.00000000 -3.69438224
-0.86024592 -1.14367798 0.00000000 -3.21452791
&TRANSITION 7->...
0 0.0 # (r2r2) 30
0.0 0.0 0.0 1.0 # electr. mom.
0.0 0.0 0.0 # magnet. mom.
0 0.0 # (r2r2) 30
0.0 0.0 0.0 1.0 # electr. mom.
0.0 0.0 0.0 # magnet. mom.
35 9713.0 # 1029.5 nm, (r3r2)
-0.00952 0.072828 0.00000000 1.0 # electr. mom.
0.0 0.0 -1.983345155 # magnet. mom.
2.59150928 0.04298365 0.00000000 -2.61347821
0.53489436 -1.20761772 0.00000000 -5.92551600
1.23557636 -0.00671127 0.00000000 41.51376150
0.54866949 1.20505723 0.00000000 -10.81940110
-0.84197793 1.20827070 0.00000000 5.20241595
-1.55492799 0.00867526 0.00000000 -7.23221619
-0.86024592 -1.19659568 0.00000000 -6.19695513
2.53859158 0.04298365 0.00000000 2.18078791
2.64442698 0.04298365 0.00000000 1.53567054
2.59150928 -0.00993405 0.00000000 -0.50733155
2.59150928 0.09590135 0.00000000 -0.53335209
0.48197666 -1.20761772 0.00000000 6.59523145
0.58781206 -1.20761772 0.00000000 6.47237610
0.53489436 -1.26053542 0.00000000 -3.13511111
0.53489436 -1.15470002 0.00000000 -3.81988981
1.18265866 -0.00671127 0.00000000 -7.46335167
1.28849406 -0.00671127 0.00000000 -8.02019600
1.23557636 -0.05962897 0.00000000 -14.17736872
1.23557636 0.04620643 0.00000000 -11.96335885
0.49575179 1.20505723 0.00000000 6.05240241
0.60158719 1.20505723 0.00000000 4.52522697
0.54866949 1.15213953 0.00000000 0.22779697
0.54866949 1.25797493 0.00000000 -0.00200322
-0.89489563 1.20827070 0.00000000 4.42451086
-0.78906023 1.20827070 0.00000000 5.41177733
-0.84197793 1.15535300 0.00000000 -7.93708359
-0.84197793 1.26118840 0.00000000 -7.11427968
-1.60784569 0.00867526 0.00000000 2.93103733
-1.50201029 0.00867526 0.00000000 3.21517451
-1.55492799 -0.04424244 0.00000000 0.55659709
-1.55492799 0.06159296 0.00000000 0.55736863
-0.91316362 -1.19659568 0.00000000 -6.25869372
-0.80732822 -1.19659568 0.00000000 -8.68039255
-0.86024592 -1.24951338 0.00000000 9.99570718
-0.86024592 -1.14367798 0.00000000 11.00213649
35 14440.0 # 692.5 nm, (r6r2) 35
-0.02442 0.058548 0.00000000 1.0 # electr. mom.
0.0 0.0 0.3558985919 # magnet. mom.
2.59150928 0.04298365 0.00000000 -9.82148129
0.53489436 -1.20761772 0.00000000 -48.17962592
1.23557636 -0.00671127 0.00000000 3.18745584
0.54866949 1.20505723 0.00000000 72.20447149
-0.84197793 1.20827070 0.00000000 -78.50021689
```

```
      -1.55492799       0.00867526       0.00000000       6.84692661
      -0.86024592      -1.19659568       0.00000000      53.30224524
       2.53859158       0.04298365       0.00000000       3.96704479
       2.64442698       0.04298365       0.00000000       3.36452400
       2.59150928      -0.00993405       0.00000000       1.13669323
       2.59150928       0.09590135       0.00000000       1.38017973
       0.48197666      -1.20761772       0.00000000      -5.32946571
       0.58781206      -1.20761772       0.00000000      -1.07781062
       0.53489436      -1.26053542       0.00000000      26.10940925
       0.53489436      -1.15470002       0.00000000      27.93686479
       1.18265866      -0.00671127       0.00000000      10.65853605
       1.28849406      -0.00671127       0.00000000       9.86127843
       1.23557636      -0.05962897       0.00000000     -10.75935294
       1.23557636       0.04620643       0.00000000     -12.94158349
       0.49575179       1.20505723       0.00000000      -3.03593503
       0.60158719       1.20505723       0.00000000      -4.53709605
       0.54866949       1.15213953       0.00000000     -33.69338418
       0.54866949       1.25797493       0.00000000     -30.78970175
      -0.89489563       1.20827070       0.00000000      26.20218044
      -0.78906023       1.20827070       0.00000000      27.91086985
      -0.84197793       1.15535300       0.00000000      12.06808407
      -0.84197793       1.26118840       0.00000000      12.12228494
      -1.60784569       0.00867526       0.00000000       4.53596140
      -1.50201029       0.00867526       0.00000000       6.16530987
      -1.55492799      -0.04424244       0.00000000      -8.69872845
      -1.55492799       0.06159296       0.00000000      -8.67501548
      -0.91316362      -1.19659568       0.00000000       5.33729426
      -0.80732822      -1.19659568       0.00000000      10.83953469
      -0.86024592      -1.24951338       0.00000000     -32.34178889
      -0.86024592      -1.14367798       0.00000000     -36.75596228
 35    15471.0    # 646.4 nm,    (r7r2) 35
      -0.10623          0.02506          0.00000000 1.0        # electr. mom.
       0.0              0.0              0.2038478162E-01      # magnet. mom.
       2.59150928       0.04298365       0.00000000      -5.69473738
       0.53489436      -1.20761772       0.00000000      59.43442949
       1.23557636      -0.00671127       0.00000000     -31.56845434
       0.54866949       1.20505723       0.00000000      54.75212032
      -0.84197793       1.20827070       0.00000000      49.06963741
      -1.55492799       0.00867526       0.00000000     -71.62669177
      -0.86024592      -1.19659568       0.00000000      43.71921029
       2.53859158       0.04298365       0.00000000       3.60874382
       2.64442698       0.04298365       0.00000000       3.02473695
       2.59150928      -0.00993405       0.00000000      -0.71491645
       2.59150928       0.09590135       0.00000000      -0.27098705
       0.48197666      -1.20761772       0.00000000     -31.05302521
       0.58781206      -1.20761772       0.00000000     -28.52208673
       0.53489436      -1.26053542       0.00000000      -1.13235883
       0.53489436      -1.15470002       0.00000000       1.44678619
       1.18265866      -0.00671127       0.00000000      -4.83351043
       1.28849406      -0.00671127       0.00000000      -1.58491324
       1.23557636      -0.05962897       0.00000000      19.14202232
       1.23557636       0.04620643       0.00000000      18.31490171
       0.49575179       1.20505723       0.00000000     -31.48488797
       0.60158719       1.20505723       0.00000000     -28.50205528
       0.54866949       1.15213953       0.00000000       4.41556688
       0.54866949       1.25797493       0.00000000       1.04795790
      -0.89489563       1.20827070       0.00000000     -17.54524785
      -0.78906023       1.20827070       0.00000000     -17.31695444
      -0.84197793       1.15535300       0.00000000      -6.76228204
      -0.84197793       1.26118840       0.00000000      -7.20154340
      -1.60784569       0.00867526       0.00000000       9.54589057
      -1.50201029       0.00867526       0.00000000       8.25322567
      -1.55492799      -0.04424244       0.00000000      26.73182474
      -1.55492799       0.06159296       0.00000000      26.78369670
      -0.91316362      -1.19659568       0.00000000     -16.41192632
      -0.80732822      -1.19659568       0.00000000     -16.31697867
      -0.86024592      -1.24951338       0.00000000      -5.74681682
      -0.86024592      -1.14367798       0.00000000      -5.00037674
&TRANSITION 8->...
 0      0.0    # (r2r2) 30
    0.0    0.0      0.0 1.0             # electr. mom.
                      0.0    0.0    0.0 # magnet. mom.
 35       9713.0    # 1029.5 nm,    (r3r2)
      -0.00476          0.036414         0.00000000 1.0        # electr. mom.
       0.0              0.0             -1.983345155           # magnet. mom.
       2.59150928       0.04298365       0.00000000      -1.30673911
       0.53489436      -1.20761772       0.00000000      -2.96275800
       1.23557636      -0.00671127       0.00000000      20.75688075
       0.54866949       1.20505723       0.00000000      -5.40970055
      -0.84197793       1.20827070       0.00000000       2.60120797
      -1.55492799       0.00867526       0.00000000      -3.61610810
      -0.86024592      -1.19659568       0.00000000      -3.09847757
       2.53859158       0.04298365       0.00000000       1.09039395
       2.64442698       0.04298365       0.00000000       0.76783527
       2.59150928      -0.00993405       0.00000000      -0.25366577
       2.59150928       0.09590135       0.00000000      -0.26667604
       0.48197666      -1.20761772       0.00000000       3.29761573
```

```
      0.58781206     -1.20761772      0.00000000      3.23618805
      0.53489436     -1.26053542      0.00000000     -1.56755556
      0.53489436     -1.15470002      0.00000000     -1.90994491
      1.18265866     -0.00671127      0.00000000     -3.73167583
      1.28849406     -0.00671127      0.00000000     -4.01009800
      1.23557636     -0.05962897      0.00000000     -7.08868436
      1.23557636      0.04620643      0.00000000     -5.98167943
      0.49575179      1.20505723      0.00000000      3.02620120
      0.60158719      1.20505723      0.00000000      2.26261348
      0.54866949      1.15213953      0.00000000      0.11389848
      0.54866949      1.25797493      0.00000000     -0.00100161
     -0.89489563      1.20827070      0.00000000      2.21225543
     -0.78906023      1.20827070      0.00000000      2.70588867
     -0.84197793      1.15535300      0.00000000     -3.96854180
     -0.84197793      1.26118840      0.00000000     -3.55713984
     -1.60784569      0.00867526      0.00000000      1.46551867
     -1.50201029      0.00867526      0.00000000      1.60758726
     -1.55492799     -0.04424244      0.00000000      0.27829855
     -1.55492799      0.06159296      0.00000000      0.27868431
     -0.91316362     -1.19659568      0.00000000     -3.12934686
     -0.80732822     -1.19659568      0.00000000     -4.34019628
     -0.86024592     -1.24951338      0.00000000      4.99785359
     -0.86024592     -1.14367798      0.00000000      5.50106825
35    14440.0    # 692.5 nm,    (r6r2) 35
     -0.01221        0.029274        0.00000000 1.0       # electr. mom.
      0.0            0.0             0.3558985919          # magnet. mom.
      2.59150928      0.04298365      0.00000000     -4.91074065
      0.53489436     -1.20761772      0.00000000    -24.08981296
      1.23557636     -0.00671127      0.00000000      1.59372792
      0.54866949      1.20505723      0.00000000     36.10223575
     -0.84197793      1.20827070      0.00000000    -39.25010844
     -1.55492799      0.00867526      0.00000000      3.42346330
     -0.86024592     -1.19659568      0.00000000     26.65112262
      2.53859158      0.04298365      0.00000000      1.98352239
      2.64442698      0.04298365      0.00000000      1.68226200
      2.59150928     -0.00993405      0.00000000      0.56834661
      2.59150928      0.09590135      0.00000000      0.69008986
      0.48197666     -1.20761772      0.00000000     -2.66473285
      0.58781206     -1.20761772      0.00000000     -0.53890531
      0.53489436     -1.26053542      0.00000000     13.05470463
      0.53489436     -1.15470002      0.00000000     13.96843239
      1.18265866     -0.00671127      0.00000000      5.32926803
      1.28849406     -0.00671127      0.00000000      4.93063922
      1.23557636     -0.05962897      0.00000000     -5.37967647
      1.23557636      0.04620643      0.00000000     -6.47079174
      0.49575179      1.20505723      0.00000000     -1.51796751
      0.60158719      1.20505723      0.00000000     -2.26854802
      0.54866949      1.15213953      0.00000000    -16.84669209
      0.54866949      1.25797493      0.00000000    -15.39485088
     -0.89489563      1.20827070      0.00000000     13.10109022
     -0.78906023      1.20827070      0.00000000     13.95543493
     -0.84197793      1.15535300      0.00000000      6.03404204
     -0.84197793      1.26118840      0.00000000      6.06114247
     -1.60784569      0.00867526      0.00000000      2.26798070
     -1.50201029      0.00867526      0.00000000      3.08265493
     -1.55492799     -0.04424244      0.00000000     -4.34936422
     -1.55492799      0.06159296      0.00000000     -4.33750774
     -0.91316362     -1.19659568      0.00000000      2.66864713
     -0.80732822     -1.19659568      0.00000000      5.41976735
     -0.86024592     -1.24951338      0.00000000    -16.17089445
     -0.86024592     -1.14367798      0.00000000    -18.37798114
35    15471.0    # 646.4 nm,    (r7r2) 35
     -0.05312        0.01253         0.00000000 1.0       # electr. mom.
      0.0            0.0             0.2038478162E-01      # magnet. mom.
      2.59150928      0.04298365      0.00000000     -2.84736869
      0.53489436     -1.20761772      0.00000000     29.71721474
      1.23557636     -0.00671127      0.00000000    -15.78422717
      0.54866949      1.20505723      0.00000000     27.37606016
     -0.84197793      1.20827070      0.00000000     24.53481870
     -1.55492799      0.00867526      0.00000000    -35.81334588
     -0.86024592     -1.19659568      0.00000000     21.85960514
      2.53859158      0.04298365      0.00000000      1.80437191
      2.64442698      0.04298365      0.00000000      1.51236848
      2.59150928     -0.00993405      0.00000000     -0.35745822
      2.59150928      0.09590135      0.00000000     -0.13549353
      0.48197666     -1.20761772      0.00000000    -15.52651260
      0.58781206     -1.20761772      0.00000000    -14.26104336
      0.53489436     -1.26053542      0.00000000     -0.56617941
      0.53489436     -1.15470002      0.00000000      0.72339310
      1.18265866     -0.00671127      0.00000000     -2.41675522
      1.28849406     -0.00671127      0.00000000     -0.79245662
      1.23557636     -0.05962897      0.00000000      9.57101116
      1.23557636      0.04620643      0.00000000      9.15745086
      0.49575179      1.20505723      0.00000000    -15.74244398
      0.60158719      1.20505723      0.00000000    -14.25102764
      0.54866949      1.15213953      0.00000000      2.20778344
      0.54866949      1.25797493      0.00000000      0.52397895
```

```
        -0.89489563      1.20827070      0.00000000      -8.77262393
        -0.78906023      1.20827070      0.00000000      -8.65847722
        -0.84197793      1.15535300      0.00000000      -3.38114102
        -0.84197793      1.26118840      0.00000000      -3.60077170
        -1.60784569      0.00867526      0.00000000       4.77294528
        -1.50201029      0.00867526      0.00000000       4.12661284
        -1.55492799     -0.04424244      0.00000000      13.36591237
        -1.55492799      0.06159296      0.00000000      13.39184835
        -0.91316362     -1.19659568      0.00000000      -8.20596316
        -0.80732822     -1.19659568      0.00000000      -8.15848933
        -0.86024592     -1.24951338      0.00000000      -2.87340841
        -0.86024592     -1.14367798      0.00000000      -2.50018837
&TRANSITION 9->...
 35      9713.0    # 1029.5 nm,    (r3r2)
        -0.00476         0.036414        0.00000000 1.0       # electr. mom.
         0.0             0.0            -1.983345155          # magnet. mom.
         2.59150928      0.04298365      0.00000000      -1.30673911
         0.53489436     -1.20761772      0.00000000      -2.96275800
         1.23557636     -0.00671127      0.00000000      20.75688075
         0.54866949      1.20505723      0.00000000      -5.40970055
        -0.84197793      1.20827070      0.00000000       2.60120797
        -1.55492799      0.00867526      0.00000000      -3.61610810
        -0.86024592     -1.19659568      0.00000000      -3.09847757
         2.53859158      0.04298365      0.00000000       1.09039395
         2.64442698      0.04298365      0.00000000       0.76783527
         2.59150928     -0.00993405      0.00000000      -0.25366577
         2.59150928      0.09590135      0.00000000      -0.26667604
         0.48197666     -1.20761772      0.00000000       3.29761573
         0.58781206     -1.20761772      0.00000000       3.23618805
         0.53489436     -1.26053542      0.00000000      -1.56755556
         0.53489436     -1.15470002      0.00000000      -1.90994491
         1.18265866     -0.00671127      0.00000000      -3.73167583
         1.28849406     -0.00671127      0.00000000      -4.01009800
         1.23557636     -0.05962897      0.00000000      -7.08868436
         1.23557636      0.04620643      0.00000000      -5.98167943
         0.49575179      1.20505723      0.00000000       3.02620120
         0.60158719      1.20505723      0.00000000       2.26261348
         0.54866949      1.15213953      0.00000000       0.11389848
         0.54866949      1.25797493      0.00000000      -0.00100161
        -0.89489563      1.20827070      0.00000000       2.21225543
        -0.78906023      1.20827070      0.00000000       2.70588867
        -0.84197793      1.15535300      0.00000000      -3.96854180
        -0.84197793      1.26118840      0.00000000      -3.55713984
        -1.60784569      0.00867526      0.00000000       1.46551867
        -1.50201029      0.00867526      0.00000000       1.60758726
        -1.55492799     -0.04424244      0.00000000       0.27829855
        -1.55492799      0.06159296      0.00000000       0.27868431
        -0.91316362     -1.19659568      0.00000000      -3.12934686
        -0.80732822     -1.19659568      0.00000000      -4.34019628
        -0.86024592     -1.24951338      0.00000000       4.99785359
        -0.86024592     -1.14367798      0.00000000       5.50106825
 35    14440.0    # 692.5 nm,    (r6r2) 35
        -0.01221         0.029274        0.00000000 1.0       # electr. mom.
         0.0             0.0             0.3558985919         # magnet. mom.
         2.59150928      0.04298365      0.00000000      -4.91074065
         0.53489436     -1.20761772      0.00000000     -24.08981296
         1.23557636     -0.00671127      0.00000000       1.59372792
         0.54866949      1.20505723      0.00000000      36.10223575
        -0.84197793      1.20827070      0.00000000     -39.25010844
        -1.55492799      0.00867526      0.00000000       3.42346330
        -0.86024592     -1.19659568      0.00000000      26.65112262
         2.53859158      0.04298365      0.00000000       1.98352239
         2.64442698      0.04298365      0.00000000       1.68226200
         2.59150928     -0.00993405      0.00000000       0.56834661
         2.59150928      0.09590135      0.00000000       0.69008986
         0.48197666     -1.20761772      0.00000000      -2.66473285
         0.58781206     -1.20761772      0.00000000      -0.53890531
         0.53489436     -1.26053542      0.00000000      13.05470463
         0.53489436     -1.15470002      0.00000000      13.96843239
         1.18265866     -0.00671127      0.00000000       5.32926803
         1.28849406     -0.00671127      0.00000000       4.93063922
         1.23557636     -0.05962897      0.00000000      -5.37967647
         1.23557636      0.04620643      0.00000000      -6.47079174
         0.49575179      1.20505723      0.00000000      -1.51796751
         0.60158719      1.20505723      0.00000000      -2.26854802
         0.54866949      1.15213953      0.00000000     -16.84669209
         0.54866949      1.25797493      0.00000000     -15.39485088
        -0.89489563      1.20827070      0.00000000      13.10109022
        -0.78906023      1.20827070      0.00000000      13.95543493
        -0.84197793      1.15535300      0.00000000       6.03404204
        -0.84197793      1.26118840      0.00000000       6.06114247
        -1.60784569      0.00867526      0.00000000       2.26798070
        -1.50201029      0.00867526      0.00000000       3.08265493
        -1.55492799     -0.04424244      0.00000000      -4.34936422
        -1.55492799      0.06159296      0.00000000      -4.33750774
        -0.91316362     -1.19659568      0.00000000       2.66864713
        -0.80732822     -1.19659568      0.00000000       5.41976735
```

-0.86024592 -1.24951338 0.00000000 -16.17089445
-0.86024592 -1.14367798 0.00000000 -18.37798114
35 15471.0 # 646.4 nm, (r7r2) 35
-0.05312 0.01253 0.00000000 1.0 # electr. mom.
0.0 0.0 0.2038478162E-01 # magnet. mom.
2.59150928 0.04298365 0.00000000 -2.84736869
0.53489436 -1.20761772 0.00000000 29.71721474
1.23557636 -0.00671127 0.00000000 -15.78422717
0.54866949 1.20505723 0.00000000 27.37606016
-0.84197793 1.20827070 0.00000000 24.53481870
-1.55492799 0.00867526 0.00000000 -35.81334588
-0.86024592 -1.19659568 0.00000000 21.85960514
2.53859158 0.04298365 0.00000000 1.80437191
2.64442698 0.04298365 0.00000000 1.51236848
2.59150928 -0.00993405 0.00000000 -0.35745822
2.59150928 0.09590135 0.00000000 -0.13549353
0.48197666 -1.20761772 0.00000000 -15.52651260
0.58781206 -1.20761772 0.00000000 -14.26104336
0.53489436 -1.26053542 0.00000000 -0.56617941
0.53489436 -1.15470002 0.00000000 0.72339310
1.18265866 -0.00671127 0.00000000 -2.41675522
1.28849406 -0.00671127 0.00000000 -0.79245662
1.23557636 -0.05962897 0.00000000 9.57101116
1.23557636 0.04620643 0.00000000 9.15745086
0.49575179 1.20505723 0.00000000 -15.74244398
0.60158719 1.20505723 0.00000000 -14.25102764
0.54866949 1.15213953 0.00000000 2.20778344
0.54866949 1.25797493 0.00000000 0.52397895
-0.89489563 1.20827070 0.00000000 -8.77262393
-0.78906023 1.20827070 0.00000000 -8.65847722
-0.84197793 1.15535300 0.00000000 -3.38114102
-0.84197793 1.26118840 0.00000000 -3.60077170
-1.60784569 0.00867526 0.00000000 4.77294528
-1.50201029 0.00867526 0.00000000 4.12661284
-1.55492799 -0.04424244 0.00000000 13.36591237
-1.55492799 0.06159296 0.00000000 13.39184835
-0.91316362 -1.19659568 0.00000000 -8.20596316
-0.80732822 -1.19659568 0.00000000 -8.15848933
-0.86024592 -1.24951338 0.00000000 -2.87340841
-0.86024592 -1.14367798 0.00000000 -2.50018837
&TRANSITION 10->...
35 4727.0 # 2115.5 nm, (r6r3) 35
1.48807284 -0.09106818 0.00000000 1.0 # electr. mom.
0.0 0.0 0.8639312209E-02 # magnet. mom.
2.59150928 0.04298365 0.00000000 95.82477960
0.53489436 -1.20761772 0.00000000 606.84022723
1.23557636 -0.00671127 0.00000000 -100.25727379
0.54866949 1.20505723 0.00000000 140.65527614
-0.84197793 1.20827070 0.00000000 90.87267418
-1.55492799 0.00867526 0.00000000 439.77957734
-0.86024592 -1.19659568 0.00000000 -353.41451414
2.53859158 0.04298365 0.00000000 -49.88669285
2.64442698 0.04298365 0.00000000 -38.44625818
2.59150928 -0.00993405 0.00000000 -3.90766085
2.59150928 0.09590135 0.00000000 -4.84615685
0.48197666 -1.20761772 0.00000000 -214.98854611
0.58781206 -1.20761772 0.00000000 -184.12219090
0.53489436 -1.26053542 0.00000000 -103.49581493
0.53489436 -1.15470002 0.00000000 -105.81629314
1.18265866 -0.00671127 0.00000000 255.49410422
1.28849406 -0.00671127 0.00000000 270.59974334
1.23557636 -0.05962897 0.00000000 -223.34285683
1.23557636 0.04620643 0.00000000 -200.46883478
0.49575179 1.20505723 0.00000000 75.14450275
0.60158719 1.20505723 0.00000000 48.90079685
0.54866949 1.15213953 0.00000000 -141.42217603
0.54866949 1.25797493 0.00000000 -123.05166610
-0.89489563 1.20827070 0.00000000 -21.30914170
-0.78906023 1.20827070 0.00000000 -30.23327918
-0.84197793 1.15535300 0.00000000 -18.26947987
-0.84197793 1.26118840 0.00000000 -21.74375851
-1.60784569 0.00867526 0.00000000 -181.02478358
-1.50201029 0.00867526 0.00000000 -182.68935269
-1.55492799 -0.04424244 0.00000000 -32.34233611
-1.55492799 0.06159296 0.00000000 -44.18543741
-0.91316362 -1.19659568 0.00000000 202.93825190
-0.80732822 -1.19659568 0.00000000 242.43571219
-0.86024592 -1.24951338 0.00000000 -40.40657904
-0.86024592 -1.14367798 0.00000000 -49.81456219
35 5758.0 # 1736.7 nm, (r7r3) 35
0.06188870 -0.59855896 0.00000000 1.0 # electr. mom.
0.0 0.0 0.1938816566 # magnet. mom.
2.59150928 0.04298365 0.00000000 -56.80432771
0.53489436 -1.20761772 0.00000000 343.96857984
1.23557636 -0.00671127 0.00000000 68.09730778
0.54866949 1.20505723 0.00000000 -148.10345506
-0.84197793 1.20827070 0.00000000 -81.01286400

-1.55492799 0.00867526 0.00000000 87.84957902
-0.86024592 -1.19659568 0.00000000 45.39205238
2.53859158 0.04298365 0.00000000 25.91228045
2.64442698 0.04298365 0.00000000 19.66331674
2.59150928 -0.00993405 0.00000000 5.46859661
2.59150928 0.09590135 0.00000000 6.28629168
0.48197666 -1.20761772 0.00000000 -190.35928829
0.58781206 -1.20761772 0.00000000 -168.20208542
0.53489436 -1.26053542 0.00000000 5.87880528
0.53489436 -1.15470002 0.00000000 5.81837971
1.18265866 -0.00671127 0.00000000 -36.87609163
1.28849406 -0.00671127 0.00000000 -43.95000989
1.23557636 -0.05962897 0.00000000 23.33978923
1.23557636 0.04620643 0.00000000 -10.87510214
0.49575179 1.20505723 0.00000000 108.70889155
0.60158719 1.20505723 0.00000000 91.08807576
0.54866949 1.15213953 0.00000000 -26.39638805
0.54866949 1.25797493 0.00000000 -23.19109099
-0.89489563 1.20827070 0.00000000 -63.90757104
-0.78906023 1.20827070 0.00000000 -89.97184906
-0.84197793 1.15535300 0.00000000 128.79463229
-0.84197793 1.26118840 0.00000000 105.56864088
-1.60784569 0.00867526 0.00000000 -14.58262229
-1.50201029 0.00867526 0.00000000 -16.30915392
-1.55492799 -0.04424244 0.00000000 -23.59581514
-1.55492799 0.06159296 0.00000000 -33.57896822
-0.91316362 -1.19659568 0.00000000 84.04830912
-0.80732822 -1.19659568 0.00000000 115.53638266
-0.86024592 -1.24951338 0.00000000 -111.13663243
-0.86024592 -1.14367798 0.00000000 -132.56659565
&TRANSITION 11->...
35 1031.0 # 9699.3 nm, (r7r6) 35
-0.02861315 0.22079985 0.00000000 1.0 # electr. mom.
0.0 0.0 -0.6922170416 # magnet. mom.
2.59150928 0.04298365 0.00000000 17.95361717
0.53489436 -1.20761772 0.00000000 -79.68137611
1.23557636 -0.00671127 0.00000000 -115.51194437
0.54866949 1.20505723 0.00000000 196.05966499
-0.84197793 1.20827070 0.00000000 24.18781066
-1.55492799 0.00867526 0.00000000 3.27544963
-0.86024592 -1.19659568 0.00000000 -25.42560389
2.53859158 0.04298365 0.00000000 -14.03637115
2.64442698 0.04298365 0.00000000 -9.91459810
2.59150928 -0.00993405 0.00000000 2.95834714
2.59150928 0.09590135 0.00000000 2.62170547
0.48197666 -1.20761772 0.00000000 22.61200464
0.58781206 -1.20761772 0.00000000 22.98484958
0.53489436 -1.26053542 0.00000000 15.48826464
0.53489436 -1.15470002 0.00000000 18.68170286
1.18265866 -0.00671127 0.00000000 86.35292376
1.28849406 -0.00671127 0.00000000 87.86403403
1.23557636 -0.05962897 0.00000000 -33.32300563
1.23557636 0.04620643 0.00000000 -24.72148320
0.49575179 1.20505723 0.00000000 -24.65676840
0.60158719 1.20505723 0.00000000 -27.48297197
0.54866949 1.15213953 0.00000000 -77.09111131
0.54866949 1.25797493 0.00000000 -67.04909995
-0.89489563 1.20827070 0.00000000 -7.88635483
-0.78906023 1.20827070 0.00000000 -9.47487926
-0.84197793 1.15535300 0.00000000 -4.67172869
-0.84197793 1.26118840 0.00000000 -2.54105434
-1.60784569 0.00867526 0.00000000 -7.26994306
-1.50201029 0.00867526 0.00000000 -5.98156277
-1.55492799 -0.04424244 0.00000000 4.96209787
-1.55492799 0.06159296 0.00000000 5.23788542
-0.91316362 -1.19659568 0.00000000 27.16570651
-0.80732822 -1.19659568 0.00000000 31.49091019
-0.86024592 -1.24951338 0.00000000 -15.41465275
-0.86024592 -1.14367798 0.00000000 -17.76246475
&PERMANENT MOMENTS
35 0.0 # 0 nm, Grd. (r1r1) 35
-0.15749616 -1.86603626 0.00000000 1.0 # electr. mom.
2.59150928 0.04298365 0.00000000 -1924.10150683
0.53489436 -1.20761772 0.00000000 -1472.54504770
1.23557636 -0.00671127 0.00000000 2497.02559066
0.54866949 1.20505723 0.00000000 -1471.43893886
-0.84197793 1.20827070 0.00000000 -616.03295818
-1.55492799 0.00867526 0.00000000 -871.46214854
-0.86024592 -1.19659568 0.00000000 -583.90681046
2.53859158 0.04298365 0.00000000 638.75816098
2.64442698 0.04298365 0.00000000 555.70149154
2.59150928 -0.00993405 0.00000000 400.40103619
2.59150928 0.09590135 0.00000000 335.36552846
0.48197666 -1.20761772 0.00000000 241.18450646
0.58781206 -1.20761772 0.00000000 283.35191280
0.53489436 -1.26053542 0.00000000 440.83310755
0.53489436 -1.15470002 0.00000000 516.39761429

```
          1.18265866          -0.00671127           0.00000000      -539.58730824
          1.28849406          -0.00671127           0.00000000      -549.05070187
          1.23557636          -0.05962897           0.00000000      -769.77916814
          1.23557636           0.04620643           0.00000000      -653.30047766
          0.49575179           1.20505723           0.00000000       544.73951774
          0.60158719           1.20505723           0.00000000       488.11764898
          0.54866949           1.15213953           0.00000000       216.13401744
          0.54866949           1.25797493           0.00000000       227.58120691
         -0.89489563           1.20827070           0.00000000        -0.01520006
         -0.78906023           1.20827070           0.00000000       -87.03183725
         -0.84197793           1.15535300           0.00000000       366.39633254
         -0.84197793           1.26118840           0.00000000       334.42500672
         -1.60784569           0.00867526           0.00000000       403.92961229
         -1.50201029           0.00867526           0.00000000       397.60028582
         -1.55492799          -0.04424244           0.00000000        44.82510989
         -1.55492799           0.06159296           0.00000000        22.37399438
         -0.91316362          -1.19659568           0.00000000       -42.56573429
         -0.80732822          -1.19659568           0.00000000       -77.75350320
         -0.86024592          -1.24951338           0.00000000       350.98667347
         -0.86024592          -1.14367798           0.00000000       352.44298613
35 0.0 # 0 nm, 1st Exc. (r2r2) 35
  -0.27436031    -1.89076488      0.00000000 1.0 # electr. mom.
          2.59150928           0.04298365           0.00000000     -2205.70471032
          0.53489436          -1.20761772           0.00000000     -3003.41318842
          1.23557636          -0.00671127           0.00000000      4740.91499434
          0.54866949           1.20505723           0.00000000     -2124.42920827
         -0.84197793           1.20827070           0.00000000      -548.14161648
         -1.55492799           0.00867526           0.00000000      -825.95875243
         -0.86024592          -1.19659568           0.00000000      -193.71857688
          2.53859158           0.04298365           0.00000000       676.26396192
          2.64442698           0.04298365           0.00000000       570.55042397
          2.59150928          -0.00993405           0.00000000       514.44075974
          2.59150928           0.09590135           0.00000000       453.13345056
          0.48197666          -1.20761772           0.00000000       986.39369962
          0.58781206          -1.20761772           0.00000000       923.96598446
          0.53489436          -1.26053542           0.00000000       509.02108056
          0.53489436          -1.15470002           0.00000000       602.19467539
          1.18265866          -0.00671127           0.00000000     -1278.40392773
          1.28849406          -0.00671127           0.00000000     -1280.73548683
          1.23557636          -0.05962897           0.00000000     -1151.86390584
          1.23557636           0.04620643           0.00000000     -1051.81903709
          0.49575179           1.20505723           0.00000000       815.84302128
          0.60158719           1.20505723           0.00000000       730.27539302
          0.54866949           1.15213953           0.00000000       302.75434656
          0.54866949           1.25797493           0.00000000       285.64775098
         -0.89489563           1.20827070           0.00000000       -96.24443683
         -0.78906023           1.20827070           0.00000000      -204.95098315
         -0.84197793           1.15535300           0.00000000       439.69365494
         -0.84197793           1.26118840           0.00000000       404.76535080
         -1.60784569           0.00867526           0.00000000       429.79578902
         -1.50201029           0.00867526           0.00000000       427.94098488
         -1.55492799          -0.04424244           0.00000000       -13.38929496
         -1.55492799           0.06159296           0.00000000       -21.28232400
         -0.91316362          -1.19659568           0.00000000      -213.96873542
         -0.80732822          -1.19659568           0.00000000      -335.15434598
         -0.86024592          -1.24951338           0.00000000       360.35389915
         -0.86024592          -1.14367798           0.00000000       375.22930939
35 0.0 # 0 nm, 1st Exc. (r2r2) 35
  -0.27436031    -1.89076488      0.00000000 1.0 # electr. mom.
          2.59150928           0.04298365           0.00000000     -2205.70471032
          0.53489436          -1.20761772           0.00000000     -3003.41318842
          1.23557636          -0.00671127           0.00000000      4740.91499434
          0.54866949           1.20505723           0.00000000     -2124.42920827
         -0.84197793           1.20827070           0.00000000      -548.14161648
         -1.55492799           0.00867526           0.00000000      -825.95875243
         -0.86024592          -1.19659568           0.00000000      -193.71857688
          2.53859158           0.04298365           0.00000000       676.26396192
          2.64442698           0.04298365           0.00000000       570.55042397
          2.59150928          -0.00993405           0.00000000       514.44075974
          2.59150928           0.09590135           0.00000000       453.13345056
          0.48197666          -1.20761772           0.00000000       986.39369962
          0.58781206          -1.20761772           0.00000000       923.96598446
          0.53489436          -1.26053542           0.00000000       509.02108056
          0.53489436          -1.15470002           0.00000000       602.19467539
          1.18265866          -0.00671127           0.00000000     -1278.40392773
          1.28849406          -0.00671127           0.00000000     -1280.73548683
          1.23557636          -0.05962897           0.00000000     -1151.86390584
          1.23557636           0.04620643           0.00000000     -1051.81903709
          0.49575179           1.20505723           0.00000000       815.84302128
          0.60158719           1.20505723           0.00000000       730.27539302
          0.54866949           1.15213953           0.00000000       302.75434656
          0.54866949           1.25797493           0.00000000       285.64775098
         -0.89489563           1.20827070           0.00000000       -96.24443683
         -0.78906023           1.20827070           0.00000000      -204.95098315
         -0.84197793           1.15535300           0.00000000       439.69365494
         -0.84197793           1.26118840           0.00000000       404.76535080
         -1.60784569           0.00867526           0.00000000       429.79578902
```

-1.50201029 0.00867526 0.00000000 427.94098488
-1.55492799 -0.04424244 0.00000000 -13.38929496
-1.55492799 0.06159296 0.00000000 -21.28232400
-0.91316362 -1.19659568 0.00000000 -213.96873542
-0.80732822 -1.19659568 0.00000000 -335.15434598
-0.86024592 -1.24951338 0.00000000 360.35389915
-0.86024592 -1.14367798 0.00000000 375.22930939
35 0.0 # 0 nm, 1st Exc. (r2r2) 35
-0.27436031 -1.89076488 0.00000000 1.0 # electr. mom.
2.59150928 0.04298365 0.00000000 -2205.70471032
0.53489436 -1.20761772 0.00000000 -3003.41318842
1.23557636 -0.00671127 0.00000000 4740.91499434
0.54866949 1.20505723 0.00000000 -2124.42920827
-0.84197793 1.20827070 0.00000000 -548.14161648
-1.55492799 0.00867526 0.00000000 -825.95875243
-0.86024592 -1.19659568 0.00000000 -193.71857688
2.53859158 0.04298365 0.00000000 676.26396192
2.64442698 0.04298365 0.00000000 570.55042397
2.59150928 -0.00993405 0.00000000 514.44075974
2.59150928 0.09590135 0.00000000 453.13345056
0.48197666 -1.20761772 0.00000000 986.39369962
0.58781206 -1.20761772 0.00000000 923.96598446
0.53489436 -1.26053542 0.00000000 509.02108056
0.53489436 -1.15470002 0.00000000 602.19467539
1.18265866 -0.00671127 0.00000000 -1278.40392773
1.28849406 -0.00671127 0.00000000 -1280.73548683
1.23557636 -0.05962897 0.00000000 -1151.86390584
1.23557636 0.04620643 0.00000000 -1051.81903709
0.49575179 1.20505723 0.00000000 815.84302128
0.60158719 1.20505723 0.00000000 730.27539302
0.54866949 1.15213953 0.00000000 302.75434656
0.54866949 1.25797493 0.00000000 285.64775098
-0.89489563 1.20827070 0.00000000 -96.24443683
-0.78906023 1.20827070 0.00000000 -204.95098315
-0.84197793 1.15535300 0.00000000 439.69365494
-0.84197793 1.26118840 0.00000000 404.76535080
-1.60784569 0.00867526 0.00000000 429.79578902
-1.50201029 0.00867526 0.00000000 427.94098488
-1.55492799 -0.04424244 0.00000000 -13.38929496
-1.55492799 0.06159296 0.00000000 -21.28232400
-0.91316362 -1.19659568 0.00000000 -213.96873542
-0.80732822 -1.19659568 0.00000000 -335.15434598
-0.86024592 -1.24951338 0.00000000 360.35389915
-0.86024592 -1.14367798 0.00000000 375.22930939
35 0.0 # 0 nm, 1st Exc. (r2r2) 35
-0.27436031 -1.89076488 0.00000000 1.0 # electr. mom.
2.59150928 0.04298365 0.00000000 -2205.70471032
0.53489436 -1.20761772 0.00000000 -3003.41318842
1.23557636 -0.00671127 0.00000000 4740.91499434
0.54866949 1.20505723 0.00000000 -2124.42920827
-0.84197793 1.20827070 0.00000000 -548.14161648
-1.55492799 0.00867526 0.00000000 -825.95875243
-0.86024592 -1.19659568 0.00000000 -193.71857688
2.53859158 0.04298365 0.00000000 676.26396192
2.64442698 0.04298365 0.00000000 570.55042397
2.59150928 -0.00993405 0.00000000 514.44075974
2.59150928 0.09590135 0.00000000 453.13345056
0.48197666 -1.20761772 0.00000000 986.39369962
0.58781206 -1.20761772 0.00000000 923.96598446
0.53489436 -1.26053542 0.00000000 509.02108056
0.53489436 -1.15470002 0.00000000 602.19467539
1.18265866 -0.00671127 0.00000000 -1278.40392773
1.28849406 -0.00671127 0.00000000 -1280.73548683
1.23557636 -0.05962897 0.00000000 -1151.86390584
1.23557636 0.04620643 0.00000000 -1051.81903709
0.49575179 1.20505723 0.00000000 815.84302128
0.60158719 1.20505723 0.00000000 730.27539302
0.54866949 1.15213953 0.00000000 302.75434656
0.54866949 1.25797493 0.00000000 285.64775098
-0.89489563 1.20827070 0.00000000 -96.24443683
-0.78906023 1.20827070 0.00000000 -204.95098315
-0.84197793 1.15535300 0.00000000 439.69365494
-0.84197793 1.26118840 0.00000000 404.76535080
-1.60784569 0.00867526 0.00000000 429.79578902
-1.50201029 0.00867526 0.00000000 427.94098488
-1.55492799 -0.04424244 0.00000000 -13.38929496
-1.55492799 0.06159296 0.00000000 -21.28232400
-0.91316362 -1.19659568 0.00000000 -213.96873542
-0.80732822 -1.19659568 0.00000000 -335.15434598
-0.86024592 -1.24951338 0.00000000 360.35389915
-0.86024592 -1.14367798 0.00000000 375.22930939
35 0.0 # 0 nm, 1st Exc. (r2r2) 35
-0.27436031 -1.89076488 0.00000000 1.0 # electr. mom.
2.59150928 0.04298365 0.00000000 -2205.70471032
0.53489436 -1.20761772 0.00000000 -3003.41318842
1.23557636 -0.00671127 0.00000000 4740.91499434
0.54866949 1.20505723 0.00000000 -2124.42920827

```
-0.84197793   1.20827070   0.00000000   -548.14161648
-1.55492799   0.00867526   0.00000000   -825.95875243
-0.86024592  -1.19659568   0.00000000   -193.71857688
 2.53859158   0.04298365   0.00000000    676.26396192
 2.64442698   0.04298365   0.00000000    570.55042397
 2.59150928  -0.00993405   0.00000000    514.44075974
 2.59150928   0.09590135   0.00000000    453.13345056
 0.48197666  -1.20761772   0.00000000    986.39369962
 0.58781206  -1.20761772   0.00000000    923.96598446
 0.53489436  -1.26053542   0.00000000    509.02108056
 0.53489436  -1.15470002   0.00000000    602.19467539
 1.18265866  -0.00671127   0.00000000  -1278.40392773
 1.28849406  -0.00671127   0.00000000  -1280.73548683
 1.23557636  -0.05962897   0.00000000  -1151.86390584
 1.23557636   0.04620643   0.00000000  -1051.81903709
 0.49575179   1.20505723   0.00000000    815.84302128
 0.60158719   1.20505723   0.00000000    730.27539302
 0.54866949   1.15213953   0.00000000    302.75434656
 0.54866949   1.25797493   0.00000000    285.64775098
-0.89489563   1.20827070   0.00000000    -96.24443683
-0.78906023   1.20827070   0.00000000   -204.95098315
-0.84197793   1.15535300   0.00000000    439.69365494
-0.84197793   1.26118840   0.00000000    404.76535080
-1.60784569   0.00867526   0.00000000    429.79578902
-1.50201029   0.00867526   0.00000000    427.94098488
-1.55492799  -0.04424244   0.00000000    -13.38929496
-1.55492799   0.06159296   0.00000000    -21.28232400
-0.91316362  -1.19659568   0.00000000   -213.96873542
-0.80732822  -1.19659568   0.00000000   -335.15434598
-0.86024592  -1.24951338   0.00000000    360.35389915
-0.86024592  -1.14367798   0.00000000    375.22930939
35 0.0 # 0 nm, 1st Exc. (r2r2) 35
 -0.27436031    -1.89076488    0.00000000 1.0 # electr. mom.
 2.59150928   0.04298365   0.00000000  -2205.70471032
 0.53489436  -1.20761772   0.00000000  -3003.41318842
 1.23557636  -0.00671127   0.00000000   4740.91499434
 0.54866949   1.20505723   0.00000000  -2124.42920827
-0.84197793   1.20827070   0.00000000   -548.14161648
-1.55492799   0.00867526   0.00000000   -825.95875243
-0.86024592  -1.19659568   0.00000000   -193.71857688
 2.53859158   0.04298365   0.00000000    676.26396192
 2.64442698   0.04298365   0.00000000    570.55042397
 2.59150928  -0.00993405   0.00000000    514.44075974
 2.59150928   0.09590135   0.00000000    453.13345056
 0.48197666  -1.20761772   0.00000000    986.39369962
 0.58781206  -1.20761772   0.00000000    923.96598446
 0.53489436  -1.26053542   0.00000000    509.02108056
 0.53489436  -1.15470002   0.00000000    602.19467539
 1.18265866  -0.00671127   0.00000000  -1278.40392773
 1.28849406  -0.00671127   0.00000000  -1280.73548683
 1.23557636  -0.05962897   0.00000000  -1151.86390584
 1.23557636   0.04620643   0.00000000  -1051.81903709
 0.49575179   1.20505723   0.00000000    815.84302128
 0.60158719   1.20505723   0.00000000    730.27539302
 0.54866949   1.15213953   0.00000000    302.75434656
 0.54866949   1.25797493   0.00000000    285.64775098
-0.89489563   1.20827070   0.00000000    -96.24443683
-0.78906023   1.20827070   0.00000000   -204.95098315
-0.84197793   1.15535300   0.00000000    439.69365494
-0.84197793   1.26118840   0.00000000    404.76535080
-1.60784569   0.00867526   0.00000000    429.79578902
-1.50201029   0.00867526   0.00000000    427.94098488
-1.55492799  -0.04424244   0.00000000    -13.38929496
-1.55492799   0.06159296   0.00000000    -21.28232400
-0.91316362  -1.19659568   0.00000000   -213.96873542
-0.80732822  -1.19659568   0.00000000   -335.15434598
-0.86024592  -1.24951338   0.00000000    360.35389915
-0.86024592  -1.14367798   0.00000000    375.22930939
35 0.0 # 0 nm, 1st Exc. (r2r2) 35
 -0.27436031    -1.89076488    0.00000000 1.0 # electr. mom.
 2.59150928   0.04298365   0.00000000  -2205.70471032
 0.53489436  -1.20761772   0.00000000  -3003.41318842
 1.23557636  -0.00671127   0.00000000   4740.91499434
 0.54866949   1.20505723   0.00000000  -2124.42920827
-0.84197793   1.20827070   0.00000000   -548.14161648
-1.55492799   0.00867526   0.00000000   -825.95875243
-0.86024592  -1.19659568   0.00000000   -193.71857688
 2.53859158   0.04298365   0.00000000    676.26396192
 2.64442698   0.04298365   0.00000000    570.55042397
 2.59150928  -0.00993405   0.00000000    514.44075974
 2.59150928   0.09590135   0.00000000    453.13345056
 0.48197666  -1.20761772   0.00000000    986.39369962
 0.58781206  -1.20761772   0.00000000    923.96598446
 0.53489436  -1.26053542   0.00000000    509.02108056
 0.53489436  -1.15470002   0.00000000    602.19467539
 1.18265866  -0.00671127   0.00000000  -1278.40392773
 1.28849406  -0.00671127   0.00000000  -1280.73548683
```

```
    1.23557636    -0.05962897     0.00000000   -1151.86390584
    1.23557636     0.04620643     0.00000000   -1051.81903709
    0.49575179     1.20505723     0.00000000     815.84302128
    0.60158719     1.20505723     0.00000000     730.27539302
    0.54866949     1.15213953     0.00000000     302.75434656
    0.54866949     1.25797493     0.00000000     285.64775098
   -0.89489563     1.20827070     0.00000000     -96.24443683
   -0.78906023     1.20827070     0.00000000    -204.95098315
   -0.84197793     1.15535300     0.00000000     439.69365494
   -0.84197793     1.26118840     0.00000000     404.76535080
   -1.60784569     0.00867526     0.00000000     429.79578902
   -1.50201029     0.00867526     0.00000000     427.94098488
   -1.55492799    -0.04424244     0.00000000     -13.38929496
   -1.55492799     0.06159296     0.00000000     -21.28232400
   -0.91316362    -1.19659568     0.00000000    -213.96873542
   -0.80732822    -1.19659568     0.00000000    -335.15434598
   -0.86024592    -1.24951338     0.00000000     360.35389915
   -0.86024592    -1.14367798     0.00000000     375.22930939
35 0.0 # 0 nm, 1st Exc. (r2r2) 35
 -0.27436031    -1.89076488     0.00000000 1.0 # electr. mom.
    2.59150928     0.04298365     0.00000000   -2205.70471032
    0.53489436    -1.20761772     0.00000000   -3003.41318842
    1.23557636    -0.00671127     0.00000000    4740.91499434
    0.54866949     1.20505723     0.00000000   -2124.42920827
   -0.84197793     1.20827070     0.00000000    -548.14161648
   -1.55492799     0.00867526     0.00000000    -825.95875243
   -0.86024592    -1.19659568     0.00000000    -193.71857688
    2.53859158     0.04298365     0.00000000     676.26396192
    2.64442698     0.04298365     0.00000000     570.55042397
    2.59150928    -0.00993405     0.00000000     514.44075974
    2.59150928     0.09590135     0.00000000     453.13345056
    0.48197666    -1.20761772     0.00000000     986.39369962
    0.58781206    -1.20761772     0.00000000     923.96598446
    0.53489436    -1.26053542     0.00000000     509.02108056
    0.53489436    -1.15470002     0.00000000     602.19467539
    1.18265866    -0.00671127     0.00000000   -1278.40392773
    1.28849406    -0.00671127     0.00000000   -1280.73548683
    1.23557636    -0.05962897     0.00000000   -1151.86390584
    1.23557636     0.04620643     0.00000000   -1051.81903709
    0.49575179     1.20505723     0.00000000     815.84302128
    0.60158719     1.20505723     0.00000000     730.27539302
    0.54866949     1.15213953     0.00000000     302.75434656
    0.54866949     1.25797493     0.00000000     285.64775098
   -0.89489563     1.20827070     0.00000000     -96.24443683
   -0.78906023     1.20827070     0.00000000    -204.95098315
   -0.84197793     1.15535300     0.00000000     439.69365494
   -0.84197793     1.26118840     0.00000000     404.76535080
   -1.60784569     0.00867526     0.00000000     429.79578902
   -1.50201029     0.00867526     0.00000000     427.94098488
   -1.55492799    -0.04424244     0.00000000     -13.38929496
   -1.55492799     0.06159296     0.00000000     -21.28232400
   -0.91316362    -1.19659568     0.00000000    -213.96873542
   -0.80732822    -1.19659568     0.00000000    -335.15434598
   -0.86024592    -1.24951338     0.00000000     360.35389915
   -0.86024592    -1.14367798     0.00000000     375.22930939
35 0.0 # 0 nm, 2nd Exc. (r3r3) 35
  2.57808686    -1.78202820     0.00000000 1.0 # electr. mom.
    2.59150928     0.04298365     0.00000000   -2130.28508213
    0.53489436    -1.20761772     0.00000000   -4141.60188144
    1.23557636    -0.00671127     0.00000000    5249.55833784
    0.54866949     1.20505723     0.00000000   -2442.45488717
   -0.84197793     1.20827070     0.00000000    -298.86572981
   -1.55492799     0.00867526     0.00000000   -1916.94511632
   -0.86024592    -1.19659568     0.00000000     448.98565277
    2.53859158     0.04298365     0.00000000     651.50498837
    2.64442698     0.04298365     0.00000000     540.36514541
    2.59150928    -0.00993405     0.00000000     504.35508821
    2.59150928     0.09590135     0.00000000     444.03678384
    0.48197666    -1.20761772     0.00000000    1553.80218230
    0.58781206    -1.20761772     0.00000000    1410.00418930
    0.53489436    -1.26053542     0.00000000     554.08593571
    0.53489436    -1.15470002     0.00000000     648.55549555
    1.18265866    -0.00671127     0.00000000   -1528.37101934
    1.28849406    -0.00671127     0.00000000   -1545.73866072
    1.23557636    -0.05962897     0.00000000   -1149.49572835
    1.23557636     0.04620643     0.00000000   -1048.91683061
    0.49575179     1.20505723     0.00000000     934.27349074
    0.60158719     1.20505723     0.00000000     827.46651058
    0.54866949     1.15213953     0.00000000     357.47538221
    0.54866949     1.25797493     0.00000000     335.65543056
   -0.89489563     1.20827070     0.00000000    -268.52914099
   -0.78906023     1.20827070     0.00000000    -407.67874094
   -0.84197793     1.15535300     0.00000000     502.37847187
   -0.84197793     1.26118840     0.00000000     465.29809819
   -1.60784569     0.00867526     0.00000000     594.36510797
   -1.50201029     0.00867526     0.00000000     607.39937016
   -1.55492799    -0.04424244     0.00000000     360.84938443
```

```
-1.55492799 0.06159296 0.00000000 351.71309141
-0.91316362 -1.19659568 0.00000000 -550.66996867
-0.80732822 -1.19659568 0.00000000 -755.47941408
-0.86024592 -1.24951338 0.00000000 411.56188027
-0.86024592 -1.14367798 0.00000000 431.34218280
35 0.0 # 0 nm, 3rd Exc. (r6r6) 35
1.94379795 -1.83538966 0.00000000 1.0 # electr. mom.
2.59150928 0.04298365 0.00000000 -2104.92830160
0.53489436 -1.20761772 0.00000000 -2968.94001955
1.23557636 -0.00671127 0.00000000 4778.39202974
0.54866949 1.20505723 0.00000000 -2348.00595902
-0.84197793 1.20827070 0.00000000 -711.50000990
-1.55492799 0.00867526 0.00000000 -1176.99612576
-0.86024592 -1.19659568 0.00000000 -419.58363538
2.53859158 0.04298365 0.00000000 660.31864008
2.64442698 0.04298365 0.00000000 560.64612211
2.59150928 -0.00993405 0.00000000 478.01464896
2.59150928 0.09590135 0.00000000 414.52018886
0.48197666 -1.20761772 0.00000000 1025.17773394
0.58781206 -1.20761772 0.00000000 955.98062568
0.53489436 -1.26053542 0.00000000 459.39825538
0.53489436 -1.15470002 0.00000000 546.87444686
1.18265866 -0.00671127 0.00000000 -1240.24718275
1.28849406 -0.00671127 0.00000000 -1239.80410814
1.23557636 -0.05962897 0.00000000 -1211.66279314
1.23557636 0.04620643 0.00000000 -1107.87477350
0.49575179 1.20505723 0.00000000 957.29562096
0.60158719 1.20505723 0.00000000 854.95073683
0.54866949 1.15213953 0.00000000 278.82492264
0.54866949 1.25797493 0.00000000 267.91960997
-0.89489563 1.20827070 0.00000000 -37.06887062
-0.78906023 1.20827070 0.00000000 -151.96975344
-0.84197793 1.15535300 0.00000000 466.25421096
-0.84197793 1.26118840 0.00000000 428.56477138
-1.60784569 0.00867526 0.00000000 508.48388832
-1.50201029 0.00867526 0.00000000 507.86096021
-1.55492799 -0.04424244 0.00000000 84.88229856
-1.55492799 0.06159296 0.00000000 72.39201144
-0.91316362 -1.19659568 0.00000000 -167.27068368
-0.80732822 -1.19659568 0.00000000 -286.18744234
-0.86024592 -1.24951338 0.00000000 424.31954491
-0.86024592 -1.14367798 0.00000000 440.96839094
35 0.0 # 0 nm, 4th Exc. (r7r7) 35
0.04690673 -1.76174511 0.00000000 1.0 # electr. mom.
2.59150928 0.04298365 0.00000000 -2204.12660366
0.53489436 -1.20761772 0.00000000 -3323.73920011
1.23557636 -0.00671127 0.00000000 4830.30737606
0.54866949 1.20505723 0.00000000 -2341.95905122
-0.84197793 1.20827070 0.00000000 -538.29001594
-1.55492799 0.00867526 0.00000000 -1193.05124006
-0.86024592 -1.19659568 0.00000000 -209.74860398
2.53859158 0.04298365 0.00000000 666.00444312
2.64442698 0.04298365 0.00000000 560.04657365
2.59150928 -0.00993405 0.00000000 523.84917134
2.59150928 0.09590135 0.00000000 463.04404680
0.48197666 -1.20761772 0.00000000 1063.56867869
0.58781206 -1.20761772 0.00000000 995.30997024
0.53489436 -1.26053542 0.00000000 591.06339101
0.53489436 -1.15470002 0.00000000 693.14805566
1.18265866 -0.00671127 0.00000000 -1352.48610043
1.28849406 -0.00671127 0.00000000 -1347.30507154
1.23557636 -0.05962897 0.00000000 -1126.60287792
1.23557636 0.04620643 0.00000000 -1026.88980154
0.49575179 1.20505723 0.00000000 875.30309938
0.60158719 1.20505723 0.00000000 781.33299917
0.54866949 1.15213953 0.00000000 360.82675570
0.54866949 1.25797493 0.00000000 336.06115358
-0.89489563 1.20827070 0.00000000 -150.04387037
-0.78906023 1.20827070 0.00000000 -273.35614930
-0.84197793 1.15535300 0.00000000 494.39510477
-0.84197793 1.26118840 0.00000000 461.02928021
-1.60784569 0.00867526 0.00000000 498.43575038
-1.50201029 0.00867526 0.00000000 508.06344384
-1.55492799 -0.04424244 0.00000000 94.65384662
-1.55492799 0.06159296 0.00000000 89.75177280
-0.91316362 -1.19659568 0.00000000 -216.07583453
-0.80732822 -1.19659568 0.00000000 -347.77335442
-0.86024592 -1.24951338 0.00000000 376.80039989
-0.86024592 -1.14367798 0.00000000 388.45246210
```

```
-TRPVIB00-
9 # Tryptophan_aromatic group / toluene
 1.32524160   1.15754119   0.00000000 1.0   # Cg
-0.04178901   0.70146732   0.00000000 1.0   # Cd2
 0.00000000  -0.70208856   0.00000000 1.0   # Ce2
-1.28611127   1.35334701   0.00000000 1.0   # Ce3
 2.11092008   0.04406598   0.00000000 1.0   # Cd1
 1.31876712  -1.08237515   0.00000000 1.0   # Ne1
-1.16182210  -1.48163547   0.00000000 1.0   # Cz2
-2.44031892   0.59060606   0.00000000 1.0   # Cz3
-2.37713680  -0.81881549   0.00000000 1.0   # Ch2
&TRANSITION 1->...
45   34424   # 290.5 nm,   (r2r1v1) 45 = 1Lb_v1 state
 0.446297     0.428546     0.00000000 1.0   # electr. mom.
 0.00000000   0.00000000   0.6618344880E-01   # magnet. mom.
 1.31876649  -1.08237463   0.00000000  -277.64078714
 0.00000000  -0.70208822   0.00000000  -461.75329033
 2.11091906   0.04406596   0.00000000   540.42202991
 1.32524096   1.15754064   0.00000000  -347.77662620
-0.04178899   0.70146698   0.00000000   433.76265503
-1.16182154  -1.48163476   0.00000000   202.72961486
-2.37713566  -0.81881509   0.00000000   -71.94993426
-2.44031774   0.59060577   0.00000000   -12.97513683
-1.28611065   1.35334636   0.00000000    32.30473501
 1.26584879  -1.08237463   0.00000000   111.05542497
 1.37168419  -1.08237463   0.00000000   101.60083067
 1.31876649  -1.13529233   0.00000000    31.52825164
 1.31876649  -1.02945693   0.00000000    33.67735344
-0.05291770  -0.70208822   0.00000000   164.91310212
 0.05291770  -0.70208822   0.00000000   164.87789491
 0.00000000  -0.75500592   0.00000000    64.20899956
 0.00000000  -0.64917052   0.00000000    70.05420401
 2.05800137   0.04406596   0.00000000   -66.63753945
 2.16383676   0.04406596   0.00000000   -53.97027625
 2.11091906  -0.00885174   0.00000000  -207.56251836
 2.11091906   0.09698366   0.00000000  -212.98399500
 1.27232327   1.15754064   0.00000000   179.86698343
 1.37815866   1.15754064   0.00000000   157.97088538
 1.32524096   1.10462294   0.00000000     7.29782154
 1.32524096   1.21045834   0.00000000     4.57900696
-0.09470669   0.70146698   0.00000000     2.28995459
 0.01112871   0.70146698   0.00000000    -9.09822488
-0.04178899   0.64854929   0.00000000  -221.37782800
-0.04178899   0.75438469   0.00000000  -208.51453764
-1.21473924  -1.48163476   0.00000000   -53.64106404
-1.10890384  -1.48163476   0.00000000   -58.08421463
-1.16182154  -1.53455246   0.00000000   -39.98894314
-1.16182154  -1.42871706   0.00000000   -52.50443795
-2.43005336  -0.81881509   0.00000000    23.43428639
-2.32421796  -0.81881509   0.00000000    30.36073870
-2.37713566  -0.87173279   0.00000000     8.81465922
-2.37713566  -0.76589740   0.00000000    10.14078687
-2.49323545   0.59060577   0.00000000    16.10707973
-2.38740004   0.59060577   0.00000000    15.95258558
-2.44031774   0.53768808   0.00000000   -11.27038448
-2.44031774   0.64352347   0.00000000    -8.15292193
-1.33902835   1.35334636   0.00000000    10.52086740
-1.23319294   1.35334636   0.00000000    12.99171097
-1.28611065   1.30042866   0.00000000   -29.81988328
-1.28611065   1.40626406   0.00000000   -25.75991909
45   35273   # 283.5 nm,   (r2v2r1) 45 = 1Lb_v2 state
 0.338322     0.324866     0.00000000 1.0   # electr. mom.
 0.00000000   0.00000000   0.6618344880E-01   # magnet. mom.
 1.31876649  -1.08237463   0.00000000  -210.46962896
 0.00000000  -0.70208822   0.00000000  -350.03878461
 2.11091906   0.04406596   0.00000000   409.67476461
 1.32524096   1.15754064   0.00000000  -263.63711986
-0.04178899   0.70146698   0.00000000   328.82007720
-1.16182154  -1.48163476   0.00000000   153.68212740
-2.37713566  -0.81881509   0.00000000   -54.54269210
-2.44031774   0.59060577   0.00000000    -9.83599082
-1.28611065   1.35334636   0.00000000    24.48907332
 1.26584879  -1.08237463   0.00000000    84.18717699
 1.37168419  -1.08237463   0.00000000    77.01998454
 1.31876649  -1.13529233   0.00000000    23.90044883
 1.31876649  -1.02945693   0.00000000    25.52960664
-0.05291770  -0.70208822   0.00000000   125.01477096
 0.05291770  -0.70208822   0.00000000   124.98808162
 0.00000000  -0.75500592   0.00000000    48.67456418
 0.00000000  -0.64917052   0.00000000    53.10560627
 2.05800137   0.04406596   0.00000000   -50.51555410
 2.16383676   0.04406596   0.00000000   -40.91295135
 2.11091906  -0.00885174   0.00000000  -157.34578005
 2.11091906   0.09698366   0.00000000  -161.45560911
 1.27232327   1.15754064   0.00000000   136.35077776
 1.37815866   1.15754064   0.00000000   119.75212279
 1.32524096   1.10462294   0.00000000     5.53221955
```

```
 1.32524096    1.21045834   0.00000000     3.47118269
-0.09470669    0.70146698   0.00000000     1.73593332
 0.01112871    0.70146698   0.00000000    -6.89704144
-0.04178899    0.64854929   0.00000000  -167.81867606
-0.04178899    0.75438469   0.00000000  -158.06747208
-1.21473924   -1.48163476   0.00000000   -40.66338726
-1.10890384   -1.48163476   0.00000000   -44.03158206
-1.16182154   -1.53455246   0.00000000   -30.31419883
-1.16182154   -1.42871706   0.00000000   -39.80175135
-2.43005336   -0.81881509   0.00000000    17.76470097
-2.32421796   -0.81881509   0.00000000    23.01539869
-2.37713566   -0.87173279   0.00000000     6.68208038
-2.37713566   -0.76589740   0.00000000     7.68737069
-2.49323545    0.59060577   0.00000000    12.21020560
-2.38740004    0.59060577   0.00000000    12.09308907
-2.44031774    0.53768808   0.00000000    -8.54367856
-2.44031774    0.64352347   0.00000000    -6.18044082
-1.33902835    1.35334636   0.00000000     7.97549625
-1.23319294    1.35334636   0.00000000     9.84855509
-1.28611065    1.30042866   0.00000000   -22.60539539
-1.28611065    1.40626406   0.00000000   -19.52768060
45    35381    # 282.6 nm,    (r2v3r1) 45 = 1Lb_v3 state
 0.316727      0.304129     0.00000000 1.0      # electr. mom.
 0.00000000    0.00000000   0.6618344880E-01    # magnet. mom.
 1.31876649   -1.08237463   0.00000000  -197.03539733
 0.00000000   -0.70208822   0.00000000  -327.69588346
 2.11091906    0.04406596   0.00000000   383.52531155
 1.32524096    1.15754064   0.00000000  -246.80921859
-0.04178899    0.70146698   0.00000000   307.83156164
-1.16182154   -1.48163476   0.00000000   143.87262990
-2.37713566   -0.81881509   0.00000000   -51.06124367
-2.44031774    0.59060577   0.00000000    -9.20816162
-1.28611065    1.35334636   0.00000000    22.92594098
 1.26584879   -1.08237463   0.00000000    78.81352740
 1.37168419   -1.08237463   0.00000000    72.10381532
 1.31876649   -1.13529233   0.00000000    22.37488826
 1.31876649   -1.02945693   0.00000000    23.90005728
-0.05291770   -0.70208822   0.00000000   117.03510473
 0.05291770   -0.70208822   0.00000000   117.01011897
 0.00000000   -0.75500592   0.00000000    45.56767710
 0.00000000   -0.64917052   0.00000000    49.71588672
 2.05800137    0.04406596   0.00000000   -47.29115703
 2.16383676    0.04406596   0.00000000   -38.30148637
 2.11091906   -0.00885174   0.00000000  -147.30243238
 2.11091906    0.09698366   0.00000000  -151.14993194
 1.27232327    1.15754064   0.00000000   127.64753663
 1.37815866    1.15754064   0.00000000   112.10837027
 1.32524096    1.10462294   0.00000000     5.17909916
 1.32524096    1.21045834   0.00000000     3.24961784
-0.09470669    0.70146698   0.00000000     1.62512907
 0.01112871    0.70146698   0.00000000    -6.45680475
-0.04178899    0.64854929   0.00000000  -157.10684568
-0.04178899    0.75438469   0.00000000  -147.97805897
-1.21473924   -1.48163476   0.00000000   -38.06785190
-1.10890384   -1.48163476   0.00000000   -41.22105555
-1.16182154   -1.53455246   0.00000000   -28.37924997
-1.16182154   -1.42871706   0.00000000   -37.26121403
-2.43005336   -0.81881509   0.00000000    16.63078389
-2.32421796   -0.81881509   0.00000000    21.54633069
-2.37713566   -0.87173279   0.00000000     6.25556461
-2.37713566   -0.76589740   0.00000000     7.19668746
-2.49323545    0.59060577   0.00000000    11.43083078
-2.38740004    0.59060577   0.00000000    11.32118977
-2.44031774    0.53768808   0.00000000    -7.99833738
-2.44031774    0.64352347   0.00000000    -5.78594460
-1.33902835    1.35334636   0.00000000     7.46642202
-1.23319294    1.35334636   0.00000000     9.21992392
-1.28611065    1.30042866   0.00000000   -21.16249781
-1.28611065    1.40626406   0.00000000   -18.28123290
45    35972    # 278.0 nm,    (r2v4r1) 45 = 1Lb_v4 state
 0.165562      0.158977     0.00000000 1.0      # electr. mom.
 0.00000000    0.00000000   0.6618344880E-01    # magnet. mom.
 1.31876649   -1.08237463   0.00000000  -102.99577588
 0.00000000   -0.70208822   0.00000000  -171.29557545
 2.11091906    0.04406596   0.00000000   200.47914013
 1.32524096    1.15754064   0.00000000  -129.01390972
-0.04178899    0.70146698   0.00000000   160.91195267
-1.16182154   -1.48163476   0.00000000    75.20614745
-2.37713566   -0.81881509   0.00000000   -26.69110464
-2.44031774    0.59060577   0.00000000    -4.81335721
-1.28611065    1.35334636   0.00000000    11.98401460
 1.26584879   -1.08237463   0.00000000    41.19798023
 1.37168419   -1.08237463   0.00000000    37.69063073
 1.31876649   -1.13529233   0.00000000    11.69596432
 1.31876649   -1.02945693   0.00000000    12.49321176
-0.05291770   -0.70208822   0.00000000    61.17744111
 0.05291770   -0.70208822   0.00000000    61.16438037
```

0.00000000 -0.75500592 0.00000000 23.81946758
0.00000000 -0.64917052 0.00000000 25.98784987
2.05800137 0.04406596 0.00000000 -24.72037754
2.16383676 0.04406596 0.00000000 -20.02123151
2.11091906 -0.00885174 0.00000000 -76.99899875
2.11091906 0.09698366 0.00000000 -79.01019169
1.27232327 1.15754064 0.00000000 66.72484869
1.37815866 1.15754064 0.00000000 58.60210264
1.32524096 1.10462294 0.00000000 2.70725638
1.32524096 1.21045834 0.00000000 1.69866387
-0.09470669 0.70146698 0.00000000 0.84949928
0.01112871 0.70146698 0.00000000 -3.37514794
-0.04178899 0.64854929 0.00000000 -82.12403297
-0.04178899 0.75438469 0.00000000 -77.35216719
-1.21473924 -1.48163476 0.00000000 -19.89910440
-1.10890384 -1.48163476 0.00000000 -21.54736994
-1.16182154 -1.53455246 0.00000000 -14.83460794
-1.16182154 -1.42871706 0.00000000 -19.47745279
-2.43005336 -0.81881509 0.00000000 8.69336431
-2.32421796 -0.81881509 0.00000000 11.26285468
-2.37713566 -0.87173279 0.00000000 3.26995423
-2.37713566 -0.76589740 0.00000000 3.76190481
-2.49323545 0.59060577 0.00000000 5.97520700
-2.38740004 0.59060577 0.00000000 5.91789465
-2.44031774 0.53768808 0.00000000 -4.18094908
-2.44031774 0.64352347 0.00000000 -3.02447104
-1.33902835 1.35334636 0.00000000 3.90290242
-1.23319294 1.35334636 0.00000000 4.81950568
-1.28611065 1.30042866 0.00000000 -11.06221477
-1.28611065 1.40626406 0.00000000 -9.55609902
45 36157 # 276.6 nm, (r2v5r1) 45 = 1Lb_v5 state
0.237545 0.228097 0.00000000 1.0 # electr. mom.
0.00000000 0.00000000 0.6618344880E-01 # magnet. mom.
1.31876649 -1.08237463 0.00000000 -147.77654800
0.00000000 -0.70208822 0.00000000 -245.77191260
2.11091906 0.04406596 0.00000000 287.64398366
1.32524096 1.15754064 0.00000000 -185.10691395
-0.04178899 0.70146698 0.00000000 230.87367123
-1.16182154 -1.48163476 0.00000000 107.90447243
-2.37713566 -0.81881509 0.00000000 -38.29593275
-2.44031774 0.59060577 0.00000000 -6.90612122
-1.28611065 1.35334636 0.00000000 17.19445573
1.26584879 -1.08237463 0.00000000 59.11014555
1.37168419 -1.08237463 0.00000000 54.07786149
1.31876649 -1.13529233 0.00000000 16.78116620
1.31876649 -1.02945693 0.00000000 17.92504296
-0.05291770 -0.70208822 0.00000000 87.77632855
0.05291770 -0.70208822 0.00000000 87.75758922
0.00000000 -0.75500592 0.00000000 34.17575783
0.00000000 -0.64917052 0.00000000 37.28691504
2.05800137 0.04406596 0.00000000 -35.46836777
2.16383676 0.04406596 0.00000000 -28.72611478
2.11091906 -0.00885174 0.00000000 -110.47682429
2.11091906 0.09698366 0.00000000 -113.36244895
1.27232327 1.15754064 0.00000000 95.73565247
1.37815866 1.15754064 0.00000000 84.08127770
1.32524096 1.10462294 0.00000000 3.88432437
1.32524096 1.21045834 0.00000000 2.43721338
-0.09470669 0.70146698 0.00000000 1.21884680
0.01112871 0.70146698 0.00000000 -4.84260357
-0.04178899 0.64854929 0.00000000 -117.83013426
-0.04178899 0.75438469 0.00000000 -110.98354423
-1.21473924 -1.48163476 0.00000000 -28.55088893
-1.10890384 -1.48163476 0.00000000 -30.91579166
-1.16182154 -1.53455246 0.00000000 -21.28443748
-1.16182154 -1.42871706 0.00000000 -27.94591052
-2.43005336 -0.81881509 0.00000000 12.47308792
-2.32421796 -0.81881509 0.00000000 16.15974802
-2.37713566 -0.87173279 0.00000000 4.69167346
-2.37713566 -0.76589740 0.00000000 5.39751559
-2.49323545 0.59060577 0.00000000 8.57312308
-2.38740004 0.59060577 0.00000000 8.49089232
-2.44031774 0.53768808 0.00000000 -5.99875303
-2.44031774 0.64352347 0.00000000 -4.33945845
-1.33902835 1.35334636 0.00000000 5.59981652
-1.23319294 1.35334636 0.00000000 6.91494294
-1.28611065 1.30042866 0.00000000 -15.87187336
-1.28611065 1.40626406 0.00000000 -13.71092467
45 36751 # 272.1 nm, (r2v6r1) 45 = 1Lb_v6 state
0.06478505 0.06220829 0.00000000 1.0 # electr. mom.
0.00000000 0.00000000 0.6618344880E-01 # magnet. mom.
1.31876649 -1.08237463 0.00000000 -40.30269491
0.00000000 -0.70208822 0.00000000 -67.02870344
2.11091906 0.04406596 0.00000000 78.44835918
1.32524096 1.15754064 0.00000000 -50.48370380
-0.04178899 0.70146698 0.00000000 62.96554670
-1.16182154 -1.48163476 0.00000000 29.42849248

-2.37713566	-0.81881509	0.00000000	-10.44434530
-2.44031774	0.59060577	0.00000000	-1.88348760
-1.28611065	1.35334636	0.00000000	4.68939702
1.26584879	-1.08237463	0.00000000	16.12094879
1.37168419	-1.08237463	0.00000000	14.74850768
1.31876649	-1.13529233	0.00000000	4.57668169
1.31876649	-1.02945693	0.00000000	4.88864808
-0.05291770	-0.70208822	0.00000000	23.93899869
0.05291770	-0.70208822	0.00000000	23.93388797
0.00000000	-0.75500592	0.00000000	9.32066123
0.00000000	-0.64917052	0.00000000	10.16915865
2.05800137	0.04406596	0.00000000	-9.67319121
2.16383676	0.04406596	0.00000000	-7.83439494
2.11091906	-0.00885174	0.00000000	-30.13004299
2.11091906	0.09698366	0.00000000	-30.91703153
1.27232327	1.15754064	0.00000000	26.10972340
1.37815866	1.15754064	0.00000000	22.93125755
1.32524096	1.10462294	0.00000000	1.05936119
1.32524096	1.21045834	0.00000000	0.66469456
-0.09470669	0.70146698	0.00000000	0.33241276
0.01112871	0.70146698	0.00000000	-1.32071006
-0.04178899	0.64854929	0.00000000	-32.13549116
-0.04178899	0.75438469	0.00000000	-30.26823933
-1.21473924	-1.48163476	0.00000000	-7.78660607
-1.10890384	-1.48163476	0.00000000	-8.43157954
-1.16182154	-1.53455246	0.00000000	-5.80484658
-1.16182154	-1.42871706	0.00000000	-7.62161196
-2.43005336	-0.81881509	0.00000000	3.40175125
-2.32421796	-0.81881509	0.00000000	4.40720400
-2.37713566	-0.87173279	0.00000000	1.27954731
-2.37713566	-0.76589740	0.00000000	1.47204971
-2.49323545	0.59060577	0.00000000	2.33812448
-2.38740004	0.59060577	0.00000000	2.31569791
-2.44031774	0.53768808	0.00000000	-1.63602355
-2.44031774	0.64352347	0.00000000	-1.18348867
-1.33902835	1.35334636	0.00000000	1.52722269
-1.23319294	1.35334636	0.00000000	1.88589353
-1.28611065	1.30042866	0.00000000	-4.32869273
-1.28611065	1.40626406	0.00000000	-3.73934309

45 36933 # 270.8 nm, (r2v7r1) 45 = 1Lb_v7 state

0.122372	0.117505	0.00000000 1.0	# electr. mom.
0.00000000	0.00000000	0.6618344880E-01	# magnet. mom.
1.31876649	-1.08237463	0.00000000	-76.12731260
0.00000000	-0.70208822	0.00000000	-126.60977316
2.11091906	0.04406596	0.00000000	148.18023401
1.32524096	1.15754064	0.00000000	-95.35810718
-0.04178899	0.70146698	0.00000000	118.93492154
-1.16182154	-1.48163476	0.00000000	55.58715246
-2.37713566	-0.81881509	0.00000000	-19.72820778
-2.44031774	0.59060577	0.00000000	-3.55769881
-1.28611065	1.35334636	0.00000000	8.85774992
1.26584879	-1.08237463	0.00000000	30.45068104
1.37168419	-1.08237463	0.00000000	27.85829228
1.31876649	-1.13529233	0.00000000	8.64484319
1.31876649	-1.02945693	0.00000000	9.23411304
-0.05291770	-0.70208822	0.00000000	45.21810864
0.05291770	-0.70208822	0.00000000	45.20845506
0.00000000	-0.75500592	0.00000000	17.60569343
0.00000000	-0.64917052	0.00000000	19.20841078
2.05800137	0.04406596	0.00000000	-18.27158340
2.16383676	0.04406596	0.00000000	-14.79830155
2.11091906	-0.00885174	0.00000000	-56.91230342
2.11091906	0.09698366	0.00000000	-58.39883734
1.27232327	1.15754064	0.00000000	49.31836642
1.37815866	1.15754064	0.00000000	43.31459760
1.32524096	1.10462294	0.00000000	2.00101558
1.32524096	1.21045834	0.00000000	1.25553417
-0.09470669	0.70146698	0.00000000	0.62789078
0.01112871	0.70146698	0.00000000	-2.49467456
-0.04178899	0.64854929	0.00000000	-60.70037219
-0.04178899	0.75438469	0.00000000	-57.17334096
-1.21473924	-1.48163476	0.00000000	-14.70803369
-1.10890384	-1.48163476	0.00000000	-15.92631692
-1.16182154	-1.53455246	0.00000000	-10.96471021
-1.16182154	-1.42871706	0.00000000	-14.39637815
-2.43005336	-0.81881509	0.00000000	6.42553014
-2.32421796	-0.81881509	0.00000000	8.32471867
-2.37713566	-0.87173279	0.00000000	2.41692269
-2.37713566	-0.76589740	0.00000000	2.78053834
-2.49323545	0.59060577	0.00000000	4.41645735
-2.38740004	0.59060577	0.00000000	4.37409605
-2.44031774	0.53768808	0.00000000	-3.09026671
-2.44031774	0.64352347	0.00000000	-2.23547859
-1.33902835	1.35334636	0.00000000	2.88475396
-1.23319294	1.35334636	0.00000000	3.56224333
-1.28611065	1.30042866	0.00000000	-8.17641961
-1.28611065	1.40626406	0.00000000	-7.06320362

```
45    35088    # 285.0 nm,    (r3v1r1) 45 = 1La_v1 state
   -0.62355          0.698522         0.00000000 1.0      # electr. mom.
    0.00000000       0.00000000       0.9507726948E-01    # magnet. mom.
    1.31876649      -1.08237463       0.00000000       39.59439777
    0.00000000      -0.70208822       0.00000000       10.85405556
    2.11091906       0.04406596       0.00000000     -239.74808547
    1.32524096       1.15754064       0.00000000       67.06770967
   -0.04178899       0.70146698       0.00000000     -245.46820460
   -1.16182154      -1.48163476       0.00000000      -10.51080187
   -2.37713566      -0.81881509       0.00000000      -40.06565998
   -2.44031774       0.59060577       0.00000000      -70.82267074
   -1.28611065       1.35334636       0.00000000      239.90449604
    1.26584879      -1.08237463       0.00000000      -44.49138627
    1.37168419      -1.08237463       0.00000000      -39.78676751
    1.31876649      -1.13529233       0.00000000       21.45537430
    1.31876649      -1.02945693       0.00000000       23.11584854
   -0.05291770      -0.70208822       0.00000000      -10.28323534
    0.05291770      -0.70208822       0.00000000      -18.59177079
    0.00000000      -0.75500592       0.00000000       11.99426129
    0.00000000      -0.64917052       0.00000000        6.39082161
    2.05800137       0.04406596       0.00000000       54.60740616
    2.16383676       0.04406596       0.00000000       52.67722360
    2.11091906      -0.00885174       0.00000000       66.12359676
    2.11091906       0.09698366       0.00000000       66.04296670
    1.27232327       1.15754064       0.00000000      -42.44981669
    1.37815866       1.15754064       0.00000000      -37.90799347
    1.32524096       1.10462294       0.00000000        7.68721283
    1.32524096       1.21045834       0.00000000        5.42911142
   -0.09470669       0.70146698       0.00000000      109.44907775
    0.01112871       0.70146698       0.00000000      111.85325610
   -0.04178899       0.64854929       0.00000000        9.05937962
   -0.04178899       0.75438469       0.00000000       15.92633020
   -1.21473924      -1.48163476       0.00000000      -11.30629578
   -1.10890384      -1.48163476       0.00000000      -10.74419829
   -1.16182154      -1.53455246       0.00000000       16.30644405
   -1.16182154      -1.42871706       0.00000000       15.94736208
   -2.43005336      -0.81881509       0.00000000      -42.28201185
   -2.32421796      -0.81881509       0.00000000      -49.73694577
   -2.37713566      -0.87173279       0.00000000       60.41085401
   -2.37713566      -0.76589740       0.00000000       72.14151242
   -2.49323545       0.59060577       0.00000000        8.70453522
   -2.38740004       0.59060577       0.00000000       11.33115727
   -2.44031774       0.53768808       0.00000000       23.16615646
   -2.44031774       0.64352347       0.00000000       27.44921087
   -1.33902835       1.35334636       0.00000000      -86.60118714
   -1.23319294       1.35334636       0.00000000      -83.85325886
   -1.28611065       1.30042866       0.00000000      -39.15929801
   -1.28611065       1.40626406       0.00000000      -30.88016983
45    35872    # 278.8 nm,    (r3v2r1) 45 = 1La_v2 state
   -0.57979          0.649503         0.00000000 1.0      # electr. mom.
    0.00000000       0.00000000       0.9507726948E-01    # magnet. mom.
    1.31876649      -1.08237463       0.00000000       36.81584354
    0.00000000      -0.70208822       0.00000000       10.09236745
    2.11091906       0.04406596       0.00000000     -222.92365842
    1.32524096       1.15754064       0.00000000       62.36120373
   -0.04178899       0.70146698       0.00000000     -228.24236568
   -1.16182154      -1.48163476       0.00000000       -9.77320174
   -2.37713566      -0.81881509       0.00000000      -37.25403472
   -2.44031774       0.59060577       0.00000000      -65.85265876
   -1.28611065       1.35334636       0.00000000      223.06909281
    1.26584879      -1.08237463       0.00000000      -41.36918372
    1.37168419      -1.08237463       0.00000000      -36.99471365
    1.31876649      -1.13529233       0.00000000       19.94973400
    1.31876649      -1.02945693       0.00000000       21.49368373
   -0.05291770      -0.70208822       0.00000000       -9.56160479
    0.05291770      -0.70208822       0.00000000      -17.28708512
    0.00000000      -0.75500592       0.00000000       11.15255874
    0.00000000      -0.64917052       0.00000000        5.94234290
    2.05800137       0.04406596       0.00000000       50.77530748
    2.16383676       0.04406596       0.00000000       48.98057633
    2.11091906      -0.00885174       0.00000000       61.48334435
    2.11091906       0.09698366       0.00000000       61.40837255
    1.27232327       1.15754064       0.00000000      -39.47088219
    1.37815866       1.15754064       0.00000000      -35.24778340
    1.32524096       1.10462294       0.00000000        7.14775929
    1.32524096       1.21045834       0.00000000        5.04812115
   -0.09470669       0.70146698       0.00000000      101.76844071
    0.01112871       0.70146698       0.00000000      104.00390480
   -0.04178899       0.64854929       0.00000000        8.42363368
   -0.04178899       0.75438469       0.00000000       14.80869300
   -1.21473924      -1.48163476       0.00000000      -10.51287152
   -1.10890384      -1.48163476       0.00000000       -9.99021947
   -1.16182154      -1.53455246       0.00000000       15.16213219
   -1.16182154      -1.42871706       0.00000000       14.82824895
   -2.43005336      -0.81881509       0.00000000      -39.31485312
   -2.32421796      -0.81881509       0.00000000      -46.24663379
   -2.37713566      -0.87173279       0.00000000       56.17149583
```

-2.37713566 -0.76589740 0.00000000 67.07895015
-2.49323545 0.59060577 0.00000000 8.09369064
-2.38740004 0.59060577 0.00000000 10.53598834
-2.44031774 0.53768808 0.00000000 21.54046127
-2.44031774 0.64352347 0.00000000 25.52295045
-1.33902835 1.35334636 0.00000000 -80.52391085
-1.23319294 1.35334636 0.00000000 -77.96881964
-1.28611065 1.30042866 0.00000000 -36.41127709
-1.28611065 1.40626406 0.00000000 -28.71314037
45 36135 # 276.7 nm, (r3v3r1) 45 = 1La_v3 state
-0.35006 0.392153 0.00000000 1.0 # electr. mom.
0.00000000 0.00000000 0.9507726948E-01 # magnet. mom.
1.31876649 -1.08237463 0.00000000 22.22843383
0.00000000 -0.70208822 0.00000000 6.09350488
2.11091906 0.04406596 0.00000000 -134.59541640
1.32524096 1.15754064 0.00000000 37.65204753
-0.04178899 0.70146698 0.00000000 -137.80671136
-1.16182154 -1.48163476 0.00000000 -5.90080105
-2.37713566 -0.81881509 0.00000000 -22.49300209
-2.44031774 0.59060577 0.00000000 -39.76009585
-1.28611065 1.35334636 0.00000000 134.68322585
1.26584879 -1.08237463 0.00000000 -24.97762036
1.37168419 -1.08237463 0.00000000 -22.33643088
1.31876649 -1.13529233 0.00000000 12.04512241
1.31876649 -1.02945693 0.00000000 12.97731848
-0.05291770 -0.70208822 0.00000000 -5.77304440
0.05291770 -0.70208822 0.00000000 -10.43748535
0.00000000 -0.75500592 0.00000000 6.73362037
0.00000000 -0.64917052 0.00000000 3.58782967
2.05800137 0.04406596 0.00000000 30.65678942
2.16383676 0.04406596 0.00000000 29.57317816
2.11091906 -0.00885174 0.00000000 37.12201923
2.11091906 0.09698366 0.00000000 37.07675324
1.27232327 1.15754064 0.00000000 -23.83147604
1.37815866 1.15754064 0.00000000 -21.28168054
1.32524096 1.10462294 0.00000000 4.31562825
1.32524096 1.21045834 0.00000000 3.04792220
-0.09470669 0.70146698 0.00000000 61.44509628
0.01112871 0.70146698 0.00000000 62.79481044
-0.04178899 0.64854929 0.00000000 5.08596750
-0.04178899 0.75438469 0.00000000 8.94109766
-1.21473924 -1.48163476 0.00000000 -6.34739412
-1.10890384 -1.48163476 0.00000000 -6.03183062
-1.16182154 -1.53455246 0.00000000 9.15449491
-1.16182154 -1.42871706 0.00000000 8.95290503
-2.43005336 -0.81881509 0.00000000 -23.73726981
-2.32421796 -0.81881509 0.00000000 -27.92249587
-2.37713566 -0.87173279 0.00000000 33.91486541
-2.37713566 -0.76589740 0.00000000 40.50049820
-2.49323545 0.59060577 0.00000000 4.88675661
-2.38740004 0.59060577 0.00000000 6.36135145
-2.44031774 0.53768808 0.00000000 13.00556152
-2.44031774 0.64352347 0.00000000 15.41008329
-1.33902835 1.35334636 0.00000000 -48.61821032
-1.23319294 1.35334636 0.00000000 -47.07551375
-1.28611065 1.30042866 0.00000000 -21.98416730
-1.28611065 1.40626406 0.00000000 -17.33623569
45 36656 # 272.8 nm, (r3v4r1) 45 = 1La_v4 state
-0.37194 0.416662 0.00000000 1.0 # electr. mom.
0.00000000 0.00000000 0.9507726948E-01 # magnet. mom.
1.31876649 -1.08237463 0.00000000 23.61771095
0.00000000 -0.70208822 0.00000000 6.47434893
2.11091906 0.04406596 0.00000000 -143.00762993
1.32524096 1.15754064 0.00000000 40.00530050
-0.04178899 0.70146698 0.00000000 -146.41963082
-1.16182154 -1.48163476 0.00000000 -6.26960112
-2.37713566 -0.81881509 0.00000000 -23.89881472
-2.44031774 0.59060577 0.00000000 -42.24510184
-1.28611065 1.35334636 0.00000000 143.10092747
1.26584879 -1.08237463 0.00000000 -26.53872163
1.37168419 -1.08237463 0.00000000 -23.73245781
1.31876649 -1.13529233 0.00000000 12.79794256
1.31876649 -1.02945693 0.00000000 13.78840088
-0.05291770 -0.70208822 0.00000000 -6.13385968
0.05291770 -0.70208822 0.00000000 -11.08982819
0.00000000 -0.75500592 0.00000000 7.15447164
0.00000000 -0.64917052 0.00000000 3.81206903
2.05800137 0.04406596 0.00000000 32.57283876
2.16383676 0.04406596 0.00000000 31.42150180
2.11091906 -0.00885174 0.00000000 39.44214543
2.11091906 0.09698366 0.00000000 39.39405031
1.27232327 1.15754064 0.00000000 -25.32094329
1.37815866 1.15754064 0.00000000 -22.61178558
1.32524096 1.10462294 0.00000000 4.58535502
1.32524096 1.21045834 0.00000000 3.23841734
-0.09470669 0.70146698 0.00000000 65.28541480
0.01112871 0.70146698 0.00000000 66.71948610

-0.04178899	0.64854929	0.00000000	5.40384047
-0.04178899	0.75438469	0.00000000	9.49991626
-1.21473924	-1.48163476	0.00000000	-6.74410626
-1.10890384	-1.48163476	0.00000000	-6.40882003
-1.16182154	-1.53455246	0.00000000	9.72665084
-1.16182154	-1.42871706	0.00000000	9.51246159
-2.43005336	-0.81881509	0.00000000	-25.22084917
-2.32421796	-0.81881509	0.00000000	-29.66765186
-2.37713566	-0.87173279	0.00000000	36.03454450
-2.37713566	-0.76589740	0.00000000	43.03177934
-2.49323545	0.59060577	0.00000000	5.19217890
-2.38740004	0.59060577	0.00000000	6.75893592
-2.44031774	0.53768808	0.00000000	13.81840912
-2.44031774	0.64352347	0.00000000	16.37321350
-1.33902835	1.35334636	0.00000000	-51.65684847
-1.23319294	1.35334636	0.00000000	-50.01773336
-1.28611065	1.30042866	0.00000000	-23.35817776
-1.28611065	1.40626406	0.00000000	-18.41975042

45 36919 # 270.9 nm, (r3v1r1) 45 = 1La_v5 state

-0.32818	0.367643	0.00000000 1.0	# electr. mom.
0.00000000	0.00000000	0.9507726948E-01	# magnet. mom.
1.31876649	-1.08237463	0.00000000	20.83915672
0.00000000	-0.70208822	0.00000000	5.71266082
2.11091906	0.04406596	0.00000000	-126.18320288
1.32524096	1.15754064	0.00000000	35.29879456
-0.04178899	0.70146698	0.00000000	-129.19379190
-1.16182154	-1.48163476	0.00000000	-5.53200098
-2.37713566	-0.81881509	0.00000000	-21.08718946
-2.44031774	0.59060577	0.00000000	-37.27508986
-1.28611065	1.35334636	0.00000000	126.26552423
1.26584879	-1.08237463	0.00000000	-23.41651909
1.37168419	-1.08237463	0.00000000	-20.94040395
1.31876649	-1.13529233	0.00000000	11.29230226
1.31876649	-1.02945693	0.00000000	12.16623607
-0.05291770	-0.70208822	0.00000000	-5.41222913
0.05291770	-0.70208822	0.00000000	-9.78514252
0.00000000	-0.75500592	0.00000000	6.31276910
0.00000000	-0.64917052	0.00000000	3.36359032
2.05800137	0.04406596	0.00000000	28.74074008
2.16383676	0.04406596	0.00000000	27.72485453
2.11091906	-0.00885174	0.00000000	34.80189303
2.11091906	0.09698366	0.00000000	34.75945616
1.27232327	1.15754064	0.00000000	-22.34200879
1.37815866	1.15754064	0.00000000	-19.95157551
1.32524096	1.10462294	0.00000000	4.04590149
1.32524096	1.21045834	0.00000000	2.85742706
-0.09470669	0.70146698	0.00000000	57.60477776
0.01112871	0.70146698	0.00000000	58.87013479
-0.04178899	0.64854929	0.00000000	4.76809454
-0.04178899	0.75438469	0.00000000	8.38227905
-1.21473924	-1.48163476	0.00000000	-5.95068199
-1.10890384	-1.48163476	0.00000000	-5.65484121
-1.16182154	-1.53455246	0.00000000	8.58233897
-1.16182154	-1.42871706	0.00000000	8.39334846
-2.43005336	-0.81881509	0.00000000	-22.25369045
-2.32421796	-0.81881509	0.00000000	-26.17733988
-2.37713566	-0.87173279	0.00000000	31.79518632
-2.37713566	-0.76589740	0.00000000	37.96921706
-2.49323545	0.59060577	0.00000000	4.58133433
-2.38740004	0.59060577	0.00000000	5.96376698
-2.44031774	0.53768808	0.00000000	12.19271393
-2.44031774	0.64352347	0.00000000	14.44695309
-1.33902835	1.35334636	0.00000000	-45.57957218
-1.23319294	1.35334636	0.00000000	-44.13329414
-1.28611065	1.30042866	0.00000000	-20.61015685
-1.28611065	1.40626406	0.00000000	-16.25272096

45 37440 # 267.1 nm, (r3v2r1) 45 = 1La_v6 state

-0.19691	0.220586	0.00000000 1.0	# electr. mom.
0.00000000	0.00000000	0.9507726948E-01	# magnet. mom.
1.31876649	-1.08237463	0.00000000	12.50349403
0.00000000	-0.70208822	0.00000000	3.42759649
2.11091906	0.04406596	0.00000000	-75.70992173
1.32524096	1.15754064	0.00000000	21.17927674
-0.04178899	0.70146698	0.00000000	-77.51627514
-1.16182154	-1.48163476	0.00000000	-3.31920059
-2.37713566	-0.81881509	0.00000000	-12.65231368
-2.44031774	0.59060577	0.00000000	-22.36505392
-1.28611065	1.35334636	0.00000000	75.75931454
1.26584879	-1.08237463	0.00000000	-14.04991145
1.37168419	-1.08237463	0.00000000	-12.56424237
1.31876649	-1.13529233	0.00000000	6.77538136
1.31876649	-1.02945693	0.00000000	7.29974164
-0.05291770	-0.70208822	0.00000000	-3.24733748
0.05291770	-0.70208822	0.00000000	-5.87108551
0.00000000	-0.75500592	0.00000000	3.78766146
0.00000000	-0.64917052	0.00000000	2.01815419
2.05800137	0.04406596	0.00000000	17.24444405

2.16383676 0.04406596 0.00000000 16.63491272
2.11091906 -0.00885174 0.00000000 20.88113582
2.11091906 0.09698366 0.00000000 20.85567369
1.27232327 1.15754064 0.00000000 -13.40520527
1.37815866 1.15754064 0.00000000 -11.97094531
1.32524096 1.10462294 0.00000000 2.42754089
1.32524096 1.21045834 0.00000000 1.71445624
-0.09470669 0.70146698 0.00000000 34.56286666
0.01112871 0.70146698 0.00000000 35.32208088
-0.04178899 0.64854929 0.00000000 2.86085672
-0.04178899 0.75438469 0.00000000 5.02936743
-1.21473924 -1.48163476 0.00000000 -3.57040920
-1.10890384 -1.48163476 0.00000000 -3.39290472
-1.16182154 -1.53455246 0.00000000 5.14940338
-1.16182154 -1.42871706 0.00000000 5.03600908
-2.43005336 -0.81881509 0.00000000 -13.35221427
-2.32421796 -0.81881509 0.00000000 -15.70640393
-2.37713566 -0.87173279 0.00000000 19.07711179
-2.37713566 -0.76589740 0.00000000 22.78153024
-2.49323545 0.59060577 0.00000000 2.74880060
-2.38740004 0.59060577 0.00000000 3.57826019
-2.44031774 0.53768808 0.00000000 7.31562836
-2.44031774 0.64352347 0.00000000 8.66817185
-1.33902835 1.35334636 0.00000000 -27.34774331
-1.23319294 1.35334636 0.00000000 -26.47997648
-1.28611065 1.30042866 0.00000000 -12.36609411
-1.28611065 1.40626406 0.00000000 -9.75163258
45 37966 # 263.4 nm, (r3v3r1) 45 = 1La_v7 state
-0.20785 0.232841 0.00000000 1.0 # electr. mom.
0.00000000 0.00000000 0.9507726948E-01 # magnet. mom.
1.31876649 -1.08237463 0.00000000 13.19813259
0.00000000 -0.70208822 0.00000000 3.61801852
2.11091906 0.04406596 0.00000000 -79.91602849
1.32524096 1.15754064 0.00000000 22.35590322
-0.04178899 0.70146698 0.00000000 -81.82273487
-1.16182154 -1.48163476 0.00000000 -3.50360062
-2.37713566 -0.81881509 0.00000000 -13.35521999
-2.44031774 0.59060577 0.00000000 -23.60755691
-1.28611065 1.35334636 0.00000000 79.96816535
1.26584879 -1.08237463 0.00000000 -14.83046209
1.37168419 -1.08237463 0.00000000 -13.26225584
1.31876649 -1.13529233 0.00000000 7.15179143
1.31876649 -1.02945693 0.00000000 7.70528285
-0.05291770 -0.70208822 0.00000000 -3.42774511
0.05291770 -0.70208822 0.00000000 -6.19725693
0.00000000 -0.75500592 0.00000000 3.99808710
0.00000000 -0.64917052 0.00000000 2.13027387
2.05800137 0.04406596 0.00000000 18.20246872
2.16383676 0.04406596 0.00000000 17.55907453
2.11091906 -0.00885174 0.00000000 22.04119892
2.11091906 0.09698366 0.00000000 22.01432223
1.27232327 1.15754064 0.00000000 -14.14993890
1.37815866 1.15754064 0.00000000 -12.63599782
1.32524096 1.10462294 0.00000000 2.56240428
1.32524096 1.21045834 0.00000000 1.80970381
-0.09470669 0.70146698 0.00000000 36.48302592
0.01112871 0.70146698 0.00000000 37.28441870
-0.04178899 0.64854929 0.00000000 3.01979321
-0.04178899 0.75438469 0.00000000 5.30877673
-1.21473924 -1.48163476 0.00000000 -3.76876526
-1.10890384 -1.48163476 0.00000000 -3.58139943
-1.16182154 -1.53455246 0.00000000 5.43548135
-1.16182154 -1.42871706 0.00000000 5.31578736
-2.43005336 -0.81881509 0.00000000 -14.09400395
-2.32421796 -0.81881509 0.00000000 -16.57898192
-2.37713566 -0.87173279 0.00000000 20.13695134
-2.37713566 -0.76589740 0.00000000 24.04717081
-2.49323545 0.59060577 0.00000000 2.90151174
-2.38740004 0.59060577 0.00000000 3.77705242
-2.44031774 0.53768808 0.00000000 7.72205215
-2.44031774 0.64352347 0.00000000 9.14973696
-1.33902835 1.35334636 0.00000000 -28.86706238
-1.23319294 1.35334636 0.00000000 -27.95108629
-1.28611065 1.30042866 0.00000000 -13.05309934
-1.28611065 1.40626406 0.00000000 -10.29338994
45 38224 # 261.6 nm, (r3v4r1) 45 = 1La_v8 state
-0.08752 0.087038 0.00000000 1.0 # electr. mom.
0.00000000 0.00000000 0.9507726948E-01 # magnet. mom.
1.31876649 -1.08237463 0.00000000 5.55710846
0.00000000 -0.70208822 0.00000000 1.52337622
2.11091906 0.04406596 0.00000000 -33.64885410
1.32524096 1.15754064 0.00000000 9.41301188
-0.04178899 0.70146698 0.00000000 -34.45167784
-1.16182154 -1.48163476 0.00000000 -1.47520026
-2.37713566 -0.81881509 0.00000000 -5.62325052
-2.44031774 0.59060577 0.00000000 -9.94002396
-1.28611065 1.35334636 0.00000000 33.67080646

```
     1.26584879     -1.08237463     0.00000000      -6.24440509
     1.37168419     -1.08237463     0.00000000      -5.58410772
     1.31876649     -1.13529233     0.00000000       3.01128060
     1.31876649     -1.02945693     0.00000000       3.24432962
    -0.05291770     -0.70208822     0.00000000      -1.44326110
     0.05291770     -0.70208822     0.00000000      -2.60937134
     0.00000000     -0.75500592     0.00000000       1.68340509
     0.00000000     -0.64917052     0.00000000       0.89695742
     2.05800137      0.04406596     0.00000000       7.66419736
     2.16383676      0.04406596     0.00000000       7.39329454
     2.11091906     -0.00885174     0.00000000       9.28050481
     2.11091906      0.09698366     0.00000000       9.26918831
     1.27232327      1.15754064     0.00000000      -5.95786901
     1.37815866      1.15754064     0.00000000      -5.32042014
     1.32524096      1.10462294     0.00000000       1.07890706
     1.32524096      1.21045834     0.00000000       0.76198055
    -0.09470669      0.70146698     0.00000000      15.36127407
     0.01112871      0.70146698     0.00000000      15.69870261
    -0.04178899      0.64854929     0.00000000       1.27149188
    -0.04178899      0.75438469     0.00000000       2.23527441
    -1.21473924     -1.48163476     0.00000000      -1.58684853
    -1.10890384     -1.48163476     0.00000000      -1.50795766
    -1.16182154     -1.53455246     0.00000000       2.28862373
    -1.16182154     -1.42871706     0.00000000       2.23822626
    -2.43005336     -0.81881509     0.00000000      -5.93431745
    -2.32421796     -0.81881509     0.00000000      -6.98062397
    -2.37713566     -0.87173279     0.00000000       8.47871635
    -2.37713566     -0.76589740     0.00000000      10.12512455
    -2.49323545      0.59060577     0.00000000       1.22168915
    -2.38740004      0.59060577     0.00000000       1.59033786
    -2.44031774      0.53768808     0.00000000       3.25139038
    -2.44031774      0.64352347     0.00000000       3.85252082
    -1.33902835      1.35334636     0.00000000     -12.15455258
    -1.23319294      1.35334636     0.00000000     -11.76887844
    -1.28611065      1.30042866     0.00000000      -5.49604183
    -1.28611065      1.40626406     0.00000000      -4.33405892
45    39013    # 256.3 nm,    (r3v4r1) 45 = 1La_v9 state
    -0.10939         0.122548         0.00000000 1.0      # electr. mom.
     0.00000000      0.00000000       0.9507726948E-01 # magnet. mom.
     1.31876649     -1.08237463       0.00000000       6.94638557
     0.00000000     -0.70208822       0.00000000       1.90422027
     2.11091906      0.04406596       0.00000000     -42.06106763
     1.32524096      1.15754064       0.00000000      11.76626485
    -0.04178899      0.70146698       0.00000000     -43.06459730
    -1.16182154     -1.48163476       0.00000000      -1.84400033
    -2.37713566     -0.81881509       0.00000000      -7.02906315
    -2.44031774      0.59060577       0.00000000     -12.42502995
    -1.28611065      1.35334636       0.00000000      42.08850808
     1.26584879     -1.08237463       0.00000000      -7.80550636
     1.37168419     -1.08237463       0.00000000      -6.98013465
     1.31876649     -1.13529233       0.00000000       3.76410075
     1.31876649     -1.02945693       0.00000000       4.05541202
    -0.05291770     -0.70208822       0.00000000      -1.80407638
     0.05291770     -0.70208822       0.00000000      -3.26171417
     0.00000000     -0.75500592       0.00000000       2.10425637
     0.00000000     -0.64917052       0.00000000       1.12119677
     2.05800137      0.04406596       0.00000000       9.58024669
     2.16383676      0.04406596       0.00000000       9.24161818
     2.11091906     -0.00885174       0.00000000      11.60063101
     2.11091906      0.09698366       0.00000000      11.58648539
     1.27232327      1.15754064       0.00000000      -7.44733626
     1.37815866      1.15754064       0.00000000      -6.65052517
     1.32524096      1.10462294       0.00000000       1.34863383
     1.32524096      1.21045834       0.00000000       0.95247569
    -0.09470669      0.70146698       0.00000000      19.20159259
     0.01112871      0.70146698       0.00000000      19.62337826
    -0.04178899      0.64854929       0.00000000       1.58936485
    -0.04178899      0.75438469       0.00000000       2.79409302
    -1.21473924     -1.48163476       0.00000000      -1.98356066
    -1.10890384     -1.48163476       0.00000000      -1.88494707
    -1.16182154     -1.53455246       0.00000000       2.86077966
    -1.16182154     -1.42871706       0.00000000       2.79778282
    -2.43005336     -0.81881509       0.00000000      -7.41789682
    -2.32421796     -0.81881509       0.00000000      -8.72577996
    -2.37713566     -0.87173279       0.00000000      10.59839544
    -2.37713566     -0.76589740       0.00000000      12.65640569
    -2.49323545      0.59060577       0.00000000       1.52711144
    -2.38740004      0.59060577       0.00000000       1.98792233
    -2.44031774      0.53768808       0.00000000       4.06423798
    -2.44031774      0.64352347       0.00000000       4.81565103
    -1.33902835      1.35334636       0.00000000     -15.19319073
    -1.23319294      1.35334636       0.00000000     -14.71109805
    -1.28611065      1.30042866       0.00000000      -6.87005228
    -1.28611065      1.40626406       0.00000000      -5.41757365
45    48951.0    # 204.3 nm,    (r7r1) 45 = 1Bb state
     3.63207311     -2.46606417       0.00000000 1.0      # electr. mom.
     0.00000000      0.00000000       0.8170710246E-01 # magnet. mom.
```

1.31876649	-1.08237463	0.00000000	273.58236816
0.00000000	-0.70208822	0.00000000	-265.65930610
2.11091906	0.04406596	0.00000000	-115.47421142
1.32524096	1.15754064	0.00000000	0.23796456
-0.04178899	0.70146698	0.00000000	-361.15542547
-1.16182154	-1.48163476	0.00000000	-60.38212469
-2.37713566	-0.81881509	0.00000000	54.11125387
-2.44031774	0.59060577	0.00000000	-40.47837144
-1.28611065	1.35334636	0.00000000	-176.05929730
1.26584879	-1.08237463	0.00000000	-120.18342346
1.37168419	-1.08237463	0.00000000	-112.55475106
1.31876649	-1.13529233	0.00000000	-16.55443680
1.31876649	-1.02945693	0.00000000	-27.40340530
-0.05291770	-0.70208822	0.00000000	-15.24288341
0.05291770	-0.70208822	0.00000000	21.41905675
0.00000000	-0.75500592	0.00000000	121.86345384
0.00000000	-0.64917052	0.00000000	141.63808133
2.05800137	0.04406596	0.00000000	-165.43904117
2.16383676	0.04406596	0.00000000	-146.56078618
2.11091906	-0.00885174	0.00000000	235.05330168
2.11091906	0.09698366	0.00000000	193.56262186
1.27232327	1.15754064	0.00000000	-72.11298134
1.37815866	1.15754064	0.00000000	-66.98146392
1.32524096	1.10462294	0.00000000	76.18560024
1.32524096	1.21045834	0.00000000	63.46618186
-0.09470669	0.70146698	0.00000000	231.75718234
0.01112871	0.70146698	0.00000000	209.07036739
-0.04178899	0.64854929	0.00000000	-58.60513608
-0.04178899	0.75438469	0.00000000	-22.60532597
-1.21473924	-1.48163476	0.00000000	3.82053778
-1.10890384	-1.48163476	0.00000000	-0.67954829
-1.16182154	-1.53455246	0.00000000	23.40262949
-1.16182154	-1.42871706	0.00000000	34.96314259
-2.43005336	-0.81881509	0.00000000	-13.30868530
-2.32421796	-0.81881509	0.00000000	-20.66109768
-2.37713566	-0.87173279	0.00000000	-9.86099971
-2.37713566	-0.76589740	0.00000000	-11.01732744
-2.49323545	0.59060577	0.00000000	22.32187454
-2.38740004	0.59060577	0.00000000	24.65745350
-2.44031774	0.53768808	0.00000000	-3.42862325
-2.44031774	0.64352347	0.00000000	-2.95631741
-1.33902835	1.35334636	0.00000000	57.77673394
-1.23319294	1.35334636	0.00000000	54.19642608
-1.28611065	1.30042866	0.00000000	26.87626915
-1.28611065	1.40626406	0.00000000	35.40246936

45 51032.0 # 196.0 nm, (r8r1) 45 = 1Ba state

3.62907333	2.12605217	0.00000000 1.0 # electr. mom.	
0.00000000	0.00000000	0.7736875962	# magnet. mom.
1.31876649	-1.08237463	0.00000000	41.08026403
0.00000000	-0.70208822	0.00000000	-343.80311510
2.11091906	0.04406596	0.00000000	524.75399578
1.32524096	1.15754064	0.00000000	-141.34580429
-0.04178899	0.70146698	0.00000000	320.58709099
-1.16182154	-1.48163476	0.00000000	-208.87443946
-2.37713566	-0.81881509	0.00000000	-375.25407322
-2.44031774	0.59060577	0.00000000	-11.29418962
-1.28611065	1.35334636	0.00000000	232.68651542
1.26584879	-1.08237463	0.00000000	-3.15269424
1.37168419	-1.08237463	0.00000000	1.42337698
1.31876649	-1.13529233	0.00000000	-18.07970088
1.31876649	-1.02945693	0.00000000	-21.54373656
-0.05291770	-0.70208822	0.00000000	258.42064886
0.05291770	-0.70208822	0.00000000	265.34171789
0.00000000	-0.75500592	0.00000000	-78.67097654
0.00000000	-0.64917052	0.00000000	-102.30132562
2.05800137	0.04406596	0.00000000	-137.57776229
2.16383676	0.04406596	0.00000000	-124.76790816
2.11091906	-0.00885174	0.00000000	-132.17957971
2.11091906	0.09698366	0.00000000	-130.18179466
1.27232327	1.15754064	0.00000000	11.94680750
1.37815866	1.15754064	0.00000000	14.20895232
1.32524096	1.10462294	0.00000000	63.68987227
1.32524096	1.21045834	0.00000000	52.27207022
-0.09470669	0.70146698	0.00000000	-254.72888904
0.01112871	0.70146698	0.00000000	-247.66321642
-0.04178899	0.64854929	0.00000000	100.91234117
-0.04178899	0.75438469	0.00000000	81.26213131
-1.21473924	-1.48163476	0.00000000	63.19609210
-1.10890384	-1.48163476	0.00000000	67.34083464
-1.16182154	-1.53455246	0.00000000	38.93285534
-1.16182154	-1.42871706	0.00000000	38.80641912
-2.43005336	-0.81881509	0.00000000	41.08805155
-2.32421796	-0.81881509	0.00000000	39.60116971
-2.37713566	-0.87173279	0.00000000	138.02162731
-2.37713566	-0.76589740	0.00000000	157.11609917
-2.49323545	0.59060577	0.00000000	11.11832798
-2.38740004	0.59060577	0.00000000	8.46978019

```
      -2.44031774        0.53768808        0.00000000      -11.23878984
      -2.44031774        0.64352347        0.00000000        1.30995821
      -1.33902835        1.35334636        0.00000000      -88.14937474
      -1.23319294        1.35334636        0.00000000      -87.46272826
      -1.28611065        1.30042866        0.00000000      -22.34592149
      -1.28611065        1.40626406        0.00000000      -32.97098002
&TRANSITION 2->...
 0     0.0    # (r2v2r2v1) 45
      0.00000000       0.00000000       0.00000000 1.0       # electr. mom.
      0.00000000       0.00000000       0.00000000            # magnet. mom.
 0     0.0    # (r2v3r2v1) 45
      0.00000000       0.00000000       0.00000000 1.0       # electr. mom.
      0.00000000       0.00000000       0.00000000            # magnet. mom.
 0     0.0    # (r2v4r2v1) 45
      0.00000000       0.00000000       0.00000000 1.0       # electr. mom.
      0.00000000       0.00000000       0.00000000            # magnet. mom.
 0     0.0    # (r2v5r2v1) 45
      0.00000000       0.00000000       0.00000000 1.0       # electr. mom.
      0.00000000       0.00000000       0.00000000            # magnet. mom.
 0     0.0    # (r2v6r2v1) 45
      0.00000000       0.00000000       0.00000000 1.0       # electr. mom.
      0.00000000       0.00000000       0.00000000            # magnet. mom.
 0     0.0    # (r2v7r2v1) 45
      0.00000000       0.00000000       0.00000000 1.0       # electr. mom.
      0.00000000       0.00000000       0.00000000            # magnet. mom.
 45    2657.0    # 737.7 nm,    (r3v1r2v1) 45
      0.133678         0.254709         0.00000000 1.0     # electr. mom.
      0.00000000       0.00000000      -1.664952771         # magnet. mom.
      1.31876649      -1.08237463       0.00000000      -400.58815753
      0.00000000      -0.70208822       0.00000000       742.49707019
      2.11091906       0.04406596       0.00000000       118.24790001
      1.32524096       1.15754064       0.00000000      -450.41464296
     -0.04178899       0.70146698       0.00000000       788.30948850
     -1.16182154      -1.48163476       0.00000000      -245.25217239
     -2.37713566      -0.81881509       0.00000000        38.43347860
     -2.44031774       0.59060577       0.00000000        37.17212963
     -1.28611065       1.35334636       0.00000000      -439.38764308
      1.26584879      -1.08237463       0.00000000       102.68215677
      1.37168419      -1.08237463       0.00000000        88.16474334
      1.31876649      -1.13529233       0.00000000        95.71141240
      1.31876649      -1.02945693       0.00000000       116.69708925
     -0.05291770      -0.70208822       0.00000000      -180.59242258
      0.05291770      -0.70208822       0.00000000      -188.06104126
      0.00000000      -0.75500592       0.00000000      -189.81981270
      0.00000000      -0.64917052       0.00000000      -188.00211461
      2.05800137       0.04406596       0.00000000       -34.95118913
      2.16383676       0.04406596       0.00000000       -17.66341430
      2.11091906      -0.00885174       0.00000000       -30.96074422
      2.11091906       0.09698366       0.00000000       -36.79812556
      1.27232327       1.15754064       0.00000000       133.59851462
      1.37815866       1.15754064       0.00000000       118.33135476
      1.32524096       1.10462294       0.00000000       113.96373062
      1.32524096       1.21045834       0.00000000        88.10090515
     -0.09470669       0.70146698       0.00000000      -377.53681829
      0.01112871       0.70146698       0.00000000      -382.91017511
     -0.04178899       0.64854929       0.00000000        -3.59985621
     -0.04178899       0.75438469       0.00000000       -27.55377330
     -1.21473924      -1.48163476       0.00000000        77.93346901
     -1.10890384      -1.48163476       0.00000000        86.46891455
     -1.16182154      -1.53455246       0.00000000        36.63881285
     -1.16182154      -1.42871706       0.00000000        45.76091746
     -2.43005336      -0.81881509       0.00000000       -12.46217516
     -2.32421796      -0.81881509       0.00000000       -16.56422108
     -2.37713566      -0.87173279       0.00000000        -5.09089085
     -2.37713566      -0.76589740       0.00000000        -4.68551970
     -2.49323545       0.59060577       0.00000000       -29.25284975
     -2.38740004       0.59060577       0.00000000       -38.68013971
     -2.44031774       0.53768808       0.00000000        15.22333706
     -2.44031774       0.64352347       0.00000000        14.79157244
     -1.33902835       1.35334636       0.00000000        82.13914818
     -1.23319294       1.35334636       0.00000000        90.04681439
     -1.28611065       1.30042866       0.00000000       147.93525348
     -1.28611065       1.40626406       0.00000000       121.97968616
 45    2657.0    # 737.7 nm,    (r3v2r2v1) 45
      0.124297         0.236835         0.00000000 1.0     # electr. mom.
      0.00000000       0.00000000      -1.664952771         # magnet. mom.
      1.31876649      -1.08237463       0.00000000      -372.47670787
      0.00000000      -0.70208822       0.00000000       690.39201263
      2.11091906       0.04406596       0.00000000       109.94980176
      1.32524096       1.15754064       0.00000000      -418.80659784
     -0.04178899       0.70146698       0.00000000       732.98952439
     -1.16182154      -1.48163476       0.00000000      -228.04149362
     -2.37713566      -0.81881509       0.00000000        35.73639238
     -2.44031774       0.59060577       0.00000000        34.56355913
     -1.28611065       1.35334636       0.00000000      -408.55342251
      1.26584879      -1.08237463       0.00000000        95.47639138
      1.37168419      -1.08237463       0.00000000        81.97774381
```

```
 1.31876649   -1.13529233   0.00000000    88.99482206
 1.31876649   -1.02945693   0.00000000   108.50781983
-0.05291770   -0.70208822   0.00000000  -167.91927012
 0.05291770   -0.70208822   0.00000000  -174.86377520
 0.00000000   -0.75500592   0.00000000  -176.49912409
 0.00000000   -0.64917052   0.00000000  -174.80898376
 2.05800137    0.04406596   0.00000000   -32.49847411
 2.16383676    0.04406596   0.00000000   -16.42387645
 2.11091906   -0.00885174   0.00000000   -28.78806042
 2.11091906    0.09698366   0.00000000   -34.21580095
 1.27232327    1.15754064   0.00000000   124.22318026
 1.37815866    1.15754064   0.00000000   110.02740004
 1.32524096    1.10462294   0.00000000   105.96627584
 1.32524096    1.21045834   0.00000000    81.91838549
-0.09470669    0.70146698   0.00000000  -351.04300648
 0.01112871    0.70146698   0.00000000  -356.03928563
-0.04178899    0.64854929   0.00000000    -3.34723472
-0.04178899    0.75438469   0.00000000   -25.62017517
-1.21473924   -1.48163476   0.00000000    72.46445364
-1.10890384   -1.48163476   0.00000000    80.40092054
-1.16182154   -1.53455246   0.00000000    34.06766808
-1.16182154   -1.42871706   0.00000000    42.54962501
-2.43005336   -0.81881509   0.00000000   -11.58763656
-2.32421796   -0.81881509   0.00000000   -15.40181960
-2.37713566   -0.87173279   0.00000000    -4.73363536
-2.37713566   -0.76589740   0.00000000    -4.35671130
-2.49323545    0.59060577   0.00000000   -27.20001819
-2.38740004    0.59060577   0.00000000   -35.96574394
-2.44031774    0.53768808   0.00000000    14.15503270
-2.44031774    0.64352347   0.00000000    13.75356736
-1.33902835    1.35334636   0.00000000    76.37499743
-1.23319294    1.35334636   0.00000000    83.72773969
-1.28611065    1.30042866   0.00000000   137.55383218
-1.28611065    1.40626406   0.00000000   113.41970819
45    2657.0    # 737.7 nm,    (r3v3r2v1) 45
 0.075047      0.142994     0.00000000 1.0     # electr. mom.
 0.00000000    0.00000000  -1.664952771        # magnet. mom.
 1.31876649   -1.08237463   0.00000000  -224.89159721
 0.00000000   -0.70208822   0.00000000   416.84046046
 2.11091906    0.04406596   0.00000000    66.38478597
 1.32524096    1.15754064   0.00000000  -252.86436096
-0.04178899    0.70146698   0.00000000   442.55971284
-1.16182154   -1.48163476   0.00000000  -137.68543011
-2.37713566   -0.81881509   0.00000000    21.57668974
-2.44031774    0.59060577   0.00000000    20.86856400
-1.28611065    1.35334636   0.00000000  -246.67376454
 1.26584879   -1.08237463   0.00000000    57.64612310
 1.37168419   -1.08237463   0.00000000    49.49599626
 1.31876649   -1.13529233   0.00000000    53.73272275
 1.31876649   -1.02945693   0.00000000    65.51415537
-0.05291770   -0.70208822   0.00000000  -101.38521969
 0.05291770   -0.70208822   0.00000000  -105.57812842
 0.00000000   -0.75500592   0.00000000  -106.56550888
 0.00000000   -0.64917052   0.00000000  -105.54504680
 2.05800137    0.04406596   0.00000000   -19.62172021
 2.16383676    0.04406596   0.00000000    -9.91630276
 2.11091906   -0.00885174   0.00000000   -17.38147044
 2.11091906    0.09698366   0.00000000   -20.65859680
 1.27232327    1.15754064   0.00000000    75.00267487
 1.37815866    1.15754064   0.00000000    66.43163776
 1.32524096    1.10462294   0.00000000    63.97963824
 1.32524096    1.21045834   0.00000000    49.46015728
-0.09470669    0.70146698   0.00000000  -211.95049448
 0.01112871    0.70146698   0.00000000  -214.96711585
-0.04178899    0.64854929   0.00000000    -2.02097191
-0.04178899    0.75438469   0.00000000   -15.46878501
-1.21473924   -1.48163476   0.00000000    43.75212295
-1.10890384   -1.48163476   0.00000000    48.54395203
-1.16182154   -1.53455246   0.00000000    20.56915809
-1.16182154   -1.42871706   0.00000000    25.69033963
-2.43005336   -0.81881509   0.00000000    -6.99630886
-2.32421796   -0.81881509   0.00000000    -9.29921183
-2.37713566   -0.87173279   0.00000000    -2.85804399
-2.37713566   -0.76589740   0.00000000    -2.63046720
-2.49323545    0.59060577   0.00000000   -16.42265249
-2.38740004    0.59060577   0.00000000   -21.71516615
-2.44031774    0.53768808   0.00000000     8.54643484
-2.44031774    0.64352347   0.00000000     8.30404067
-1.33902835    1.35334636   0.00000000    46.11320599
-1.23319294    1.35334636   0.00000000    50.55259755
-1.28611065    1.30042866   0.00000000    83.05137037
-1.28611065    1.40626406   0.00000000    68.47982381
45    2657.0    # 737.7 nm,    (r3v4r2v1) 45
 0.079738      0.151932     0.00000000 1.0     # electr. mom.
 0.00000000    0.00000000  -1.664952771        # magnet. mom.
 1.31876649   -1.08237463   0.00000000  -238.94732203
 0.00000000   -0.70208822   0.00000000   442.89298923
```

2.11091906	0.04406596	0.00000000	70.53383509
1.32524096	1.15754064	0.00000000	-268.66838352
-0.04178899	0.70146698	0.00000000	470.21969489
-1.16182154	-1.48163476	0.00000000	-146.29076949
-2.37713566	-0.81881509	0.00000000	22.92523285
-2.44031774	0.59060577	0.00000000	22.17284925
-1.28611065	1.35334636	0.00000000	-262.09087482
1.26584879	-1.08237463	0.00000000	61.24900579
1.37168419	-1.08237463	0.00000000	52.58949603
1.31876649	-1.13529233	0.00000000	57.09101792
1.31876649	-1.02945693	0.00000000	69.60879008
-0.05291770	-0.70208822	0.00000000	-107.72179592
0.05291770	-0.70208822	0.00000000	-112.17676145
0.00000000	-0.75500592	0.00000000	-113.22585319
0.00000000	-0.64917052	0.00000000	-112.14161222
2.05800137	0.04406596	0.00000000	-20.84807773
2.16383676	0.04406596	0.00000000	-10.53607169
2.11091906	-0.00885174	0.00000000	-18.46781234
2.11091906	0.09698366	0.00000000	-21.94975910
1.27232327	1.15754064	0.00000000	79.69034205
1.37815866	1.15754064	0.00000000	70.58361512
1.32524096	1.10462294	0.00000000	67.97836563
1.32524096	1.21045834	0.00000000	52.55141711
-0.09470669	0.70146698	0.00000000	-225.19740039
0.01112871	0.70146698	0.00000000	-228.40256059
-0.04178899	0.64854929	0.00000000	-2.14728265
-0.04178899	0.75438469	0.00000000	-16.43558407
-1.21473924	-1.48163476	0.00000000	46.48663064
-1.10890384	-1.48163476	0.00000000	51.57794903
-1.16182154	-1.53455246	0.00000000	21.85473047
-1.16182154	-1.42871706	0.00000000	27.29598585
-2.43005336	-0.81881509	0.00000000	-7.43357817
-2.32421796	-0.81881509	0.00000000	-9.88041257
-2.37713566	-0.87173279	0.00000000	-3.03667174
-2.37713566	-0.76589740	0.00000000	-2.79487140
-2.49323545	0.59060577	0.00000000	-17.44906827
-2.38740004	0.59060577	0.00000000	-23.07236403
-2.44031774	0.53768808	0.00000000	9.08058702
-2.44031774	0.64352347	0.00000000	8.82304321
-1.33902835	1.35334636	0.00000000	48.99528137
-1.23319294	1.35334636	0.00000000	53.71213490
-1.28611065	1.30042866	0.00000000	88.24208102
-1.28611065	1.40626406	0.00000000	72.75981280
45 2657.0 # 737.7 nm,	(r3v5r2v1) 45		
0.070357	0.134057	0.00000000 1.0	# electr. mom.
0.00000000	0.00000000	-1.664952771	# magnet. mom.
1.31876649	-1.08237463	0.00000000	-210.83587238
0.00000000	-0.70208822	0.00000000	390.78793168
2.11091906	0.04406596	0.00000000	62.23573685
1.32524096	1.15754064	0.00000000	-237.06033840
-0.04178899	0.70146698	0.00000000	414.89973079
-1.16182154	-1.48163476	0.00000000	-129.08009073
-2.37713566	-0.81881509	0.00000000	20.22814663
-2.44031774	0.59060577	0.00000000	19.56427875
-1.28611065	1.35334636	0.00000000	-231.25665425
1.26584879	-1.08237463	0.00000000	54.04324040
1.37168419	-1.08237463	0.00000000	46.40249650
1.31876649	-1.13529233	0.00000000	50.37442758
1.31876649	-1.02945693	0.00000000	61.41952066
-0.05291770	-0.70208822	0.00000000	-95.04864346
0.05291770	-0.70208822	0.00000000	-98.97949540
0.00000000	-0.75500592	0.00000000	-99.90516458
0.00000000	-0.64917052	0.00000000	-98.94848137
2.05800137	0.04406596	0.00000000	-18.39536270
2.16383676	0.04406596	0.00000000	-9.29653384
2.11091906	-0.00885174	0.00000000	-16.29512854
2.11091906	0.09698366	0.00000000	-19.36743450
1.27232327	1.15754064	0.00000000	70.31500770
1.37815866	1.15754064	0.00000000	62.27966040
1.32524096	1.10462294	0.00000000	59.98091085
1.32524096	1.21045834	0.00000000	46.36889745
-0.09470669	0.70146698	0.00000000	-198.70358858
0.01112871	0.70146698	0.00000000	-201.53167111
-0.04178899	0.64854929	0.00000000	-1.89466116
-0.04178899	0.75438469	0.00000000	-14.50198595
-1.21473924	-1.48163476	0.00000000	41.01761527
-1.10890384	-1.48163476	0.00000000	45.50995502
-1.16182154	-1.53455246	0.00000000	19.28358571
-1.16182154	-1.42871706	0.00000000	24.08469340
-2.43005336	-0.81881509	0.00000000	-6.55903956
-2.32421796	-0.81881509	0.00000000	-8.71801109
-2.37713566	-0.87173279	0.00000000	-2.67941624
-2.37713566	-0.76589740	0.00000000	-2.46606300
-2.49323545	0.59060577	0.00000000	-15.39623671
-2.38740004	0.59060577	0.00000000	-20.35796827
-2.44031774	0.53768808	0.00000000	8.01228266
-2.44031774	0.64352347	0.00000000	7.78503813

```
-1.33902835 1.35334636 0.00000000 43.23113062
-1.23319294 1.35334636 0.00000000 47.39306020
-1.28611065 1.30042866 0.00000000 77.86065972
-1.28611065 1.40626406 0.00000000 64.19983482
45 2657.0 # 737.7 nm, (r3v6r2v1) 45
0.042214 0.080434 0.00000000 1.0 # electr. mom.
0.00000000 0.00000000 -1.664952771 # magnet. mom.
1.31876649 -1.08237463 0.00000000 -126.50152343
0.00000000 -0.70208822 0.00000000 234.47275901
2.11091906 0.04406596 0.00000000 37.34144211
1.32524096 1.15754064 0.00000000 -142.23620304
-0.04178899 0.70146698 0.00000000 248.93983847
-1.16182154 -1.48163476 0.00000000 -77.44805444
-2.37713566 -0.81881509 0.00000000 12.13688798
-2.44031774 0.59060577 0.00000000 11.73856725
-1.28611065 1.35334636 0.00000000 -138.75399255
1.26584879 -1.08237463 0.00000000 32.42594424
1.37168419 -1.08237463 0.00000000 27.84149790
1.31876649 -1.13529233 0.00000000 30.22465655
1.31876649 -1.02945693 0.00000000 36.85171240
-0.05291770 -0.70208822 0.00000000 -57.02918608
0.05291770 -0.70208822 0.00000000 -59.38769724
0.00000000 -0.75500592 0.00000000 -59.94309875
0.00000000 -0.64917052 0.00000000 -59.36908882
2.05800137 0.04406596 0.00000000 -11.03721762
2.16383676 0.04406596 0.00000000 -5.57792030
2.11091906 -0.00885174 0.00000000 -9.77707712
2.11091906 0.09698366 0.00000000 -11.62046070
1.27232327 1.15754064 0.00000000 42.18900462
1.37815866 1.15754064 0.00000000 37.36779624
1.32524096 1.10462294 0.00000000 35.98854651
1.32524096 1.21045834 0.00000000 27.82133847
-0.09470669 0.70146698 0.00000000 -119.22215315
0.01112871 0.70146698 0.00000000 -120.91900267
-0.04178899 0.64854929 0.00000000 -1.13679670
-0.04178899 0.75438469 0.00000000 -8.70119157
-1.21473924 -1.48163476 0.00000000 24.61056916
-1.10890384 -1.48163476 0.00000000 27.30597301
-1.16182154 -1.53455246 0.00000000 11.57015142
-1.16182154 -1.42871706 0.00000000 14.45081604
-2.43005336 -0.81881509 0.00000000 -3.93542374
-2.32421796 -0.81881509 0.00000000 -5.23080666
-2.37713566 -0.87173279 0.00000000 -1.60764974
-2.37713566 -0.76589740 0.00000000 -1.47963780
-2.49323545 0.59060577 0.00000000 -9.23774203
-2.38740004 0.59060577 0.00000000 -12.21478096
-2.44031774 0.53768808 0.00000000 4.80736960
-2.44031774 0.64352347 0.00000000 4.67102288
-1.33902835 1.35334636 0.00000000 25.93867837
-1.23319294 1.35334636 0.00000000 28.43583612
-1.28611065 1.30042866 0.00000000 46.71639583
-1.28611065 1.40626406 0.00000000 38.51990089
45 2657.0 # 737.7 nm, (r3v7r2v1) 45
0.084903 0.018762 0.00000000 1.0 # electr. mom.
0.00000000 0.00000000 -1.664952771 # magnet. mom.
1.31876649 -1.08237463 0.00000000 -133.52938584
0.00000000 -0.70208822 0.00000000 247.49902340
2.11091906 0.04406596 0.00000000 39.41596667
1.32524096 1.15754064 0.00000000 -150.13821432
-0.04178899 0.70146698 0.00000000 262.76982950
-1.16182154 -1.48163476 0.00000000 -81.75072413
-2.37713566 -0.81881509 0.00000000 12.81115953
-2.44031774 0.59060577 0.00000000 12.39070988
-1.28611065 1.35334636 0.00000000 -146.46254769
1.26584879 -1.08237463 0.00000000 34.22738559
1.37168419 -1.08237463 0.00000000 29.38824778
1.31876649 -1.13529233 0.00000000 31.90380413
1.31876649 -1.02945693 0.00000000 38.89902975
-0.05291770 -0.70208822 0.00000000 -60.19747419
0.05291770 -0.70208822 0.00000000 -62.68701375
0.00000000 -0.75500592 0.00000000 -63.27327090
0.00000000 -0.64917052 0.00000000 -62.66737154
2.05800137 0.04406596 0.00000000 -11.65039638
2.16383676 0.04406596 0.00000000 -5.88780477
2.11091906 -0.00885174 0.00000000 -10.32024807
2.11091906 0.09698366 0.00000000 -12.26604185
1.27232327 1.15754064 0.00000000 44.53283821
1.37815866 1.15754064 0.00000000 39.44378492
1.32524096 1.10462294 0.00000000 37.98791021
1.32524096 1.21045834 0.00000000 29.36696838
-0.09470669 0.70146698 0.00000000 -125.84560610
0.01112871 0.70146698 0.00000000 -127.63672504
-0.04178899 0.64854929 0.00000000 -1.19995207
-0.04178899 0.75438469 0.00000000 -9.18459110
-1.21473924 -1.48163476 0.00000000 25.97782300
-1.10890384 -1.48163476 0.00000000 28.82297152
-1.16182154 -1.53455246 0.00000000 12.21293762
```

```
     -1.16182154      -1.42871706       0.00000000       15.25363915
     -2.43005336      -0.81881509       0.00000000       -4.15405839
     -2.32421796      -0.81881509       0.00000000       -5.52140703
     -2.37713566      -0.87173279       0.00000000       -1.69696362
     -2.37713566      -0.76589740       0.00000000       -1.56183990
     -2.49323545       0.59060577       0.00000000       -9.75094992
     -2.38740004       0.59060577       0.00000000      -12.89337990
     -2.44031774       0.53768808       0.00000000        5.07444569
     -2.44031774       0.64352347       0.00000000        4.93052415
     -1.33902835       1.35334636       0.00000000       27.37971606
     -1.23319294       1.35334636       0.00000000       30.01560480
     -1.28611065       1.30042866       0.00000000       49.31175116
     -1.28611065       1.40626406       0.00000000       40.65989539
45    2657.0    # 737.7 nm,    (r3v8r2v1) 45
      0.018762         0.035749         0.00000000 1.0      # electr. mom.
      0.00000000       0.00000000      -1.664952771          # magnet. mom.
      1.31876649      -1.08237463       0.00000000      -56.22289930
      0.00000000      -0.70208822       0.00000000      104.21011511
      2.11091906       0.04406596       0.00000000       16.59619649
      1.32524096       1.15754064       0.00000000      -63.21609024
     -0.04178899       0.70146698       0.00000000      110.63992821
     -1.16182154      -1.48163476       0.00000000      -34.42135753
     -2.37713566      -0.81881509       0.00000000        5.39417243
     -2.44031774       0.59060577       0.00000000        5.21714100
     -1.28611065       1.35334636       0.00000000      -61.66844113
      1.26584879      -1.08237463       0.00000000       14.41153077
      1.37168419      -1.08237463       0.00000000       12.37399907
      1.31876649      -1.13529233       0.00000000       13.43318069
      1.31876649      -1.02945693       0.00000000       16.37853884
     -0.05291770      -0.70208822       0.00000000      -25.34630492
      0.05291770      -0.70208822       0.00000000      -26.39453211
      0.00000000      -0.75500592       0.00000000      -26.64137722
      0.00000000      -0.64917052       0.00000000      -26.38626170
      2.05800137       0.04406596       0.00000000       -4.90543005
      2.16383676       0.04406596       0.00000000       -2.47907569
      2.11091906      -0.00885174       0.00000000       -4.34536761
      2.11091906       0.09698366       0.00000000       -5.16464920
      1.27232327       1.15754064       0.00000000       18.75066872
      1.37815866       1.15754064       0.00000000       16.60790944
      1.32524096       1.10462294       0.00000000       15.99490956
      1.32524096       1.21045834       0.00000000       12.36503932
     -0.09470669       0.70146698       0.00000000      -52.98762362
      0.01112871       0.70146698       0.00000000      -53.74177896
     -0.04178899       0.64854929       0.00000000       -0.50524298
     -0.04178899       0.75438469       0.00000000       -3.86719625
     -1.21473924      -1.48163476       0.00000000       10.93803074
     -1.10890384      -1.48163476       0.00000000       12.13598801
     -1.16182154      -1.53455246       0.00000000        5.14228952
     -1.16182154      -1.42871706       0.00000000        6.42258491
     -2.43005336      -0.81881509       0.00000000       -1.74907722
     -2.32421796      -0.81881509       0.00000000       -2.32480296
     -2.37713566      -0.87173279       0.00000000       -0.71451100
     -2.37713566      -0.76589740       0.00000000       -0.65761680
     -2.49323545       0.59060577       0.00000000       -4.10566312
     -2.38740004       0.59060577       0.00000000       -5.42879154
     -2.44031774       0.53768808       0.00000000        2.13660871
     -2.44031774       0.64352347       0.00000000        2.07601017
     -1.33902835       1.35334636       0.00000000       11.52830150
     -1.23319294       1.35334636       0.00000000       12.63814939
     -1.28611065       1.30042866       0.00000000       20.76284259
     -1.28611065       1.40626406       0.00000000       17.11995595
45    2657.0    # 737.7 nm,    (r3v9r2v1) 45
      0.023452         0.044686         0.00000000 1.0      # electr. mom.
      0.00000000       0.00000000      -1.664952771          # magnet. mom.
      1.31876649      -1.08237463       0.00000000      -70.27862413
      0.00000000      -0.70208822       0.00000000      130.26264389
      2.11091906       0.04406596       0.00000000       20.74524562
      1.32524096       1.15754064       0.00000000      -79.02011280
     -0.04178899       0.70146698       0.00000000      138.29991026
     -1.16182154      -1.48163476       0.00000000      -43.02669691
     -2.37713566      -0.81881509       0.00000000        6.74271554
     -2.44031774       0.59060577       0.00000000        6.52142625
     -1.28611065       1.35334636       0.00000000      -77.08555142
      1.26584879      -1.08237463       0.00000000       18.01441347
      1.37168419      -1.08237463       0.00000000       15.46749883
      1.31876649      -1.13529233       0.00000000       16.79147586
      1.31876649      -1.02945693       0.00000000       20.47317355
     -0.05291770      -0.70208822       0.00000000      -31.68288115
      0.05291770      -0.70208822       0.00000000      -32.99316513
      0.00000000      -0.75500592       0.00000000      -33.30172153
      0.00000000      -0.64917052       0.00000000      -32.98282712
      2.05800137       0.04406596       0.00000000       -6.13178757
      2.16383676       0.04406596       0.00000000       -3.09884461
      2.11091906      -0.00885174       0.00000000       -5.43170951
      2.11091906       0.09698366       0.00000000       -6.45581150
      1.27232327       1.15754064       0.00000000       23.43833590
      1.37815866       1.15754064       0.00000000       20.75988680
```

	1.32524096	1.10462294	0.00000000	19.99363695
	1.32524096	1.21045834	0.00000000	15.45629915
	-0.09470669	0.70146698	0.00000000	-66.23452953
	0.01112871	0.70146698	0.00000000	-67.17722370
	-0.04178899	0.64854929	0.00000000	-0.63155372
	-0.04178899	0.75438469	0.00000000	-4.83399532
	-1.21473924	-1.48163476	0.00000000	13.67253842
	-1.10890384	-1.48163476	0.00000000	15.16998501
	-1.16182154	-1.53455246	0.00000000	6.42786190
	-1.16182154	-1.42871706	0.00000000	8.02823113
	-2.43005336	-0.81881509	0.00000000	-2.18634652
	-2.32421796	-0.81881509	0.00000000	-2.90600370
	-2.37713566	-0.87173279	0.00000000	-0.89313875
	-2.37713566	-0.76589740	0.00000000	-0.82202100
	-2.49323545	0.59060577	0.00000000	-5.13207890
	-2.38740004	0.59060577	0.00000000	-6.78598942
	-2.44031774	0.53768808	0.00000000	2.67076089
	-2.44031774	0.64352347	0.00000000	2.59501271
	-1.33902835	1.35334636	0.00000000	14.41037687
	-1.23319294	1.35334636	0.00000000	15.79768673
	-1.28611065	1.30042866	0.00000000	25.95355324
	-1.28611065	1.40626406	0.00000000	21.39994494
45	13555.0 # 737.7 nm, (r7r2v1) 45			
	0.323667	-0.07592	0.00000000 1.0	# electr. mom.
	0.00000000	0.00000000	-0.8485163126	# magnet. mom.
	1.31876649	-1.08237463	0.00000000	21.91434632
	0.00000000	-0.70208822	0.00000000	10.60586690
	2.11091906	0.04406596	0.00000000	9.38841172
	1.32524096	1.15754064	0.00000000	-13.57595939
	-0.04178899	0.70146698	0.00000000	119.23643020
	-1.16182154	-1.48163476	0.00000000	-125.26753406
	-2.37713566	-0.81881509	0.00000000	71.02561559
	-2.44031774	0.59060577	0.00000000	-30.88815023
	-1.28611065	1.35334636	0.00000000	-55.64821871
	1.26584879	-1.08237463	0.00000000	-6.73786065
	1.37168419	-1.08237463	0.00000000	-4.79976884
	1.31876649	-1.13529233	0.00000000	-4.09003888
	1.31876649	-1.02945693	0.00000000	-6.66024327
	-0.05291770	-0.70208822	0.00000000	46.32660500
	0.05291770	-0.70208822	0.00000000	47.61974419
	0.00000000	-0.75500592	0.00000000	-48.70598062
	0.00000000	-0.64917052	0.00000000	-55.74979321
	2.05800137	0.04406596	0.00000000	-6.38200102
	2.16383676	0.04406596	0.00000000	-6.16608127
	2.11091906	-0.00885174	0.00000000	0.78958265
	2.11091906	0.09698366	0.00000000	2.53957350
	1.27232327	1.15754064	0.00000000	-16.86695860
	1.37815866	1.15754064	0.00000000	-14.60880539
	1.32524096	1.10462294	0.00000000	24.24226485
	1.32524096	1.21045834	0.00000000	20.75271424
	-0.09470669	0.70146698	0.00000000	-61.04164442
	0.01112871	0.70146698	0.00000000	-64.27683286
	-0.04178899	0.64854929	0.00000000	2.33761827
	-0.04178899	0.75438469	0.00000000	4.34750233
	-1.21473924	-1.48163476	0.00000000	44.05721897
	-1.10890384	-1.48163476	0.00000000	45.67899927
	-1.16182154	-1.53455246	0.00000000	17.69496876
	-1.16182154	-1.42871706	0.00000000	17.55751946
	-2.43005336	-0.81881509	0.00000000	-18.47348807
	-2.32421796	-0.81881509	0.00000000	-19.61488457
	-2.37713566	-0.87173279	0.00000000	-16.37496360
	-2.37713566	-0.76589740	0.00000000	-16.25603967
	-2.49323545	0.59060577	0.00000000	15.88203744
	-2.38740004	0.59060577	0.00000000	18.78079357
	-2.44031774	0.53768808	0.00000000	-3.74142267
	-2.44031774	0.64352347	0.00000000	-0.10037215
	-1.33902835	1.35334636	0.00000000	6.20232032
	-1.23319294	1.35334636	0.00000000	5.75431146
	-1.28611065	1.30042866	0.00000000	22.18792280
	-1.28611065	1.40626406	0.00000000	21.10467433
45	15636.0 # 639.5 nm, (r8r2v1) 45			
	-0.85074	0.122021	0.00000000 1.0	# electr. mom.
	0.00000000	0.00000000	-0.9999114666E-01	# magnet. mom.
	1.31876649	-1.08237463	0.00000000	-46.45731431
	0.00000000	-0.70208822	0.00000000	153.79976151
	2.11091906	0.04406596	0.00000000	21.23880438
	1.32524096	1.15754064	0.00000000	169.86951950
	-0.04178899	0.70146698	0.00000000	-283.12922580
	-1.16182154	-1.48163476	0.00000000	-30.65002339
	-2.37713566	-0.81881509	0.00000000	53.16858893
	-2.44031774	0.59060577	0.00000000	-50.88375522
	-1.28611065	1.35334636	0.00000000	125.30260283
	1.26584879	-1.08237463	0.00000000	5.62503126
	1.37168419	-1.08237463	0.00000000	5.68185160
	1.31876649	-1.13529233	0.00000000	17.27348300
	1.31876649	-1.02945693	0.00000000	18.12706908
	-0.05291770	-0.70208822	0.00000000	-36.29592093

```
        0.05291770       -0.70208822        0.00000000      -32.26410274
        0.00000000       -0.75500592        0.00000000      -43.51332666
        0.00000000       -0.64917052        0.00000000      -42.15295647
        2.05800137        0.04406596        0.00000000        7.23926719
        2.16383676        0.04406596        0.00000000        6.89429737
        2.11091906       -0.00885174        0.00000000      -22.60549497
        2.11091906        0.09698366        0.00000000      -12.89432382
        1.27232327        1.15754064        0.00000000      -46.39006120
        1.37815866        1.15754064        0.00000000      -44.64969232
        1.32524096        1.10462294        0.00000000      -41.58256563
        1.32524096        1.21045834        0.00000000      -37.57681978
       -0.09470669        0.70146698        0.00000000      113.48066076
        0.01112871        0.70146698        0.00000000      106.02701625
       -0.04178899        0.64854929        0.00000000       29.86311215
       -0.04178899        0.75438469        0.00000000       34.28828866
       -1.21473924       -1.48163476        0.00000000        1.18980650
       -1.10890384       -1.48163476        0.00000000        3.28194854
       -1.16182154       -1.53455246        0.00000000       10.41999105
       -1.16182154       -1.42871706        0.00000000       16.47935573
       -2.43005336       -0.81881509        0.00000000      -32.52060266
       -2.32421796       -0.81881509        0.00000000      -37.98420516
       -2.37713566       -0.87173279        0.00000000        7.89720568
       -2.37713566       -0.76589740        0.00000000        9.08400850
       -2.49323545        0.59060577        0.00000000        5.59530348
       -2.38740004        0.59060577        0.00000000       11.16609595
       -2.44031774        0.53768808        0.00000000       18.46863734
       -2.44031774        0.64352347        0.00000000       16.33773149
       -1.33902835        1.35334636        0.00000000      -33.21055314
       -1.23319294        1.35334636        0.00000000      -33.55658712
       -1.28611065        1.30042866        0.00000000      -33.66565892
       -1.28611065        1.40626406        0.00000000      -25.81624852
&TRANSITION 3->...
  0    0.0   # (r2v3r2v2) 45
        0.00000000        0.00000000        0.00000000 1.0       # electr. mom.
        0.00000000        0.00000000        0.00000000             # magnet. mom.
  0    0.0   # (r2v4r2v2) 45
        0.00000000        0.00000000        0.00000000 1.0       # electr. mom.
        0.00000000        0.00000000        0.00000000             # magnet. mom.
  0    0.0   # (r2v5r2v2) 45
        0.00000000        0.00000000        0.00000000 1.0       # electr. mom.
        0.00000000        0.00000000        0.00000000             # magnet. mom.
  0    0.0   # (r2v6r2v2) 45
        0.00000000        0.00000000        0.00000000 1.0       # electr. mom.
        0.00000000        0.00000000        0.00000000             # magnet. mom.
  0    0.0   # (r2v7r2v2) 45
        0.00000000        0.00000000        0.00000000 1.0       # electr. mom.
        0.00000000        0.00000000        0.00000000             # magnet. mom.
 45   2657.0   # 737.7 nm,   (r3v1r2v2) 45
        0.101337          0.193086          0.00000000 1.0     # electr. mom.
        0.00000000        0.00000000       -1.664952771         # magnet. mom.
        1.31876649       -1.08237463        0.00000000     -303.67166780
        0.00000000       -0.70208822        0.00000000      562.86068224
        2.11091906        0.04406596        0.00000000       89.63953710
        1.32524096        1.15754064        0.00000000     -341.44335837
       -0.04178899        0.70146698        0.00000000      597.58945096
       -1.16182154       -1.48163476        0.00000000     -185.91696939
       -2.37713566       -0.81881509        0.00000000       29.13505635
       -2.44031774        0.59060577        0.00000000       28.17887246
       -1.28611065        1.35334636        0.00000000     -333.08418104
        1.26584879       -1.08237463        0.00000000       77.83969949
        1.37168419       -1.08237463        0.00000000       66.83456350
        1.31876649       -1.13529233        0.00000000       72.55542553
        1.31876649       -1.02945693        0.00000000       88.46392250
       -0.05291770       -0.70208822        0.00000000     -136.90070744
        0.05291770       -0.70208822        0.00000000     -142.56240224
        0.00000000       -0.75500592        0.00000000     -143.89566447
        0.00000000       -0.64917052        0.00000000     -142.51773204
        2.05800137        0.04406596        0.00000000      -26.49525628
        2.16383676        0.04406596        0.00000000      -13.39000761
        2.11091906       -0.00885174        0.00000000      -23.47024159
        2.11091906        0.09698366        0.00000000      -27.89535324
        1.27232327        1.15754064        0.00000000      101.27629334
        1.37815866        1.15754064        0.00000000       89.70280119
        1.32524096        1.10462294        0.00000000       86.39186031
        1.32524096        1.21045834        0.00000000       66.78617004
       -0.09470669        0.70146698        0.00000000     -286.19726548
        0.01112871        0.70146698        0.00000000     -290.27061662
       -0.04178899        0.64854929        0.00000000       -2.72892326
       -0.04178899        0.75438469        0.00000000      -20.88753782
       -1.21473924       -1.48163476        0.00000000       59.07859747
       -1.10890384       -1.48163476        0.00000000       65.54901587
       -1.16182154       -1.53455246        0.00000000       27.77458393
       -1.16182154       -1.42871706        0.00000000       34.68972775
       -2.43005336       -0.81881509        0.00000000       -9.44713279
       -2.32421796       -0.81881509        0.00000000      -12.55674824
       -2.37713566       -0.87173279        0.00000000       -3.85922371
       -2.37713566       -0.76589740        0.00000000       -3.55192623
```

```
   -2.49323545     0.59060577     0.00000000    -22.17554739
   -2.38740004     0.59060577     0.00000000    -29.32204139
   -2.44031774     0.53768808     0.00000000     11.54027164
   -2.44031774     0.64352347     0.00000000     11.21296621
   -1.33902835     1.35334636     0.00000000     62.26677362
   -1.23319294     1.35334636     0.00000000     68.26129478
   -1.28611065     1.30042866     0.00000000    112.14446634
   -1.28611065     1.40626406     0.00000000     92.46847177
45    2657.0    # 737.7 nm,    (r3v2r2v2) 45
    0.094225       0.179536       0.00000000 1.0     # electr. mom.
    0.00000000     0.00000000    -1.664952771       # magnet. mom.
    1.31876649    -1.08237463     0.00000000   -282.36137532
    0.00000000    -0.70208822     0.00000000    523.36168699
    2.11091906     0.04406596     0.00000000     83.34904327
    1.32524096     1.15754064     0.00000000   -317.48242094
   -0.04178899     0.70146698     0.00000000    555.65334914
   -1.16182154    -1.48163476     0.00000000   -172.87016452
   -2.37713566    -0.81881509     0.00000000     27.09049100
   -2.44031774     0.59060577     0.00000000     26.20140773
   -1.28611065     1.35334636     0.00000000   -309.70985255
    1.26584879    -1.08237463     0.00000000     72.37726443
    1.37168419    -1.08237463     0.00000000     62.14441870
    1.31876649    -1.13529233     0.00000000     67.46381672
    1.31876649    -1.02945693     0.00000000     82.25592794
   -0.05291770    -0.70208822     0.00000000   -127.29364025
    0.05291770    -0.70208822     0.00000000   -132.55802314
    0.00000000    -0.75500592     0.00000000   -133.79772310
    0.00000000    -0.64917052     0.00000000   -132.51648769
    2.05800137     0.04406596     0.00000000    -24.63594005
    2.16383676     0.04406596     0.00000000    -12.45035796
    2.11091906    -0.00885174     0.00000000    -21.82320709
    2.11091906     0.09698366     0.00000000    -25.93778459
    1.27232327     1.15754064     0.00000000     94.16918504
    1.37815866     1.15754064     0.00000000     83.40786777
    1.32524096     1.10462294     0.00000000     80.32927362
    1.32524096     1.21045834     0.00000000     62.09942126
   -0.09470669     0.70146698     0.00000000   -266.11324685
    0.01112871     0.70146698     0.00000000   -269.90074879
   -0.04178899     0.64854929     0.00000000     -2.53741987
   -0.04178899     0.75438469     0.00000000    -19.42174570
   -1.21473924    -1.48163476     0.00000000     54.93273098
   -1.10890384    -1.48163476     0.00000000     60.94908493
   -1.16182154    -1.53455246     0.00000000     25.82549032
   -1.16182154    -1.42871706     0.00000000     32.25536089
   -2.43005336    -0.81881509     0.00000000     -8.78417610
   -2.32421796    -0.81881509     0.00000000    -11.67557292
   -2.37713566    -0.87173279     0.00000000     -3.58840100
   -2.37713566    -0.76589740     0.00000000     -3.30266824
   -2.49323545     0.59060577     0.00000000    -20.61936863
   -2.38740004     0.59060577     0.00000000    -27.26435427
   -2.44031774     0.53768808     0.00000000     10.73042802
   -2.44031774     0.64352347     0.00000000     10.42609139
   -1.33902835     1.35334636     0.00000000     57.89717547
   -1.23319294     1.35334636     0.00000000     63.47102848
   -1.28611065     1.30042866     0.00000000    104.27467923
   -1.28611065     1.40626406     0.00000000     85.97945621
45    2657.0    # 737.7 nm,    (r3v3r2v2) 45
    0.056891       0.108399       0.00000000 1.0     # electr. mom.
    0.00000000     0.00000000    -1.664952771       # magnet. mom.
    1.31876649    -1.08237463     0.00000000   -170.48233982
    0.00000000    -0.70208822     0.00000000    315.99196196
    2.11091906     0.04406596     0.00000000     50.32395065
    1.32524096     1.15754064     0.00000000   -191.68749944
   -0.04178899     0.70146698     0.00000000    335.48881457
   -1.16182154    -1.48163476     0.00000000   -104.37443896
   -2.37713566    -0.81881509     0.00000000     16.35652287
   -2.44031774     0.59060577     0.00000000     15.81971787
   -1.28611065     1.35334636     0.00000000   -186.99462795
    1.26584879    -1.08237463     0.00000000     43.69948041
    1.37168419    -1.08237463     0.00000000     37.52115846
    1.31876649    -1.13529233     0.00000000     40.73287047
    1.31876649    -1.02945693     0.00000000     49.66395649
   -0.05291770    -0.70208822     0.00000000    -76.85653751
    0.05291770    -0.70208822     0.00000000    -80.03503284
    0.00000000    -0.75500592     0.00000000    -80.78353093
    0.00000000    -0.64917052     0.00000000    -80.00995483
    2.05800137     0.04406596     0.00000000    -14.87452984
    2.16383676     0.04406596     0.00000000     -7.51719726
    2.11091906    -0.00885174     0.00000000    -13.17627598
    2.11091906     0.09698366     0.00000000    -15.66054919
    1.27232327     1.15754064     0.00000000     56.85686644
    1.37815866     1.15754064     0.00000000     50.35946733
    1.32524096     1.10462294     0.00000000     48.50069351
    1.32524096     1.21045834     0.00000000     37.49399020
   -0.09470669     0.70146698     0.00000000   -160.67214904
    0.01112871     0.70146698     0.00000000   -162.95894266
   -0.04178899     0.64854929     0.00000000     -1.53202709
```

```
   -0.04178899     0.75438469     0.00000000    -11.72633702
   -1.21473924    -1.48163476     0.00000000     33.16693191
   -1.10890384    -1.48163476     0.00000000     36.79944750
   -1.16182154    -1.53455246     0.00000000     15.59274887
   -1.16182154    -1.42871706     0.00000000     19.47493488
   -2.43005336    -0.81881509     0.00000000     -5.30365349
   -2.32421796    -0.81881509     0.00000000     -7.04940252
   -2.37713566    -0.87173279     0.00000000     -2.16658173
   -2.37713566    -0.76589740     0.00000000     -1.99406385
   -2.49323545     0.59060577     0.00000000    -12.44943011
   -2.38740004     0.59060577     0.00000000    -16.46149692
   -2.44031774     0.53768808     0.00000000      6.47874899
   -2.44031774     0.64352347     0.00000000      6.29499857
   -1.33902835     1.35334636     0.00000000     34.95678519
   -1.23319294     1.35334636     0.00000000     38.32213040
   -1.28611065     1.30042866     0.00000000     62.95829689
   -1.28611065     1.40626406     0.00000000     51.91212450
45   2657.0   # 737.7 nm,   (r3v4r2v2) 45
    0.060446       0.115174       0.00000000 1.0   # electr. mom.
    0.00000000     0.00000000    -1.664952771      # magnet. mom.
    1.31876649    -1.08237463     0.00000000   -181.13748606
    0.00000000    -0.70208822     0.00000000    335.74145958
    2.11091906     0.04406596     0.00000000     53.46919757
    1.32524096     1.15754064     0.00000000   -203.66796815
   -0.04178899     0.70146698     0.00000000    356.45686548
   -1.16182154    -1.48163476     0.00000000   -110.89784139
   -2.37713566    -0.81881509     0.00000000     17.37880554
   -2.44031774     0.59060577     0.00000000     16.80845024
   -1.28611065     1.35334636     0.00000000   -198.68179220
    1.26584879    -1.08237463     0.00000000     46.43069794
    1.37168419    -1.08237463     0.00000000     39.86623086
    1.31876649    -1.13529233     0.00000000     43.27867488
    1.31876649    -1.02945693     0.00000000     52.76795377
   -0.05291770    -0.70208822     0.00000000    -81.66007110
    0.05291770    -0.70208822     0.00000000    -85.03722239
    0.00000000    -0.75500592     0.00000000    -85.83250161
    0.00000000    -0.64917052     0.00000000    -85.01057701
    2.05800137     0.04406596     0.00000000    -15.80418796
    2.16383676     0.04406596     0.00000000     -7.98702209
    2.11091906    -0.00885174     0.00000000    -13.99979323
    2.11091906     0.09698366     0.00000000    -16.63933351
    1.27232327     1.15754064     0.00000000     60.41042059
    1.37815866     1.15754064     0.00000000     53.50693404
    1.32524096     1.10462294     0.00000000     51.53198685
    1.32524096     1.21045834     0.00000000     39.83736458
   -0.09470669     0.70146698     0.00000000   -170.71415836
    0.01112871     0.70146698     0.00000000   -173.14387658
   -0.04178899     0.64854929     0.00000000     -1.62777878
   -0.04178899     0.75438469     0.00000000    -12.45923309
   -1.21473924    -1.48163476     0.00000000     35.23986516
   -1.10890384    -1.48163476     0.00000000     39.09941297
   -1.16182154    -1.53455246     0.00000000     16.56729568
   -1.16182154    -1.42871706     0.00000000     20.69211831
   -2.43005336    -0.81881509     0.00000000     -5.63513184
   -2.32421796    -0.81881509     0.00000000     -7.48999018
   -2.37713566    -0.87173279     0.00000000     -2.30199309
   -2.37713566    -0.76589740     0.00000000     -2.11869284
   -2.49323545     0.59060577     0.00000000    -13.22751950
   -2.38740004     0.59060577     0.00000000    -17.49034048
   -2.44031774     0.53768808     0.00000000      6.88367080
   -2.44031774     0.64352347     0.00000000      6.68843598
   -1.33902835     1.35334636     0.00000000     37.14158426
   -1.23319294     1.35334636     0.00000000     40.71726355
   -1.28611065     1.30042866     0.00000000     66.89319045
   -1.28611065     1.40626406     0.00000000     55.15663228
45   2657.0   # 737.7 nm,   (r3v5r2v2) 45
    0.053335       0.101624       0.00000000 1.0   # electr. mom.
    0.00000000     0.00000000    -1.664952771      # magnet. mom.
    1.31876649    -1.08237463     0.00000000   -159.82719358
    0.00000000    -0.70208822     0.00000000    296.24246434
    2.11091906     0.04406596     0.00000000     47.17870374
    1.32524096     1.15754064     0.00000000   -179.70703072
   -0.04178899     0.70146698     0.00000000    314.52076366
   -1.16182154    -1.48163476     0.00000000    -97.85103652
   -2.37713566    -0.81881509     0.00000000     15.33424019
   -2.44031774     0.59060577     0.00000000     14.83098551
   -1.28611065     1.35334636     0.00000000   -175.30746371
    1.26584879    -1.08237463     0.00000000     40.96826289
    1.37168419    -1.08237463     0.00000000     35.17608605
    1.31876649    -1.13529233     0.00000000     38.18706607
    1.31876649    -1.02945693     0.00000000     46.55995921
   -0.05291770    -0.70208822     0.00000000    -72.05300391
    0.05291770    -0.70208822     0.00000000    -75.03284329
    0.00000000    -0.75500592     0.00000000    -75.73456025
    0.00000000    -0.64917052     0.00000000    -75.00933266
    2.05800137     0.04406596     0.00000000    -13.94487173
    2.16383676     0.04406596     0.00000000     -7.04737243
```

```
      2.11091906      -0.00885174      0.00000000      -12.35275873
      2.11091906       0.09698366      0.00000000      -14.68176486
      1.27232327       1.15754064      0.00000000       53.30331228
      1.37815866       1.15754064      0.00000000       47.21200063
      1.32524096       1.10462294      0.00000000       45.46940016
      1.32524096       1.21045834      0.00000000       35.15061581
     -0.09470669       0.70146698      0.00000000     -150.63013973
      0.01112871       0.70146698      0.00000000     -152.77400875
     -0.04178899       0.64854929      0.00000000       -1.43627540
     -0.04178899       0.75438469      0.00000000      -10.99344096
     -1.21473924      -1.48163476      0.00000000       31.09399867
     -1.10890384      -1.48163476      0.00000000       34.49948203
     -1.16182154      -1.53455246      0.00000000       14.61820207
     -1.16182154      -1.42871706      0.00000000       18.25775145
     -2.43005336      -0.81881509      0.00000000       -4.97217515
     -2.32421796      -0.81881509      0.00000000       -6.60881486
     -2.37713566      -0.87173279      0.00000000       -2.03117037
     -2.37713566      -0.76589740      0.00000000       -1.86943486
     -2.49323545       0.59060577      0.00000000      -11.67134073
     -2.38740004       0.59060577      0.00000000      -15.43265336
     -2.44031774       0.53768808      0.00000000        6.07382718
     -2.44031774       0.64352347      0.00000000        5.90156116
     -1.33902835       1.35334636      0.00000000       32.77198612
     -1.23319294       1.35334636      0.00000000       35.92699725
     -1.28611065       1.30042866      0.00000000       59.02340334
     -1.28611065       1.40626406      0.00000000       48.66761672
45    2657.0    # 737.7 nm,    (r3v6r2v2) 45
      0.032001         0.060974        0.00000000 1.0      # electr. mom.
      0.00000000       0.00000000     -1.664952771         # magnet. mom.
      1.31876649      -1.08237463      0.00000000      -95.89631615
      0.00000000      -0.70208822      0.00000000      177.74547860
      2.11091906       0.04406596      0.00000000       28.30722224
      1.32524096       1.15754064      0.00000000     -107.82421843
     -0.04178899       0.70146698      0.00000000      188.71245820
     -1.16182154      -1.48163476      0.00000000      -58.71062191
     -2.37713566      -0.81881509      0.00000000        9.20054411
     -2.44031774       0.59060577      0.00000000        8.89859130
     -1.28611065       1.35334636      0.00000000     -105.18447822
      1.26584879      -1.08237463      0.00000000       24.58095773
      1.37168419      -1.08237463      0.00000000       21.10565163
      1.31876649      -1.13529233      0.00000000       22.91223964
      1.31876649      -1.02945693      0.00000000       27.93597553
     -0.05291770      -0.70208822      0.00000000      -43.23180235
      0.05291770      -0.70208822      0.00000000      -45.01970597
      0.00000000      -0.75500592      0.00000000      -45.44073615
      0.00000000      -0.64917052      0.00000000      -45.00559959
      2.05800137       0.04406596      0.00000000       -8.36692304
      2.16383676       0.04406596      0.00000000       -4.22842346
      2.11091906      -0.00885174      0.00000000       -7.41165524
      2.11091906       0.09698366      0.00000000       -8.80905892
      1.27232327       1.15754064      0.00000000       31.98198737
      1.37815866       1.15754064      0.00000000       28.32720038
      1.32524096       1.10462294      0.00000000       27.28164010
      1.32524096       1.21045834      0.00000000       21.09036949
     -0.09470669       0.70146698      0.00000000      -90.37808384
      0.01112871       0.70146698      0.00000000      -91.66440525
     -0.04178899       0.64854929      0.00000000       -0.86176524
     -0.04178899       0.75438469      0.00000000       -6.59606458
     -1.21473924      -1.48163476      0.00000000       18.65639920
     -1.10890384      -1.48163476      0.00000000       20.69968922
     -1.16182154      -1.53455246      0.00000000        8.77092124
     -1.16182154      -1.42871706      0.00000000       10.95465087
     -2.43005336      -0.81881509      0.00000000       -2.98330509
     -2.32421796      -0.81881509      0.00000000       -3.96528892
     -2.37713566      -0.87173279      0.00000000       -1.21870222
     -2.37713566      -0.76589740      0.00000000       -1.12166091
     -2.49323545       0.59060577      0.00000000       -7.00280444
     -2.38740004       0.59060577      0.00000000       -9.25959202
     -2.44031774       0.53768808      0.00000000        3.64429631
     -2.44031774       0.64352347      0.00000000        3.54093670
     -1.33902835       1.35334636      0.00000000       19.66319167
     -1.23319294       1.35334636      0.00000000       21.55619835
     -1.28611065       1.30042866      0.00000000       35.41404200
     -1.28611065       1.40626406      0.00000000       29.20057003
45    2657.0    # 737.7 nm,    (r3v7r2v2) 45
      0.033779         0.064362        0.00000000 1.0      # electr. mom.
      0.00000000       0.00000000     -1.664952771         # magnet. mom.
      1.31876649      -1.08237463      0.00000000     -101.22388927
      0.00000000      -0.70208822      0.00000000      187.62022741
      2.11091906       0.04406596      0.00000000       29.87984570
      1.32524096       1.15754064      0.00000000     -113.81445279
     -0.04178899       0.70146698      0.00000000      199.19648365
     -1.16182154      -1.48163476      0.00000000      -61.97232313
     -2.37713566      -0.81881509      0.00000000        9.71168545
     -2.44031774       0.59060577      0.00000000        9.39295749
     -1.28611065       1.35334636      0.00000000     -111.02806035
      1.26584879      -1.08237463      0.00000000       25.94656650
```

1.37168419 -1.08237463 0.00000000 22.27818783
1.31876649 -1.13529233 0.00000000 24.18514184
1.31876649 -1.02945693 0.00000000 29.48797417
-0.05291770 -0.70208822 0.00000000 -45.63356915
0.05291770 -0.70208822 0.00000000 -47.52080075
0.00000000 -0.75500592 0.00000000 -47.96522149
0.00000000 -0.64917052 0.00000000 -47.50591068
2.05800137 0.04406596 0.00000000 -8.83175209
2.16383676 0.04406596 0.00000000 -4.46333587
2.11091906 -0.00885174 0.00000000 -7.82341386
2.11091906 0.09698366 0.00000000 -9.29845108
1.27232327 1.15754064 0.00000000 33.75876445
1.37815866 1.15754064 0.00000000 29.90093373
1.32524096 1.10462294 0.00000000 28.79728677
1.32524096 1.21045834 0.00000000 22.26205668
-0.09470669 0.70146698 0.00000000 -95.39908849
0.01112871 0.70146698 0.00000000 -96.75687221
-0.04178899 0.64854929 0.00000000 -0.90964109
-0.04178899 0.75438469 0.00000000 -6.96251261
-1.21473924 -1.48163476 0.00000000 19.69286582
-1.10890384 -1.48163476 0.00000000 21.84967196
-1.16182154 -1.53455246 0.00000000 9.25819464
-1.16182154 -1.42871706 0.00000000 11.56324258
-2.43005336 -0.81881509 0.00000000 -3.14904426
-2.32421796 -0.81881509 0.00000000 -4.18558275
-2.37713566 -0.87173279 0.00000000 -1.28640790
-2.37713566 -0.76589740 0.00000000 -1.18397541
-2.49323545 0.59060577 0.00000000 -7.39184913
-2.38740004 0.59060577 0.00000000 -9.77401380
-2.44031774 0.53768808 0.00000000 3.84675721
-2.44031774 0.64352347 0.00000000 3.73765540
-1.33902835 1.35334636 0.00000000 20.75559121
-1.23319294 1.35334636 0.00000000 22.75376493
-1.28611065 1.30042866 0.00000000 37.38148878
-1.28611065 1.40626406 0.00000000 30.82282392
45 2657.0 # 737.7 nm, (r3v8r2v2) 45
0.014223 0.0271 0.00000000 1.0 # electr. mom.
0.00000000 0.00000000 -1.664952771 # magnet. mom.
1.31876649 -1.08237463 0.00000000 -42.62058495
0.00000000 -0.70208822 0.00000000 78.99799049
2.11091906 0.04406596 0.00000000 12.58098766
1.32524096 1.15754064 0.00000000 -47.92187486
-0.04178899 0.70146698 0.00000000 83.87220364
-1.16182154 -1.48163476 0.00000000 -26.09360974
-2.37713566 -0.81881509 0.00000000 4.08913072
-2.44031774 0.59060577 0.00000000 3.95492947
-1.28611065 1.35334636 0.00000000 -46.74865699
1.26584879 -1.08237463 0.00000000 10.92487010
1.37168419 -1.08237463 0.00000000 9.38028961
1.31876649 -1.13529233 0.00000000 10.18321762
1.31876649 -1.02945693 0.00000000 12.41598912
-0.05291770 -0.70208822 0.00000000 -19.21413438
0.05291770 -0.70208822 0.00000000 -20.00875821
0.00000000 -0.75500592 0.00000000 -20.19588273
0.00000000 -0.64917052 0.00000000 -20.00248871
2.05800137 0.04406596 0.00000000 -3.71863246
2.16383676 0.04406596 0.00000000 -1.87929931
2.11091906 -0.00885174 0.00000000 -3.29406899
2.11091906 0.09698366 0.00000000 -3.91513730
1.27232327 1.15754064 0.00000000 14.21421661
1.37815866 1.15754064 0.00000000 12.58986683
1.32524096 1.10462294 0.00000000 12.12517338
1.32524096 1.21045834 0.00000000 9.37349755
-0.09470669 0.70146698 0.00000000 -40.16803726
0.01112871 0.70146698 0.00000000 -40.73973567
-0.04178899 0.64854929 0.00000000 -0.38300677
-0.04178899 0.75438469 0.00000000 -2.93158426
-1.21473924 -1.48163476 0.00000000 8.29173298
-1.10890384 -1.48163476 0.00000000 9.19986188
-1.16182154 -1.53455246 0.00000000 3.89818722
-1.16182154 -1.42871706 0.00000000 4.86873372
-2.43005336 -0.81881509 0.00000000 -1.32591337
-2.32421796 -0.81881509 0.00000000 -1.76235063
-2.37713566 -0.87173279 0.00000000 -0.54164543
-2.37713566 -0.76589740 0.00000000 -0.49851596
-2.49323545 0.59060577 0.00000000 -3.11235753
-2.38740004 0.59060577 0.00000000 -4.11537423
-2.44031774 0.53768808 0.00000000 1.61968725
-2.44031774 0.64352347 0.00000000 1.57374964
-1.33902835 1.35334636 0.00000000 8.73919630
-1.23319294 1.35334636 0.00000000 9.58053260
-1.28611065 1.30042866 0.00000000 15.73957422
-1.28611065 1.40626406 0.00000000 12.97803113
45 2657.0 # 737.7 nm, (r3v9r2v2) 45
0.017778 0.033875 0.00000000 1.0 # electr. mom.
0.00000000 0.00000000 -1.664952771 # magnet. mom.
1.31876649 -1.08237463 0.00000000 -53.27573119

```
     0.00000000     -0.70208822      0.00000000       98.74748811
     2.11091906      0.04406596      0.00000000       15.72623458
     1.32524096      1.15754064      0.00000000      -59.90234357
    -0.04178899      0.70146698      0.00000000      104.84025455
    -1.16182154     -1.48163476      0.00000000      -32.61701217
    -2.37713566     -0.81881509      0.00000000        5.11141340
    -2.44031774      0.59060577      0.00000000        4.94366184
    -1.28611065      1.35334636      0.00000000      -58.43582124
     1.26584879     -1.08237463      0.00000000       13.65608763
     1.37168419     -1.08237463      0.00000000       11.72536202
     1.31876649     -1.13529233      0.00000000       12.72902202
     1.31876649     -1.02945693      0.00000000       15.51998640
    -0.05291770     -0.70208822      0.00000000      -24.01766797
     0.05291770     -0.70208822      0.00000000      -25.01094776
     0.00000000     -0.75500592      0.00000000      -25.24485342
     0.00000000     -0.64917052      0.00000000      -25.00311089
     2.05800137      0.04406596      0.00000000       -4.64829058
     2.16383676      0.04406596      0.00000000       -2.34912414
     2.11091906     -0.00885174      0.00000000       -4.11758624
     2.11091906      0.09698366      0.00000000       -4.89392162
     1.27232327      1.15754064      0.00000000       17.76777076
     1.37815866      1.15754064      0.00000000       15.73733354
     1.32524096      1.10462294      0.00000000       15.15646672
     1.32524096      1.21045834      0.00000000       11.71687194
    -0.09470669      0.70146698      0.00000000      -50.21004658
     0.01112871      0.70146698      0.00000000      -50.92466958
    -0.04178899      0.64854929      0.00000000       -0.47875847
    -0.04178899      0.75438469      0.00000000       -3.66448032
    -1.21473924     -1.48163476      0.00000000       10.36466622
    -1.10890384     -1.48163476      0.00000000       11.49982734
    -1.16182154     -1.53455246      0.00000000        4.87273402
    -1.16182154     -1.42871706      0.00000000        6.08591715
    -2.43005336     -0.81881509      0.00000000       -1.65739172
    -2.32421796     -0.81881509      0.00000000       -2.20293829
    -2.37713566     -0.87173279      0.00000000       -0.67705679
    -2.37713566     -0.76589740      0.00000000       -0.62314495
    -2.49323545      0.59060577      0.00000000       -3.89044691
    -2.38740004      0.59060577      0.00000000       -5.14421779
    -2.44031774      0.53768808      0.00000000        2.02460906
    -2.44031774      0.64352347      0.00000000        1.96718705
    -1.33902835      1.35334636      0.00000000       10.92399537
    -1.23319294      1.35334636      0.00000000       11.97566575
    -1.28611065      1.30042866      0.00000000       19.67446778
    -1.28611065      1.40626406      0.00000000       16.22253891
45    13555.0    # 737.7 nm,    (r7r2v2) 45
     0.24536        -0.05755         0.00000000 1.0      # electr. mom.
     0.00000000      0.00000000     -0.8485163126         # magnet. mom.
     1.31876649     -1.08237463      0.00000000       16.61248834
     0.00000000     -0.70208822      0.00000000        8.03993136
     2.11091906      0.04406596      0.00000000        7.11702179
     1.32524096      1.15754064      0.00000000      -10.29145308
    -0.04178899      0.70146698      0.00000000       90.38890676
    -1.16182154     -1.48163476      0.00000000      -94.96087260
    -2.37713566     -0.81881509      0.00000000       53.84199892
    -2.44031774      0.59060577      0.00000000      -23.41521066
    -1.28611065      1.35334636      0.00000000      -42.18493999
     1.26584879     -1.08237463      0.00000000       -5.10773307
     1.37168419     -1.08237463      0.00000000       -3.63853444
     1.31876649     -1.13529233      0.00000000       -3.10051334
     1.31876649     -1.02945693      0.00000000       -5.04889409
    -0.05291770     -0.70208822      0.00000000       35.11855540
     0.05291770     -0.70208822      0.00000000       36.09883834
     0.00000000     -0.75500592      0.00000000      -36.92227563
     0.00000000     -0.64917052      0.00000000      -42.26194002
     2.05800137      0.04406596      0.00000000       -4.83796851
     2.16383676      0.04406596      0.00000000       -4.67428741
     2.11091906     -0.00885174      0.00000000        0.59855459
     2.11091906      0.09698366      0.00000000        1.92516056
     1.27232327      1.15754064      0.00000000      -12.78624281
     1.37815866      1.15754064      0.00000000      -11.07441699
     1.32524096      1.10462294      0.00000000       18.37720077
     1.32524096      1.21045834      0.00000000       15.73189628
    -0.09470669      0.70146698      0.00000000      -46.27350464
     0.01112871      0.70146698      0.00000000      -48.72598620
    -0.04178899      0.64854929      0.00000000        1.77206546
    -0.04178899      0.75438469      0.00000000        3.29568725
    -1.21473924     -1.48163476      0.00000000       33.39821438
    -1.10890384     -1.48163476      0.00000000       34.62762848
    -1.16182154     -1.53455246      0.00000000       13.41392793
    -1.16182154     -1.42871706      0.00000000       13.30973250
    -2.43005336     -0.81881509      0.00000000      -14.00409580
    -2.32421796     -0.81881509      0.00000000      -14.86934798
    -2.37713566     -0.87173279      0.00000000      -12.41327886
    -2.37713566     -0.76589740      0.00000000      -12.32312685
    -2.49323545      0.59060577      0.00000000       12.03960903
    -2.38740004      0.59060577      0.00000000       14.23705319
    -2.44031774      0.53768808      0.00000000       -2.83623977
```

```
    -2.44031774    0.64352347    0.00000000    -0.07608856
    -1.33902835    1.35334636    0.00000000     4.70175895
    -1.23319294    1.35334636    0.00000000     4.36213933
    -1.28611065    1.30042866    0.00000000    16.81987696
    -1.28611065    1.40626406    0.00000000    15.99870474
45  15636.0   # 639.5 nm,   (r8r2v2) 45
    -0.64492    0.0925    0.00000000 1.0   # electr. mom.
     0.00000000    0.00000000    -0.9999114666E-01   # magnet. mom.
     1.31876649   -1.08237463    0.00000000   -35.21764149
     0.00000000   -0.70208822    0.00000000   116.59014179
     2.11091906    0.04406596    0.00000000    16.10038397
     1.32524096    1.15754064    0.00000000   128.77205511
    -0.04178899    0.70146698    0.00000000  -214.63021956
    -1.16182154   -1.48163476    0.00000000   -23.23469515
    -2.37713566   -0.81881509    0.00000000    40.30522064
    -2.44031774    0.59060577    0.00000000   -38.57316928
    -1.28611065    1.35334636    0.00000000    94.98745698
     1.26584879   -1.08237463    0.00000000     4.26413660
     1.37168419   -1.08237463    0.00000000     4.30721009
     1.31876649   -1.13529233    0.00000000    13.09441453
     1.31876649   -1.02945693    0.00000000    13.74148785
    -0.05291770   -0.70208822    0.00000000   -27.51464974
     0.05291770   -0.70208822    0.00000000   -24.45827143
     0.00000000   -0.75500592    0.00000000   -32.98590892
     0.00000000   -0.64917052    0.00000000   -31.95466055
     2.05800137    0.04406596    0.00000000     5.48783158
     2.16383676    0.04406596    0.00000000     5.22632220
     2.11091906   -0.00885174    0.00000000   -17.13642361
     2.11091906    0.09698366    0.00000000    -9.77472935
     1.27232327    1.15754064    0.00000000   -35.16665930
     1.37815866    1.15754064    0.00000000   -33.84734741
     1.32524096    1.10462294    0.00000000   -31.52226749
     1.32524096    1.21045834    0.00000000   -28.48565370
    -0.09470669    0.70146698    0.00000000    86.02566219
     0.01112871    0.70146698    0.00000000    80.37531877
    -0.04178899    0.64854929    0.00000000    22.63816566
    -0.04178899    0.75438469    0.00000000    25.99273495
    -1.21473924   -1.48163476    0.00000000     0.90195009
    -1.10890384   -1.48163476    0.00000000     2.48792873
    -1.16182154   -1.53455246    0.00000000     7.89902547
    -1.16182154   -1.42871706    0.00000000    12.49241483
    -2.43005336   -0.81881509    0.00000000   -24.65271492
    -2.32421796   -0.81881509    0.00000000   -28.79447811
    -2.37713566   -0.87173279    0.00000000     5.98659140
    -2.37713566   -0.76589740    0.00000000     6.88626451
    -2.49323545    0.59060577    0.00000000     4.24160103
    -2.38740004    0.59060577    0.00000000     8.46462112
    -2.44031774    0.53768808    0.00000000    14.00041863
    -2.44031774    0.64352347    0.00000000    12.38505452
    -1.33902835    1.35334636    0.00000000   -25.17574190
    -1.23319294    1.35334636    0.00000000   -25.43805798
    -1.28611065    1.30042866    0.00000000   -25.52074144
    -1.28611065    1.40626406    0.00000000   -19.57038194
&TRANSITION 4->...
0    0.0   # (r2v4r2v3) 45
     0.00000000    0.00000000    0.00000000 1.0    # electr. mom.
     0.00000000    0.00000000    0.00000000        # magnet. mom.
0    0.0   # (r2v5r2v3) 45
     0.00000000    0.00000000    0.00000000 1.0    # electr. mom.
     0.00000000    0.00000000    0.00000000        # magnet. mom.
0    0.0   # (r2v6r2v3) 45
     0.00000000    0.00000000    0.00000000 1.0    # electr. mom.
     0.00000000    0.00000000    0.00000000        # magnet. mom.
0    0.0   # (r2v7r2v3) 45
     0.00000000    0.00000000    0.00000000 1.0    # electr. mom.
     0.00000000    0.00000000    0.00000000        # magnet. mom.
45  2657.0   # 737.7 nm,   (r3v1r2v3) 45
     0.094868    0.180761    0.00000000 1.0   # electr. mom.
     0.00000000    0.00000000    -1.664952771     # magnet. mom.
     1.31876649   -1.08237463    0.00000000  -284.28836986
     0.00000000   -0.70208822    0.00000000   526.93340465
     2.11091906    0.04406596    0.00000000    83.91786452
     1.32524096    1.15754064    0.00000000  -319.64910145
    -0.04178899    0.70146698    0.00000000   559.44544345
    -1.16182154   -1.48163476    0.00000000  -174.04992879
    -2.37713566   -0.81881509    0.00000000    27.27537191
    -2.44031774    0.59060577    0.00000000    26.38022103
    -1.28611065    1.35334636    0.00000000  -311.82348864
     1.26584879   -1.08237463    0.00000000    72.87120803
     1.37168419   -1.08237463    0.00000000    62.56852754
     1.31876649   -1.13529233    0.00000000    67.92422816
     1.31876649   -1.02945693    0.00000000    82.81728915
    -0.05291770   -0.70208822    0.00000000  -128.16236441
     0.05291770   -0.70208822    0.00000000  -133.46267444
     0.00000000   -0.75500592    0.00000000  -134.71083482
     0.00000000   -0.64917052    0.00000000  -133.42085553
     2.05800137    0.04406596    0.00000000   -24.80406971
```

```
    2.16383676       0.04406596       0.00000000     -12.53532628
    2.11091906      -0.00885174       0.00000000     -21.97214106
    2.11091906       0.09698366       0.00000000     -26.11479878
    1.27232327       1.15754064       0.00000000      94.81184909
    1.37815866       1.15754064       0.00000000      83.97709047
    1.32524096       1.10462294       0.00000000      80.87748624
    1.32524096       1.21045834       0.00000000      62.52322301
   -0.09470669       0.70146698       0.00000000    -267.92935492
    0.01112871       0.70146698       0.00000000    -271.74270492
   -0.04178899       0.64854929       0.00000000      -2.55473667
   -0.04178899       0.75438469       0.00000000     -19.55429073
   -1.21473924      -1.48163476       0.00000000      55.30762317
   -1.10890384      -1.48163476       0.00000000      61.36503613
   -1.16182154      -1.53455246       0.00000000      26.00173815
   -1.16182154      -1.42871706       0.00000000      32.47548981
   -2.43005336      -0.81881509       0.00000000      -8.84412431
   -2.32421796      -0.81881509       0.00000000     -11.75525367
   -2.37713566      -0.87173279       0.00000000      -3.61289028
   -2.37713566      -0.76589740       0.00000000      -3.32520753
   -2.49323545       0.59060577       0.00000000     -20.76008692
   -2.38740004       0.59060577       0.00000000     -27.45042173
   -2.44031774       0.53768808       0.00000000      10.80365856
   -2.44031774       0.64352347       0.00000000      10.49724496
   -1.33902835       1.35334636       0.00000000      58.29229871
   -1.23319294       1.35334636       0.00000000      63.90419085
   -1.28611065       1.30042866       0.00000000     104.98630892
   -1.28611065       1.40626406       0.00000000      86.56622889
45    2657.0    # 737.7 nm,    (r3v2r2v3) 45
    0.088211         0.168076         0.00000000 1.0     # electr. mom.
    0.00000000       0.00000000      -1.664952771        # magnet. mom.
    1.31876649      -1.08237463       0.00000000    -264.33830881
    0.00000000      -0.70208822       0.00000000     489.95562187
    2.11091906       0.04406596       0.00000000      78.02889157
    1.32524096       1.15754064       0.00000000    -297.21758556
   -0.04178899       0.70146698       0.00000000     520.18611409
   -1.16182154      -1.48163476       0.00000000    -161.83589870
   -2.37713566      -0.81881509       0.00000000      25.36131072
   -2.44031774       0.59060577       0.00000000      24.52897745
   -1.28611065       1.35334636       0.00000000    -289.94113856
    1.26584879      -1.08237463       0.00000000      67.75743905
    1.37168419      -1.08237463       0.00000000      58.17775367
    1.31876649      -1.13529233       0.00000000      63.15761565
    1.31876649      -1.02945693       0.00000000      77.00554956
   -0.05291770      -0.70208822       0.00000000    -119.16851428
    0.05291770      -0.70208822       0.00000000    -124.09687272
    0.00000000      -0.75500592       0.00000000    -125.25744290
    0.00000000      -0.64917052       0.00000000    -124.05798848
    2.05800137       0.04406596       0.00000000     -23.06343324
    2.16383676       0.04406596       0.00000000     -11.65565426
    2.11091906      -0.00885174       0.00000000     -20.43023642
    2.11091906       0.09698366       0.00000000     -24.28218132
    1.27232327       1.15754064       0.00000000      88.15838599
    1.37815866       1.15754064       0.00000000      78.08396132
    1.32524096       1.10462294       0.00000000      75.20187317
    1.32524096       1.21045834       0.00000000      58.13562842
   -0.09470669       0.70146698       0.00000000    -249.12729492
    0.01112871       0.70146698       0.00000000    -252.67304142
   -0.04178899       0.64854929       0.00000000      -2.37545690
   -0.04178899       0.75438469       0.00000000     -18.18205980
   -1.21473924      -1.48163476       0.00000000      51.42638645
   -1.10890384      -1.48163476       0.00000000      57.05871780
   -1.16182154      -1.53455246       0.00000000      24.17705477
   -1.16182154      -1.42871706       0.00000000      30.19650807
   -2.43005336      -0.81881509       0.00000000      -8.22348401
   -2.32421796      -0.81881509       0.00000000     -10.93032359
   -2.37713566      -0.87173279       0.00000000      -3.35935412
   -2.37713566      -0.76589740       0.00000000      -3.09185963
   -2.49323545       0.59060577       0.00000000     -19.30323872
   -2.38740004       0.59060577       0.00000000     -25.52407634
   -2.44031774       0.53768808       0.00000000      10.04550708
   -2.44031774       0.64352347       0.00000000       9.76059619
   -1.33902835       1.35334636       0.00000000      54.20161108
   -1.23319294       1.35334636       0.00000000      59.41968623
   -1.28611065       1.30042866       0.00000000      97.61884864
   -1.28611065       1.40626406       0.00000000      80.49140581
45    2657.0    # 737.7 nm,    (r3v3r2v3) 45
    0.053259         0.10148          0.00000000 1.0     # electr. mom.
    0.00000000       0.00000000      -1.664952771        # magnet. mom.
    1.31876649      -1.08237463       0.00000000    -159.60048834
    0.00000000      -0.70208822       0.00000000     295.82226226
    2.11091906       0.04406596       0.00000000      47.11178359
    1.32524096       1.15754064       0.00000000    -179.45212713
   -0.04178899       0.70146698       0.00000000     314.07463492
   -1.16182154      -1.48163476       0.00000000     -97.71224072
   -2.37713566      -0.81881509       0.00000000      15.31248949
   -2.44031774       0.59060577       0.00000000      14.80994865
   -1.28611065       1.35334636       0.00000000    -175.05880064
```

```
1.26584879  -1.08237463  0.00000000  40.91015188
1.37168419  -1.08237463  0.00000000  35.12619090
1.31876649  -1.13529233  0.00000000  38.13290002
1.31876649  -1.02945693  0.00000000  46.49391671
-0.05291770  -0.70208822  0.00000000  -71.95080107
0.05291770  -0.70208822  0.00000000  -74.92641372
0.00000000  -0.75500592  0.00000000  -75.62713534
0.00000000  -0.64917052  0.00000000  -74.90293644
2.05800137  0.04406596  0.00000000  -13.92509177
2.16383676  0.04406596  0.00000000  -7.03737616
2.11091906  -0.00885174  0.00000000  -12.33523709
2.11091906  0.09698366  0.00000000  -14.66093967
1.27232327  1.15754064  0.00000000  53.22770475
1.37815866  1.15754064  0.00000000  47.14503325
1.32524096  1.10462294  0.00000000  45.40490456
1.32524096  1.21045834  0.00000000  35.10075678
-0.09470669  0.70146698  0.00000000  -150.41647995
0.01112871  0.70146698  0.00000000  -152.55730802
-0.04178899  0.64854929  0.00000000  -1.43423813
-0.04178899  0.75438469  0.00000000  -10.97784743
-1.21473924  -1.48163476  0.00000000  31.04989371
-1.10890384  -1.48163476  0.00000000  34.45054660
-1.16182154  -1.53455246  0.00000000  14.59746703
-1.16182154  -1.42871706  0.00000000  18.23185393
-2.43005336  -0.81881509  0.00000000  -4.96512242
-2.32421796  -0.81881509  0.00000000  -6.59944066
-2.37713566  -0.87173279  0.00000000  -2.02828928
-2.37713566  -0.76589740  0.00000000  -1.86678317
-2.49323545  0.59060577  0.00000000  -11.65478564
-2.38740004  0.59060577  0.00000000  -15.41076307
-2.44031774  0.53768808  0.00000000  6.06521182
-2.44031774  0.64352347  0.00000000  5.89319015
-1.33902835  1.35334636  0.00000000  32.72550103
-1.23319294  1.35334636  0.00000000  35.87603697
-1.28611065  1.30042866  0.00000000  58.93968220
-1.28611065  1.40626406  0.00000000  48.59858464
45  2657.0  # 737.7 nm,  (r3v4r2v3) 45
0.056588  0.107822  0.00000000 1.0  # electr. mom.
0.00000000  0.00000000  -1.664952771  # magnet. mom.
1.31876649  -1.08237463  0.00000000  -169.57551886
0.00000000  -0.70208822  0.00000000  314.31115365
2.11091906  0.04406596  0.00000000  50.05627007
1.32524096  1.15754064  0.00000000  -190.66788508
-0.04178899  0.70146698  0.00000000  333.70429960
-1.16182154  -1.48163476  0.00000000  -103.81925577
-2.37713566  -0.81881509  0.00000000  16.26952008
-2.44031774  0.59060577  0.00000000  15.73557044
-1.28611065  1.35334636  0.00000000  -185.99997568
1.26584879  -1.08237463  0.00000000  43.46703637
1.37168419  -1.08237463  0.00000000  37.32157783
1.31876649  -1.13529233  0.00000000  40.51620627
1.31876649  -1.02945693  0.00000000  49.39978651
-0.05291770  -0.70208822  0.00000000  -76.44772614
0.05291770  -0.70208822  0.00000000  -79.60931458
0.00000000  -0.75500592  0.00000000  -80.35383130
0.00000000  -0.64917052  0.00000000  -79.58436997
2.05800137  0.04406596  0.00000000  -14.79541000
2.16383676  0.04406596  0.00000000  -7.47721217
2.11091906  -0.00885174  0.00000000  -13.10618940
2.11091906  0.09698366  0.00000000  -15.57724840
1.27232327  1.15754064  0.00000000  56.55443630
1.37815866  1.15754064  0.00000000  50.09159783
1.32524096  1.10462294  0.00000000  48.24271109
1.32524096  1.21045834  0.00000000  37.29455408
-0.09470669  0.70146698  0.00000000  -159.81750995
0.01112871  0.70146698  0.00000000  -162.09213978
-0.04178899  0.64854929  0.00000000  -1.52387801
-0.04178899  0.75438469  0.00000000  -11.66396289
-1.21473924  -1.48163476  0.00000000  32.99051206
-1.10890384  -1.48163476  0.00000000  36.60370576
-1.16182154  -1.53455246  0.00000000  15.50980872
-1.16182154  -1.42871706  0.00000000  19.37134480
-2.43005336  -0.81881509  0.00000000  -5.27544257
-2.32421796  -0.81881509  0.00000000  -7.01190570
-2.37713566  -0.87173279  0.00000000  -2.15505736
-2.37713566  -0.76589740  0.00000000  -1.98345712
-2.49323545  0.59060577  0.00000000  -12.38320974
-2.38740004  0.59060577  0.00000000  -16.37393577
-2.44031774  0.53768808  0.00000000  6.44428756
-2.44031774  0.64352347  0.00000000  6.26151454
-1.33902835  1.35334636  0.00000000  34.77084484
-1.23319294  1.35334636  0.00000000  38.11828928
-1.28611065  1.30042866  0.00000000  62.62341234
-1.28611065  1.40626406  0.00000000  51.63599618
45  2657.0  # 737.7 nm,  (r3v5r2v3) 45
0.049931  0.095137  0.00000000 1.0  # electr. mom.
0.00000000  0.00000000  -1.664952771  # magnet. mom.
```

1.31876649	-1.08237463	0.00000000	-149.62545782
0.00000000	-0.70208822	0.00000000	277.33337087
2.11091906	0.04406596	0.00000000	44.16729712
1.32524096	1.15754064	0.00000000	-168.23636919
-0.04178899	0.70146698	0.00000000	294.44497024
-1.16182154	-1.48163476	0.00000000	-91.60522568
-2.37713566	-0.81881509	0.00000000	14.35545890
-2.44031774	0.59060577	0.00000000	13.88432686
-1.28611065	1.35334636	0.00000000	-164.11762560
1.26584879	-1.08237463	0.00000000	38.35326738
1.37168419	-1.08237463	0.00000000	32.93080397
1.31876649	-1.13529233	0.00000000	35.74959377
1.31876649	-1.02945693	0.00000000	43.58804692
-0.05291770	-0.70208822	0.00000000	-67.45387601
0.05291770	-0.70208822	0.00000000	-70.24351286
0.00000000	-0.75500592	0.00000000	-70.90043938
0.00000000	-0.64917052	0.00000000	-70.22150291
2.05800137	0.04406596	0.00000000	-13.05477353
2.16383676	0.04406596	0.00000000	-6.59754015
2.11091906	-0.00885174	0.00000000	-11.56428477
2.11091906	0.09698366	0.00000000	-13.74463094
1.27232327	1.15754064	0.00000000	49.90097320
1.37815866	1.15754064	0.00000000	44.19846867
1.32524096	1.10462294	0.00000000	42.56709802
1.32524096	1.21045834	0.00000000	32.90695948
-0.09470669	0.70146698	0.00000000	-141.01544996
0.01112871	0.70146698	0.00000000	-143.02247627
-0.04178899	0.64854929	0.00000000	-1.34459824
-0.04178899	0.75438469	0.00000000	-10.29173196
-1.21473924	-1.48163476	0.00000000	29.10927535
-1.10890384	-1.48163476	0.00000000	32.29738744
-1.16182154	-1.53455246	0.00000000	13.68512534
-1.16182154	-1.42871706	0.00000000	17.09236306
-2.43005336	-0.81881509	0.00000000	-4.65480227
-2.32421796	-0.81881509	0.00000000	-6.18697561
-2.37713566	-0.87173279	0.00000000	-1.90152120
-2.37713566	-0.76589740	0.00000000	-1.75010923
-2.49323545	0.59060577	0.00000000	-10.92636154
-2.38740004	0.59060577	0.00000000	-14.44759038
-2.44031774	0.53768808	0.00000000	5.68613608
-2.44031774	0.64352347	0.00000000	5.52486577
-1.33902835	1.35334636	0.00000000	30.68015721
-1.23319294	1.35334636	0.00000000	33.63378466
-1.28611065	1.30042866	0.00000000	55.25595206
-1.28611065	1.40626406	0.00000000	45.56117310

45 2657.0 # 737.7 nm, (r3v6r2v3) 45

0.029958	0.057082	0.00000000	1.0	# electr. mom.
0.00000000	0.00000000	-1.664952771		# magnet. mom.
1.31876649	-1.08237463	0.00000000	-89.77527469	
0.00000000	-0.70208822	0.00000000	166.40002252	
2.11091906	0.04406596	0.00000000	26.50037827	
1.32524096	1.15754064	0.00000000	-100.94182151	
-0.04178899	0.70146698	0.00000000	176.66698214	
-1.16182154	-1.48163476	0.00000000	-54.96313541	
-2.37713566	-0.81881509	0.00000000	8.61327534	
-2.44031774	0.59060577	0.00000000	8.33059611	
-1.28611065	1.35334636	0.00000000	-98.47057536	
1.26584879	-1.08237463	0.00000000	23.01196043	
1.37168419	-1.08237463	0.00000000	19.75848238	
1.31876649	-1.13529233	0.00000000	21.44975626	
1.31876649	-1.02945693	0.00000000	26.15282815	
-0.05291770	-0.70208822	0.00000000	-40.47232560	
0.05291770	-0.70208822	0.00000000	-42.14610772	
0.00000000	-0.75500592	0.00000000	-42.54026363	
0.00000000	-0.64917052	0.00000000	-42.13290175	
2.05800137	0.04406596	0.00000000	-7.83286412	
2.16383676	0.04406596	0.00000000	-3.95852409	
2.11091906	-0.00885174	0.00000000	-6.93857086	
2.11091906	0.09698366	0.00000000	-8.24677856	
1.27232327	1.15754064	0.00000000	29.94058392	
1.37815866	1.15754064	0.00000000	26.51908120	
1.32524096	1.10462294	0.00000000	25.54025881	
1.32524096	1.21045834	0.00000000	19.74417569	
-0.09470669	0.70146698	0.00000000	-84.60926997	
0.01112871	0.70146698	0.00000000	-85.81348576	
-0.04178899	0.64854929	0.00000000	-0.80675895	
-0.04178899	0.75438469	0.00000000	-6.17503918	
-1.21473924	-1.48163476	0.00000000	17.46556521	
-1.10890384	-1.48163476	0.00000000	19.37843246	
-1.16182154	-1.53455246	0.00000000	8.21107520	
-1.16182154	-1.42871706	0.00000000	10.25541784	
-2.43005336	-0.81881509	0.00000000	-2.79288136	
-2.32421796	-0.81881509	0.00000000	-3.71218537	
-2.37713566	-0.87173279	0.00000000	-1.14091272	
-2.37713566	-0.76589740	0.00000000	-1.05006554	
-2.49323545	0.59060577	0.00000000	-6.55581692	
-2.38740004	0.59060577	0.00000000	-8.66855423	

```
   -2.44031774      0.53768808      0.00000000       3.41168165
   -2.44031774      0.64352347      0.00000000       3.31491946
   -1.33902835      1.35334636      0.00000000      18.40809433
   -1.23319294      1.35334636      0.00000000      20.18027080
   -1.28611065      1.30042866      0.00000000      33.15357124
   -1.28611065      1.40626406      0.00000000      27.33670386
45    2657.0    # 737.7 nm,    (r3v7r2v3) 45
    0.031623        0.060254        0.00000000 1.0     # electr. mom.
    0.00000000      0.00000000     -1.664952771          # magnet. mom.
    1.31876649     -1.08237463      0.00000000     -94.76278995
    0.00000000     -0.70208822      0.00000000     175.64446822
    2.11091906      0.04406596      0.00000000      27.97262151
    1.32524096      1.15754064      0.00000000    -106.54970048
   -0.04178899      0.70146698      0.00000000     186.48181448
   -1.16182154     -1.48163476      0.00000000     -58.01664293
   -2.37713566     -0.81881509      0.00000000       9.09179064
   -2.44031774      0.59060577      0.00000000       8.79340701
   -1.28611065      1.35334636      0.00000000    -103.94116288
    1.26584879     -1.08237463      0.00000000      24.29040268
    1.37168419     -1.08237463      0.00000000      20.85617585
    1.31876649     -1.13529233      0.00000000      22.64140939
    1.31876649     -1.02945693      0.00000000      27.60576305
   -0.05291770     -0.70208822      0.00000000     -42.72078814
    0.05291770     -0.70208822      0.00000000     -44.48755815
    0.00000000     -0.75500592      0.00000000     -44.90361161
    0.00000000     -0.64917052      0.00000000     -44.47361851
    2.05800137      0.04406596      0.00000000      -8.26802324
    2.16383676      0.04406596      0.00000000      -4.17844209
    2.11091906     -0.00885174      0.00000000      -7.32404702
    2.11091906      0.09698366      0.00000000      -8.70493293
    1.27232327      1.15754064      0.00000000      31.60394970
    1.37815866      1.15754064      0.00000000      27.99236349
    1.32524096      1.10462294      0.00000000      26.95916208
    1.32524096      1.21045834      0.00000000      20.84107434
   -0.09470669      0.70146698      0.00000000     -89.30978497
    0.01112871      0.70146698      0.00000000     -90.58090164
   -0.04178899      0.64854929      0.00000000      -0.85157889
   -0.04178899      0.75438469      0.00000000      -6.51809691
   -1.21473924     -1.48163476      0.00000000      18.43587439
   -1.10890384     -1.48163476      0.00000000      20.45501204
   -1.16182154     -1.53455246      0.00000000       8.66724605
   -1.16182154     -1.42871706      0.00000000      10.82516327
   -2.43005336     -0.81881509      0.00000000      -2.94804144
   -2.32421796     -0.81881509      0.00000000      -3.91841789
   -2.37713566     -0.87173279      0.00000000      -1.20429676
   -2.37713566     -0.76589740      0.00000000      -1.10840251
   -2.49323545      0.59060577      0.00000000      -6.92002897
   -2.38740004      0.59060577      0.00000000      -9.15014058
   -2.44031774      0.53768808      0.00000000       3.60121952
   -2.44031774      0.64352347      0.00000000       3.49908165
   -1.33902835      1.35334636      0.00000000      19.43076624
   -1.23319294      1.35334636      0.00000000      21.30139695
   -1.28611065      1.30042866      0.00000000      34.99543631
   -1.28611065      1.40626406      0.00000000      28.85540963
45    2657.0    # 737.7 nm,    (r3v8r2v3) 45
    0.013315        0.02537         0.00000000 1.0     # electr. mom.
    0.00000000      0.00000000     -1.664952771          # magnet. mom.
    1.31876649     -1.08237463      0.00000000     -39.90012209
    0.00000000     -0.70208822      0.00000000      73.95556556
    2.11091906      0.04406596      0.00000000      11.77794590
    1.32524096      1.15754064      0.00000000     -44.86303178
   -0.04178899      0.70146698      0.00000000      78.51865873
   -1.16182154     -1.48163476      0.00000000     -24.42806018
   -2.37713566     -0.81881509      0.00000000       3.82812237
   -2.44031774      0.59060577      0.00000000       3.70248716
   -1.28611065      1.35334636      0.00000000     -43.76470016
    1.26584879     -1.08237463      0.00000000      10.22753797
    1.37168419     -1.08237463      0.00000000       8.78154772
    1.31876649     -1.13529233      0.00000000       9.53322500
    1.31876649     -1.02945693      0.00000000      11.62347918
   -0.05291770     -0.70208822      0.00000000     -17.98770027
    0.05291770     -0.70208822      0.00000000     -18.73160343
    0.00000000     -0.75500592      0.00000000     -18.90678383
    0.00000000     -0.64917052      0.00000000     -18.72573411
    2.05800137      0.04406596      0.00000000      -3.48127294
    2.16383676      0.04406596      0.00000000      -1.75934404
    2.11091906     -0.00885174      0.00000000      -3.08380927
    2.11091906      0.09698366      0.00000000      -3.66523492
    1.27232327      1.15754064      0.00000000      13.30692619
    1.37815866      1.15754064      0.00000000      11.78625831
    1.32524096      1.10462294      0.00000000      11.35122614
    1.32524096      1.21045834      0.00000000       8.77518919
   -0.09470669      0.70146698      0.00000000     -37.60411999
    0.01112871      0.70146698      0.00000000     -38.13932701
   -0.04178899      0.64854929      0.00000000      -0.35855953
   -0.04178899      0.75438469      0.00000000      -2.74446186
   -1.21473924     -1.48163476      0.00000000       7.76247343
```

-1.10890384	-1.48163476	0.00000000	8.61263665
-1.16182154	-1.53455246	0.00000000	3.64936676
-1.16182154	-1.42871706	0.00000000	4.55796348
-2.43005336	-0.81881509	0.00000000	-1.24128060
-2.32421796	-0.81881509	0.00000000	-1.64986016
-2.37713566	-0.87173279	0.00000000	-0.50707232
-2.37713566	-0.76589740	0.00000000	-0.46669579
-2.49323545	0.59060577	0.00000000	-2.91369641
-2.38740004	0.59060577	0.00000000	-3.85269077
-2.44031774	0.53768808	0.00000000	1.51630296
-2.44031774	0.64352347	0.00000000	1.47329754
-1.33902835	1.35334636	0.00000000	8.18137526
-1.23319294	1.35334636	0.00000000	8.96900924
-1.28611065	1.30042866	0.00000000	14.73492055
-1.28611065	1.40626406	0.00000000	12.14964616

45 2657.0 # 737.7 nm, (r3v9r2v3) 45

0.016644	0.031712	0.00000000 1.0	# electr. mom.
0.00000000	0.00000000	-1.664952771	# magnet. mom.
1.31876649	-1.08237463	0.00000000	-49.87515261
0.00000000	-0.70208822	0.00000000	92.44445696
2.11091906	0.04406596	0.00000000	14.72243237
1.32524096	1.15754064	0.00000000	-56.07878973
-0.04178899	0.70146698	0.00000000	98.14832341
-1.16182154	-1.48163476	0.00000000	-30.53507523
-2.37713566	-0.81881509	0.00000000	4.78515297
-2.44031774	0.59060577	0.00000000	4.62810895
-1.28611065	1.35334636	0.00000000	-54.70587520
1.26584879	-1.08237463	0.00000000	12.78442246
1.37168419	-1.08237463	0.00000000	10.97693466
1.31876649	-1.13529233	0.00000000	11.91653126
1.31876649	-1.02945693	0.00000000	14.52934897
-0.05291770	-0.70208822	0.00000000	-22.48462534
0.05291770	-0.70208822	0.00000000	-23.41450429
0.00000000	-0.75500592	0.00000000	-23.63347979
0.00000000	-0.64917052	0.00000000	-23.40716764
2.05800137	0.04406596	0.00000000	-4.35159118
2.16383676	0.04406596	0.00000000	-2.19918005
2.11091906	-0.00885174	0.00000000	-3.85476159
2.11091906	0.09698366	0.00000000	-4.58154365
1.27232327	1.15754064	0.00000000	16.63365773
1.37815866	1.15754064	0.00000000	14.73282289
1.32524096	1.10462294	0.00000000	14.18903267
1.32524096	1.21045834	0.00000000	10.96898649
-0.09470669	0.70146698	0.00000000	-47.00514999
0.01112871	0.70146698	0.00000000	-47.67415876
-0.04178899	0.64854929	0.00000000	-0.44819941
-0.04178899	0.75438469	0.00000000	-3.43057732
-1.21473924	-1.48163476	0.00000000	9.70309178
-1.10890384	-1.48163476	0.00000000	10.76579581
-1.16182154	-1.53455246	0.00000000	4.56170845
-1.16182154	-1.42871706	0.00000000	5.69745435
-2.43005336	-0.81881509	0.00000000	-1.55160076
-2.32421796	-0.81881509	0.00000000	-2.06232520
-2.37713566	-0.87173279	0.00000000	-0.63384040
-2.37713566	-0.76589740	0.00000000	-0.58336974
-2.49323545	0.59060577	0.00000000	-3.64212051
-2.38740004	0.59060577	0.00000000	-4.81586346
-2.44031774	0.53768808	0.00000000	1.89537869
-2.44031774	0.64352347	0.00000000	1.84162192
-1.33902835	1.35334636	0.00000000	10.22671907
-1.23319294	1.35334636	0.00000000	11.21126155
-1.28611065	1.30042866	0.00000000	18.41865069
-1.28611065	1.40626406	0.00000000	15.18705770

45 13555.0 # 737.7 nm, (r7r2v3) 45

0.229699	-0.05388	0.00000000 1.0	# electr. mom.
0.00000000	0.00000000	-0.8485163126	# magnet. mom.
1.31876649	-1.08237463	0.00000000	15.55211674
0.00000000	-0.70208822	0.00000000	7.52674425
2.11091906	0.04406596	0.00000000	6.66274380
1.32524096	1.15754064	0.00000000	-9.63455182
-0.04178899	0.70146698	0.00000000	84.61940208
-1.16182154	-1.48163476	0.00000000	-88.89954030
-2.37713566	-0.81881509	0.00000000	50.40527558
-2.44031774	0.59060577	0.00000000	-21.92062275
-1.28611065	1.35334636	0.00000000	-39.49228424
1.26584879	-1.08237463	0.00000000	-4.78170756
1.37168419	-1.08237463	0.00000000	-3.40628756
1.31876649	-1.13529233	0.00000000	-2.90260824
1.31876649	-1.02945693	0.00000000	-4.72662426
-0.05291770	-0.70208822	0.00000000	32.87694548
0.05291770	-0.70208822	0.00000000	33.79465717
0.00000000	-0.75500592	0.00000000	-34.56553463
0.00000000	-0.64917052	0.00000000	-39.56436938
2.05800137	0.04406596	0.00000000	-4.52916201
2.16383676	0.04406596	0.00000000	-4.37592864
2.11091906	-0.00885174	0.00000000	0.56034898
2.11091906	0.09698366	0.00000000	1.80227797

1.27232327 1.15754064 0.00000000 -11.97009965
1.37815866 1.15754064 0.00000000 -10.36753931
1.32524096 1.10462294 0.00000000 17.20418796
1.32524096 1.21045834 0.00000000 14.72773269
-0.09470669 0.70146698 0.00000000 -43.31987669
0.01112871 0.70146698 0.00000000 -45.61581687
-0.04178899 0.64854929 0.00000000 1.65895490
-0.04178899 0.75438469 0.00000000 3.08532424
-1.21473924 -1.48163476 0.00000000 31.26641346
-1.10890384 -1.48163476 0.00000000 32.41735432
-1.16182154 -1.53455246 0.00000000 12.55771976
-1.16182154 -1.42871706 0.00000000 12.46017510
-2.43005336 -0.81881509 0.00000000 -13.11021734
-2.32421796 -0.81881509 0.00000000 -13.92024066
-2.37713566 -0.87173279 0.00000000 -11.62094191
-2.37713566 -0.76589740 0.00000000 -11.53654428
-2.49323545 0.59060577 0.00000000 11.27112334
-2.38740004 0.59060577 0.00000000 13.32830512
-2.44031774 0.53768808 0.00000000 -2.65520319
-2.44031774 0.64352347 0.00000000 -0.07123185
-1.33902835 1.35334636 0.00000000 4.40164668
-1.23319294 1.35334636 0.00000000 4.08370491
-1.28611065 1.30042866 0.00000000 15.74626779
-1.28611065 1.40626406 0.00000000 14.97751082
45 15636.0 # 639.5 nm, (r8r2v3) 45
-0.60375 0.086595 0.00000000 1.0 # electr. mom.
0.00000000 0.00000000 -0.9999114666E-01 # magnet. mom.
1.31876649 -1.08237463 0.00000000 -32.96970693
0.00000000 -0.70208822 0.00000000 109.14821785
2.11091906 0.04406596 0.00000000 15.07269988
1.32524096 1.15754064 0.00000000 120.55256223
-0.04178899 0.70146698 0.00000000 -200.93041831
-1.16182154 -1.48163476 0.00000000 -21.75162950
-2.37713566 -0.81881509 0.00000000 37.73254698
-2.44031774 0.59060577 0.00000000 -36.11105209
-1.28611065 1.35334636 0.00000000 88.92442781
1.26584879 -1.08237463 0.00000000 3.99195767
1.37168419 -1.08237463 0.00000000 4.03228178
1.31876649 -1.13529233 0.00000000 12.25860084
1.31876649 -1.02945693 0.00000000 12.86437160
-0.05291770 -0.70208822 0.00000000 -25.75839550
0.05291770 -0.70208822 0.00000000 -22.89710517
0.00000000 -0.75500592 0.00000000 -30.88042537
0.00000000 -0.64917052 0.00000000 -29.91500137
2.05800137 0.04406596 0.00000000 5.13754446
2.16383676 0.04406596 0.00000000 4.89272716
2.11091906 -0.00885174 0.00000000 -16.04260933
2.11091906 0.09698366 0.00000000 -9.15081045
1.27232327 1.15754064 0.00000000 -32.92197892
1.37815866 1.15754064 0.00000000 -31.68687842
1.32524096 1.10462294 0.00000000 -29.51020787
1.32524096 1.21045834 0.00000000 -26.66742049
-0.09470669 0.70146698 0.00000000 80.53466248
0.01112871 0.70146698 0.00000000 75.24497927
-0.04178899 0.64854929 0.00000000 21.19317636
-0.04178899 0.75438469 0.00000000 24.33362421
-1.21473924 -1.48163476 0.00000000 0.84437881
-1.10890384 -1.48163476 0.00000000 2.32912477
-1.16182154 -1.53455246 0.00000000 7.39483236
-1.16182154 -1.42871706 0.00000000 11.69502665
-2.43005336 -0.81881509 0.00000000 -23.07913737
-2.32421796 -0.81881509 0.00000000 -26.95653270
-2.37713566 -0.87173279 0.00000000 5.60446854
-2.37713566 -0.76589740 0.00000000 6.44671571
-2.49323545 0.59060577 0.00000000 3.97086054
-2.38740004 0.59060577 0.00000000 7.92432616
-2.44031774 0.53768808 0.00000000 13.10677489
-2.44031774 0.64352347 0.00000000 11.59451912
-1.33902835 1.35334636 0.00000000 -23.56877965
-1.23319294 1.35334636 0.00000000 -23.81435215
-1.28611065 1.30042866 0.00000000 -23.89175794
-1.28611065 1.40626406 0.00000000 -18.32120862
&TRANSITION 5->...
0 0.0 # (r2v5r2v4) 45
0.00000000 0.00000000 0.00000000 1.0 # electr. mom.
0.00000000 0.00000000 0.00000000 # magnet. mom.
0 0.0 # (r2v6r2v4) 45
0.00000000 0.00000000 0.00000000 1.0 # electr. mom.
0.00000000 0.00000000 0.00000000 # magnet. mom.
0 0.0 # (r2v7r2v4) 45
0.00000000 0.00000000 0.00000000 1.0 # electr. mom.
0.00000000 0.00000000 0.00000000 # magnet. mom.
45 2657.0 # 737.7 nm, (r3v1r2v4) 45
0.04959 0.094489 0.00000000 1.0 # electr. mom.
0.00000000 0.00000000 -1.664952771 # magnet. mom.
1.31876649 -1.08237463 0.00000000 -148.60528424
0.00000000 -0.70208822 0.00000000 275.44246152

2.11091906	0.04406596	0.00000000	43.86615645
1.32524096	1.15754064	0.00000000	-167.08930303
-0.04178899	0.70146698	0.00000000	292.43739089
-1.16182154	-1.48163476	0.00000000	-90.98064460
-2.37713566	-0.81881509	0.00000000	14.25758077
-2.44031774	0.59060577	0.00000000	13.78966099
-1.28611065	1.35334636	0.00000000	-162.99864179
1.26584879	-1.08237463	0.00000000	38.09176783
1.37168419	-1.08237463	0.00000000	32.70627576
1.31876649	-1.13529233	0.00000000	35.50584654
1.31876649	-1.02945693	0.00000000	43.29085569
-0.05291770	-0.70208822	0.00000000	-66.99396321
0.05291770	-0.70208822	0.00000000	-69.76457982
0.00000000	-0.75500592	0.00000000	-70.41702729
0.00000000	-0.64917052	0.00000000	-69.74271994
2.05800137	0.04406596	0.00000000	-12.96576371
2.16383676	0.04406596	0.00000000	-6.55255692
2.11091906	-0.00885174	0.00000000	-11.48543737
2.11091906	0.09698366	0.00000000	-13.65091754
1.27232327	1.15754064	0.00000000	49.56073929
1.37815866	1.15754064	0.00000000	43.89711548
1.32524096	1.10462294	0.00000000	42.27686781
1.32524096	1.21045834	0.00000000	32.68259385
-0.09470669	0.70146698	0.00000000	-140.05398098
0.01112871	0.70146698	0.00000000	-142.04732303
-0.04178899	0.64854929	0.00000000	-1.33543053
-0.04178899	0.75438469	0.00000000	-10.22156106
-1.21473924	-1.48163476	0.00000000	28.91080302
-1.10890384	-1.48163476	0.00000000	32.07717798
-1.16182154	-1.53455246	0.00000000	13.59181767
-1.16182154	-1.42871706	0.00000000	16.97582422
-2.43005336	-0.81881509	0.00000000	-4.62306498
-2.32421796	-0.81881509	0.00000000	-6.14479169
-2.37713566	-0.87173279	0.00000000	-1.88855628
-2.37713566	-0.76589740	0.00000000	-1.73817666
-2.49323545	0.59060577	0.00000000	-10.85186362
-2.38740004	0.59060577	0.00000000	-14.34908408
-2.44031774	0.53768808	0.00000000	5.64736697
-2.44031774	0.64352347	0.00000000	5.48719623
-1.33902835	1.35334636	0.00000000	30.47097432
-1.23319294	1.35334636	0.00000000	33.40446340
-1.28611065	1.30042866	0.00000000	54.87920693
-1.28611065	1.40626406	0.00000000	45.25052874

45 2657.0 # 737.7 nm, (r3v2r2v4) 45

0.04611 0.087858 0.00000000 1.0 # electr. mom.

0.00000000 0.00000000 -1.664952771 # magnet. mom.

1.31876649	-1.08237463	0.00000000	-138.17684324
0.00000000	-0.70208822	0.00000000	256.11316598
2.11091906	0.04406596	0.00000000	40.78782969
1.32524096	1.15754064	0.00000000	-155.36373791
-0.04178899	0.70146698	0.00000000	271.91546873
-1.16182154	-1.48163476	0.00000000	-84.59603796
-2.37713566	-0.81881509	0.00000000	13.25704879
-2.44031774	0.59060577	0.00000000	12.82196548
-1.28611065	1.35334636	0.00000000	-151.56014061
1.26584879	-1.08237463	0.00000000	35.41866132
1.37168419	-1.08237463	0.00000000	30.41109851
1.31876649	-1.13529233	0.00000000	33.01420818
1.31876649	-1.02945693	0.00000000	40.25290090
-0.05291770	-0.70208822	0.00000000	-62.29263246
0.05291770	-0.70208822	0.00000000	-64.86881983
0.00000000	-0.75500592	0.00000000	-65.47548152
0.00000000	-0.64917052	0.00000000	-64.84849398
2.05800137	0.04406596	0.00000000	-12.05588556
2.16383676	0.04406596	0.00000000	-6.09272836
2.11091906	-0.00885174	0.00000000	-10.67944177
2.11091906	0.09698366	0.00000000	-12.69295842
1.27232327	1.15754064	0.00000000	46.08279268
1.37815866	1.15754064	0.00000000	40.81661614
1.32524096	1.10462294	0.00000000	39.31007007
1.32524096	1.21045834	0.00000000	30.38907849
-0.09470669	0.70146698	0.00000000	-130.22563144
0.01112871	0.70146698	0.00000000	-132.07908983
-0.04178899	0.64854929	0.00000000	-1.24171611
-0.04178899	0.75438469	0.00000000	-9.50425853
-1.21473924	-1.48163476	0.00000000	26.88197474
-1.10890384	-1.48163476	0.00000000	29.82614794
-1.16182154	-1.53455246	0.00000000	12.63800590
-1.16182154	-1.42871706	0.00000000	15.78453831
-2.43005336	-0.81881509	0.00000000	-4.29863937
-2.32421796	-0.81881509	0.00000000	-5.71357824
-2.37713566	-0.87173279	0.00000000	-1.75602602
-2.37713566	-0.76589740	0.00000000	-1.61619935
-2.49323545	0.59060577	0.00000000	-10.09032933
-2.38740004	0.59060577	0.00000000	-13.34213082
-2.44031774	0.53768808	0.00000000	5.25106052
-2.44031774	0.64352347	0.00000000	5.10212983

```
    -1.33902835      1.35334636      0.00000000      28.33266034
    -1.23319294      1.35334636      0.00000000      31.06029053
    -1.28611065      1.30042866      0.00000000      51.02803452
    -1.28611065      1.40626406      0.00000000      42.07505304
45  2657.0  # 737.7 nm,  (r3v3r2v4) 45
     0.02784         0.053046        0.00000000 1.0    # electr. mom.
     0.00000000      0.00000000     -1.664952771       # magnet. mom.
     1.31876649     -1.08237463      0.00000000     -83.42752800
     0.00000000     -0.70208822      0.00000000     154.63436436
     2.11091906      0.04406596      0.00000000      24.62661415
     1.32524096      1.15754064      0.00000000     -93.80452100
    -0.04178899      0.70146698      0.00000000     164.17537734
    -1.16182154     -1.48163476      0.00000000     -51.07685311
    -2.37713566     -0.81881509      0.00000000       8.00425587
    -2.44031774      0.59060577      0.00000000       7.74156407
    -1.28611065      1.35334636      0.00000000     -91.50800942
     1.26584879     -1.08237463      0.00000000      21.38485212
     1.37168419     -1.08237463      0.00000000      18.36141797
     1.31876649     -1.13529233      0.00000000      19.93310683
     1.31876649     -1.02945693      0.00000000      24.30363828
    -0.05291770     -0.70208822      0.00000000     -37.61064601
     0.05291770     -0.70208822      0.00000000     -39.16607990
     0.00000000     -0.75500592      0.00000000     -39.53236620
     0.00000000     -0.64917052      0.00000000     -39.15380768
     2.05800137      0.04406596      0.00000000      -7.27902524
     2.16383676      0.04406596      0.00000000      -3.67862844
     2.11091906     -0.00885174      0.00000000      -6.44796484
     2.11091906      0.09698366      0.00000000      -7.66367301
     1.27232327      1.15754064      0.00000000      27.82357294
     1.37815866      1.15754064      0.00000000      24.64399465
     1.32524096      1.10462294      0.00000000      23.73438193
     1.32524096      1.21045834      0.00000000      18.34812286
    -0.09470669      0.70146698      0.00000000     -78.62679634
     0.01112871      0.70146698      0.00000000     -79.74586556
    -0.04178899      0.64854929      0.00000000      -0.74971539
    -0.04178899      0.75438469      0.00000000      -5.73842025
    -1.21473924     -1.48163476      0.00000000      16.23062626
    -1.10890384     -1.48163476      0.00000000      18.00824027
    -1.16182154     -1.53455246      0.00000000       7.63049413
    -1.16182154     -1.42871706      0.00000000       9.53028728
    -2.43005336     -0.81881509      0.00000000      -2.59540490
    -2.32421796     -0.81881509      0.00000000      -3.44970762
    -2.37713566     -0.87173279      0.00000000      -1.06024212
    -2.37713566     -0.76589740      0.00000000      -0.97581848
    -2.49323545      0.59060577      0.00000000      -6.09227431
    -2.38740004      0.59060577      0.00000000      -8.05562615
    -2.44031774      0.53768808      0.00000000       3.17045163
    -2.44031774      0.64352347      0.00000000       3.08053122
    -1.33902835      1.35334636      0.00000000      17.10651190
    -1.23319294      1.35334636      0.00000000      18.75338296
    -1.28611065      1.30042866      0.00000000      30.80937933
    -1.28611065      1.40626406      0.00000000      25.40380561
45  2657.0  # 737.7 nm,  (r3v4r2v4) 45
     0.02958         0.056362        0.00000000 1.0    # electr. mom.
     0.00000000      0.00000000     -1.664952771       # magnet. mom.
     1.31876649     -1.08237463      0.00000000     -88.64174850
     0.00000000     -0.70208822      0.00000000     164.29901214
     2.11091906      0.04406596      0.00000000      26.16577753
     1.32524096      1.15754064      0.00000000     -99.66730356
    -0.04178899      0.70146698      0.00000000     174.43633843
    -1.16182154     -1.48163476      0.00000000     -54.26915643
    -2.37713566     -0.81881509      0.00000000       8.50452186
    -2.44031774      0.59060577      0.00000000       8.22541182
    -1.28611065      1.35334636      0.00000000     -97.22726001
     1.26584879     -1.08237463      0.00000000      22.72140537
     1.37168419     -1.08237463      0.00000000      19.50900659
     1.31876649     -1.13529233      0.00000000      21.17892600
     1.31876649     -1.02945693      0.00000000      25.82261567
    -0.05291770     -0.70208822      0.00000000     -39.96131139
     0.05291770     -0.70208822      0.00000000     -41.61395989
     0.00000000     -0.75500592      0.00000000     -42.00313909
     0.00000000     -0.64917052      0.00000000     -41.60092066
     2.05800137      0.04406596      0.00000000      -7.73396432
     2.16383676      0.04406596      0.00000000      -3.90854272
     2.11091906     -0.00885174      0.00000000      -6.85096264
     2.11091906      0.09698366      0.00000000      -8.14265257
     1.27232327      1.15754064      0.00000000      29.56254625
     1.37815866      1.15754064      0.00000000      26.18424432
     1.32524096      1.10462294      0.00000000      25.21778080
     1.32524096      1.21045834      0.00000000      19.49488054
    -0.09470669      0.70146698      0.00000000     -83.54097111
     0.01112871      0.70146698      0.00000000     -84.72998216
    -0.04178899      0.64854929      0.00000000      -0.79657260
    -0.04178899      0.75438469      0.00000000      -6.09707151
    -1.21473924     -1.48163476      0.00000000      17.24504040
    -1.10890384     -1.48163476      0.00000000      19.13375528
    -1.16182154     -1.53455246      0.00000000       8.10740001
```

-1.16182154 -1.42871706 0.00000000 10.12593024
-2.43005336 -0.81881509 0.00000000 -2.75761771
-2.32421796 -0.81881509 0.00000000 -3.66531434
-2.37713566 -0.87173279 0.00000000 -1.12650726
-2.37713566 -0.76589740 0.00000000 -1.03680713
-2.49323545 0.59060577 0.00000000 -6.47304146
-2.38740004 0.59060577 0.00000000 -8.55910279
-2.44031774 0.53768808 0.00000000 3.36860486
-2.44031774 0.64352347 0.00000000 3.27306442
-1.33902835 1.35334636 0.00000000 18.17566890
-1.23319294 1.35334636 0.00000000 19.92546940
-1.28611065 1.30042866 0.00000000 32.73496554
-1.28611065 1.40626406 0.00000000 26.99154346
45 2657.0 # 737.7 nm, (r3v5r2v4) 45
0.0261 0.049731 0.00000000 1.0 # electr. mom.
0.00000000 0.00000000 -1.664952771 # magnet. mom.
1.31876649 -1.08237463 0.00000000 -78.21330750
0.00000000 -0.70208822 0.00000000 144.96971659
2.11091906 0.04406596 0.00000000 23.08745077
1.32524096 1.15754064 0.00000000 -87.94173844
-0.04178899 0.70146698 0.00000000 153.91441626
-1.16182154 -1.48163476 0.00000000 -47.88454979
-2.37713566 -0.81881509 0.00000000 7.50398988
-2.44031774 0.59060577 0.00000000 7.25771631
-1.28611065 1.35334636 0.00000000 -85.78875884
1.26584879 -1.08237463 0.00000000 20.04829886
1.37168419 -1.08237463 0.00000000 17.21382935
1.31876649 -1.13529233 0.00000000 18.68728765
1.31876649 -1.02945693 0.00000000 22.78466089
-0.05291770 -0.70208822 0.00000000 -35.25998064
0.05291770 -0.70208822 0.00000000 -36.71819991
0.00000000 -0.75500592 0.00000000 -37.06159331
0.00000000 -0.64917052 0.00000000 -36.70669470
2.05800137 0.04406596 0.00000000 -6.82408616
2.16383676 0.04406596 0.00000000 -3.44871417
2.11091906 -0.00885174 0.00000000 -6.04496704
2.11091906 0.09698366 0.00000000 -7.18469344
1.27232327 1.15754064 0.00000000 26.08459963
1.37815866 1.15754064 0.00000000 23.10374499
1.32524096 1.10462294 0.00000000 22.25098306
1.32524096 1.21045834 0.00000000 17.20136518
-0.09470669 0.70146698 0.00000000 -73.71262157
0.01112871 0.70146698 0.00000000 -74.76174896
-0.04178899 0.64854929 0.00000000 -0.70285817
-0.04178899 0.75438469 0.00000000 -5.37976898
-1.21473924 -1.48163476 0.00000000 15.21621211
-1.10890384 -1.48163476 0.00000000 16.88272525
-1.16182154 -1.53455246 0.00000000 7.15358825
-1.16182154 -1.42871706 0.00000000 8.93464433
-2.43005336 -0.81881509 0.00000000 -2.43319209
-2.32421796 -0.81881509 0.00000000 -3.23410089
-2.37713566 -0.87173279 0.00000000 -0.99397699
-2.37713566 -0.76589740 0.00000000 -0.91482982
-2.49323545 0.59060577 0.00000000 -5.71150717
-2.38740004 0.59060577 0.00000000 -7.55214952
-2.44031774 0.53768808 0.00000000 2.97229841
-2.44031774 0.64352347 0.00000000 2.88799802
-1.33902835 1.35334636 0.00000000 16.03735491
-1.23319294 1.35334636 0.00000000 17.58129653
-1.28611065 1.30042866 0.00000000 28.88379312
-1.28611065 1.40626406 0.00000000 23.81606776
45 2657.0 # 737.7 nm, (r3v6r2v4) 45
0.01566 0.029839 0.00000000 1.0 # electr. mom.
0.00000000 0.00000000 -1.664952771 # magnet. mom.
1.31876649 -1.08237463 0.00000000 -46.92798450
0.00000000 -0.70208822 0.00000000 86.98182995
2.11091906 0.04406596 0.00000000 13.85247046
1.32524096 1.15754064 0.00000000 -52.76504306
-0.04178899 0.70146698 0.00000000 92.34864976
-1.16182154 -1.48163476 0.00000000 -28.73072987
-2.37713566 -0.81881509 0.00000000 4.50239393
-2.44031774 0.59060577 0.00000000 4.35462979
-1.28611065 1.35334636 0.00000000 -51.47325530
1.26584879 -1.08237463 0.00000000 12.02897932
1.37168419 -1.08237463 0.00000000 10.32829761
1.31876649 -1.13529233 0.00000000 11.21237259
1.31876649 -1.02945693 0.00000000 13.67079653
-0.05291770 -0.70208822 0.00000000 -21.15598838
0.05291770 -0.70208822 0.00000000 -22.03091994
0.00000000 -0.75500592 0.00000000 -22.23695599
0.00000000 -0.64917052 0.00000000 -22.02401682
2.05800137 0.04406596 0.00000000 -4.09445170
2.16383676 0.04406596 0.00000000 -2.06922850
2.11091906 -0.00885174 0.00000000 -3.62698022
2.11091906 0.09698366 0.00000000 -4.31081607
1.27232327 1.15754064 0.00000000 15.65075978
1.37815866 1.15754064 0.00000000 13.86224699

```
   1.32524096     1.10462294     0.00000000     13.35058983
   1.32524096     1.21045834     0.00000000     10.32081911
  -0.09470669     0.70146698     0.00000000    -44.22757294
   0.01112871     0.70146698     0.00000000    -44.85704938
  -0.04178899     0.64854929     0.00000000     -0.42171490
  -0.04178899     0.75438469     0.00000000     -3.22786139
  -1.21473924    -1.48163476     0.00000000      9.12972727
  -1.10890384    -1.48163476     0.00000000     10.12963515
  -1.16182154    -1.53455246     0.00000000      4.29215295
  -1.16182154    -1.42871706     0.00000000      5.36078660
  -2.43005336    -0.81881509     0.00000000     -1.45991526
  -2.32421796    -0.81881509     0.00000000     -1.94046053
  -2.37713566    -0.87173279     0.00000000     -0.59638620
  -2.37713566    -0.76589740     0.00000000     -0.54889789
  -2.49323545     0.59060577     0.00000000     -3.42690430
  -2.38740004     0.59060577     0.00000000     -4.53128971
  -2.44031774     0.53768808     0.00000000      1.78337904
  -2.44031774     0.64352347     0.00000000      1.73279881
  -1.33902835     1.35334636     0.00000000      9.62241294
  -1.23319294     1.35334636     0.00000000     10.54877792
  -1.28611065     1.30042866     0.00000000     17.33027587
  -1.28611065     1.40626406     0.00000000     14.28964065
45   2657.0   # 737.7 nm,   (r3v7r2v4) 45
   0.01653        0.031496       0.00000000 1.0    # electr. mom.
   0.00000000     0.00000000    -1.664952771       # magnet. mom.
   1.31876649    -1.08237463     0.00000000    -49.53509475
   0.00000000    -0.70208822     0.00000000     91.81415384
   2.11091906     0.04406596     0.00000000     14.62205215
   1.32524096     1.15754064     0.00000000    -55.69643434
  -0.04178899     0.70146698     0.00000000     97.47913030
  -1.16182154    -1.48163476     0.00000000    -30.32688153
  -2.37713566    -0.81881509     0.00000000      4.75252692
  -2.44031774     0.59060577     0.00000000      4.59655366
  -1.28611065     1.35334636     0.00000000    -54.33288060
   1.26584879    -1.08237463     0.00000000     12.69725594
   1.37168419    -1.08237463     0.00000000     10.90209192
   1.31876649    -1.13529233     0.00000000     11.83528218
   1.31876649    -1.02945693     0.00000000     14.43028523
  -0.05291770    -0.70208822     0.00000000    -22.33132107
   0.05291770    -0.70208822     0.00000000    -23.25485994
   0.00000000    -0.75500592     0.00000000    -23.47234243
   0.00000000    -0.64917052     0.00000000    -23.24757331
   2.05800137     0.04406596     0.00000000     -4.32192124
   2.16383676     0.04406596     0.00000000     -2.18418564
   2.11091906    -0.00885174     0.00000000     -3.82847912
   2.11091906     0.09698366     0.00000000     -4.55030585
   1.27232327     1.15754064     0.00000000     16.52024643
   1.37815866     1.15754064     0.00000000     14.63237183
   1.32524096     1.10462294     0.00000000     14.09228927
   1.32524096     1.21045834     0.00000000     10.89419795
  -0.09470669     0.70146698     0.00000000    -46.68466033
   0.01112871     0.70146698     0.00000000    -47.34910768
  -0.04178899     0.64854929     0.00000000     -0.44514351
  -0.04178899     0.75438469     0.00000000     -3.40718702
  -1.21473924    -1.48163476     0.00000000      9.63693434
  -1.10890384    -1.48163476     0.00000000     10.69239266
  -1.16182154    -1.53455246     0.00000000      4.53060589
  -1.16182154    -1.42871706     0.00000000      5.65860807
  -2.43005336    -0.81881509     0.00000000     -1.54102166
  -2.32421796    -0.81881509     0.00000000     -2.04826390
  -2.37713566    -0.87173279     0.00000000     -0.62951876
  -2.37713566    -0.76589740     0.00000000     -0.57939222
  -2.49323545     0.59060577     0.00000000     -3.61728787
  -2.38740004     0.59060577     0.00000000     -4.78302803
  -2.44031774     0.53768808     0.00000000      1.88245566
  -2.44031774     0.64352347     0.00000000      1.82906541
  -1.33902835     1.35334636     0.00000000     10.15699144
  -1.23319294     1.35334636     0.00000000     11.13482113
  -1.28611065     1.30042866     0.00000000     18.29306898
  -1.28611065     1.40626406     0.00000000     15.08350958
45   2657.0   # 737.7 nm,   (r3v8r2v4) 45
   0.00696        0.013262       0.00000000 1.0    # electr. mom.
   0.00000000     0.00000000    -1.664952771       # magnet. mom.
   1.31876649    -1.08237463     0.00000000    -20.85688200
   0.00000000    -0.70208822     0.00000000     38.65859109
   2.11091906     0.04406596     0.00000000      6.15665354
   1.32524096     1.15754064     0.00000000    -23.45113025
  -0.04178899     0.70146698     0.00000000     41.04384434
  -1.16182154    -1.48163476     0.00000000    -12.76921328
  -2.37713566    -0.81881509     0.00000000      2.00106397
  -2.44031774     0.59060577     0.00000000      1.93539102
  -1.28611065     1.35334636     0.00000000    -22.87700236
   1.26584879    -1.08237463     0.00000000      5.34621303
   1.37168419    -1.08237463     0.00000000      4.59035449
   1.31876649    -1.13529233     0.00000000      4.98327671
   1.31876649    -1.02945693     0.00000000      6.07590957
  -0.05291770    -0.70208822     0.00000000     -9.40266150
```

```
      0.05291770        -0.70208822        0.00000000        -9.79151997
      0.00000000        -0.75500592        0.00000000        -9.88309155
      0.00000000        -0.64917052        0.00000000        -9.78845192
      2.05800137         0.04406596        0.00000000        -1.81975631
      2.16383676         0.04406596        0.00000000        -0.91965711
      2.11091906        -0.00885174        0.00000000        -1.61199121
      2.11091906         0.09698366        0.00000000        -1.91591825
      1.27232327         1.15754064        0.00000000         6.95589323
      1.37815866         1.15754064        0.00000000         6.16099866
      1.32524096         1.10462294        0.00000000         5.93359548
      1.32524096         1.21045834        0.00000000         4.58703072
     -0.09470669         0.70146698        0.00000000       -19.65669908
      0.01112871         0.70146698        0.00000000       -19.93646639
     -0.04178899         0.64854929        0.00000000        -0.18742885
     -0.04178899         0.75438469        0.00000000        -1.43460506
     -1.21473924        -1.48163476        0.00000000         4.05765656
     -1.10890384        -1.48163476        0.00000000         4.50206007
     -1.16182154        -1.53455246        0.00000000         1.90762353
     -1.16182154        -1.42871706        0.00000000         2.38257182
     -2.43005336        -0.81881509        0.00000000        -0.64885123
     -2.32421796        -0.81881509        0.00000000        -0.86242690
     -2.37713566        -0.87173279        0.00000000        -0.26506053
     -2.37713566        -0.76589740        0.00000000        -0.24395462
     -2.49323545         0.59060577        0.00000000        -1.52306858
     -2.38740004         0.59060577        0.00000000        -2.01390654
     -2.44031774         0.53768808        0.00000000         0.79261291
     -2.44031774         0.64352347        0.00000000         0.77013280
     -1.33902835         1.35334636        0.00000000         4.27662798
     -1.23319294         1.35334636        0.00000000         4.68834574
     -1.28611065         1.30042866        0.00000000         7.70234483
     -1.28611065         1.40626406        0.00000000         6.35095140
45    2657.0    # 737.7 nm,    (r3v9r2v4) 45
      0.0087             0.016577          0.00000000 1.0      # electr. mom.
      0.00000000         0.00000000       -1.664952771         # magnet. mom.
      1.31876649        -1.08237463        0.00000000       -26.07110250
      0.00000000        -0.70208822        0.00000000        48.32323886
      2.11091906         0.04406596        0.00000000         7.69581692
      1.32524096         1.15754064        0.00000000       -29.31391281
     -0.04178899         0.70146698        0.00000000        51.30480542
     -1.16182154        -1.48163476        0.00000000       -15.96151660
     -2.37713566        -0.81881509        0.00000000         2.50132996
     -2.44031774         0.59060577        0.00000000         2.41923877
     -1.28611065         1.35334636        0.00000000       -28.59625295
      1.26584879        -1.08237463        0.00000000         6.68276629
      1.37168419        -1.08237463        0.00000000         5.73794312
      1.31876649        -1.13529233        0.00000000         6.22909588
      1.31876649        -1.02945693        0.00000000         7.59488696
     -0.05291770        -0.70208822        0.00000000       -11.75332688
      0.05291770        -0.70208822        0.00000000       -12.23939997
      0.00000000        -0.75500592        0.00000000       -12.35386444
      0.00000000        -0.64917052        0.00000000       -12.23556490
      2.05800137         0.04406596        0.00000000        -2.27469539
      2.16383676         0.04406596        0.00000000        -1.14957139
      2.11091906        -0.00885174        0.00000000        -2.01498901
      2.11091906         0.09698366        0.00000000        -2.39489781
      1.27232327         1.15754064        0.00000000         8.69486654
      1.37815866         1.15754064        0.00000000         7.70124833
      1.32524096         1.10462294        0.00000000         7.41699435
      1.32524096         1.21045834        0.00000000         5.73378839
     -0.09470669         0.70146698        0.00000000       -24.57087386
      0.01112871         0.70146698        0.00000000       -24.92058299
     -0.04178899         0.64854929        0.00000000        -0.23428606
     -0.04178899         0.75438469        0.00000000        -1.79325633
     -1.21473924        -1.48163476        0.00000000         5.07207070
     -1.10890384        -1.48163476        0.00000000         5.62757508
     -1.16182154        -1.53455246        0.00000000         2.38452942
     -1.16182154        -1.42871706        0.00000000         2.97821478
     -2.43005336        -0.81881509        0.00000000        -0.81106403
     -2.32421796        -0.81881509        0.00000000        -1.07803363
     -2.37713566        -0.87173279        0.00000000        -0.33132566
     -2.37713566        -0.76589740        0.00000000        -0.30494327
     -2.49323545         0.59060577        0.00000000        -1.90383572
     -2.38740004         0.59060577        0.00000000        -2.51738317
     -2.44031774         0.53768808        0.00000000         0.99076614
     -2.44031774         0.64352347        0.00000000         0.96266601
     -1.33902835         1.35334636        0.00000000         5.34578497
     -1.23319294         1.35334636        0.00000000         5.86043218
     -1.28611065         1.30042866        0.00000000         9.62793104
     -1.28611065         1.40626406        0.00000000         7.93868925
45    13555.0    # 737.7 nm,    (r7r2v4) 45
      0.12007           -0.02816           0.00000000 1.0      # electr. mom.
      0.00000000         0.00000000       -0.8485163126        # magnet. mom.
      1.31876649        -1.08237463        0.00000000         8.12951557
      0.00000000        -0.70208822        0.00000000         3.93443449
      2.11091906         0.04406596        0.00000000         3.48279790
      1.32524096         1.15754064        0.00000000        -5.03624300
     -0.04178899         0.70146698        0.00000000        44.23286927
```

-1.16182154	-1.48163476	0.00000000	-46.47021425
-2.37713566	-0.81881509	0.00000000	26.34821224
-2.44031774	0.59060577	0.00000000	-11.45850734
-1.28611065	1.35334636	0.00000000	-20.64369404
1.26584879	-1.08237463	0.00000000	-2.49952895
1.37168419	-1.08237463	0.00000000	-1.78055941
1.31876649	-1.13529233	0.00000000	-1.51727249
1.31876649	-1.02945693	0.00000000	-2.47073541
-0.05291770	-0.70208822	0.00000000	17.18567605
0.05291770	-0.70208822	0.00000000	17.66538897
0.00000000	-0.75500592	0.00000000	-18.06834765
0.00000000	-0.64917052	0.00000000	-20.68137490
2.05800137	0.04406596	0.00000000	-2.36751651
2.16383676	0.04406596	0.00000000	-2.28741725
2.11091906	-0.00885174	0.00000000	0.29290969
2.11091906	0.09698366	0.00000000	0.94209985
1.27232327	1.15754064	0.00000000	-6.25709755
1.37815866	1.15754064	0.00000000	-5.41939555
1.32524096	1.10462294	0.00000000	8.99309825
1.32524096	1.21045834	0.00000000	7.69858754
-0.09470669	0.70146698	0.00000000	-22.64448100
0.01112871	0.70146698	0.00000000	-23.84463155
-0.04178899	0.64854929	0.00000000	0.86718097
-0.04178899	0.75438469	0.00000000	1.61278312
-1.21473924	-1.48163476	0.00000000	16.34380704
-1.10890384	-1.48163476	0.00000000	16.94543521
-1.16182154	-1.53455246	0.00000000	6.56426260
-1.16182154	-1.42871706	0.00000000	6.51327335
-2.43005336	-0.81881509	0.00000000	-6.85306816
-2.32421796	-0.81881509	0.00000000	-7.27648944
-2.37713566	-0.87173279	0.00000000	-6.07458327
-2.37713566	-0.76589740	0.00000000	-6.03046633
-2.49323545	0.59060577	0.00000000	5.89172357
-2.38740004	0.59060577	0.00000000	6.96706858
-2.44031774	0.53768808	0.00000000	-1.38794712
-2.44031774	0.64352347	0.00000000	-0.03723483
-1.33902835	1.35334636	0.00000000	2.30086076
-1.23319294	1.35334636	0.00000000	2.13466393
-1.28611065	1.30042866	0.00000000	8.23100362
-1.28611065	1.40626406	0.00000000	7.82915338

45 15636.0 # 639.5 nm, (r8r2v4) 45

-0.3156	0.045266	0.00000000 1.0	# electr. mom.
0.00000000	0.00000000	-0.9999114666E-01	# magnet. mom.
1.31876649	-1.08237463	0.00000000	-17.23416499
0.00000000	-0.70208822	0.00000000	57.05475024
2.11091906	0.04406596	0.00000000	7.87891130
1.32524096	1.15754064	0.00000000	63.01611207
-0.04178899	0.70146698	0.00000000	-105.03180957
-1.16182154	-1.48163476	0.00000000	-11.37016997
-2.37713566	-0.81881509	0.00000000	19.72383138
-2.44031774	0.59060577	0.00000000	-18.87623178
-1.28611065	1.35334636	0.00000000	46.48322363
1.26584879	-1.08237463	0.00000000	2.08670514
1.37168419	-1.08237463	0.00000000	2.10778366
1.31876649	-1.13529233	0.00000000	6.40790498
1.31876649	-1.02945693	0.00000000	6.72455788
-0.05291770	-0.70208822	0.00000000	-13.46461583
0.05291770	-0.70208822	0.00000000	-11.96894134
0.00000000	-0.75500592	0.00000000	-16.14204054
0.00000000	-0.64917052	0.00000000	-15.63738708
2.05800137	0.04406596	0.00000000	2.68553460
2.16383676	0.04406596	0.00000000	2.55756193
2.11091906	-0.00885174	0.00000000	-8.38590942
2.11091906	0.09698366	0.00000000	-4.78337819
1.27232327	1.15754064	0.00000000	-17.20921625
1.37815866	1.15754064	0.00000000	-16.56359554
1.32524096	1.10462294	0.00000000	-15.42579048
1.32524096	1.21045834	0.00000000	-13.93978798
-0.09470669	0.70146698	0.00000000	42.09766448
0.01112871	0.70146698	0.00000000	39.33260280
-0.04178899	0.64854929	0.00000000	11.07825128
-0.04178899	0.75438469	0.00000000	12.71984902
-1.21473924	-1.48163476	0.00000000	0.44137983
-1.10890384	-1.48163476	0.00000000	1.21749704
-1.16182154	-1.53455246	0.00000000	3.86548055
-1.16182154	-1.42871706	0.00000000	6.11330938
-2.43005336	-0.81881509	0.00000000	-12.06409453
-2.32421796	-0.81881509	0.00000000	-14.09091482
-2.37713566	-0.87173279	0.00000000	2.92960856
-2.37713566	-0.76589740	0.00000000	3.36987412
-2.49323545	0.59060577	0.00000000	2.07567710
-2.38740004	0.59060577	0.00000000	4.14226140
-2.44031774	0.53768808	0.00000000	6.85126869
-2.44031774	0.64352347	0.00000000	6.06077136
-1.33902835	1.35334636	0.00000000	-12.32004391
-1.23319294	1.35334636	0.00000000	-12.44841135
-1.28611065	1.30042866	0.00000000	-12.48887347

-1.28611065 1.40626406 0.00000000 -9.57699542
&TRANSITION 6->...
0 0.0 # (r2v6r2v5) 45
0.00000000 0.00000000 0.00000000 1.0 # electr. mom.
0.00000000 0.00000000 0.00000000 # magnet. mom.
0 0.0 # (r2v7vr2v5) 45
0.00000000 0.00000000 0.00000000 1.0 # electr. mom.
0.00000000 0.00000000 0.00000000 # magnet. mom.
45 2657.0 # 737.7 nm, (r3v1r2v5) 45
0.071151 0.135571 0.00000000 1.0 # electr. mom.
0.00000000 0.00000000 -1.664952771 # magnet. mom.
1.31876649 -1.08237463 0.00000000 -213.21627739
0.00000000 -0.70208822 0.00000000 395.20005349
2.11091906 0.04406596 0.00000000 62.93839839
1.32524096 1.15754064 0.00000000 -239.73682609
-0.04178899 0.70146698 0.00000000 419.58408259
-1.16182154 -1.48163476 0.00000000 -130.53744659
-2.37713566 -0.81881509 0.00000000 20.45652893
-2.44031774 0.59060577 0.00000000 19.78516577
-1.28611065 1.35334636 0.00000000 -233.86761648
1.26584879 -1.08237463 0.00000000 54.65340602
1.37168419 -1.08237463 0.00000000 46.92639565
1.31876649 -1.13529233 0.00000000 50.94317112
1.31876649 -1.02945693 0.00000000 62.11296686
-0.05291770 -0.70208822 0.00000000 -96.12177331
0.05291770 -0.70208822 0.00000000 -100.09700583
0.00000000 -0.75500592 0.00000000 -101.03312612
0.00000000 -0.64917052 0.00000000 -100.06564165
2.05800137 0.04406596 0.00000000 -18.60305228
2.16383676 0.04406596 0.00000000 -9.40149471
2.11091906 -0.00885174 0.00000000 -16.47910579
2.11091906 0.09698366 0.00000000 -19.58609909
1.27232327 1.15754064 0.00000000 71.10888681
1.37815866 1.15754064 0.00000000 62.98281786
1.32524096 1.10462294 0.00000000 60.65811468
1.32524096 1.21045834 0.00000000 46.89241726
-0.09470669 0.70146698 0.00000000 -200.94701619
0.01112871 0.70146698 0.00000000 -203.80702869
-0.04178899 0.64854929 0.00000000 -1.91605250
-0.04178899 0.75438469 0.00000000 -14.66571805
-1.21473924 -1.48163476 0.00000000 41.48071737
-1.10890384 -1.48163476 0.00000000 46.02377710
-1.16182154 -1.53455246 0.00000000 19.50130361
-1.16182154 -1.42871706 0.00000000 24.35661736
-2.43005336 -0.81881509 0.00000000 -6.63309323
-2.32421796 -0.81881509 0.00000000 -8.81644025
-2.37713566 -0.87173279 0.00000000 -2.70966771
-2.37713566 -0.76589740 0.00000000 -2.49390565
-2.49323545 0.59060577 0.00000000 -15.57006519
-2.38740004 0.59060577 0.00000000 -20.58781629
-2.44031774 0.53768808 0.00000000 8.10274392
-2.44031774 0.64352347 0.00000000 7.87293372
-1.33902835 1.35334636 0.00000000 43.71922403
-1.23319294 1.35334636 0.00000000 47.92814314
-1.28611065 1.30042866 0.00000000 78.73973169
-1.28611065 1.40626406 0.00000000 64.92467167
45 2657.0 # 737.7 nm, (r3v2r2v5) 45
0.066158 0.126057 0.00000000 1.0 # electr. mom.
0.00000000 0.00000000 -1.664952771 # magnet. mom.
1.31876649 -1.08237463 0.00000000 -198.25373161
0.00000000 -0.70208822 0.00000000 367.46671640
2.11091906 0.04406596 0.00000000 58.52166868
1.32524096 1.15754064 0.00000000 -222.91318917
-0.04178899 0.70146698 0.00000000 390.13958556
-1.16182154 -1.48163476 0.00000000 -121.37692403
-2.37713566 -0.81881509 0.00000000 19.02098304
-2.44031774 0.59060577 0.00000000 18.39673309
-1.28611065 1.35334636 0.00000000 -217.45585392
1.26584879 -1.08237463 0.00000000 50.81807928
1.37168419 -1.08237463 0.00000000 43.63331525
1.31876649 -1.13529233 0.00000000 47.36821174
1.31876649 -1.02945693 0.00000000 57.75416217
-0.05291770 -0.70208822 0.00000000 -89.37638571
0.05291770 -0.70208822 0.00000000 -93.07265454
0.00000000 -0.75500592 0.00000000 -93.94308218
0.00000000 -0.64917052 0.00000000 -93.04349136
2.05800137 0.04406596 0.00000000 -17.29757493
2.16383676 0.04406596 0.00000000 -8.74174069
2.11091906 -0.00885174 0.00000000 -15.32267732
2.11091906 0.09698366 0.00000000 -18.21163599
1.27232327 1.15754064 0.00000000 66.11878949
1.37815866 1.15754064 0.00000000 58.56297099
1.32524096 1.10462294 0.00000000 56.40140488
1.32524096 1.21045834 0.00000000 43.60172131
-0.09470669 0.70146698 0.00000000 -186.84547119
0.01112871 0.70146698 0.00000000 -189.50478106
-0.04178899 0.64854929 0.00000000 -1.78159267

```
     -0.04178899     0.75438469     0.00000000    -13.63654485
     -1.21473924    -1.48163476     0.00000000     38.56978984
     -1.10890384    -1.48163476     0.00000000     42.79403835
     -1.16182154    -1.53455246     0.00000000     18.13279108
     -1.16182154    -1.42871706     0.00000000     22.64738105
     -2.43005336    -0.81881509     0.00000000     -6.16761301
     -2.32421796    -0.81881509     0.00000000     -8.19774269
     -2.37713566    -0.87173279     0.00000000     -2.51951559
     -2.37713566    -0.76589740     0.00000000     -2.31889472
     -2.49323545     0.59060577     0.00000000    -14.47742904
     -2.38740004     0.59060577     0.00000000    -19.14305726
     -2.44031774     0.53768808     0.00000000      7.53413031
     -2.44031774     0.64352347     0.00000000      7.32044714
     -1.33902835     1.35334636     0.00000000     40.65120831
     -1.23319294     1.35334636     0.00000000     44.56476467
     -1.28611065     1.30042866     0.00000000     73.21413648
     -1.28611065     1.40626406     0.00000000     60.36855436
45    2657.0    # 737.7 nm,    (r3v3r2v5) 45
      0.039945       0.07611        0.00000000 1.0     # electr. mom.
      0.00000000     0.00000000    -1.664952771         # magnet. mom.
      1.31876649    -1.08237463     0.00000000   -119.70036626
      0.00000000    -0.70208822     0.00000000    221.86669669
      2.11091906     0.04406596     0.00000000     35.33383769
      1.32524096     1.15754064     0.00000000   -134.58909535
     -0.04178899     0.70146698     0.00000000    235.55597619
     -1.16182154    -1.48163476     0.00000000    -73.28418054
     -2.37713566    -0.81881509     0.00000000     11.48436712
     -2.44031774     0.59060577     0.00000000     11.10746149
     -1.28611065     1.35334636     0.00000000   -131.29410048
      1.26584879    -1.08237463     0.00000000     30.68261391
      1.37168419    -1.08237463     0.00000000     26.34464317
      1.31876649    -1.13529233     0.00000000     28.59967501
      1.31876649    -1.02945693     0.00000000     34.87043754
     -0.05291770    -0.70208822     0.00000000    -53.96310080
      0.05291770    -0.70208822     0.00000000    -56.19481029
      0.00000000    -0.75500592     0.00000000    -56.72035150
      0.00000000    -0.64917052     0.00000000    -56.17720233
      2.05800137     0.04406596     0.00000000    -10.44381882
      2.16383676     0.04406596     0.00000000     -5.27803212
      2.11091906    -0.00885174     0.00000000     -9.25142781
      2.11091906     0.09698366     0.00000000    -10.99570475
      1.27232327     1.15754064     0.00000000     39.92077856
      1.37815866     1.15754064     0.00000000     35.35877494
      1.32524096     1.10462294     0.00000000     34.05367842
      1.32524096     1.21045834     0.00000000     26.32556758
     -0.09470669     0.70146698     0.00000000   -112.81235997
      0.01112871     0.70146698     0.00000000   -114.41798102
     -0.04178899     0.64854929     0.00000000     -1.07567860
     -0.04178899     0.75438469     0.00000000     -8.23338557
     -1.21473924    -1.48163476     0.00000000     23.28742028
     -1.10890384    -1.48163476     0.00000000     25.83790995
     -1.16182154    -1.53455246     0.00000000     10.94810027
     -1.16182154    -1.42871706     0.00000000     13.67389045
     -2.43005336    -0.81881509     0.00000000     -3.72384181
     -2.32421796    -0.81881509     0.00000000     -4.94958049
     -2.37713566    -0.87173279     0.00000000     -1.52121696
     -2.37713566    -0.76589740     0.00000000     -1.40008738
     -2.49323545     0.59060577     0.00000000     -8.74108923
     -2.38740004     0.59060577     0.00000000    -11.55807231
     -2.44031774     0.53768808     0.00000000      4.54890887
     -2.44031774     0.64352347     0.00000000      4.41989261
     -1.33902835     1.35334636     0.00000000     24.54412577
     -1.23319294     1.35334636     0.00000000     26.90702773
     -1.28611065     1.30042866     0.00000000     44.20476165
     -1.28611065     1.40626406     0.00000000     36.44893848
45    2657.0    # 737.7 nm,    (r3v4r2v5) 45
      0.042441       0.080867       0.00000000 1.0     # electr. mom.
      0.00000000     0.00000000    -1.664952771         # magnet. mom.
      1.31876649    -1.08237463     0.00000000   -127.18163915
      0.00000000    -0.70208822     0.00000000    235.73336524
      2.11091906     0.04406596     0.00000000     37.54220255
      1.32524096     1.15754064     0.00000000   -143.00091381
     -0.04178899     0.70146698     0.00000000    250.27822470
     -1.16182154    -1.48163476     0.00000000    -77.86444183
     -2.37713566    -0.81881509     0.00000000     12.20214006
     -2.44031774     0.59060577     0.00000000     11.80167783
     -1.28611065     1.35334636     0.00000000   -139.49998176
      1.26584879    -1.08237463     0.00000000     32.60027728
      1.37168419    -1.08237463     0.00000000     27.99118337
      1.31876649    -1.13529233     0.00000000     30.38715470
      1.31876649    -1.02945693     0.00000000     37.04983988
     -0.05291770    -0.70208822     0.00000000    -57.33579460
      0.05291770    -0.70208822     0.00000000    -59.70698593
      0.00000000    -0.75500592     0.00000000    -60.26537347
      0.00000000    -0.64917052     0.00000000    -59.68827747
      2.05800137     0.04406596     0.00000000    -11.09655750
      2.16383676     0.04406596     0.00000000     -5.60790912
```

```
   2.11091906     -0.00885174     0.00000000     -9.82964205
   2.11091906      0.09698366     0.00000000    -11.68293630
   1.27232327      1.15754064     0.00000000     42.41582722
   1.37815866      1.15754064     0.00000000     37.56869837
   1.32524096      1.10462294     0.00000000     36.18203332
   1.32524096      1.21045834     0.00000000     27.97091556
  -0.09470669      0.70146698     0.00000000   -119.86313246
   0.01112871      0.70146698     0.00000000   -121.56910483
  -0.04178899      0.64854929     0.00000000     -1.14290851
  -0.04178899      0.75438469     0.00000000     -8.74797217
  -1.21473924     -1.48163476     0.00000000     24.74288405
  -1.10890384     -1.48163476     0.00000000     27.45277932
  -1.16182154     -1.53455246     0.00000000     11.63235654
  -1.16182154     -1.42871706     0.00000000     14.52850860
  -2.43005336     -0.81881509     0.00000000     -3.95658193
  -2.32421796     -0.81881509     0.00000000     -5.25892927
  -2.37713566     -0.87173279     0.00000000     -1.61629302
  -2.37713566     -0.76589740     0.00000000     -1.48759284
  -2.49323545      0.59060577     0.00000000     -9.28740731
  -2.38740004      0.59060577     0.00000000    -12.28045182
  -2.44031774      0.53768808     0.00000000      4.83321567
  -2.44031774      0.64352347     0.00000000      4.69613590
  -1.33902835      1.35334636     0.00000000     26.07813363
  -1.23319294      1.35334636     0.00000000     28.58871696
  -1.28611065      1.30042866     0.00000000     46.96755925
  -1.28611065      1.40626406     0.00000000     38.72699713
45    2657.0    # 737.7 nm,    (r3v5r2v5) 45
   0.037448        0.071353       0.00000000 1.0      # electr. mom.
   0.00000000      0.00000000    -1.664952771          # magnet. mom.
   1.31876649     -1.08237463     0.00000000   -112.21909336
   0.00000000     -0.70208822     0.00000000    208.00002815
   2.11091906      0.04406596     0.00000000     33.12547284
   1.32524096      1.15754064     0.00000000   -126.17727689
  -0.04178899      0.70146698     0.00000000    220.83372768
  -1.16182154     -1.48163476     0.00000000    -68.70391926
  -2.37713566     -0.81881509     0.00000000     10.76659417
  -2.44031774      0.59060577     0.00000000     10.41324514
  -1.28611065      1.35334636     0.00000000   -123.08821920
   1.26584879     -1.08237463     0.00000000     28.76495054
   1.37168419     -1.08237463     0.00000000     24.69810297
   1.31876649     -1.13529233     0.00000000     26.81219533
   1.31876649     -1.02945693     0.00000000     32.69103519
  -0.05291770     -0.70208822     0.00000000    -50.59040700
   0.05291770     -0.70208822     0.00000000    -52.68263465
   0.00000000     -0.75500592     0.00000000    -53.17532953
   0.00000000     -0.64917052     0.00000000    -52.66612718
   2.05800137      0.04406596     0.00000000     -9.79108015
   2.16383676      0.04406596     0.00000000     -4.94815511
   2.11091906     -0.00885174     0.00000000     -8.67321358
   2.11091906      0.09698366     0.00000000    -10.30847320
   1.27232327      1.15754064     0.00000000     37.42572990
   1.37815866      1.15754064     0.00000000     33.14885150
   1.32524096      1.10462294     0.00000000     31.92532352
   1.32524096      1.21045834     0.00000000     24.68021961
  -0.09470669      0.70146698     0.00000000   -105.76158747
   0.01112871      0.70146698     0.00000000   -107.26685720
  -0.04178899      0.64854929     0.00000000     -1.00844868
  -0.04178899      0.75438469     0.00000000     -7.71879897
  -1.21473924     -1.48163476     0.00000000     21.83195651
  -1.10890384     -1.48163476     0.00000000     24.22304058
  -1.16182154     -1.53455246     0.00000000     10.26384401
  -1.16182154     -1.42871706     0.00000000     12.81927229
  -2.43005336     -0.81881509     0.00000000     -3.49110170
  -2.32421796     -0.81881509     0.00000000     -4.64023171
  -2.37713566     -0.87173279     0.00000000     -1.42614090
  -2.37713566     -0.76589740     0.00000000     -1.31258192
  -2.49323545      0.59060577     0.00000000     -8.19477115
  -2.38740004      0.59060577     0.00000000    -10.83569279
  -2.44031774      0.53768808     0.00000000      4.26460206
  -2.44031774      0.64352347     0.00000000      4.14364933
  -1.33902835      1.35334636     0.00000000     23.01011791
  -1.23319294      1.35334636     0.00000000     25.22533850
  -1.28611065      1.30042866     0.00000000     41.44196405
  -1.28611065      1.40626406     0.00000000     34.17087982
45    2657.0    # 737.7 nm,    (r3v6r2v5) 45
   0.022469        0.042812       0.00000000 1.0      # electr. mom.
   0.00000000      0.00000000    -1.664952771          # magnet. mom.
   1.31876649     -1.08237463     0.00000000    -67.33145602
   0.00000000     -0.70208822     0.00000000    124.80001689
   2.11091906      0.04406596     0.00000000     19.87528370
   1.32524096      1.15754064     0.00000000    -75.70636613
  -0.04178899      0.70146698     0.00000000    132.50023661
  -1.16182154     -1.48163476     0.00000000    -41.22235156
  -2.37713566     -0.81881509     0.00000000      6.45995650
  -2.44031774      0.59060577     0.00000000      6.24794709
  -1.28611065      1.35334636     0.00000000    -73.85293152
   1.26584879     -1.08237463     0.00000000     17.25897032
```

```
     1.37168419        -1.08237463         0.00000000         14.81886178
     1.31876649        -1.13529233         0.00000000         16.08731720
     1.31876649        -1.02945693         0.00000000         19.61462111
    -0.05291770        -0.70208822         0.00000000        -30.35424420
     0.05291770        -0.70208822         0.00000000        -31.60958079
     0.00000000        -0.75500592         0.00000000        -31.90519772
     0.00000000        -0.64917052         0.00000000        -31.59967631
     2.05800137         0.04406596         0.00000000         -5.87464809
     2.16383676         0.04406596         0.00000000         -2.96889307
     2.11091906        -0.00885174         0.00000000         -5.20392815
     2.11091906         0.09698366         0.00000000         -6.18508392
     1.27232327         1.15754064         0.00000000         22.45543794
     1.37815866         1.15754064         0.00000000         19.88931090
     1.32524096         1.10462294         0.00000000         19.15519411
     1.32524096         1.21045834         0.00000000         14.80813177
    -0.09470669         0.70146698         0.00000000        -63.45695248
     0.01112871         0.70146698         0.00000000        -64.36011432
    -0.04178899         0.64854929         0.00000000         -0.60506921
    -0.04178899         0.75438469         0.00000000         -4.63127938
    -1.21473924        -1.48163476         0.00000000         13.09917391
    -1.10890384        -1.48163476         0.00000000         14.53382435
    -1.16182154        -1.53455246         0.00000000          6.15830640
    -1.16182154        -1.42871706         0.00000000          7.69156338
    -2.43005336        -0.81881509         0.00000000         -2.09466102
    -2.32421796        -0.81881509         0.00000000         -2.78413903
    -2.37713566        -0.87173279         0.00000000         -0.85568454
    -2.37713566        -0.76589740         0.00000000         -0.78754915
    -2.49323545         0.59060577         0.00000000         -4.91686269
    -2.38740004         0.59060577         0.00000000         -6.50141567
    -2.44031774         0.53768808         0.00000000          2.55876124
    -2.44031774         0.64352347         0.00000000          2.48618960
    -1.33902835         1.35334636         0.00000000         13.80607075
    -1.23319294         1.35334636         0.00000000         15.13520310
    -1.28611065         1.30042866         0.00000000         24.86517843
    -1.28611065         1.40626406         0.00000000         20.50252789
45    2657.0    # 737.7 nm,    (r3v7r2v5) 45
     0.023717           0.04519            0.00000000 1.0      # electr. mom.
     0.00000000         0.00000000        -1.664952771          # magnet. mom.
     1.31876649        -1.08237463         0.00000000        -71.07209246
     0.00000000        -0.70208822         0.00000000        131.73335116
     2.11091906         0.04406596         0.00000000         20.97946613
     1.32524096         1.15754064         0.00000000        -79.91227536
    -0.04178899         0.70146698         0.00000000        139.86136086
    -1.16182154        -1.48163476         0.00000000        -43.51248220
    -2.37713566        -0.81881509         0.00000000          6.81884298
    -2.44031774         0.59060577         0.00000000          6.59505526
    -1.28611065         1.35334636         0.00000000        -77.95587216
     1.26584879        -1.08237463         0.00000000         18.21780201
     1.37168419        -1.08237463         0.00000000         15.64213188
     1.31876649        -1.13529233         0.00000000         16.98105704
     1.31876649        -1.02945693         0.00000000         20.70432229
    -0.05291770        -0.70208822         0.00000000        -32.04059110
     0.05291770        -0.70208822         0.00000000        -33.36566861
     0.00000000        -0.75500592         0.00000000        -33.67770871
     0.00000000        -0.64917052         0.00000000        -33.35521388
     2.05800137         0.04406596         0.00000000         -6.20101743
     2.16383676         0.04406596         0.00000000         -3.13383157
     2.11091906        -0.00885174         0.00000000         -5.49303526
     2.11091906         0.09698366         0.00000000         -6.52869970
     1.27232327         1.15754064         0.00000000         23.70296227
     1.37815866         1.15754064         0.00000000         20.99427262
     1.32524096         1.10462294         0.00000000         20.21937156
     1.32524096         1.21045834         0.00000000         15.63080575
    -0.09470669         0.70146698         0.00000000        -66.98233873
     0.01112871         0.70146698         0.00000000        -67.93567623
    -0.04178899         0.64854929         0.00000000         -0.63868417
    -0.04178899         0.75438469         0.00000000         -4.88857268
    -1.21473924        -1.48163476         0.00000000         13.82690579
    -1.10890384        -1.48163476         0.00000000         15.34125903
    -1.16182154        -1.53455246         0.00000000          6.50043454
    -1.16182154        -1.42871706         0.00000000          8.11887245
    -2.43005336        -0.81881509         0.00000000         -2.21103108
    -2.32421796        -0.81881509         0.00000000         -2.93881342
    -2.37713566        -0.87173279         0.00000000         -0.90322257
    -2.37713566        -0.76589740         0.00000000         -0.83130188
    -2.49323545         0.59060577         0.00000000         -5.19002173
    -2.38740004         0.59060577         0.00000000         -6.86260543
    -2.44031774         0.53768808         0.00000000          2.70091464
    -2.44031774         0.64352347         0.00000000          2.62431124
    -1.33902835         1.35334636         0.00000000         14.57307468
    -1.23319294         1.35334636         0.00000000         15.97604771
    -1.28611065         1.30042866         0.00000000         26.24657723
    -1.28611065         1.40626406         0.00000000         21.64155722
45    2657.0    # 737.7 nm,    (r3v8r2v5) 45
     0.009986           0.019027           0.00000000 1.0      # electr. mom.
     0.00000000         0.00000000        -1.664952771          # magnet. mom.
     1.31876649        -1.08237463         0.00000000        -29.92509156
```

```
     0.00000000     -0.70208822      0.00000000      55.46667417
     2.11091906      0.04406596      0.00000000       8.83345942
     1.32524096      1.15754064      0.00000000     -33.64727384
    -0.04178899      0.70146698      0.00000000      58.88899405
    -1.16182154     -1.48163476      0.00000000     -18.32104514
    -2.37713566     -0.81881509      0.00000000       2.87109178
    -2.44031774      0.59060577      0.00000000       2.77686537
    -1.28611065      1.35334636      0.00000000     -32.82352512
     1.26584879     -1.08237463      0.00000000       7.67065348
     1.37168419     -1.08237463      0.00000000       6.58616079
     1.31876649     -1.13529233      0.00000000       7.14991875
     1.31876649     -1.02945693      0.00000000       8.71760938
    -0.05291770     -0.70208822      0.00000000     -13.49077520
     0.05291770     -0.70208822      0.00000000     -14.04870257
     0.00000000     -0.75500592      0.00000000     -14.18008788
     0.00000000     -0.64917052      0.00000000     -14.04430058
     2.05800137      0.04406596      0.00000000      -2.61095471
     2.16383676      0.04406596      0.00000000      -1.31950803
     2.11091906     -0.00885174      0.00000000      -2.31285695
     2.11091906      0.09698366      0.00000000      -2.74892619
     1.27232327      1.15754064      0.00000000       9.98019464
     1.37815866      1.15754064      0.00000000       8.83969373
     1.32524096      1.10462294      0.00000000       8.51341960
     1.32524096      1.21045834      0.00000000       6.58139190
    -0.09470669      0.70146698      0.00000000     -28.20308999
     0.01112871      0.70146698      0.00000000     -28.60449525
    -0.04178899      0.64854929      0.00000000      -0.26891965
    -0.04178899      0.75438469      0.00000000      -2.05834639
    -1.21473924     -1.48163476      0.00000000       5.82185507
    -1.10890384     -1.48163476      0.00000000       6.45947749
    -1.16182154     -1.53455246      0.00000000       2.73702507
    -1.16182154     -1.42871706      0.00000000       3.41847261
    -2.43005336     -0.81881509      0.00000000      -0.93096045
    -2.32421796     -0.81881509      0.00000000      -1.23739512
    -2.37713566     -0.87173279      0.00000000      -0.38030424
    -2.37713566     -0.76589740      0.00000000      -0.35002185
    -2.49323545      0.59060577      0.00000000      -2.18527231
    -2.38740004      0.59060577      0.00000000      -2.88951808
    -2.44031774      0.53768808      0.00000000       1.13722722
    -2.44031774      0.64352347      0.00000000       1.10497315
    -1.33902835      1.35334636      0.00000000       6.13603144
    -1.23319294      1.35334636      0.00000000       6.72675693
    -1.28611065      1.30042866      0.00000000      11.05119041
    -1.28611065      1.40626406      0.00000000       9.11223462
45    2657.0    # 737.7 nm,    (r3v9r2v5) 45
     0.012483        0.023784        0.00000000 1.0      # electr. mom.
     0.00000000      0.00000000     -1.664952771         # magnet. mom.
     1.31876649     -1.08237463      0.00000000     -37.40636445
     0.00000000     -0.70208822      0.00000000      69.33334272
     2.11091906      0.04406596      0.00000000      11.04182428
     1.32524096      1.15754064      0.00000000     -42.05909230
    -0.04178899      0.70146698      0.00000000      73.61124256
    -1.16182154     -1.48163476      0.00000000     -22.90130642
    -2.37713566     -0.81881509      0.00000000       3.58886472
    -2.44031774      0.59060577      0.00000000       3.47108171
    -1.28611065      1.35334636      0.00000000     -41.02940640
     1.26584879     -1.08237463      0.00000000       9.58831685
     1.37168419     -1.08237463      0.00000000       8.23270099
     1.31876649     -1.13529233      0.00000000       8.93739844
     1.31876649     -1.02945693      0.00000000      10.89701173
    -0.05291770     -0.70208822      0.00000000     -16.86346900
     0.05291770     -0.70208822      0.00000000     -17.56087822
     0.00000000     -0.75500592      0.00000000     -17.72510984
     0.00000000     -0.64917052      0.00000000     -17.55537573
     2.05800137      0.04406596      0.00000000      -3.26369338
     2.16383676      0.04406596      0.00000000      -1.64938504
     2.11091906     -0.00885174      0.00000000      -2.89107119
     2.11091906      0.09698366      0.00000000      -3.43615773
     1.27232327      1.15754064      0.00000000      12.47524330
     1.37815866      1.15754064      0.00000000      11.04961717
     1.32524096      1.10462294      0.00000000      10.64177451
     1.32524096      1.21045834      0.00000000       8.22673987
    -0.09470669      0.70146698      0.00000000     -35.25386249
     0.01112871      0.70146698      0.00000000     -35.75561907
    -0.04178899      0.64854929      0.00000000      -0.33614956
    -0.04178899      0.75438469      0.00000000      -2.57293299
    -1.21473924     -1.48163476      0.00000000       7.27731884
    -1.10890384     -1.48163476      0.00000000       8.07434686
    -1.16182154     -1.53455246      0.00000000       3.42128134
    -1.16182154     -1.42871706      0.00000000       4.27309076
    -2.43005336     -0.81881509      0.00000000      -1.16370057
    -2.32421796     -0.81881509      0.00000000      -1.54674390
    -2.37713566     -0.87173279      0.00000000      -0.47538030
    -2.37713566     -0.76589740      0.00000000      -0.43752731
    -2.49323545      0.59060577      0.00000000      -2.73159038
    -2.38740004      0.59060577      0.00000000      -3.61189760
    -2.44031774      0.53768808      0.00000000       1.42153402
```

	-2.44031774	0.64352347	0.00000000	1.38121644
	-1.33902835	1.35334636	0.00000000	7.67003930
	-1.23319294	1.35334636	0.00000000	8.40844617
	-1.28611065	1.30042866	0.00000000	13.81398802
	-1.28611065	1.40626406	0.00000000	11.39029327
45	13555.0 # 737.7 nm,	(r7r2v5) 45		
	0.172274	-0.04041	0.00000000 1.0	# electr. mom.
	0.00000000	0.00000000	-0.8485163126	# magnet. mom.
	1.31876649	-1.08237463	0.00000000	11.66408756
	0.00000000	-0.70208822	0.00000000	5.64505819
	2.11091906	0.04406596	0.00000000	4.99705785
	1.32524096	1.15754064	0.00000000	-7.22591387
	-0.04178899	0.70146698	0.00000000	63.46455156
	-1.16182154	-1.48163476	0.00000000	-66.67465523
	-2.37713566	-0.81881509	0.00000000	37.80395669
	-2.44031774	0.59060577	0.00000000	-16.44046706
	-1.28611065	1.35334636	0.00000000	-29.61921318
	1.26584879	-1.08237463	0.00000000	-3.58628067
	1.37168419	-1.08237463	0.00000000	-2.55471567
	1.31876649	-1.13529233	0.00000000	-2.17695618
	1.31876649	-1.02945693	0.00000000	-3.54496819
	-0.05291770	-0.70208822	0.00000000	24.65770911
	0.05291770	-0.70208822	0.00000000	25.34599288
	0.00000000	-0.75500592	0.00000000	-25.92415098
	0.00000000	-0.64917052	0.00000000	-29.67327703
	2.05800137	0.04406596	0.00000000	-3.39687151
	2.16383676	0.04406596	0.00000000	-3.28194648
	2.11091906	-0.00885174	0.00000000	0.42026173
	2.11091906	0.09698366	0.00000000	1.35170848
	1.27232327	1.15754064	0.00000000	-8.97757474
	1.37815866	1.15754064	0.00000000	-7.77565448
	1.32524096	1.10462294	0.00000000	12.90314097
	1.32524096	1.21045834	0.00000000	11.04579951
	-0.09470669	0.70146698	0.00000000	-32.48990752
	0.01112871	0.70146698	0.00000000	-34.21186265
	-0.04178899	0.64854929	0.00000000	1.24421618
	-0.04178899	0.75438469	0.00000000	2.31399318
	-1.21473924	-1.48163476	0.00000000	23.44981009
	-1.10890384	-1.48163476	0.00000000	24.31301574
	-1.16182154	-1.53455246	0.00000000	9.41828982
	-1.16182154	-1.42871706	0.00000000	9.34513133
	-2.43005336	-0.81881509	0.00000000	-9.83266301
	-2.32421796	-0.81881509	0.00000000	-10.44018049
	-2.37713566	-0.87173279	0.00000000	-8.71570643
	-2.37713566	-0.76589740	0.00000000	-8.65240821
	-2.49323545	0.59060577	0.00000000	8.45334251
	-2.38740004	0.59060577	0.00000000	9.99622884
	-2.44031774	0.53768808	0.00000000	-1.99140239
	-2.44031774	0.64352347	0.00000000	-0.05342388
	-1.33902835	1.35334636	0.00000000	3.30123501
	-1.23319294	1.35334636	0.00000000	3.06277868
	-1.28611065	1.30042866	0.00000000	11.80970085
	-1.28611065	1.40626406	0.00000000	11.23313311
45	15636.0 # 639.5 nm,	(r8r2v5) 45		
	-0.45281	0.064947	0.00000000 1.0	# electr. mom.
	0.00000000	0.00000000	-0.9999114666E-01	# magnet. mom.
	1.31876649	-1.08237463	0.00000000	-24.72728020
	0.00000000	-0.70208822	0.00000000	81.86116339
	2.11091906	0.04406596	0.00000000	11.30452491
	1.32524096	1.15754064	0.00000000	90.41442167
	-0.04178899	0.70146698	0.00000000	-150.69781373
	-1.16182154	-1.48163476	0.00000000	-16.31372213
	-2.37713566	-0.81881509	0.00000000	28.29941023
	-2.44031774	0.59060577	0.00000000	-27.08328907
	-1.28611065	1.35334636	0.00000000	66.69332086
	1.26584879	-1.08237463	0.00000000	2.99396825
	1.37168419	-1.08237463	0.00000000	3.02421134
	1.31876649	-1.13529233	0.00000000	9.19395063
	1.31876649	-1.02945693	0.00000000	9.64827870
	-0.05291770	-0.70208822	0.00000000	-19.31879662
	0.05291770	-0.70208822	0.00000000	-17.17282888
	0.00000000	-0.75500592	0.00000000	-23.16031903
	0.00000000	-0.64917052	0.00000000	-22.43625103
	2.05800137	0.04406596	0.00000000	3.85315835
	2.16383676	0.04406596	0.00000000	3.66954537
	2.11091906	-0.00885174	0.00000000	-12.03195700
	2.11091906	0.09698366	0.00000000	-6.86310784
	1.27232327	1.15754064	0.00000000	-24.69148419
	1.37815866	1.15754064	0.00000000	-23.76515882
	1.32524096	1.10462294	0.00000000	-22.13265590
	1.32524096	1.21045834	0.00000000	-20.00056537
	-0.09470669	0.70146698	0.00000000	60.40099686
	0.01112871	0.70146698	0.00000000	56.43373445
	-0.04178899	0.64854929	0.00000000	15.89488227
	-0.04178899	0.75438469	0.00000000	18.25021816
	-1.21473924	-1.48163476	0.00000000	0.63328410
	-1.10890384	-1.48163476	0.00000000	1.74684358

```
-1.16182154   -1.53455246   0.00000000   5.54612427
-1.16182154   -1.42871706   0.00000000   8.77126999
-2.43005336   -0.81881509   0.00000000   -17.30935303
-2.32421796   -0.81881509   0.00000000   -20.21739952
-2.37713566   -0.87173279   0.00000000   4.20335141
-2.37713566   -0.76589740   0.00000000   4.83503678
-2.49323545   0.59060577   0.00000000   2.97814540
-2.38740004   0.59060577   0.00000000   5.94324462
-2.44031774   0.53768808   0.00000000   9.83008116
-2.44031774   0.64352347   0.00000000   8.69588934
-1.33902835   1.35334636   0.00000000   -17.67658474
-1.23319294   1.35334636   0.00000000   -17.86076411
-1.28611065   1.30042866   0.00000000   -17.91881846
-1.28611065   1.40626406   0.00000000   -13.74090647
&TRANSITION 7->...
0   0.0   # (r2v7r2v6) 45
0.00000000   0.00000000   0.00000000 1.0   # electr. mom.
0.00000000   0.00000000   0.00000000   # magnet. mom.
45   2657.0   # 737.7 nm,   (r3v1r2v6) 45
0.019405   0.036974   0.00000000 1.0   # electr. mom.
0.00000000   0.00000000   -1.664952771   # magnet. mom.
1.31876649   -1.08237463   0.00000000   -58.14989383
0.00000000   -0.70208822   0.00000000   107.78183277
2.11091906   0.04406596   0.00000000   17.16501774
1.32524096   1.15754064   0.00000000   -65.38277075
-0.04178899   0.70146698   0.00000000   114.43202252
-1.16182154   -1.48163476   0.00000000   -35.60112180
-2.37713566   -0.81881509   0.00000000   5.57905334
-2.44031774   0.59060577   0.00000000   5.39595430
-1.28611065   1.35334636   0.00000000   -63.78207722
1.26584879   -1.08237463   0.00000000   14.90547437
1.37168419   -1.08237463   0.00000000   12.79810790
1.31876649   -1.13529233   0.00000000   13.89359212
1.31876649   -1.02945693   0.00000000   16.93990005
-0.05291770   -0.70208822   0.00000000   -26.21502908
0.05291770   -0.70208822   0.00000000   -27.29918341
0.00000000   -0.75500592   0.00000000   -27.55448894
0.00000000   -0.64917052   0.00000000   -27.29062954
2.05800137   0.04406596   0.00000000   -5.07355971
2.16383676   0.04406596   0.00000000   -2.56404401
2.11091906   -0.00885174   0.00000000   -4.49430158
2.11091906   0.09698366   0.00000000   -5.34166339
1.27232327   1.15754064   0.00000000   19.39333277
1.37815866   1.15754064   0.00000000   17.17713214
1.32524096   1.10462294   0.00000000   16.54312219
1.32524096   1.21045834   0.00000000   12.78884107
-0.09470669   0.70146698   0.00000000   -54.80373169
0.01112871   0.70146698   0.00000000   -55.58373510
-0.04178899   0.64854929   0.00000000   -0.52255977
-0.04178899   0.75438469   0.00000000   -3.99974129
-1.21473924   -1.48163476   0.00000000   11.31292292
-1.10890384   -1.48163476   0.00000000   12.55193921
-1.16182154   -1.53455246   0.00000000   5.31853735
-1.16182154   -1.42871706   0.00000000   6.64271383
-2.43005336   -0.81881509   0.00000000   -1.80902543
-2.32421796   -0.81881509   0.00000000   -2.40448370
-2.37713566   -0.87173279   0.00000000   -0.73900029
-2.37713566   -0.76589740   0.00000000   -0.68015609
-2.49323545   0.59060577   0.00000000   -4.24638142
-2.38740004   0.59060577   0.00000000   -5.61485899
-2.44031774   0.53768808   0.00000000   2.20983925
-2.44031774   0.64352347   0.00000000   2.14716374
-1.33902835   1.35334636   0.00000000   11.92342474
-1.23319294   1.35334636   0.00000000   13.07131177
-1.28611065   1.30042866   0.00000000   21.47447228
-1.28611065   1.40626406   0.00000000   17.70672864
45   2657.0   # 737.7 nm,   (r3v2r2v6) 45
0.018043   0.034379   0.00000000 1.0   # electr. mom.
0.00000000   0.00000000   -1.664952771   # magnet. mom.
1.31876649   -1.08237463   0.00000000   -54.06919953
0.00000000   -0.70208822   0.00000000   100.21819538
2.11091906   0.04406596   0.00000000   15.96045509
1.32524096   1.15754064   0.00000000   -60.79450614
-0.04178899   0.70146698   0.00000000   106.40170515
-1.16182154   -1.48163476   0.00000000   -33.10279746
-2.37713566   -0.81881509   0.00000000   5.18754083
-2.44031774   0.59060577   0.00000000   5.01729084
-1.28611065   1.35334636   0.00000000   -59.30614198
1.26584879   -1.08237463   0.00000000   13.85947617
1.37168419   -1.08237463   0.00000000   11.89999507
1.31876649   -1.13529233   0.00000000   12.91860320
1.31876649   -1.02945693   0.00000000   15.75113514
-0.05291770   -0.70208822   0.00000000   -24.37537792
0.05291770   -0.70208822   0.00000000   -25.38345124
0.00000000   -0.75500592   0.00000000   -25.62084059
0.00000000   -0.64917052   0.00000000   -25.37549764
2.05800137   0.04406596   0.00000000   -4.71752043
```

```
   2.16383676    0.04406596    0.00000000    -2.38411110
   2.11091906   -0.00885174    0.00000000    -4.17891200
   2.11091906    0.09698366    0.00000000    -4.96680982
   1.27232327    1.15754064    0.00000000    18.03239713
   1.37815866    1.15754064    0.00000000    15.97171936
   1.32524096    1.10462294    0.00000000    15.38220133
   1.32524096    1.21045834    0.00000000    11.89137854
  -0.09470669    0.70146698    0.00000000   -50.95785578
   0.01112871    0.70146698    0.00000000   -51.68312211
  -0.04178899    0.64854929    0.00000000    -0.48588891
  -0.04178899    0.75438469    0.00000000    -3.71905769
  -1.21473924   -1.48163476    0.00000000    10.51903359
  -1.10890384   -1.48163476    0.00000000    11.67110137
  -1.16182154   -1.53455246    0.00000000     4.94530666
  -1.16182154   -1.42871706    0.00000000     6.17655847
  -2.43005336   -0.81881509    0.00000000    -1.68207627
  -2.32421796   -0.81881509    0.00000000    -2.23574801
  -2.37713566   -0.87173279    0.00000000    -0.68714062
  -2.37713566   -0.76589740    0.00000000    -0.63242583
  -2.49323545    0.59060577    0.00000000    -3.94838974
  -2.38740004    0.59060577    0.00000000    -5.22083380
  -2.44031774    0.53768808    0.00000000     2.05476281
  -2.44031774    0.64352347    0.00000000     1.99648558
  -1.33902835    1.35334636    0.00000000    11.08669318
  -1.23319294    1.35334636    0.00000000    12.15402673
  -1.28611065    1.30042866    0.00000000    19.96749177
  -1.28611065    1.40626406    0.00000000    16.46415119
45   2657.0   # 737.7 nm,   (r3v3r2v6) 45
   0.010894      0.020757      0.00000000 1.0      # electr. mom.
   0.00000000    0.00000000   -1.664952771         # magnet. mom.
   1.31876649   -1.08237463    0.00000000   -32.64555443
   0.00000000   -0.70208822    0.00000000    60.50909910
   2.11091906    0.04406596    0.00000000     9.63650119
   1.32524096    1.15754064    0.00000000   -36.70611691
  -0.04178899    0.70146698    0.00000000    64.24253896
  -1.16182154   -1.48163476    0.00000000   -19.98659469
  -2.37713566   -0.81881509    0.00000000     3.13210012
  -2.44031774    0.59060577    0.00000000     3.02930768
  -1.28611065    1.35334636    0.00000000   -35.80748195
   1.26584879   -1.08237463    0.00000000     8.36798561
   1.37168419   -1.08237463    0.00000000     7.18490268
   1.31876649   -1.13529233    0.00000000     7.79991137
   1.31876649   -1.02945693    0.00000000     9.51011933
  -0.05291770   -0.70208822    0.00000000   -14.71720931
   0.05291770   -0.70208822    0.00000000   -15.32585735
   0.00000000   -0.75500592    0.00000000   -15.46918677
   0.00000000   -0.64917052    0.00000000   -15.32105518
   2.05800137    0.04406596    0.00000000    -2.84831422
   2.16383676    0.04406596    0.00000000    -1.43946330
   2.11091906   -0.00885174    0.00000000    -2.52311668
   2.11091906    0.09698366    0.00000000    -2.99882857
   1.27232327    1.15754064    0.00000000    10.88748506
   1.37815866    1.15754064    0.00000000     9.64330226
   1.32524096    1.10462294    0.00000000     9.28736684
   1.32524096    1.21045834    0.00000000     7.17970025
  -0.09470669    0.70146698    0.00000000   -30.76700726
   0.01112871    0.70146698    0.00000000   -31.20490391
  -0.04178899    0.64854929    0.00000000    -0.29336689
  -0.04178899    0.75438469    0.00000000    -2.24546879
  -1.21473924   -1.48163476    0.00000000     6.35111462
  -1.10890384   -1.48163476    0.00000000     7.04670271
  -1.16182154   -1.53455246    0.00000000     2.98584553
  -1.16182154   -1.42871706    0.00000000     3.72924285
  -2.43005336   -0.81881509    0.00000000    -1.01559322
  -2.32421796   -0.81881509    0.00000000    -1.34988559
  -2.37713566   -0.87173279    0.00000000    -0.41487735
  -2.37713566   -0.76589740    0.00000000    -0.38184201
  -2.49323545    0.59060577    0.00000000    -2.38393343
  -2.38740004    0.59060577    0.00000000    -3.15220154
  -2.44031774    0.53768808    0.00000000     1.24061151
  -2.44031774    0.64352347    0.00000000     1.20542526
  -1.33902835    1.35334636    0.00000000     6.69385248
  -1.23319294    1.35334636    0.00000000     7.33828029
  -1.28611065    1.30042866    0.00000000    12.05584409
  -1.28611065    1.40626406    0.00000000     9.94061959
45   2657.0   # 737.7 nm,   (r3v4r2v6) 45
   0.011575      0.022055      0.00000000 1.0      # electr. mom.
   0.00000000    0.00000000   -1.664952771         # magnet. mom.
   1.31876649   -1.08237463    0.00000000   -34.68590159
   0.00000000   -0.70208822    0.00000000    64.29091779
   2.11091906    0.04406596    0.00000000    10.23878251
   1.32524096    1.15754064    0.00000000   -39.00024922
  -0.04178899    0.70146698    0.00000000    68.25769765
  -1.16182154   -1.48163476    0.00000000   -21.23575686
  -2.37713566   -0.81881509    0.00000000     3.32785638
  -2.44031774    0.59060577    0.00000000     3.21863941
  -1.28611065    1.35334636    0.00000000   -38.04544957
```

1.26584879	-1.08237463	0.00000000	8.89098471	
1.37168419	-1.08237463	0.00000000	7.63395910	
1.31876649	-1.13529233	0.00000000	8.28740583	
1.31876649	-1.02945693	0.00000000	10.10450179	
-0.05291770	-0.70208822	0.00000000	-15.63703489	
0.05291770	-0.70208822	0.00000000	-16.28372344	
0.00000000	-0.75500592	0.00000000	-16.43601095	
0.00000000	-0.64917052	0.00000000	-16.27862113	
2.05800137	0.04406596	0.00000000	-3.02633386	
2.16383676	0.04406596	0.00000000	-1.52942976	
2.11091906	-0.00885174	0.00000000	-2.68081147	
2.11091906	0.09698366	0.00000000	-3.18625535	
1.27232327	1.15754064	0.00000000	11.56795288	
1.37815866	1.15754064	0.00000000	10.24600865	
1.32524096	1.10462294	0.00000000	9.86782727	
1.32524096	1.21045834	0.00000000	7.62843152	
-0.09470669	0.70146698	0.00000000	-32.68994522	
0.01112871	0.70146698	0.00000000	-33.15521041	
-0.04178899	0.64854929	0.00000000	-0.31170232	
-0.04178899	0.75438469	0.00000000	-2.38581059	
-1.21473924	-1.48163476	0.00000000	6.74805929	
-1.10890384	-1.48163476	0.00000000	7.48712163	
-1.16182154	-1.53455246	0.00000000	3.17246087	
-1.16182154	-1.42871706	0.00000000	3.96232053	
-2.43005336	-0.81881509	0.00000000	-1.07906780	
-2.32421796	-0.81881509	0.00000000	-1.43425344	
-2.37713566	-0.87173279	0.00000000	-0.44080719	
-2.37713566	-0.76589740	0.00000000	-0.40570714	
-2.49323545	0.59060577	0.00000000	-2.53292927	
-2.38740004	0.59060577	0.00000000	-3.34921413	
-2.44031774	0.53768808	0.00000000	1.31814973	
-2.44031774	0.64352347	0.00000000	1.28076434	
-1.33902835	1.35334636	0.00000000	7.11221826	
-1.23319294	1.35334636	0.00000000	7.79692281	
-1.28611065	1.30042866	0.00000000	12.80933434	
-1.28611065	1.40626406	0.00000000	10.56190831	
45	2657.0	# 737.7 nm,	(r3v5r2v6) 45	
0.010213	0.01946	0.00000000 1.0	# electr. mom.	
0.00000000	0.00000000	-1.664952771	# magnet. mom.	
1.31876649	-1.08237463	0.00000000	-30.60520728	
0.00000000	-0.70208822	0.00000000	56.72728040	
2.11091906	0.04406596	0.00000000	9.03421986	
1.32524096	1.15754064	0.00000000	-34.41198461	
-0.04178899	0.70146698	0.00000000	60.22738028	
-1.16182154	-1.48163476	0.00000000	-18.73743253	
-2.37713566	-0.81881509	0.00000000	2.93634387	
-2.44031774	0.59060577	0.00000000	2.83997595	
-1.28611065	1.35334636	0.00000000	-33.56951433	
1.26584879	-1.08237463	0.00000000	7.84498651	
1.37168419	-1.08237463	0.00000000	6.73584627	
1.31876649	-1.13529233	0.00000000	7.31241691	
1.31876649	-1.02945693	0.00000000	8.91573687	
-0.05291770	-0.70208822	0.00000000	-13.79738373	
0.05291770	-0.70208822	0.00000000	-14.36799127	
0.00000000	-0.75500592	0.00000000	-14.50236260	
0.00000000	-0.64917052	0.00000000	-14.36348923	
2.05800137	0.04406596	0.00000000	-2.67029459	
2.16383676	0.04406596	0.00000000	-1.34949685	
2.11091906	-0.00885174	0.00000000	-2.36542188	
2.11091906	0.09698366	0.00000000	-2.81140178	
1.27232327	1.15754064	0.00000000	10.20701725	
1.37815866	1.15754064	0.00000000	9.04059586	
1.32524096	1.10462294	0.00000000	8.70690641	
1.32524096	1.21045834	0.00000000	6.73096898	
-0.09470669	0.70146698	0.00000000	-28.84406931	
0.01112871	0.70146698	0.00000000	-29.25459742	
-0.04178899	0.64854929	0.00000000	-0.27503146	
-0.04178899	0.75438469	0.00000000	-2.10512699	
-1.21473924	-1.48163476	0.00000000	5.95416996	
-1.10890384	-1.48163476	0.00000000	6.60628379	
-1.16182154	-1.53455246	0.00000000	2.79923018	
-1.16182154	-1.42871706	0.00000000	3.49616517	
-2.43005336	-0.81881509	0.00000000	-0.95211865	
-2.32421796	-0.81881509	0.00000000	-1.26551774	
-2.37713566	-0.87173279	0.00000000	-0.38894752	
-2.37713566	-0.76589740	0.00000000	-0.35797689	
-2.49323545	0.59060577	0.00000000	-2.23493759	
-2.38740004	0.59060577	0.00000000	-2.95518894	
-2.44031774	0.53768808	0.00000000	1.16307329	
-2.44031774	0.64352347	0.00000000	1.13008618	
-1.33902835	1.35334636	0.00000000	6.27548670	
-1.23319294	1.35334636	0.00000000	6.87963777	
-1.28611065	1.30042866	0.00000000	11.30235383	
-1.28611065	1.40626406	0.00000000	9.31933086	
45	2657.0	# 737.7 nm,	(r3v6r2v6) 45	
0.006128	0.011676	0.00000000 1.0	# electr. mom.	
0.00000000	0.00000000	-1.664952771	# magnet. mom.	

1.31876649	-1.08237463	0.00000000	-18.36312437
0.00000000	-0.70208822	0.00000000	34.03636824
2.11091906	0.04406596	0.00000000	5.42053192
1.32524096	1.15754064	0.00000000	-20.64719076
-0.04178899	0.70146698	0.00000000	36.13642817
-1.16182154	-1.48163476	0.00000000	-11.24245952
-2.37713566	-0.81881509	0.00000000	1.76180632
-2.44031774	0.59060577	0.00000000	1.70398557
-1.28611065	1.35334636	0.00000000	-20.14170860
1.26584879	-1.08237463	0.00000000	4.70699191
1.37168419	-1.08237463	0.00000000	4.04150776
1.31876649	-1.13529233	0.00000000	4.38745014
1.31876649	-1.02945693	0.00000000	5.34944212
-0.05291770	-0.70208822	0.00000000	-8.27843024
0.05291770	-0.70208822	0.00000000	-8.62079476
0.00000000	-0.75500592	0.00000000	-8.70141756
0.00000000	-0.64917052	0.00000000	-8.61809354
2.05800137	0.04406596	0.00000000	-1.60217675
2.16383676	0.04406596	0.00000000	-0.80969811
2.11091906	-0.00885174	0.00000000	-1.41925313
2.11091906	0.09698366	0.00000000	-1.68684107
1.27232327	1.15754064	0.00000000	6.12421035
1.37815866	1.15754064	0.00000000	5.42435752
1.32524096	1.10462294	0.00000000	5.22414385
1.32524096	1.21045834	0.00000000	4.03858139
-0.09470669	0.70146698	0.00000000	-17.30644159
0.01112871	0.70146698	0.00000000	-17.55275845
-0.04178899	0.64854929	0.00000000	-0.16501888
-0.04178899	0.75438469	0.00000000	-1.26307620
-1.21473924	-1.48163476	0.00000000	3.57250197
-1.10890384	-1.48163476	0.00000000	3.96377028
-1.16182154	-1.53455246	0.00000000	1.67953811
-1.16182154	-1.42871706	0.00000000	2.09769910
-2.43005336	-0.81881509	0.00000000	-0.57127119
-2.32421796	-0.81881509	0.00000000	-0.75931064
-2.37713566	-0.87173279	0.00000000	-0.23336851
-2.37713566	-0.76589740	0.00000000	-0.21478613
-2.49323545	0.59060577	0.00000000	-1.34096255
-2.38740004	0.59060577	0.00000000	-1.77311337
-2.44031774	0.53768808	0.00000000	0.69784397
-2.44031774	0.64352347	0.00000000	0.67805171
-1.33902835	1.35334636	0.00000000	3.76529202
-1.23319294	1.35334636	0.00000000	4.12778266
-1.28611065	1.30042866	0.00000000	6.78141230
-1.28611065	1.40626406	0.00000000	5.59159852

45 2657.0 # 737.7 nm, (r3v7r2v6) 45

0.006468	0.012325	0.00000000 1.0	# electr. mom.
0.00000000	0.00000000	-1.664952771	# magnet. mom.
1.31876649	-1.08237463	0.00000000	-19.38329794
0.00000000	-0.70208822	0.00000000	35.92727759
2.11091906	0.04406596	0.00000000	5.72167258
1.32524096	1.15754064	0.00000000	-21.79425692
-0.04178899	0.70146698	0.00000000	38.14400751
-1.16182154	-1.48163476	0.00000000	-11.86704060
-2.37713566	-0.81881509	0.00000000	1.85968445
-2.44031774	0.59060577	0.00000000	1.79865143
-1.28611065	1.35334636	0.00000000	-21.26069241
1.26584879	-1.08237463	0.00000000	4.96849146
1.37168419	-1.08237463	0.00000000	4.26603597
1.31876649	-1.13529233	0.00000000	4.63119737
1.31876649	-1.02945693	0.00000000	5.64663335
-0.05291770	-0.70208822	0.00000000	-8.73834303
0.05291770	-0.70208822	0.00000000	-9.09972780
0.00000000	-0.75500592	0.00000000	-9.18482965
0.00000000	-0.64917052	0.00000000	-9.09687651
2.05800137	0.04406596	0.00000000	-1.69118657
2.16383676	0.04406596	0.00000000	-0.85468134
2.11091906	-0.00885174	0.00000000	-1.49810053
2.11091906	0.09698366	0.00000000	-1.78055446
1.27232327	1.15754064	0.00000000	6.46444426
1.37815866	1.15754064	0.00000000	5.72571071
1.32524096	1.10462294	0.00000000	5.51437406
1.32524096	1.21045834	0.00000000	4.26294702
-0.09470669	0.70146698	0.00000000	-18.26791056
0.01112871	0.70146698	0.00000000	-18.52791170
-0.04178899	0.64854929	0.00000000	-0.17418659
-0.04178899	0.75438469	0.00000000	-1.33324710
-1.21473924	-1.48163476	0.00000000	3.77097431
-1.10890384	-1.48163476	0.00000000	4.18397974
-1.16182154	-1.53455246	0.00000000	1.77284578
-1.16182154	-1.42871706	0.00000000	2.21423794
-2.43005336	-0.81881509	0.00000000	-0.60300848
-2.32421796	-0.81881509	0.00000000	-0.80149457
-2.37713566	-0.87173279	0.00000000	-0.24633343
-2.37713566	-0.76589740	0.00000000	-0.22671870
-2.49323545	0.59060577	0.00000000	-1.41546047
-2.38740004	0.59060577	0.00000000	-1.87161966

```
   -2.44031774     0.53768808     0.00000000      0.73661308
   -2.44031774     0.64352347     0.00000000      0.71572125
   -1.33902835     1.35334636     0.00000000      3.97447491
   -1.23319294     1.35334636     0.00000000      4.35710392
   -1.28611065     1.30042866     0.00000000      7.15815743
   -1.28611065     1.40626406     0.00000000      5.90224288
45   2657.0   # 737.7 nm,   (r3v8r2v6) 45
    0.002723       0.005189       0.00000000 1.0     # electr. mom.
    0.00000000     0.00000000    -1.664952771        # magnet. mom.
    1.31876649    -1.08237463     0.00000000     -8.16138861
    0.00000000    -0.70208822     0.00000000     15.12727477
    2.11091906     0.04406596     0.00000000      2.40912530
    1.32524096     1.15754064     0.00000000     -9.17652923
   -0.04178899     0.70146698     0.00000000     16.06063474
   -1.16182154    -1.48163476     0.00000000     -4.99664867
   -2.37713566    -0.81881509     0.00000000      0.78302503
   -2.44031774     0.59060577     0.00000000      0.75732692
   -1.28611065     1.35334636     0.00000000     -8.95187049
    1.26584879    -1.08237463     0.00000000      2.09199640
    1.37168419    -1.08237463     0.00000000      1.79622567
    1.31876649    -1.13529233     0.00000000      1.94997784
    1.31876649    -1.02945693     0.00000000      2.37752983
   -0.05291770    -0.70208822     0.00000000     -3.67930233
    0.05291770    -0.70208822     0.00000000     -3.83146434
    0.00000000    -0.75500592     0.00000000     -3.86729669
    0.00000000    -0.64917052     0.00000000     -3.83026380
    2.05800137     0.04406596     0.00000000     -0.71207856
    2.16383676     0.04406596     0.00000000     -0.35986583
    2.11091906    -0.00885174     0.00000000     -0.63077917
    2.11091906     0.09698366     0.00000000     -0.74970714
    1.27232327     1.15754064     0.00000000      2.72187127
    1.37815866     1.15754064     0.00000000      2.41082556
    1.32524096     1.10462294     0.00000000      2.32184171
    1.32524096     1.21045834     0.00000000      1.79492506
   -0.09470669     0.70146698     0.00000000     -7.69175182
    0.01112871     0.70146698     0.00000000     -7.80122598
   -0.04178899     0.64854929     0.00000000     -0.07334172
   -0.04178899     0.75438469     0.00000000     -0.56136720
   -1.21473924    -1.48163476     0.00000000      1.58777866
   -1.10890384    -1.48163476     0.00000000      1.76167568
   -1.16182154    -1.53455246     0.00000000      0.74646138
   -1.16182154    -1.42871706     0.00000000      0.93231071
   -2.43005336    -0.81881509     0.00000000     -0.25389831
   -2.32421796    -0.81881509     0.00000000     -0.33747140
   -2.37713566    -0.87173279     0.00000000     -0.10371934
   -2.37713566    -0.76589740     0.00000000     -0.09546050
   -2.49323545     0.59060577     0.00000000     -0.59598336
   -2.38740004     0.59060577     0.00000000     -0.78805038
   -2.44031774     0.53768808     0.00000000      0.31015288
   -2.44031774     0.64352347     0.00000000      0.30135631
   -1.33902835     1.35334636     0.00000000      1.67346312
   -1.23319294     1.35334636     0.00000000      1.83457007
   -1.28611065     1.30042866     0.00000000      3.01396102
   -1.28611065     1.40626406     0.00000000      2.48515490
45   2657.0   # 737.7 nm,   (r3v9r2v6) 45
    0.003404       0.006487       0.00000000 1.0     # electr. mom.
    0.00000000     0.00000000    -1.664952771        # magnet. mom.
    1.31876649    -1.08237463     0.00000000    -10.20173576
    0.00000000    -0.70208822     0.00000000     18.90909347
    2.11091906     0.04406596     0.00000000      3.01140662
    1.32524096     1.15754064     0.00000000    -11.47066154
   -0.04178899     0.70146698     0.00000000     20.07579343
   -1.16182154    -1.48163476     0.00000000     -6.24581084
   -2.37713566    -0.81881509     0.00000000      0.97878129
   -2.44031774     0.59060577     0.00000000      0.94665865
   -1.28611065     1.35334636     0.00000000    -11.18983811
    1.26584879    -1.08237463     0.00000000      2.61499550
    1.37168419    -1.08237463     0.00000000      2.24528209
    1.31876649    -1.13529233     0.00000000      2.43747230
    1.31876649    -1.02945693     0.00000000      2.97191229
   -0.05291770    -0.70208822     0.00000000     -4.59912791
    0.05291770    -0.70208822     0.00000000     -4.78933042
    0.00000000    -0.75500592     0.00000000     -4.83412087
    0.00000000    -0.64917052     0.00000000     -4.78782974
    2.05800137     0.04406596     0.00000000     -0.89009820
    2.16383676     0.04406596     0.00000000     -0.44983228
    2.11091906    -0.00885174     0.00000000     -0.78847396
    2.11091906     0.09698366     0.00000000     -0.93713393
    1.27232327     1.15754064     0.00000000      3.40233908
    1.37815866     1.15754064     0.00000000      3.01353195
    1.32524096     1.10462294     0.00000000      2.90230214
    1.32524096     1.21045834     0.00000000      2.24365633
   -0.09470669     0.70146698     0.00000000     -9.61468977
    0.01112871     0.70146698     0.00000000     -9.75153247
   -0.04178899     0.64854929     0.00000000     -0.09167715
   -0.04178899     0.75438469     0.00000000     -0.70170900
   -1.21473924    -1.48163476     0.00000000      1.98472332
```

```
   -1.10890384    -1.48163476    0.00000000    2.20209460
   -1.16182154    -1.53455246    0.00000000    0.93307673
   -1.16182154    -1.42871706    0.00000000    1.16538839
   -2.43005336    -0.81881509    0.00000000   -0.31737288
   -2.32421796    -0.81881509    0.00000000   -0.42183925
   -2.37713566    -0.87173279    0.00000000   -0.12964917
   -2.37713566    -0.76589740    0.00000000   -0.11932563
   -2.49323545     0.59060577    0.00000000   -0.74497920
   -2.38740004     0.59060577    0.00000000   -0.98506298
   -2.44031774     0.53768808    0.00000000    0.38769110
   -2.44031774     0.64352347    0.00000000    0.37669539
   -1.33902835     1.35334636    0.00000000    2.09182890
   -1.23319294     1.35334636    0.00000000    2.29321259
   -1.28611065     1.30042866    0.00000000    3.76745128
   -1.28611065     1.40626406    0.00000000    3.10644362
45   13555.0    # 737.7 nm,    (r7r2v6) 45
    0.046984      -0.01102       0.00000000 1.0     # electr. mom.
    0.00000000     0.00000000   -0.8485163126      # magnet. mom.
    1.31876649    -1.08237463    0.00000000    3.18111479
    0.00000000    -0.70208822    0.00000000    1.53956132
    2.11091906     0.04406596    0.00000000    1.36283396
    1.32524096     1.15754064    0.00000000   -1.97070378
   -0.04178899     0.70146698    0.00000000   17.30851406
   -1.16182154    -1.48163476    0.00000000  -18.18399688
   -2.37713566    -0.81881509    0.00000000   10.31017001
   -2.44031774     0.59060577    0.00000000   -4.48376374
   -1.28611065     1.35334636    0.00000000   -8.07796723
    1.26584879    -1.08237463    0.00000000   -0.97807655
    1.37168419    -1.08237463    0.00000000   -0.69674064
    1.31876649    -1.13529233    0.00000000   -0.59371532
    1.31876649    -1.02945693    0.00000000   -0.96680951
   -0.05291770    -0.70208822    0.00000000    6.72482976
    0.05291770    -0.70208822    0.00000000    6.91254351
    0.00000000    -0.75500592    0.00000000   -7.07022299
    0.00000000    -0.64917052    0.00000000   -8.09271192
    2.05800137     0.04406596    0.00000000   -0.92641950
    2.16383676     0.04406596    0.00000000   -0.89507631
    2.11091906    -0.00885174    0.00000000    0.11461684
    2.11091906     0.09698366    0.00000000    0.36864777
    1.27232327     1.15754064    0.00000000   -2.44842947
    1.37815866     1.15754064    0.00000000   -2.12063304
    1.32524096     1.10462294    0.00000000    3.51903845
    1.32524096     1.21045834    0.00000000    3.01249078
   -0.09470669     0.70146698    0.00000000   -8.86088387
    0.01112871     0.70146698    0.00000000   -9.33050800
   -0.04178899     0.64854929    0.00000000    0.33933168
   -0.04178899     0.75438469    0.00000000    0.63108905
   -1.21473924    -1.48163476    0.00000000    6.39540275
   -1.10890384    -1.48163476    0.00000000    6.63082248
   -1.16182154    -1.53455246    0.00000000    2.56862450
   -1.16182154    -1.42871706    0.00000000    2.54867218
   -2.43005336    -0.81881509    0.00000000   -2.68163537
   -2.32421796    -0.81881509    0.00000000   -2.84732195
   -2.37713566    -0.87173279    0.00000000   -2.37701085
   -2.37713566    -0.76589740    0.00000000   -2.35974769
   -2.49323545     0.59060577    0.00000000    2.30545705
   -2.38740004     0.59060577    0.00000000    2.72624423
   -2.44031774     0.53768808    0.00000000   -0.54310974
   -2.44031774     0.64352347    0.00000000   -0.01457015
   -1.33902835     1.35334636    0.00000000    0.90033682
   -1.23319294     1.35334636    0.00000000    0.83530328
   -1.28611065     1.30042866    0.00000000    3.22082750
   -1.28611065     1.40626406    0.00000000    3.06358176
45   15636.0    # 639.5 nm,    (r8r2v6) 45
   -0.12349        0.017713      0.00000000 1.0     # electr. mom.
    0.00000000     0.00000000   -0.9999114666E-01    # magnet. mom.
    1.31876649    -1.08237463    0.00000000   -6.74380369
    0.00000000    -0.70208822    0.00000000   22.32577183
    2.11091906     0.04406596    0.00000000    3.08305225
    1.32524096     1.15754064    0.00000000   24.65847864
   -0.04178899     0.70146698    0.00000000  -41.09940375
   -1.16182154    -1.48163476    0.00000000   -4.44919694
   -2.37713566    -0.81881509    0.00000000    7.71802097
   -2.44031774     0.59060577    0.00000000   -7.38635156
   -1.28611065     1.35334636    0.00000000   18.18908751
    1.26584879    -1.08237463    0.00000000    0.81653680
    1.37168419    -1.08237463    0.00000000    0.82478491
    1.31876649    -1.13529233    0.00000000    2.50744108
    1.31876649    -1.02945693    0.00000000    2.63134874
   -0.05291770    -0.70208822    0.00000000   -5.26876272
    0.05291770    -0.70208822    0.00000000   -4.68349878
    0.00000000    -0.75500592    0.00000000   -6.31645064
    0.00000000    -0.64917052    0.00000000   -6.11897755
    2.05800137     0.04406596    0.00000000    1.05086137
    2.16383676     0.04406596    0.00000000    1.00078510
    2.11091906    -0.00885174    0.00000000   -3.28144282
    2.11091906     0.09698366    0.00000000   -1.87175668
```

1.27232327 1.15754064 0.00000000 -6.73404114
1.37815866 1.15754064 0.00000000 -6.48140695
1.32524096 1.10462294 0.00000000 -6.03617888
1.32524096 1.21045834 0.00000000 -5.45469965
-0.09470669 0.70146698 0.00000000 16.47299914
0.01112871 0.70146698 0.00000000 15.39101849
-0.04178899 0.64854929 0.00000000 4.33496789
-0.04178899 0.75438469 0.00000000 4.97733222
-1.21473924 -1.48163476 0.00000000 0.17271385
-1.10890384 -1.48163476 0.00000000 0.47641189
-1.16182154 -1.53455246 0.00000000 1.51257935
-1.16182154 -1.42871706 0.00000000 2.39216454
-2.43005336 -0.81881509 0.00000000 -4.72073264
-2.32421796 -0.81881509 0.00000000 -5.51383623
-2.37713566 -0.87173279 0.00000000 1.14636857
-2.37713566 -0.76589740 0.00000000 1.31864639
-2.49323545 0.59060577 0.00000000 0.81222147
-2.38740004 0.59060577 0.00000000 1.62088490
-2.44031774 0.53768808 0.00000000 2.68093123
-2.44031774 0.64352347 0.00000000 2.37160618
-1.33902835 1.35334636 0.00000000 -4.82088675
-1.23319294 1.35334636 0.00000000 -4.87111749
-1.28611065 1.30042866 0.00000000 -4.88695049
-1.28611065 1.40626406 0.00000000 -3.74751995
&TRANSITION 8->...
45 2657.0 # 737.7 nm, (r3v1r2v7) 45
0.0036654 0.06984 0.00000000 1.0 # electr. mom.
0.00000000 0.00000000 -1.664952771 # magnet. mom.
1.31876649 -1.08237463 0.00000000 -109.83868835
0.00000000 -0.70208822 0.00000000 203.58790634
2.11091906 0.04406596 0.00000000 32.42281129
1.32524096 1.15754064 0.00000000 -123.50078920
-0.04178899 0.70146698 0.00000000 216.14937588
-1.16182154 -1.48163476 0.00000000 -67.24656340
-2.37713566 -0.81881509 0.00000000 10.53821187
-2.44031774 0.59060577 0.00000000 10.19235812
-1.28611065 1.35334636 0.00000000 -120.47725697
1.26584879 -1.08237463 0.00000000 28.15478492
1.37168419 -1.08237463 0.00000000 24.17420382
1.31876649 -1.13529233 0.00000000 26.24345179
1.31876649 -1.02945693 0.00000000 31.99758899
-0.05291770 -0.70208822 0.00000000 -49.51727716
0.05291770 -0.70208822 0.00000000 -51.56512422
0.00000000 -0.75500592 0.00000000 -52.04736800
0.00000000 -0.64917052 0.00000000 -51.54896691
2.05800137 0.04406596 0.00000000 -9.58339057
2.16383676 0.04406596 0.00000000 -4.84319424
2.11091906 -0.00885174 0.00000000 -8.48923632
2.11091906 0.09698366 0.00000000 -10.08980862
1.27232327 1.15754064 0.00000000 36.63185078
1.37815866 1.15754064 0.00000000 32.44569405
1.32524096 1.10462294 0.00000000 31.24811969
1.32524096 1.21045834 0.00000000 24.15669980
-0.09470669 0.70146698 0.00000000 -103.51815985
0.01112871 0.70146698 0.00000000 -104.99149963
-0.04178899 0.64854929 0.00000000 -0.98705735
-0.04178899 0.75438469 0.00000000 -7.55506687
-1.21473924 -1.48163476 0.00000000 21.36885440
-1.10890384 -1.48163476 0.00000000 23.70921850
-1.16182154 -1.53455246 0.00000000 10.04612610
-1.16182154 -1.42871706 0.00000000 12.54734834
-2.43005336 -0.81881509 0.00000000 -3.41704803
-2.32421796 -0.81881509 0.00000000 -4.54180255
-2.37713566 -0.87173279 0.00000000 -1.39588943
-2.37713566 -0.76589740 0.00000000 -1.28473927
-2.49323545 0.59060577 0.00000000 -8.02094267
-2.38740004 0.59060577 0.00000000 -10.60584476
-2.44031774 0.53768808 0.00000000 4.17414081
-2.44031774 0.64352347 0.00000000 4.05575373
-1.33902835 1.35334636 0.00000000 22.52202450
-1.23319294 1.35334636 0.00000000 24.69025556
-1.28611065 1.30042866 0.00000000 40.56289208
-1.28611065 1.40626406 0.00000000 33.44604298
45 2657.0 # 737.7 nm, (r3v2r2v7) 45
0.034081 0.064939 0.00000000 1.0 # electr. mom.
0.00000000 0.00000000 -1.664952771 # magnet. mom.
1.31876649 -1.08237463 0.00000000 -102.13071022
0.00000000 -0.70208822 0.00000000 189.30103572
2.11091906 0.04406596 0.00000000 30.14752629
1.32524096 1.15754064 0.00000000 -114.83406715
-0.04178899 0.70146698 0.00000000 200.98099862
-1.16182154 -1.48163476 0.00000000 -62.52750632
-2.37713566 -0.81881509 0.00000000 9.79868823
-2.44031774 0.59060577 0.00000000 9.47710492
-1.28611065 1.35334636 0.00000000 -112.02271262
1.26584879 -1.08237463 0.00000000 26.17901054
1.37168419 -1.08237463 0.00000000 22.47776846

```
1.31876649   -1.13529233   0.00000000   24.40180605
1.31876649   -1.02945693   0.00000000   29.75214415
-0.05291770   -0.70208822   0.00000000   -46.04238052
0.05291770   -0.70208822   0.00000000   -47.94651901
0.00000000   -0.75500592   0.00000000   -48.39492112
0.00000000   -0.64917052   0.00000000   -47.93149555
2.05800137   0.04406596   0.00000000   -8.91087193
2.16383676   0.04406596   0.00000000   -4.50332096
2.11091906   -0.00885174   0.00000000   -7.89350044
2.11091906   0.09698366   0.00000000   -9.38175187
1.27232327   1.15754064   0.00000000   34.06119459
1.37815866   1.15754064   0.00000000   30.16880324
1.32524096   1.10462294   0.00000000   29.05526918
1.32524096   1.21045834   0.00000000   22.46149280
-0.09470669   0.70146698   0.00000000   -96.25372758
0.01112871   0.70146698   0.00000000   -97.62367509
-0.04178899   0.64854929   0.00000000   -0.91779017
-0.04178899   0.75438469   0.00000000   -7.02488674
-1.21473924   -1.48163476   0.00000000   19.86928567
-1.10890384   -1.48163476   0.00000000   22.04541370
-1.16182154   -1.53455246   0.00000000   9.34113480
-1.16182154   -1.42871706   0.00000000   11.66683266
-2.43005336   -0.81881509   0.00000000   -3.17725518
-2.32421796   -0.81881509   0.00000000   -4.22307957
-2.37713566   -0.87173279   0.00000000   -1.29793227
-2.37713566   -0.76589740   0.00000000   -1.19458213
-2.49323545   0.59060577   0.00000000   -7.45806950
-2.38740004   0.59060577   0.00000000   -9.86157495
-2.44031774   0.53768808   0.00000000   3.88121864
-2.44031774   0.64352347   0.00000000   3.77113944
-1.33902835   1.35334636   0.00000000   20.94153155
-1.23319294   1.35334636   0.00000000   22.95760604
-1.28611065   1.30042866   0.00000000   37.71637334
-1.28611065   1.40626406   0.00000000   31.09895224
45   2657.0   # 737.7 nm,   (r3v3r2v7) 45
0.020578   0.039208   0.00000000 1.0   # electr. mom.
0.00000000   0.00000000   -1.664952771   # magnet. mom.
1.31876649   -1.08237463   0.00000000   -61.66382504
0.00000000   -0.70208822   0.00000000   114.29496496
2.11091906   0.04406596   0.00000000   18.20228002
1.32524096   1.15754064   0.00000000   -69.33377639
-0.04178899   0.70146698   0.00000000   121.34701804
-1.16182154   -1.48163476   0.00000000   -37.75245664
-2.37713566   -0.81881509   0.00000000   5.91618912
-2.44031774   0.59060577   0.00000000   5.72202561
-1.28611065   1.35334636   0.00000000   -67.63635479
1.26584879   -1.08237463   0.00000000   15.80619504
1.37168419   -1.08237463   0.00000000   13.57148285
1.31876649   -1.13529233   0.00000000   14.73316592
1.31876649   -1.02945693   0.00000000   17.96355873
-0.05291770   -0.70208822   0.00000000   -27.79917314
0.05291770   -0.70208822   0.00000000   -28.94884166
0.00000000   -0.75500592   0.00000000   -29.21957502
0.00000000   -0.64917052   0.00000000   -28.93977090
2.05800137   0.04406596   0.00000000   -5.38014909
2.16383676   0.04406596   0.00000000   -2.71898624
2.11091906   -0.00885174   0.00000000   -4.76588706
2.11091906   0.09698366   0.00000000   -5.66445396
1.27232327   1.15754064   0.00000000   20.56524956
1.37815866   1.15754064   0.00000000   18.21512648
1.32524096   1.10462294   0.00000000   17.54280403
1.32524096   1.21045834   0.00000000   13.56165603
-0.09470669   0.70146698   0.00000000   -58.11545816
0.01112871   0.70146698   0.00000000   -58.94259628
-0.04178899   0.64854929   0.00000000   -0.55413746
-0.04178899   0.75438469   0.00000000   -4.24144105
-1.21473924   -1.48163476   0.00000000   11.99654984
-1.10890384   -1.48163476   0.00000000   13.31043846
-1.16182154   -1.53455246   0.00000000   5.63993044
-1.16182154   -1.42871706   0.00000000   7.04412538
-2.43005336   -0.81881509   0.00000000   -1.91834275
-2.32421796   -0.81881509   0.00000000   -2.54978389
-2.37713566   -0.87173279   0.00000000   -0.78365722
-2.37713566   -0.76589740   0.00000000   -0.72125714
-2.49323545   0.59060577   0.00000000   -4.50298536
-2.38740004   0.59060577   0.00000000   -5.95415846
-2.44031774   0.53768808   0.00000000   2.34337729
-2.44031774   0.64352347   0.00000000   2.27691438
-1.33902835   1.35334636   0.00000000   12.64394358
-1.23319294   1.35334636   0.00000000   13.86119610
-1.28611065   1.30042866   0.00000000   22.77214994
-1.28611065   1.40626406   0.00000000   18.77672588
45   2657.0   # 737.7 nm,   (r3v4r2v7) 45
0.021864   0.041659   0.00000000 1.0   # electr. mom.
0.00000000   0.00000000   -1.664952771   # magnet. mom.
1.31876649   -1.08237463   0.00000000   -65.51781411
0.00000000   -0.70208822   0.00000000   121.43840027
```

2.11091906	0.04406596	0.00000000	19.33992253
1.32524096	1.15754064	0.00000000	-73.66713742
-0.04178899	0.70146698	0.00000000	128.93120666
-1.16182154	-1.48163476	0.00000000	-40.11198518
-2.37713566	-0.81881509	0.00000000	6.28595094
-2.44031774	0.59060577	0.00000000	6.07965221
-1.28611065	1.35334636	0.00000000	-71.86362697
1.26584879	-1.08237463	0.00000000	16.79408223
1.37168419	-1.08237463	0.00000000	14.41970052
1.31876649	-1.13529233	0.00000000	15.65398879
1.31876649	-1.02945693	0.00000000	19.08628115
-0.05291770	-0.70208822	0.00000000	-29.53662146
0.05291770	-0.70208822	0.00000000	-30.75814427
0.00000000	-0.75500592	0.00000000	-31.04579846
0.00000000	-0.64917052	0.00000000	-30.74850658
2.05800137	0.04406596	0.00000000	-5.71640841
2.16383676	0.04406596	0.00000000	-2.88892288
2.11091906	-0.00885174	0.00000000	-5.06375500
2.11091906	0.09698366	0.00000000	-6.01848233
1.27232327	1.15754064	0.00000000	21.85057766
1.37815866	1.15754064	0.00000000	19.35357189
1.32524096	1.10462294	0.00000000	18.63922929
1.32524096	1.21045834	0.00000000	14.40925953
-0.09470669	0.70146698	0.00000000	-61.74767430
0.01112871	0.70146698	0.00000000	-62.62650855
-0.04178899	0.64854929	0.00000000	-0.58877105
-0.04178899	0.75438469	0.00000000	-4.50653112
-1.21473924	-1.48163476	0.00000000	12.74633421
-1.10890384	-1.48163476	0.00000000	14.14234086
-1.16182154	-1.53455246	0.00000000	5.99242610
-1.16182154	-1.42871706	0.00000000	7.48438322
-2.43005336	-0.81881509	0.00000000	-2.03823918
-2.32421796	-0.81881509	0.00000000	-2.70914538
-2.37713566	-0.87173279	0.00000000	-0.83263580
-2.37713566	-0.76589740	0.00000000	-0.76633571
-2.49323545	0.59060577	0.00000000	-4.78442195
-2.38740004	0.59060577	0.00000000	-6.32629336
-2.44031774	0.53768808	0.00000000	2.48983838
-2.44031774	0.64352347	0.00000000	2.41922153
-1.33902835	1.35334636	0.00000000	13.43419005
-1.23319294	1.35334636	0.00000000	14.72752086
-1.28611065	1.30042866	0.00000000	24.19540931
-1.28611065	1.40626406	0.00000000	19.95027125

45 2657.0 # 737.7 nm, (r3v5r2v7) 45

0.019291	0.036758	0.00000000 1.0	# electr. mom.
0.00000000	0.00000000	-1.664952771	# magnet. mom.
1.31876649	-1.08237463	0.00000000	-57.80983598
0.00000000	-0.70208822	0.00000000	107.15152965
2.11091906	0.04406596	0.00000000	17.06463752
1.32524096	1.15754064	0.00000000	-65.00041537
-0.04178899	0.70146698	0.00000000	113.76282941
-1.16182154	-1.48163476	0.00000000	-35.39292810
-2.37713566	-0.81881509	0.00000000	5.54642730
-2.44031774	0.59060577	0.00000000	5.36439901
-1.28611065	1.35334636	0.00000000	-63.40908262
1.26584879	-1.08237463	0.00000000	14.81830785
1.37168419	-1.08237463	0.00000000	12.72326517
1.31876649	-1.13529233	0.00000000	13.81234305
1.31876649	-1.02945693	0.00000000	16.84083631
-0.05291770	-0.70208822	0.00000000	-26.06172482
0.05291770	-0.70208822	0.00000000	-27.13953906
0.00000000	-0.75500592	0.00000000	-27.39335158
0.00000000	-0.64917052	0.00000000	-27.13103522
2.05800137	0.04406596	0.00000000	-5.04388977
2.16383676	0.04406596	0.00000000	-2.54904960
2.11091906	-0.00885174	0.00000000	-4.46801911
2.11091906	0.09698366	0.00000000	-5.31042559
1.27232327	1.15754064	0.00000000	19.27992146
1.37815866	1.15754064	0.00000000	17.07668108
1.32524096	1.10462294	0.00000000	16.44637878
1.32524096	1.21045834	0.00000000	12.71405253
-0.09470669	0.70146698	0.00000000	-54.48324203
0.01112871	0.70146698	0.00000000	-55.25868401
-0.04178899	0.64854929	0.00000000	-0.51950387
-0.04178899	0.75438469	0.00000000	-3.97635099
-1.21473924	-1.48163476	0.00000000	11.24676548
-1.10890384	-1.48163476	0.00000000	12.47853606
-1.16182154	-1.53455246	0.00000000	5.28743479
-1.16182154	-1.42871706	0.00000000	6.60386755
-2.43005336	-0.81881509	0.00000000	-1.79844633
-2.32421796	-0.81881509	0.00000000	-2.39042240
-2.37713566	-0.87173279	0.00000000	-0.73467865
-2.37713566	-0.76589740	0.00000000	-0.67617856
-2.49323545	0.59060577	0.00000000	-4.22154878
-2.38740004	0.59060577	0.00000000	-5.58202356
-2.44031774	0.53768808	0.00000000	2.19691621
-2.44031774	0.64352347	0.00000000	2.13460723

```
    -1.33902835    1.35334636    0.00000000    11.85369711
    -1.23319294    1.35334636    0.00000000    12.99487135
    -1.28611065    1.30042866    0.00000000    21.34889057
    -1.28611065    1.40626406    0.00000000    17.60318052
45  2657.0  # 737.7 nm,  (r3v6r2v7) 45
     0.011575      0.022055      0.00000000 1.0   # electr. mom.
     0.00000000    0.00000000   -1.664952771      # magnet. mom.
     1.31876649   -1.08237463    0.00000000   -34.68590159
     0.00000000   -0.70208822    0.00000000    64.29091779
     2.11091906    0.04406596    0.00000000    10.23878251
     1.32524096    1.15754064    0.00000000   -39.00024922
    -0.04178899    0.70146698    0.00000000    68.25769765
    -1.16182154   -1.48163476    0.00000000   -21.23575686
    -2.37713566   -0.81881509    0.00000000     3.32785638
    -2.44031774    0.59060577    0.00000000     3.21863941
    -1.28611065    1.35334636    0.00000000   -38.04544957
     1.26584879   -1.08237463    0.00000000     8.89098471
     1.37168419   -1.08237463    0.00000000     7.63395910
     1.31876649   -1.13529233    0.00000000     8.28740583
     1.31876649   -1.02945693    0.00000000    10.10450179
    -0.05291770   -0.70208822    0.00000000   -15.63703489
     0.05291770   -0.70208822    0.00000000   -16.28372344
     0.00000000   -0.75500592    0.00000000   -16.43601095
     0.00000000   -0.64917052    0.00000000   -16.27862113
     2.05800137    0.04406596    0.00000000    -3.02633386
     2.16383676    0.04406596    0.00000000    -1.52942976
     2.11091906   -0.00885174    0.00000000    -2.68081147
     2.11091906    0.09698366    0.00000000    -3.18625535
     1.27232327    1.15754064    0.00000000    11.56795288
     1.37815866    1.15754064    0.00000000    10.24600865
     1.32524096    1.10462294    0.00000000     9.86782727
     1.32524096    1.21045834    0.00000000     7.62843152
    -0.09470669    0.70146698    0.00000000   -32.68994522
     0.01112871    0.70146698    0.00000000   -33.15521041
    -0.04178899    0.64854929    0.00000000    -0.31170232
    -0.04178899    0.75438469    0.00000000    -2.38581059
    -1.21473924   -1.48163476    0.00000000     6.74805929
    -1.10890384   -1.48163476    0.00000000     7.48712163
    -1.16182154   -1.53455246    0.00000000     3.17246087
    -1.16182154   -1.42871706    0.00000000     3.96232053
    -2.43005336   -0.81881509    0.00000000    -1.07906780
    -2.32421796   -0.81881509    0.00000000    -1.43425344
    -2.37713566   -0.87173279    0.00000000    -0.44080719
    -2.37713566   -0.76589740    0.00000000    -0.40570714
    -2.49323545    0.59060577    0.00000000    -2.53292927
    -2.38740004    0.59060577    0.00000000    -3.34921413
    -2.44031774    0.53768808    0.00000000     1.31814973
    -2.44031774    0.64352347    0.00000000     1.28076434
    -1.33902835    1.35334636    0.00000000     7.11221826
    -1.23319294    1.35334636    0.00000000     7.79692281
    -1.28611065    1.30042866    0.00000000    12.80933434
    -1.28611065    1.40626406    0.00000000    10.56190831
45  2657.0  # 737.7 nm,  (r3v7r2v7) 45
     0.012218      0.02328       0.00000000 1.0   # electr. mom.
     0.00000000    0.00000000   -1.664952771      # magnet. mom.
     1.31876649   -1.08237463    0.00000000   -36.61289612
     0.00000000   -0.70208822    0.00000000    67.86263545
     2.11091906    0.04406596    0.00000000    10.80760376
     1.32524096    1.15754064    0.00000000   -41.16692973
    -0.04178899    0.70146698    0.00000000    72.04979196
    -1.16182154   -1.48163476    0.00000000   -22.41552113
    -2.37713566   -0.81881509    0.00000000     3.51273729
    -2.44031774    0.59060577    0.00000000     3.39745271
    -1.28611065    1.35334636    0.00000000   -40.15908566
     1.26584879   -1.08237463    0.00000000     9.38492831
     1.37168419   -1.08237463    0.00000000     8.05806794
     1.31876649   -1.13529233    0.00000000     8.74781726
     1.31876649   -1.02945693    0.00000000    10.66586300
    -0.05291770   -0.70208822    0.00000000   -16.50575905
     0.05291770   -0.70208822    0.00000000   -17.18837474
     0.00000000   -0.75500592    0.00000000   -17.34912267
     0.00000000   -0.64917052    0.00000000   -17.18298897
     2.05800137    0.04406596    0.00000000    -3.19446352
     2.16383676    0.04406596    0.00000000    -1.61439808
     2.11091906   -0.00885174    0.00000000    -2.82974544
     2.11091906    0.09698366    0.00000000    -3.36326954
     1.27232327    1.15754064    0.00000000    12.21061693
     1.37815866    1.15754064    0.00000000    10.81523135
     1.32524096    1.10462294    0.00000000    10.41603990
     1.32524096    1.21045834    0.00000000     8.05223327
    -0.09470669    0.70146698    0.00000000   -34.50605328
     0.01112871    0.70146698    0.00000000   -34.99716654
    -0.04178899    0.64854929    0.00000000    -0.32901912
    -0.04178899    0.75438469    0.00000000    -2.51835562
    -1.21473924   -1.48163476    0.00000000     7.12295147
    -1.10890384   -1.48163476    0.00000000     7.90307283
    -1.16182154   -1.53455246    0.00000000     3.34870870
```

-1.16182154	-1.42871706	0.00000000	4.18244945
-2.43005336	-0.81881509	0.00000000	-1.13901601
-2.32421796	-0.81881509	0.00000000	-1.51393418
-2.37713566	-0.87173279	0.00000000	-0.46529648
-2.37713566	-0.76589740	0.00000000	-0.42824642
-2.49323545	0.59060577	0.00000000	-2.67364756
-2.38740004	0.59060577	0.00000000	-3.53528159
-2.44031774	0.53768808	0.00000000	1.39138027
-2.44031774	0.64352347	0.00000000	1.35191791
-1.33902835	1.35334636	0.00000000	7.50734150
-1.23319294	1.35334636	0.00000000	8.23008519
-1.28611065	1.30042866	0.00000000	13.52096403
-1.28611065	1.40626406	0.00000000	11.14868099

45 2657.0 # 737.7 nm, (r3v8r2v7) 45

0.005144	0.009802	0.00000000 1.0	# electr. mom.
0.00000000	0.00000000	-1.664952771	# magnet. mom.
1.31876649	-1.08237463	0.00000000	-15.41595626
0.00000000	-0.70208822	0.00000000	28.57374124
2.11091906	0.04406596	0.00000000	4.55057001
1.32524096	1.15754064	0.00000000	-17.33344410
-0.04178899	0.70146698	0.00000000	30.33675451
-1.16182154	-1.48163476	0.00000000	-9.43811416
-2.37713566	-0.81881509	0.00000000	1.47904728
-2.44031774	0.59060577	0.00000000	1.43050640
-1.28611065	1.35334636	0.00000000	-16.90908870
1.26584879	-1.08237463	0.00000000	3.95154876
1.37168419	-1.08237463	0.00000000	3.39287071
1.31876649	-1.13529233	0.00000000	3.68329148
1.31876649	-1.02945693	0.00000000	4.49088968
-0.05291770	-0.70208822	0.00000000	-6.94979329
0.05291770	-0.70208822	0.00000000	-7.23721042
0.00000000	-0.75500592	0.00000000	-7.30489375
0.00000000	-0.64917052	0.00000000	-7.23494272
2.05800137	0.04406596	0.00000000	-1.34503727
2.16383676	0.04406596	0.00000000	-0.67974656
2.11091906	-0.00885174	0.00000000	-1.19147176
2.11091906	0.09698366	0.00000000	-1.41611349
1.27232327	1.15754064	0.00000000	5.14131239
1.37815866	1.15754064	0.00000000	4.55378162
1.32524096	1.10462294	0.00000000	4.38570101
1.32524096	1.21045834	0.00000000	3.39041401
-0.09470669	0.70146698	0.00000000	-14.52886454
0.01112871	0.70146698	0.00000000	-14.73564907
-0.04178899	0.64854929	0.00000000	-0.13853436
-0.04178899	0.75438469	0.00000000	-1.06036026
-1.21473924	-1.48163476	0.00000000	2.99913746
-1.10890384	-1.48163476	0.00000000	3.32760961
-1.16182154	-1.53455246	0.00000000	1.40998261
-1.16182154	-1.42871706	0.00000000	1.76103135
-2.43005336	-0.81881509	0.00000000	-0.47958569
-2.32421796	-0.81881509	0.00000000	-0.63744597
-2.37713566	-0.87173279	0.00000000	-0.19591431
-2.37713566	-0.76589740	0.00000000	-0.18031428
-2.49323545	0.59060577	0.00000000	-1.12574634
-2.38740004	0.59060577	0.00000000	-1.48853962
-2.44031774	0.53768808	0.00000000	0.58584432
-2.44031774	0.64352347	0.00000000	0.56922859
-1.33902835	1.35334636	0.00000000	3.16098589
-1.23319294	1.35334636	0.00000000	3.46529903
-1.28611065	1.30042866	0.00000000	5.69303749
-1.28611065	1.40626406	0.00000000	4.69418147

45 2657.0 # 737.7 nm, (r3v9r2v7) 45

0.00643	0.012253	0.00000000 1.0	# electr. mom.
0.00000000	0.00000000	-1.664952771	# magnet. mom.
1.31876649	-1.08237463	0.00000000	-19.26994533
0.00000000	-0.70208822	0.00000000	35.71717655
2.11091906	0.04406596	0.00000000	5.68821251
1.32524096	1.15754064	0.00000000	-21.66680512
-0.04178899	0.70146698	0.00000000	37.92094314
-1.16182154	-1.48163476	0.00000000	-11.79764270
-2.37713566	-0.81881509	0.00000000	1.84880910
-2.44031774	0.59060577	0.00000000	1.78813300
-1.28611065	1.35334636	0.00000000	-21.13636087
1.26584879	-1.08237463	0.00000000	4.93943595
1.37168419	-1.08237463	0.00000000	4.24108839
1.31876649	-1.13529233	0.00000000	4.60411435
1.31876649	-1.02945693	0.00000000	5.61361210
-0.05291770	-0.70208822	0.00000000	-8.68724161
0.05291770	-0.70208822	0.00000000	-9.04651302
0.00000000	-0.75500592	0.00000000	-9.13111719
0.00000000	-0.64917052	0.00000000	-9.04367841
2.05800137	0.04406596	0.00000000	-1.68129659
2.16383676	0.04406596	0.00000000	-0.84968320
2.11091906	-0.00885174	0.00000000	-1.48933970
2.11091906	0.09698366	0.00000000	-1.77014186
1.27232327	1.15754064	0.00000000	6.42664049
1.37815866	1.15754064	0.00000000	5.69222703

```
    1.32524096     1.10462294     0.00000000     5.48212626
    1.32524096     1.21045834     0.00000000     4.23801751
   -0.09470669     0.70146698     0.00000000   -18.16108068
    0.01112871     0.70146698     0.00000000   -18.41956134
   -0.04178899     0.64854929     0.00000000    -0.17316796
   -0.04178899     0.75438469     0.00000000    -1.32545033
   -1.21473924    -1.48163476     0.00000000     3.74892183
   -1.10890384    -1.48163476     0.00000000     4.15951202
   -1.16182154    -1.53455246     0.00000000     1.76247826
   -1.16182154    -1.42871706     0.00000000     2.20128918
   -2.43005336    -0.81881509     0.00000000    -0.59948211
   -2.32421796    -0.81881509     0.00000000    -0.79680747
   -2.37713566    -0.87173279     0.00000000    -0.24489288
   -2.37713566    -0.76589740     0.00000000    -0.22539285
   -2.49323545     0.59060577     0.00000000    -1.40718293
   -2.38740004     0.59060577     0.00000000    -1.86067452
   -2.44031774     0.53768808     0.00000000     0.73230540
   -2.44031774     0.64352347     0.00000000     0.71153574
   -1.33902835     1.35334636     0.00000000     3.95123237
   -1.23319294     1.35334636     0.00000000     4.33162378
   -1.28611065     1.30042866     0.00000000     7.11629686
   -1.28611065     1.40626406     0.00000000     5.86772684
45    13555.0    # 737.7 nm,    (r7r2v7) 45
    0.088747      -0.02082        0.00000000 1.0     # electr. mom.
    0.00000000     0.00000000    -0.8485163126       # magnet. mom.
    1.31876649    -1.08237463     0.00000000     6.00877238
    0.00000000    -0.70208822     0.00000000     2.90806028
    2.11091906     0.04406596     0.00000000     2.57424192
    1.32524096     1.15754064     0.00000000    -3.72244048
   -0.04178899     0.70146698     0.00000000    32.69385989
   -1.16182154    -1.48163476     0.00000000   -34.34754966
   -2.37713566    -0.81881509     0.00000000    19.47476557
   -2.44031774     0.59060577     0.00000000    -8.46933152
   -1.28611065     1.35334636     0.00000000   -15.25838255
    1.26584879    -1.08237463     0.00000000    -1.84747792
    1.37168419    -1.08237463     0.00000000    -1.31606565
    1.31876649    -1.13529233     0.00000000    -1.12146227
    1.31876649    -1.02945693     0.00000000    -1.82619574
   -0.05291770    -0.70208822     0.00000000    12.70245621
    0.05291770    -0.70208822     0.00000000    13.05702663
    0.00000000    -0.75500592     0.00000000   -13.35486565
    0.00000000    -0.64917052     0.00000000   -15.28623362
    2.05800137     0.04406596     0.00000000    -1.74990351
    2.16383676     0.04406596     0.00000000    -1.69069970
    2.11091906    -0.00885174     0.00000000     0.21649847
    2.11091906     0.09698366     0.00000000     0.69633467
    1.27232327     1.15754064     0.00000000    -4.62481123
    1.37815866     1.15754064     0.00000000    -4.00564019
    1.32524096     1.10462294     0.00000000     6.64707262
    1.32524096     1.21045834     0.00000000     5.69026036
   -0.09470669     0.70146698     0.00000000   -16.73722508
    0.01112871     0.70146698     0.00000000   -17.62429288
   -0.04178899     0.64854929     0.00000000     0.64095985
   -0.04178899     0.75438469     0.00000000     1.19205709
   -1.21473924    -1.48163476     0.00000000    12.08020520
   -1.10890384    -1.48163476     0.00000000    12.52488690
   -1.16182154    -1.53455246     0.00000000     4.85184627
   -1.16182154    -1.42871706     0.00000000     4.81415856
   -2.43005336    -0.81881509     0.00000000    -5.06531125
   -2.32421796    -0.81881509     0.00000000    -5.37827480
   -2.37713566    -0.87173279     0.00000000    -4.48990938
   -2.37713566    -0.76589740     0.00000000    -4.45730120
   -2.49323545     0.59060577     0.00000000     4.35475220
   -2.38740004     0.59060577     0.00000000     5.14957243
   -2.44031774     0.53768808     0.00000000    -1.02587396
   -2.44031774     0.64352347     0.00000000    -0.02752140
   -1.33902835     1.35334636     0.00000000     1.70063622
   -1.23319294     1.35334636     0.00000000     1.57779508
   -1.28611065     1.30042866     0.00000000     6.08378528
   -1.28611065     1.40626406     0.00000000     5.78676554
45    15636.0    # 639.5 nm,    (r8r2v7) 45
   -0.23327        0.033457       0.00000000 1.0     # electr. mom.
    0.00000000     0.00000000    -0.9999114666E-01   # magnet. mom.
    1.31876649    -1.08237463     0.00000000   -12.73829586
    0.00000000    -0.70208822     0.00000000    42.17090235
    2.11091906     0.04406596     0.00000000     5.82354314
    1.32524096     1.15754064     0.00000000    46.57712631
   -0.04178899     0.70146698     0.00000000   -77.63220708
   -1.16182154    -1.48163476     0.00000000    -8.40403867
   -2.37713566    -0.81881509     0.00000000    14.57848406
   -2.44031774     0.59060577     0.00000000   -13.95199740
   -1.28611065     1.35334636     0.00000000    34.35716529
    1.26584879    -1.08237463     0.00000000     1.54234728
    1.37168419    -1.08237463     0.00000000     1.55792705
    1.31876649    -1.13529233     0.00000000     4.73627760
    1.31876649    -1.02945693     0.00000000     4.97032539
   -0.05291770    -0.70208822     0.00000000    -9.95210735
```

```
     0.05291770     -0.70208822      0.00000000      -8.84660881
     0.00000000     -0.75500592      0.00000000     -11.93107344
     0.00000000     -0.64917052      0.00000000     -11.55806871
     2.05800137      0.04406596      0.00000000       1.98496036
     2.16383676      0.04406596      0.00000000       1.89037186
     2.11091906     -0.00885174      0.00000000      -6.19828088
     2.11091906      0.09698366      0.00000000      -3.53554040
     1.27232327      1.15754064      0.00000000     -12.71985549
     1.37815866      1.15754064      0.00000000     -12.24265757
     1.32524096      1.10462294      0.00000000     -11.40167122
     1.32524096      1.21045834      0.00000000     -10.30332155
    -0.09470669      0.70146698      0.00000000      31.11566505
     0.01112871      0.70146698      0.00000000      29.07192381
    -0.04178899      0.64854929      0.00000000       8.18827269
    -0.04178899      0.75438469      0.00000000       9.40162753
    -1.21473924     -1.48163476      0.00000000       0.32623727
    -1.10890384     -1.48163476      0.00000000       0.89988912
    -1.16182154     -1.53455246      0.00000000       2.85709432
    -1.16182154     -1.42871706      0.00000000       4.51853302
    -2.43005336     -0.81881509      0.00000000      -8.91693944
    -2.32421796     -0.81881509      0.00000000     -10.41502400
    -2.37713566     -0.87173279      0.00000000       2.16536285
    -2.37713566     -0.76589740      0.00000000       2.49077652
    -2.49323545      0.59060577      0.00000000       1.53419612
    -2.38740004      0.59060577      0.00000000       3.06167147
    -2.44031774      0.53768808      0.00000000       5.06398121
    -2.44031774      0.64352347      0.00000000       4.47970057
    -1.33902835      1.35334636      0.00000000      -9.10611941
    -1.23319294      1.35334636      0.00000000      -9.20099970
    -1.28611065      1.30042866      0.00000000      -9.23090648
    -1.28611065      1.40626406      0.00000000      -7.07864879
&TRANSITION 9->...
  0     0.0    # (r3v2r3v1) 45
     0.00000000      0.00000000      0.00000000 1.0        # electr. mom.
     0.00000000      0.00000000      0.00000000            # magnet. mom.
  0     0.0    # (r3v3r3v1) 45
     0.00000000      0.00000000      0.00000000 1.0        # electr. mom.
     0.00000000      0.00000000      0.00000000            # magnet. mom.
  0     0.0    # (r3v4r3v1) 45
     0.00000000      0.00000000      0.00000000 1.0        # electr. mom.
     0.00000000      0.00000000      0.00000000            # magnet. mom.
  0     0.0    # (r3v5r3v1) 45
     0.00000000      0.00000000      0.00000000 1.0        # electr. mom.
     0.00000000      0.00000000      0.00000000            # magnet. mom.
  0     0.0    # (r3v6r3v1) 45
     0.00000000      0.00000000      0.00000000 1.0        # electr. mom.
     0.00000000      0.00000000      0.00000000            # magnet. mom.
  0     0.0    # (r3v7r3v1) 45
     0.00000000      0.00000000      0.00000000 1.0        # electr. mom.
     0.00000000      0.00000000      0.00000000            # magnet. mom.
  0     0.0    # (r3v8r3v1) 45
     0.00000000      0.00000000      0.00000000 1.0        # electr. mom.
     0.00000000      0.00000000      0.00000000            # magnet. mom.
  0     0.0    # (r3v9r3v1) 45
     0.00000000      0.00000000      0.00000000 1.0        # electr. mom.
     0.00000000      0.00000000      0.00000000            # magnet. mom.
 45   10898.0    # 917.6 nm,    (r7r3v1) 45
    -0.30567         0.469249        0.00000000 1.0     # electr. mom.
     0.00000000      0.00000000     -0.106              # magnet. mom.
     1.31876649     -1.08237463      0.00000000    -309.83504265
     0.00000000     -0.70208822      0.00000000       3.36956239
     2.11091906      0.04406596      0.00000000     -78.17595123
     1.32524096      1.15754064      0.00000000     -93.73779597
    -0.04178899      0.70146698      0.00000000     156.95133569
    -1.16182154     -1.48163476      0.00000000      28.70693291
    -2.37713566     -0.81881509      0.00000000     -79.34231383
    -2.44031774      0.59060577      0.00000000     -31.73946188
    -1.28611065      1.35334636      0.00000000     -30.22470781
     1.26584879     -1.08237463      0.00000000      45.56542874
     1.37168419     -1.08237463      0.00000000      54.14291145
     1.31876649     -1.13529233      0.00000000      94.80939633
     1.31876649     -1.02945693      0.00000000     116.15476723
    -0.05291770     -0.70208822      0.00000000     -37.95425132
     0.05291770     -0.70208822      0.00000000     -41.64909664
     0.00000000     -0.75500592      0.00000000      41.03735117
     0.00000000     -0.64917052      0.00000000      35.59088084
     2.05800137      0.04406596      0.00000000     -22.90351502
     2.16383676      0.04406596      0.00000000     -12.82945601
     2.11091906     -0.00885174      0.00000000      55.97935817
     2.11091906      0.09698366      0.00000000      56.56073965
     1.27232327      1.15754064      0.00000000      -9.44400135
     1.37815866      1.15754064      0.00000000      -8.52204618
     1.32524096      1.10462294      0.00000000      62.28135174
     1.32524096      1.21045834      0.00000000      50.39204535
    -0.09470669      0.70146698      0.00000000     -75.18249760
     0.01112871      0.70146698      0.00000000     -84.37998975
    -0.04178899      0.64854929      0.00000000      -2.40065707
```

-0.04178899 0.75438469 0.00000000 4.40338270
-1.21473924 -1.48163476 0.00000000 -11.20328248
-1.10890384 -1.48163476 0.00000000 -11.95861247
-1.16182154 -1.53455246 0.00000000 -0.68935277
-1.16182154 -1.42871706 0.00000000 -5.55139221
-2.43005336 -0.81881509 0.00000000 10.22432741
-2.32421796 -0.81881509 0.00000000 14.41327878
-2.37713566 -0.87173279 0.00000000 26.09248622
-2.37713566 -0.76589740 0.00000000 29.17340135
-2.49323545 0.59060577 0.00000000 8.18328117
-2.38740004 0.59060577 0.00000000 8.79049090
-2.44031774 0.53768808 0.00000000 6.66651013
-2.44031774 0.64352347 0.00000000 8.05109940
-1.33902835 1.35334636 0.00000000 -15.55516709
-1.23319294 1.35334636 0.00000000 -16.37197919
-1.28611065 1.30042866 0.00000000 31.54149515
-1.28611065 1.40626406 0.00000000 30.56875569
45 12979.0 # 770.5 nm, (r8r3v1) 45
-0.17628 -0.23826 0.00000000 1.0 # electr. mom.
0.00000000 0.00000000 -0.209857 # magnet. mom.
1.31876649 -1.08237463 0.00000000 -5.52211934
0.00000000 -0.70208822 0.00000000 175.67078322
2.11091906 0.04406596 0.00000000 99.41567420
1.32524096 1.15754064 0.00000000 -38.98036973
-0.04178899 0.70146698 0.00000000 -110.19881931
-1.16182154 -1.48163476 0.00000000 -98.30021509
-2.37713566 -0.81881509 0.00000000 64.14240763
-2.44031774 0.59060577 0.00000000 6.77913533
-1.28611065 1.35334636 0.00000000 42.30961672
1.26584879 -1.08237463 0.00000000 10.45141948
1.37168419 -1.08237463 0.00000000 8.50482076
1.31876649 -1.13529233 0.00000000 -7.37110884
1.31876649 -1.02945693 0.00000000 -5.70044410
-0.05291770 -0.70208822 0.00000000 -67.32474691
0.05291770 -0.70208822 0.00000000 -64.78009246
0.00000000 -0.75500592 0.00000000 -21.98434654
0.00000000 -0.64917052 0.00000000 -23.07801920
2.05800137 0.04406596 0.00000000 -24.39221938
2.16383676 0.04406596 0.00000000 -22.45140415
2.11091906 -0.00885174 0.00000000 -26.28109066
2.11091906 0.09698366 0.00000000 -26.32088255
1.27232327 1.15754064 0.00000000 29.51904477
1.37815866 1.15754064 0.00000000 26.81167537
1.32524096 1.10462294 0.00000000 -10.15074361
1.32524096 1.21045834 0.00000000 -7.34233896
-0.09470669 0.70146698 0.00000000 42.34978071
0.01112871 0.70146698 0.00000000 44.67167551
-0.04178899 0.64854929 0.00000000 16.15339363
-0.04178899 0.75438469 0.00000000 7.69111682
-1.21473924 -1.48163476 0.00000000 17.50422712
-1.10890384 -1.48163476 0.00000000 17.85646315
-1.16182154 -1.53455246 0.00000000 25.89355385
-1.16182154 -1.42871706 0.00000000 38.40280137
-2.43005336 -0.81881509 0.00000000 -39.68108571
-2.32421796 -0.81881509 0.00000000 -48.67585854
-2.37713566 -0.87173279 0.00000000 8.91278539
-2.37713566 -0.76589740 0.00000000 15.08908364
-2.49323545 0.59060577 0.00000000 19.50365682
-2.38740004 0.59060577 0.00000000 22.92016254
-2.44031774 0.53768808 0.00000000 -26.54089520
-2.44031774 0.64352347 0.00000000 -22.97846604
-1.33902835 1.35334636 0.00000000 1.01701254
-1.23319294 1.35334636 0.00000000 -1.91354286
-1.28611065 1.30042866 0.00000000 -22.44244031
-1.28611065 1.40626406 0.00000000 -19.15904107
&TRANSITION 10->...
0 0.0 # (r3v3r3v2) 45
0.00000000 0.00000000 0.00000000 1.0 # electr. mom.
0.00000000 0.00000000 0.00000000 # magnet. mom.
0 0.0 # (r3v4r3v2) 45
0.00000000 0.00000000 0.00000000 1.0 # electr. mom.
0.00000000 0.00000000 0.00000000 # magnet. mom.
0 0.0 # (r3v5r3v2) 45
0.00000000 0.00000000 0.00000000 1.0 # electr. mom.
0.00000000 0.00000000 0.00000000 # magnet. mom.
0 0.0 # (r3v6r3v2) 45
0.00000000 0.00000000 0.00000000 1.0 # electr. mom.
0.00000000 0.00000000 0.00000000 # magnet. mom.
0 0.0 # (r3v7r3v2) 45
0.00000000 0.00000000 0.00000000 1.0 # electr. mom.
0.00000000 0.00000000 0.00000000 # magnet. mom.
0 0.0 # (r3v8r3v2) 45
0.00000000 0.00000000 0.00000000 1.0 # electr. mom.
0.00000000 0.00000000 0.00000000 # magnet. mom.
0 0.0 # (r3v9r3v2) 45
0.00000000 0.00000000 0.00000000 1.0 # electr. mom.
0.00000000 0.00000000 0.00000000 # magnet. mom.

45 10898.0 # 917.6 nm, (r7r3v2) 45
-0.28422 0.436319 0.00000000 1.0 # electr. mom.
0.00000000 0.00000000 -0.0976 # magnet. mom.
1.31876649 -1.08237463 0.00000000 -288.09223264
0.00000000 -0.70208822 0.00000000 3.13310187
2.11091906 0.04406596 0.00000000 -72.68991956
1.32524096 1.15754064 0.00000000 -87.15970502
-0.04178899 0.70146698 0.00000000 145.93720687
-1.16182154 -1.48163476 0.00000000 26.69241130
-2.37713566 -0.81881509 0.00000000 -73.77443216
-2.44031774 0.59060577 0.00000000 -29.51213122
-1.28611065 1.35334636 0.00000000 -28.10367569
1.26584879 -1.08237463 0.00000000 42.36785479
1.37168419 -1.08237463 0.00000000 50.34340889
1.31876649 -1.13529233 0.00000000 88.15610536
1.31876649 -1.02945693 0.00000000 108.00355550
-0.05291770 -0.70208822 0.00000000 -35.29079508
0.05291770 -0.70208822 0.00000000 -38.72635301
0.00000000 -0.75500592 0.00000000 38.15753705
0.00000000 -0.64917052 0.00000000 33.09327516
2.05800137 0.04406596 0.00000000 -21.29625081
2.16383676 0.04406596 0.00000000 -11.92914331
2.11091906 -0.00885174 0.00000000 52.05098216
2.11091906 0.09698366 0.00000000 52.59156494
1.27232327 1.15754064 0.00000000 -8.78126441
1.37815866 1.15754064 0.00000000 -7.92400785
1.32524096 1.10462294 0.00000000 57.91073056
1.32524096 1.21045834 0.00000000 46.85576146
-0.09470669 0.70146698 0.00000000 -69.90653285
0.01112871 0.70146698 0.00000000 -78.45858696
-0.04178899 0.64854929 0.00000000 -2.23218991
-0.04178899 0.75438469 0.00000000 4.09437338
-1.21473924 -1.48163476 0.00000000 -10.41708722
-1.10890384 -1.48163476 0.00000000 -11.11941159
-1.16182154 -1.53455246 0.00000000 -0.64097713
-1.16182154 -1.42871706 0.00000000 -5.16182083
-2.43005336 -0.81881509 0.00000000 9.50683075
-2.32421796 -0.81881509 0.00000000 13.40182062
-2.37713566 -0.87173279 0.00000000 24.26143456
-2.37713566 -0.76589740 0.00000000 27.12614511
-2.49323545 0.59060577 0.00000000 7.60901582
-2.38740004 0.59060577 0.00000000 8.17361435
-2.44031774 0.53768808 0.00000000 6.19868486
-2.44031774 0.64352347 0.00000000 7.48610997
-1.33902835 1.35334636 0.00000000 -14.46357642
-1.23319294 1.35334636 0.00000000 -15.22306837
-1.28611065 1.30042866 0.00000000 29.32805689
-1.28611065 1.40626406 0.00000000 28.42357985
45 12979.0 # 770.5 nm, (r8r3v2) 45
-0.16391 -0.22154 0.00000000 1.0 # electr. mom.
0.00000000 0.00000000 -0.1937 # magnet. mom.
1.31876649 -1.08237463 0.00000000 -5.13460219
0.00000000 -0.70208822 0.00000000 163.34300896
2.11091906 0.04406596 0.00000000 92.43913566
1.32524096 1.15754064 0.00000000 -36.24490519
-0.04178899 0.70146698 0.00000000 -102.46556883
-1.16182154 -1.48163476 0.00000000 -91.40195439
-2.37713566 -0.81881509 0.00000000 59.64118605
-2.44031774 0.59060577 0.00000000 6.30340653
-1.28611065 1.35334636 0.00000000 39.34052081
1.26584879 -1.08237463 0.00000000 9.71798653
1.37168419 -1.08237463 0.00000000 7.90799124
1.31876649 -1.13529233 0.00000000 -6.85383805
1.31876649 -1.02945693 0.00000000 -5.30041293
-0.05291770 -0.70208822 0.00000000 -62.60020327
0.05291770 -0.70208822 0.00000000 -60.23412106
0.00000000 -0.75500592 0.00000000 -20.44158538
0.00000000 -0.64917052 0.00000000 -21.45850908
2.05800137 0.04406596 0.00000000 -22.68048468
2.16383676 0.04406596 0.00000000 -20.87586702
2.11091906 -0.00885174 0.00000000 -24.43680359
2.11091906 0.09698366 0.00000000 -24.47380307
1.27232327 1.15754064 0.00000000 27.44753286
1.37815866 1.15754064 0.00000000 24.93015429
1.32524096 1.10462294 0.00000000 -9.43841073
1.32524096 1.21045834 0.00000000 -6.82708710
-0.09470669 0.70146698 0.00000000 39.37786628
0.01112871 0.70146698 0.00000000 41.53682109
-0.04178899 0.64854929 0.00000000 15.01982215
-0.04178899 0.75438469 0.00000000 7.15138933
-1.21473924 -1.48163476 0.00000000 16.27586030
-1.10890384 -1.48163476 0.00000000 16.60337802
-1.16182154 -1.53455246 0.00000000 24.07646235
-1.16182154 -1.42871706 0.00000000 35.70786794
-2.43005336 -0.81881509 0.00000000 -36.89644812
-2.32421796 -0.81881509 0.00000000 -45.26000882
-2.37713566 -0.87173279 0.00000000 8.28732677

```
-2.37713566  -0.76589740  0.00000000  14.03020058
-2.49323545   0.59060577  0.00000000  18.13497915
-2.38740004   0.59060577  0.00000000  21.31173008
-2.44031774   0.53768808  0.00000000 -24.67837624
-2.44031774   0.64352347  0.00000000 -21.36594211
-1.33902835   1.35334636  0.00000000   0.94564324
-1.23319294   1.35334636  0.00000000  -1.77925915
-1.28611065   1.30042866  0.00000000 -20.86753221
-1.28611065   1.40626406  0.00000000 -17.81454696
&TRANSITION 11->...
 0   0.0   # (r3v4r3v3) 45
  0.00000000  0.00000000  0.00000000 1.0   # electr. mom.
  0.00000000  0.00000000  0.00000000       # magnet. mom.
 0   0.0   # (r3v5r3v3) 45
  0.00000000  0.00000000  0.00000000 1.0   # electr. mom.
  0.00000000  0.00000000  0.00000000       # magnet. mom.
 0   0.0   # (r3v6r3v3) 45
  0.00000000  0.00000000  0.00000000 1.0   # electr. mom.
  0.00000000  0.00000000  0.00000000       # magnet. mom.
 0   0.0   # (r3v7r3v3) 45
  0.00000000  0.00000000  0.00000000 1.0   # electr. mom.
  0.00000000  0.00000000  0.00000000       # magnet. mom.
 0   0.0   # (r3v8r3v3) 45
  0.00000000  0.00000000  0.00000000 1.0   # electr. mom.
  0.00000000  0.00000000  0.00000000       # magnet. mom.
 0   0.0   # (r3v9r3v3) 45
  0.00000000  0.00000000  0.00000000 1.0   # electr. mom.
  0.00000000  0.00000000  0.00000000       # magnet. mom.
 45  10898.0   # 917.6 nm,   (r7r3v3) 45
-0.17161      0.263438    0.00000000 1.0   # electr. mom.
  0.00000000  0.00000000  0.0610           # magnet. mom.
  1.31876649  -1.08237463  0.00000000 -173.94248009
  0.00000000  -0.70208822  0.00000000    1.89168415
  2.11091906   0.04406596  0.00000000  -43.88825332
  1.32524096   1.15754064  0.00000000  -52.62472756
-0.04178899   0.70146698  0.00000000   88.11303056
-1.16182154  -1.48163476  0.00000000   16.11617286
-2.37713566  -0.81881509  0.00000000  -44.54305338
-2.44031774   0.59060577  0.00000000  -17.81864526
-1.28611065   1.35334636  0.00000000  -16.96825702
  1.26584879  -1.08237463  0.00000000   25.58059157
  1.37168419  -1.08237463  0.00000000   30.39602046
  1.31876649  -1.13529233  0.00000000   53.22632777
  1.31876649  -1.02945693  0.00000000   65.20969388
-0.05291770  -0.70208822  0.00000000  -21.30764986
  0.05291770  -0.70208822  0.00000000  -23.38194899
  0.00000000  -0.75500592  0.00000000   23.03851294
  0.00000000  -0.64917052  0.00000000   19.98084538
  2.05800137   0.04406596  0.00000000  -12.85811370
  2.16383676   0.04406596  0.00000000   -7.20250162
  2.11091906  -0.00885174  0.00000000   31.42700810
  2.11091906   0.09698366  0.00000000   31.75339770
  1.27232327   1.15754064  0.00000000   -5.30189549
  1.37815866   1.15754064  0.00000000   -4.78430663
  1.32524096   1.10462294  0.00000000   34.96496940
  1.32524096   1.21045834  0.00000000   28.29027107
-0.09470669   0.70146698  0.00000000  -42.20771795
  0.01112871   0.70146698  0.00000000  -47.37122232
-0.04178899   0.64854929  0.00000000   -1.34773730
-0.04178899   0.75438469  0.00000000    2.47207450
-1.21473924  -1.48163476  0.00000000   -6.28956210
-1.10890384  -1.48163476  0.00000000   -6.71360700
-1.16182154  -1.53455246  0.00000000   -0.38700506
-1.16182154  -1.42871706  0.00000000   -3.11657107
-2.43005336  -0.81881509  0.00000000    5.73997328
-2.32421796  -0.81881509  0.00000000    8.09166528
-2.37713566  -0.87173279  0.00000000   14.64841332
-2.37713566  -0.76589740  0.00000000   16.37804988
-2.49323545   0.59060577  0.00000000    4.59412276
-2.38740004   0.59060577  0.00000000    4.93501244
-2.44031774   0.53768808  0.00000000    3.74260218
-2.44031774   0.64352347  0.00000000    4.51991545
-1.33902835   1.35334636  0.00000000   -8.73272539
-1.23319294   1.35334636  0.00000000   -9.19128656
-1.28611065   1.30042866  0.00000000   17.70750605
-1.28611065   1.40626406  0.00000000   17.16140670
 45  12979.0   # 770.5 nm,   (r8r3v3) 45
-0.09896     -0.13376     0.00000000 1.0   # electr. mom.
  0.00000000  0.00000000  0.1211           # magnet. mom.
  1.31876649  -1.08237463  0.00000000   -3.10013717
  0.00000000  -0.70208822  0.00000000   98.62219409
  2.11091906   0.04406596  0.00000000   55.81230832
  1.32524096   1.15754064  0.00000000  -21.88371634
-0.04178899   0.70146698  0.00000000  -61.86600382
-1.16182154  -1.48163476  0.00000000  -55.18608567
-2.37713566  -0.81881509  0.00000000   36.00977271
-2.44031774   0.59060577  0.00000000    3.80583036
```

-1.28611065 1.35334636 0.00000000 23.75276728
1.26584879 -1.08237463 0.00000000 5.86746357
1.37168419 -1.08237463 0.00000000 4.77463622
1.31876649 -1.13529233 0.00000000 -4.13816637
1.31876649 -1.02945693 0.00000000 -3.20024932
-0.05291770 -0.70208822 0.00000000 -37.79634914
0.05291770 -0.70208822 0.00000000 -36.36777121
0.00000000 -0.75500592 0.00000000 -12.34208929
0.00000000 -0.64917052 0.00000000 -12.95608096
2.05800137 0.04406596 0.00000000 -13.69387755
2.16383676 0.04406596 0.00000000 -12.60429707
2.11091906 -0.00885174 0.00000000 -14.75429651
2.11091906 0.09698366 0.00000000 -14.77663582
1.27232327 1.15754064 0.00000000 16.57209531
1.37815866 1.15754064 0.00000000 15.05216863
1.32524096 1.10462294 0.00000000 -5.69866308
1.32524096 1.21045834 0.00000000 -4.12201485
-0.09470669 0.70146698 0.00000000 23.77531549
0.01112871 0.70146698 0.00000000 25.07883537
-0.04178899 0.64854929 0.00000000 9.06857186
-0.04178899 0.75438469 0.00000000 4.31781997
-1.21473924 -1.48163476 0.00000000 9.82693452
-1.10890384 -1.48163476 0.00000000 10.02468107
-1.16182154 -1.53455246 0.00000000 14.53673199
-1.16182154 -1.42871706 0.00000000 21.55946743
-2.43005336 -0.81881509 0.00000000 -22.27710075
-2.32421796 -0.81881509 0.00000000 -27.32679778
-2.37713566 -0.87173279 0.00000000 5.00366899
-2.37713566 -0.76589740 0.00000000 8.47106450
-2.49323545 0.59060577 0.00000000 10.94942137
-2.38740004 0.59060577 0.00000000 12.86745967
-2.44031774 0.53768808 0.00000000 -14.90015169
-2.44031774 0.64352347 0.00000000 -12.90019146
-1.33902835 1.35334636 0.00000000 0.57095441
-1.23319294 1.35334636 0.00000000 -1.07426968
-1.28611065 1.30042866 0.00000000 -12.59926473
-1.28611065 1.40626406 0.00000000 -10.75595288
&TRANSITION 12->...
0 0.0 # (r3v5r3v4) 45
0.00000000 0.00000000 0.00000000 1.0 # electr. mom.
0.00000000 0.00000000 0.00000000 # magnet. mom.
0 0.0 # (r3v6r3v4) 45
0.00000000 0.00000000 0.00000000 1.0 # electr. mom.
0.00000000 0.00000000 0.00000000 # magnet. mom.
0 0.0 # (r3v7r3v4) 45
0.00000000 0.00000000 0.00000000 1.0 # electr. mom.
0.00000000 0.00000000 0.00000000 # magnet. mom.
0 0.0 # (r3v8r3v4) 45
0.00000000 0.00000000 0.00000000 1.0 # electr. mom.
0.00000000 0.00000000 0.00000000 # magnet. mom.
0 0.0 # (r3v9r3v4) 45
0.00000000 0.00000000 0.00000000 1.0 # electr. mom.
0.00000000 0.00000000 0.00000000 # magnet. mom.
45 10898.0 # 917.6 nm, (r7r3v4) 45
-0.18233 0.279903 0.00000000 1.0 # electr. mom.
0.00000000 0.00000000 0.0813 # magnet. mom.
1.31876649 -1.08237463 0.00000000 -184.81388509
0.00000000 -0.70208822 0.00000000 2.00991441
2.11091906 0.04406596 0.00000000 -46.63126915
1.32524096 1.15754064 0.00000000 -55.91377303
-0.04178899 0.70146698 0.00000000 93.62009497
-1.16182154 -1.48163476 0.00000000 17.12343366
-2.37713566 -0.81881509 0.00000000 -47.32699422
-2.44031774 0.59060577 0.00000000 -18.93231059
-1.28611065 1.35334636 0.00000000 -18.02877308
1.26584879 -1.08237463 0.00000000 27.17937854
1.37168419 -1.08237463 0.00000000 32.29577174
1.31876649 -1.13529233 0.00000000 56.55297325
1.31876649 -1.02945693 0.00000000 69.28529975
-0.05291770 -0.70208822 0.00000000 -22.63937798
0.05291770 -0.70208822 0.00000000 -24.84332080
0.00000000 -0.75500592 0.00000000 24.47842000
0.00000000 -0.64917052 0.00000000 21.22964822
2.05800137 0.04406596 0.00000000 -13.66174580
2.16383676 0.04406596 0.00000000 -7.65265797
2.11091906 -0.00885174 0.00000000 33.39119610
2.11091906 0.09698366 0.00000000 33.73798506
1.27232327 1.15754064 0.00000000 -5.63326396
1.37815866 1.15754064 0.00000000 -5.08332579
1.32524096 1.10462294 0.00000000 37.15027998
1.32524096 1.21045834 0.00000000 30.05841301
-0.09470669 0.70146698 0.00000000 -44.84570032
0.01112871 0.70146698 0.00000000 -50.33192371
-0.04178899 0.64854929 0.00000000 -1.43197088
-0.04178899 0.75438469 0.00000000 2.62657915
-1.21473924 -1.48163476 0.00000000 -6.68265973
-1.10890384 -1.48163476 0.00000000 -7.13320744

```
     -1.16182154      -1.53455246       0.00000000        -0.41119288
     -1.16182154      -1.42871706       0.00000000        -3.31135676
     -2.43005336      -0.81881509       0.00000000         6.09872161
     -2.32421796      -0.81881509       0.00000000         8.59739436
     -2.37713566      -0.87173279       0.00000000        15.56393915
     -2.37713566      -0.76589740       0.00000000        17.40167800
     -2.49323545       0.59060577       0.00000000         4.88125543
     -2.38740004       0.59060577       0.00000000         5.24345071
     -2.44031774       0.53768808       0.00000000         3.97651482
     -2.44031774       0.64352347       0.00000000         4.80241017
     -1.33902835       1.35334636       0.00000000        -9.27852072
     -1.23319294       1.35334636       0.00000000        -9.76574197
     -1.28611065       1.30042866       0.00000000        18.81422518
     -1.28611065       1.40626406       0.00000000        18.23399462
 45    12979.0    # 770.5 nm,    (r8r3v4) 45
     -0.10515         -0.14212          0.00000000 1.0    # electr. mom.
      0.00000000       0.00000000       0.1614            # magnet. mom.
      1.31876649      -1.08237463       0.00000000        -3.29389574
      0.00000000      -0.70208822       0.00000000       104.78608122
      2.11091906       0.04406596       0.00000000        59.30057759
      1.32524096       1.15754064       0.00000000       -23.25144861
     -0.04178899       0.70146698       0.00000000       -65.73262906
     -1.16182154      -1.48163476       0.00000000       -58.63521602
     -2.37713566      -0.81881509       0.00000000        38.26038350
     -2.44031774       0.59060577       0.00000000         4.04369476
     -1.28611065       1.35334636       0.00000000        25.23731524
      1.26584879      -1.08237463       0.00000000         6.23418004
      1.37168419      -1.08237463       0.00000000         5.07305098
      1.31876649      -1.13529233       0.00000000        -4.39680177
      1.31876649      -1.02945693       0.00000000        -3.40026490
     -0.05291770      -0.70208822       0.00000000       -40.15862096
      0.05291770      -0.70208822       0.00000000       -38.64075691
      0.00000000      -0.75500592       0.00000000       -13.11346987
      0.00000000      -0.64917052       0.00000000       -13.76583602
      2.05800137       0.04406596       0.00000000       -14.54974489
      2.16383676       0.04406596       0.00000000       -13.39206564
      2.11091906      -0.00885174       0.00000000       -15.67644004
      2.11091906       0.09698366       0.00000000       -15.70017556
      1.27232327       1.15754064       0.00000000        17.60785127
      1.37815866       1.15754064       0.00000000        15.99292917
      1.32524096       1.10462294       0.00000000        -6.05482952
      1.32524096       1.21045834       0.00000000        -4.37964078
     -0.09470669       0.70146698       0.00000000        25.26127271
      0.01112871       0.70146698       0.00000000        26.64626258
     -0.04178899       0.64854929       0.00000000         9.63535760
     -0.04178899       0.75438469       0.00000000         4.58768372
     -1.21473924      -1.48163476       0.00000000        10.44111793
     -1.10890384      -1.48163476       0.00000000        10.65122364
     -1.16182154      -1.53455246       0.00000000        15.44527774
     -1.16182154      -1.42871706       0.00000000        22.90693415
     -2.43005336      -0.81881509       0.00000000       -23.66941955
     -2.32421796      -0.81881509       0.00000000       -29.03472264
     -2.37713566      -0.87173279       0.00000000         5.31639830
     -2.37713566      -0.76589740       0.00000000         9.00050603
     -2.49323545       0.59060577       0.00000000        11.63376021
     -2.38740004       0.59060577       0.00000000        13.67167590
     -2.44031774       0.53768808       0.00000000       -15.83141117
     -2.44031774       0.64352347       0.00000000       -13.70645343
     -1.33902835       1.35334636       0.00000000         0.60663906
     -1.23319294       1.35334636       0.00000000        -1.14141153
     -1.28611065       1.30042866       0.00000000       -13.38671878
     -1.28611065       1.40626406       0.00000000       -11.42819994
&TRANSITION 13->...
 0     0.0    # (r3v6r3v5) 45
      0.00000000       0.00000000       0.00000000 1.0    # electr. mom.
      0.00000000       0.00000000       0.00000000        # magnet. mom.
 0     0.0    # (r3v7r3v5) 45
      0.00000000       0.00000000       0.00000000 1.0    # electr. mom.
      0.00000000       0.00000000       0.00000000        # magnet. mom.
 0     0.0    # (r3v8r3v5) 45
      0.00000000       0.00000000       0.00000000 1.0    # electr. mom.
      0.00000000       0.00000000       0.00000000        # magnet. mom.
 0     0.0    # (r3v9r3v5) 45
      0.00000000       0.00000000       0.00000000 1.0    # electr. mom.
      0.00000000       0.00000000       0.00000000        # magnet. mom.
 45    10898.0    # 917.6 nm,    (r7r3v5) 45
     -0.16088          0.246973         0.00000000 1.0    # electr. mom.
      0.00000000       0.00000000       0.0813            # magnet. mom.
      1.31876649      -1.08237463       0.00000000      -163.07107508
      0.00000000      -0.70208822       0.00000000         1.77345389
      2.11091906       0.04406596       0.00000000       -41.14523749
      1.32524096       1.15754064       0.00000000       -49.33568209
     -0.04178899       0.70146698       0.00000000        82.60596615
     -1.16182154      -1.48163476       0.00000000        15.10891206
     -2.37713566      -0.81881509       0.00000000       -41.75911254
     -2.44031774       0.59060577       0.00000000       -16.70497994
     -1.28611065       1.35334636       0.00000000       -15.90774095
```

```
1.26584879 -1.08237463 0.00000000 23.98180460
1.37168419 -1.08237463 0.00000000 28.49626918
1.31876649 -1.13529233 0.00000000 49.89968228
1.31876649 -1.02945693 0.00000000 61.13408802
-0.05291770 -0.70208822 0.00000000 -19.97592175
0.05291770 -0.70208822 0.00000000 -21.92057718
0.00000000 -0.75500592 0.00000000 21.59860588
0.00000000 -0.64917052 0.00000000 18.73204255
2.05800137 0.04406596 0.00000000 -12.05448159
2.16383676 0.04406596 0.00000000 -6.75234527
2.11091906 -0.00885174 0.00000000 29.46282009
2.11091906 0.09698366 0.00000000 29.76881034
1.27232327 1.15754064 0.00000000 -4.97052703
1.37815866 1.15754064 0.00000000 -4.48528746
1.32524096 1.10462294 0.00000000 32.77965881
1.32524096 1.21045834 0.00000000 26.52212913
-0.09470669 0.70146698 0.00000000 -39.56973558
0.01112871 0.70146698 0.00000000 -44.41052092
-0.04178899 0.64854929 0.00000000 -1.26350372
-0.04178899 0.75438469 0.00000000 2.31756984
-1.21473924 -1.48163476 0.00000000 -5.89646447
-1.10890384 -1.48163476 0.00000000 -6.29400656
-1.16182154 -1.53455246 0.00000000 -0.36281725
-1.16182154 -1.42871706 0.00000000 -2.92178537
-2.43005336 -0.81881509 0.00000000 5.38122495
-2.32421796 -0.81881509 0.00000000 7.58593620
-2.37713566 -0.87173279 0.00000000 13.73288749
-2.37713566 -0.76589740 0.00000000 15.35442176
-2.49323545 0.59060577 0.00000000 4.30699009
-2.38740004 0.59060577 0.00000000 4.62657416
-2.44031774 0.53768808 0.00000000 3.50868954
-2.44031774 0.64352347 0.00000000 4.23742074
-1.33902835 1.35334636 0.00000000 -8.18693005
-1.23319294 1.35334636 0.00000000 -8.61683115
-1.28611065 1.30042866 0.00000000 16.60078692
-1.28611065 1.40626406 0.00000000 16.08881878
45 12979.0 # 770.5 nm, (r8r3v5) 45
-0.09278 -0.1254 0.00000000 1.0 # electr. mom.
0.00000000 0.00000000 0.1614 # magnet. mom.
1.31876649 -1.08237463 0.00000000 -2.90637860
0.00000000 -0.70208822 0.00000000 92.45830696
2.11091906 0.04406596 0.00000000 52.32403905
1.32524096 1.15754064 0.00000000 -20.51598407
-0.04178899 0.70146698 0.00000000 -57.99937859
-1.16182154 -1.48163476 0.00000000 -51.73695531
-2.37713566 -0.81881509 0.00000000 33.75916191
-2.44031774 0.59060577 0.00000000 3.56796596
-1.28611065 1.35334636 0.00000000 22.26821933
1.26584879 -1.08237463 0.00000000 5.50074710
1.37168419 -1.08237463 0.00000000 4.47622145
1.31876649 -1.13529233 0.00000000 -3.87953097
1.31876649 -1.02945693 0.00000000 -3.00023374
-0.05291770 -0.70208822 0.00000000 -35.43407732
0.05291770 -0.70208822 0.00000000 -34.09478551
0.00000000 -0.75500592 0.00000000 -11.57070871
0.00000000 -0.64917052 0.00000000 -12.14632590
2.05800137 0.04406596 0.00000000 -12.83801020
2.16383676 0.04406596 0.00000000 -11.81652850
2.11091906 -0.00885174 0.00000000 -13.83215298
2.11091906 0.09698366 0.00000000 -13.85309608
1.27232327 1.15754064 0.00000000 15.53633935
1.37815866 1.15754064 0.00000000 14.11140809
1.32524096 1.10462294 0.00000000 -5.34249664
1.32524096 1.21045834 0.00000000 -3.86438893
-0.09470669 0.70146698 0.00000000 22.28935827
0.01112871 0.70146698 0.00000000 23.51140816
-0.04178899 0.64854929 0.00000000 8.50178612
-0.04178899 0.75438469 0.00000000 4.04795622
-1.21473924 -1.48163476 0.00000000 9.21275111
-1.10890384 -1.48163476 0.00000000 9.39813850
-1.16182154 -1.53455246 0.00000000 13.62818624
-1.16182154 -1.42871706 0.00000000 20.21200072
-2.43005336 -0.81881509 0.00000000 -20.88478195
-2.32421796 -0.81881509 0.00000000 -25.61887292
-2.37713566 -0.87173279 0.00000000 4.69093968
-2.37713566 -0.76589740 0.00000000 7.94162297
-2.49323545 0.59060577 0.00000000 10.26508254
-2.38740004 0.59060577 0.00000000 12.06324344
-2.44031774 0.53768808 0.00000000 -13.96889221
-2.44031774 0.64352347 0.00000000 -12.09392950
-1.33902835 1.35334636 0.00000000 0.53526976
-1.23319294 1.35334636 0.00000000 -1.00712782
-1.28611065 1.30042866 0.00000000 -11.81181069
-1.28611065 1.40626406 0.00000000 -10.08370583
&TRANSITION 14->...
0 0.0 # (r3v7r3v6) 45
0.00000000 0.00000000 0.00000000 1.0 # electr. mom.
```

0.00000000 0.00000000 0.00000000 # magnet. mom.
0 0.0 # (r3v8r3v6) 45
0.00000000 0.00000000 0.00000000 1.0 # electr. mom.
0.00000000 0.00000000 0.00000000 # magnet. mom.
0 0.0 # (r3v9r3v6) 45
0.00000000 0.00000000 0.00000000 1.0 # electr. mom.
0.00000000 0.00000000 0.00000000 # magnet. mom.
45 10898.0 # 917.6 nm, (r7r3v6) 45
-0.09653 0.148184 0.00000000 1.0 # electr. mom.
0.00000000 0.00000000 0.0813 # magnet. mom.
1.31876649 -1.08237463 0.00000000 -97.84264505
0.00000000 -0.70208822 0.00000000 1.06407233
2.11091906 0.04406596 0.00000000 -24.68714249
1.32524096 1.15754064 0.00000000 -29.60140925
-0.04178899 0.70146698 0.00000000 49.56357969
-1.16182154 -1.48163476 0.00000000 9.06534723
-2.37713566 -0.81881509 0.00000000 -25.05546753
-2.44031774 0.59060577 0.00000000 -10.02298796
-1.28611065 1.35334636 0.00000000 -9.54464457
1.26584879 -1.08237463 0.00000000 14.38908276
1.37168419 -1.08237463 0.00000000 17.09776151
1.31876649 -1.13529233 0.00000000 29.93980937
1.31876649 -1.02945693 0.00000000 36.68045281
-0.05291770 -0.70208822 0.00000000 -11.98555305
0.05291770 -0.70208822 0.00000000 -13.15234631
0.00000000 -0.75500592 0.00000000 12.95916353
0.00000000 -0.64917052 0.00000000 11.23922553
2.05800137 0.04406596 0.00000000 -7.23268895
2.16383676 0.04406596 0.00000000 -4.05140716
2.11091906 -0.00885174 0.00000000 17.67769205
2.11091906 0.09698366 0.00000000 17.86128621
1.27232327 1.15754064 0.00000000 -2.98231622
1.37815866 1.15754064 0.00000000 -2.69117248
1.32524096 1.10462294 0.00000000 19.66779528
1.32524096 1.21045834 0.00000000 15.91327748
-0.09470669 0.70146698 0.00000000 -23.74184135
0.01112871 0.70146698 0.00000000 -26.64631255
-0.04178899 0.64854929 0.00000000 -0.75810223
-0.04178899 0.75438469 0.00000000 1.39054190
-1.21473924 -1.48163476 0.00000000 -3.53787868
-1.10890384 -1.48163476 0.00000000 -3.77640394
-1.16182154 -1.53455246 0.00000000 -0.21769035
-1.16182154 -1.42871706 0.00000000 -1.75307122
-2.43005336 -0.81881509 0.00000000 3.22873497
-2.32421796 -0.81881509 0.00000000 4.55156172
-2.37713566 -0.87173279 0.00000000 8.23973249
-2.37713566 -0.76589740 0.00000000 9.21265306
-2.49323545 0.59060577 0.00000000 2.58419405
-2.38740004 0.59060577 0.00000000 2.77594449
-2.44031774 0.53768808 0.00000000 2.10521373
-2.44031774 0.64352347 0.00000000 2.54245244
-1.33902835 1.35334636 0.00000000 -4.91215803
-1.23319294 1.35334636 0.00000000 -5.17009869
-1.28611065 1.30042866 0.00000000 9.96047215
-1.28611065 1.40626406 0.00000000 9.65329127
45 12979.0 # 770.5 nm, (r8r3v6) 45
-0.05567 -0.07524 0.00000000 1.0 # electr. mom.
0.00000000 0.00000000 0.1614 # magnet. mom.
1.31876649 -1.08237463 0.00000000 -1.74382716
0.00000000 -0.70208822 0.00000000 55.47498418
2.11091906 0.04406596 0.00000000 31.39442343
1.32524096 1.15754064 0.00000000 -12.30959044
-0.04178899 0.70146698 0.00000000 -34.79962715
-1.16182154 -1.48163476 0.00000000 -31.04217319
-2.37713566 -0.81881509 0.00000000 20.25549715
-2.44031774 0.59060577 0.00000000 2.14077958
-1.28611065 1.35334636 0.00000000 13.36093160
1.26584879 -1.08237463 0.00000000 3.30044826
1.37168419 -1.08237463 0.00000000 2.68573287
1.31876649 -1.13529233 0.00000000 -2.32771858
1.31876649 -1.02945693 0.00000000 -1.80014024
-0.05291770 -0.70208822 0.00000000 -21.26044639
0.05291770 -0.70208822 0.00000000 -20.45687130
0.00000000 -0.75500592 0.00000000 -6.94242522
0.00000000 -0.64917052 0.00000000 -7.28779554
2.05800137 0.04406596 0.00000000 -7.70280612
2.16383676 0.04406596 0.00000000 -7.08991710
2.11091906 -0.00885174 0.00000000 -8.29929179
2.11091906 0.09698366 0.00000000 -8.31185765
1.27232327 1.15754064 0.00000000 9.32180361
1.37815866 1.15754064 0.00000000 8.46684485
1.32524096 1.10462294 0.00000000 -3.20549798
1.32524096 1.21045834 0.00000000 -2.31863336
-0.09470669 0.70146698 0.00000000 13.37361496
0.01112871 0.70146698 0.00000000 14.10684490
-0.04178899 0.64854929 0.00000000 5.10107167
-0.04178899 0.75438469 0.00000000 2.42877373

-1.21473924 -1.48163476 0.00000000 5.52765067
-1.10890384 -1.48163476 0.00000000 5.63888310
-1.16182154 -1.53455246 0.00000000 8.17691174
-1.16182154 -1.42871706 0.00000000 12.12720043
-2.43005336 -0.81881509 0.00000000 -12.53086917
-2.32421796 -0.81881509 0.00000000 -15.37132375
-2.37713566 -0.87173279 0.00000000 2.81456381
-2.37713566 -0.76589740 0.00000000 4.76497378
-2.49323545 0.59060577 0.00000000 6.15904952
-2.38740004 0.59060577 0.00000000 7.23794606
-2.44031774 0.53768808 0.00000000 -8.38133533
-2.44031774 0.64352347 0.00000000 -7.25635770
-1.33902835 1.35334636 0.00000000 0.32116186
-1.23319294 1.35334636 0.00000000 -0.60427669
-1.28611065 1.30042866 0.00000000 -7.08708641
-1.28611065 1.40626406 0.00000000 -6.05022350
&TRANSITION 15->...
0 0.0 # (r3v8r3v7) 45
0.00000000 0.00000000 0.00000000 1.0 # electr. mom.
0.00000000 0.00000000 0.00000000 # magnet. mom.
0 0.0 # (r3v9r3v7) 45
0.00000000 0.00000000 0.00000000 1.0 # electr. mom.
0.00000000 0.00000000 0.00000000 # magnet. mom.
45 10898.0 # 917.6 nm, (r7r3v7) 45
-0.10189 0.156416 0.00000000 1.0 # electr. mom.
0.00000000 0.00000000 0.0813 # magnet. mom.
1.31876649 -1.08237463 0.00000000 -103.27834755
0.00000000 -0.70208822 0.00000000 1.12318746
2.11091906 0.04406596 0.00000000 -26.05865041
1.32524096 1.15754064 0.00000000 -31.24593199
-0.04178899 0.70146698 0.00000000 52.31711190
-1.16182154 -1.48163476 0.00000000 9.56897764
-2.37713566 -0.81881509 0.00000000 -26.44743794
-2.44031774 0.59060577 0.00000000 -10.57982063
-1.28611065 1.35334636 0.00000000 -10.07490260
1.26584879 -1.08237463 0.00000000 15.18847625
1.37168419 -1.08237463 0.00000000 18.04763715
1.31876649 -1.13529233 0.00000000 31.60313211
1.31876649 -1.02945693 0.00000000 38.71825574
-0.05291770 -0.70208822 0.00000000 -12.65141711
0.05291770 -0.70208822 0.00000000 -13.88303221
0.00000000 -0.75500592 0.00000000 13.67911706
0.00000000 -0.64917052 0.00000000 11.86362695
2.05800137 0.04406596 0.00000000 -7.63450501
2.16383676 0.04406596 0.00000000 -4.27648534
2.11091906 -0.00885174 0.00000000 18.65978606
2.11091906 0.09698366 0.00000000 18.85357988
1.27232327 1.15754064 0.00000000 -3.14800045
1.37815866 1.15754064 0.00000000 -2.84068206
1.32524096 1.10462294 0.00000000 20.76045058
1.32524096 1.21045834 0.00000000 16.79734845
-0.09470669 0.70146698 0.00000000 -25.06083253
0.01112871 0.70146698 0.00000000 -28.12666325
-0.04178899 0.64854929 0.00000000 -0.80021902
-0.04178899 0.75438469 0.00000000 1.46779423
-1.21473924 -1.48163476 0.00000000 -3.73442749
-1.10890384 -1.48163476 0.00000000 -3.98620416
-1.16182154 -1.53455246 0.00000000 -0.22978426
-1.16182154 -1.42871706 0.00000000 -1.85046407
-2.43005336 -0.81881509 0.00000000 3.40810914
-2.32421796 -0.81881509 0.00000000 4.80442626
-2.37713566 -0.87173279 0.00000000 8.69749541
-2.37713566 -0.76589740 0.00000000 9.72446712
-2.49323545 0.59060577 0.00000000 2.72776039
-2.38740004 0.59060577 0.00000000 2.93016363
-2.44031774 0.53768808 0.00000000 2.22217004
-2.44031774 0.64352347 0.00000000 2.68369980
-1.33902835 1.35334636 0.00000000 -5.18505570
-1.23319294 1.35334636 0.00000000 -5.45732640
-1.28611065 1.30042866 0.00000000 10.51383172
-1.28611065 1.40626406 0.00000000 10.18958523
45 12979.0 # 770.5 nm, (r8r3v7) 45
-0.05876 -0.07942 0.00000000 1.0 # electr. mom.
0.00000000 0.00000000 0.1614 # magnet. mom.
1.31876649 -1.08237463 0.00000000 -1.84070645
0.00000000 -0.70208822 0.00000000 58.55692774
2.11091906 0.04406596 0.00000000 33.13855807
1.32524096 1.15754064 0.00000000 -12.99345658
-0.04178899 0.70146698 0.00000000 -36.73293977
-1.16182154 -1.48163476 0.00000000 -32.76673836
-2.37713566 -0.81881509 0.00000000 21.38080254
-2.44031774 0.59060577 0.00000000 2.25971178
-1.28611065 1.35334636 0.00000000 14.10320557
1.26584879 -1.08237463 0.00000000 3.48380649
1.37168419 -1.08237463 0.00000000 2.83494025
1.31876649 -1.13529233 0.00000000 -2.45703628
1.31876649 -1.02945693 0.00000000 -1.90014803

```
-0.05291770    -0.70208822    0.00000000    -22.44158230
0.05291770    -0.70208822    0.00000000    -21.59336415
0.00000000    -0.75500592    0.00000000    -7.32811551
0.00000000    -0.64917052    0.00000000    -7.69267307
2.05800137    0.04406596    0.00000000    -8.13073979
2.16383676    0.04406596    0.00000000    -7.48380138
2.11091906    -0.00885174    0.00000000    -8.76036355
2.11091906    0.09698366    0.00000000    -8.77362752
1.27232327    1.15754064    0.00000000    9.83968159
1.37815866    1.15754064    0.00000000    8.93722512
1.32524096    1.10462294    0.00000000    -3.38358120
1.32524096    1.21045834    0.00000000    -2.44744632
-0.09470669    0.70146698    0.00000000    14.11659357
0.01112871    0.70146698    0.00000000    14.89055850
-0.04178899    0.64854929    0.00000000    5.38446454
-0.04178899    0.75438469    0.00000000    2.56370561
-1.21473924    -1.48163476    0.00000000    5.83474237
-1.10890384    -1.48163476    0.00000000    5.95215438
-1.16182154    -1.53455246    0.00000000    8.63118462
-1.16182154    -1.42871706    0.00000000    12.80093379
-2.43005336    -0.81881509    0.00000000    -13.22702857
-2.32421796    -0.81881509    0.00000000    -16.22528618
-2.37713566    -0.87173279    0.00000000    2.97092846
-2.37713566    -0.76589740    0.00000000    5.02969455
-2.49323545    0.59060577    0.00000000    6.50121894
-2.38740004    0.59060577    0.00000000    7.64005418
-2.44031774    0.53768808    0.00000000    -8.84696507
-2.44031774    0.64352347    0.00000000    -7.65948868
-1.33902835    1.35334636    0.00000000    0.33900418
-1.23319294    1.35334636    0.00000000    -0.63784762
-1.28611065    1.30042866    0.00000000    -7.48081344
-1.28611065    1.40626406    0.00000000    -6.38634702
&TRANSITION 16->...
0    0.0    # (r3v9r3v8) 45
0.00000000    0.00000000    0.00000000 1.0    # electr. mom.
0.00000000    0.00000000    0.00000000    # magnet. mom.
45    10898.0    # 917.6 nm,    (r7r3v8) 45
-0.0429    0.065859    0.00000000 1.0    # electr. mom.
0.00000000    0.00000000    0.0813    # magnet. mom.
1.31876649    -1.08237463    0.00000000    -43.48562002
0.00000000    -0.70208822    0.00000000    0.47292104
2.11091906    0.04406596    0.00000000    -10.97206333
1.32524096    1.15754064    0.00000000    -13.15618189
-0.04178899    0.70146698    0.00000000    22.02825764
-1.16182154    -1.48163476    0.00000000    4.02904322
-2.37713566    -0.81881509    0.00000000    -11.13576334
-2.44031774    0.59060577    0.00000000    -4.45466132
-1.28611065    1.35334636    0.00000000    -4.24206425
1.26584879    -1.08237463    0.00000000    6.39514789
1.37168419    -1.08237463    0.00000000    7.59900512
1.31876649    -1.13529233    0.00000000    13.30658194
1.31876649    -1.02945693    0.00000000    16.30242347
-0.05291770    -0.70208822    0.00000000    -5.32691247
0.05291770    -0.70208822    0.00000000    -5.84548725
0.00000000    -0.75500592    0.00000000    5.75962823
0.00000000    -0.64917052    0.00000000    4.99521135
2.05800137    0.04406596    0.00000000    -3.21452842
2.16383676    0.04406596    0.00000000    -1.80062541
2.11091906    -0.00885174    0.00000000    7.85675202
2.11091906    0.09698366    0.00000000    7.93834942
1.27232327    1.15754064    0.00000000    -1.32547387
1.37815866    1.15754064    0.00000000    -1.19607666
1.32524096    1.10462294    0.00000000    8.74124235
1.32524096    1.21045834    0.00000000    7.07256777
-0.09470669    0.70146698    0.00000000    -10.55192949
0.01112871    0.70146698    0.00000000    -11.84280558
-0.04178899    0.64854929    0.00000000    -0.33693433
-0.04178899    0.75438469    0.00000000    0.61801862
-1.21473924    -1.48163476    0.00000000    -1.57239052
-1.10890384    -1.48163476    0.00000000    -1.67840175
-1.16182154    -1.53455246    0.00000000    -0.09675127
-1.16182154    -1.42871706    0.00000000    -0.77914277
-2.43005336    -0.81881509    0.00000000    1.43499332
-2.32421796    -0.81881509    0.00000000    2.02291632
-2.37713566    -0.87173279    0.00000000    3.66210333
-2.37713566    -0.76589740    0.00000000    4.09451247
-2.49323545    0.59060577    0.00000000    1.14853069
-2.38740004    0.59060577    0.00000000    1.23375311
-2.44031774    0.53768808    0.00000000    0.93565054
-2.44031774    0.64352347    0.00000000    1.12997886
-1.33902835    1.35334636    0.00000000    -2.18318135
-1.23319294    1.35334636    0.00000000    -2.29782164
-1.28611065    1.30042866    0.00000000    4.42687651
-1.28611065    1.40626406    0.00000000    4.29035168
45    12979.0    # 770.5 nm,    (r8r3v8) 45
-0.02474    -0.03344    0.00000000 1.0    # electr. mom.
0.00000000    0.00000000    0.1614    # magnet. mom.
```

1.31876649	-1.08237463	0.00000000	-0.77503429
0.00000000	-0.70208822	0.00000000	24.65554852
2.11091906	0.04406596	0.00000000	13.95307708
1.32524096	1.15754064	0.00000000	-5.47092909
-0.04178899	0.70146698	0.00000000	-15.46650096
-1.16182154	-1.48163476	0.00000000	-13.79652142
-2.37713566	-0.81881509	0.00000000	9.00244318
-2.44031774	0.59060577	0.00000000	0.95145759
-1.28611065	1.35334636	0.00000000	5.93819182
1.26584879	-1.08237463	0.00000000	1.46686589
1.37168419	-1.08237463	0.00000000	1.19365905
1.31876649	-1.13529233	0.00000000	-1.03454159
1.31876649	-1.02945693	0.00000000	-0.80006233
-0.05291770	-0.70208822	0.00000000	-9.44908729
0.05291770	-0.70208822	0.00000000	-9.09194280
0.00000000	-0.75500592	0.00000000	-3.08552232
0.00000000	-0.64917052	0.00000000	-3.23902024
2.05800137	0.04406596	0.00000000	-3.42346939
2.16383676	0.04406596	0.00000000	-3.15107427
2.11091906	-0.00885174	0.00000000	-3.68857413
2.11091906	0.09698366	0.00000000	-3.69415895
1.27232327	1.15754064	0.00000000	4.14302383
1.37815866	1.15754064	0.00000000	3.76304216
1.32524096	1.10462294	0.00000000	-1.42466577
1.32524096	1.21045834	0.00000000	-1.03050371
-0.09470669	0.70146698	0.00000000	5.94382887
0.01112871	0.70146698	0.00000000	6.26970884
-0.04178899	0.64854929	0.00000000	2.26714297
-0.04178899	0.75438469	0.00000000	1.07945499
-1.21473924	-1.48163476	0.00000000	2.45673363
-1.10890384	-1.48163476	0.00000000	2.50617027
-1.16182154	-1.53455246	0.00000000	3.63418300
-1.16182154	-1.42871706	0.00000000	5.38986686
-2.43005336	-0.81881509	0.00000000	-5.56927519
-2.32421796	-0.81881509	0.00000000	-6.83169944
-2.37713566	-0.87173279	0.00000000	1.25091725
-2.37713566	-0.76589740	0.00000000	2.11776612
-2.49323545	0.59060577	0.00000000	2.73735534
-2.38740004	0.59060577	0.00000000	3.21686492
-2.44031774	0.53768808	0.00000000	-3.72503792
-2.44031774	0.64352347	0.00000000	-3.22504787
-1.33902835	1.35334636	0.00000000	0.14273860
-1.23319294	1.35334636	0.00000000	-0.26856742
-1.28611065	1.30042866	0.00000000	-3.14981618
-1.28611065	1.40626406	0.00000000	-2.68898822

&TRANSITION 17->...

45 10898.0 # 917.6 nm, (r7r3v9) 45

-0.05363 0.082324 0.00000000 1.0 # electr. mom.

0.00000000 0.00000000 0.0813 # magnet. mom.

1.31876649	-1.08237463	0.00000000	-54.35702503
0.00000000	-0.70208822	0.00000000	0.59115130
2.11091906	0.04406596	0.00000000	-13.71507916
1.32524096	1.15754064	0.00000000	-16.44522736
-0.04178899	0.70146698	0.00000000	27.53532205
-1.16182154	-1.48163476	0.00000000	5.03630402
-2.37713566	-0.81881509	0.00000000	-13.91970418
-2.44031774	0.59060577	0.00000000	-5.56832665
-1.28611065	1.35334636	0.00000000	-5.30258032
1.26584879	-1.08237463	0.00000000	7.99393487
1.37168419	-1.08237463	0.00000000	9.49875639
1.31876649	-1.13529233	0.00000000	16.63322743
1.31876649	-1.02945693	0.00000000	20.37802934
-0.05291770	-0.70208822	0.00000000	-6.65864058
0.05291770	-0.70208822	0.00000000	-7.30685906
0.00000000	-0.75500592	0.00000000	7.19953529
0.00000000	-0.64917052	0.00000000	6.24401418
2.05800137	0.04406596	0.00000000	-4.01816053
2.16383676	0.04406596	0.00000000	-2.25078176
2.11091906	-0.00885174	0.00000000	9.82094003
2.11091906	0.09698366	0.00000000	9.92293678
1.27232327	1.15754064	0.00000000	-1.65684234
1.37815866	1.15754064	0.00000000	-1.49509582
1.32524096	1.10462294	0.00000000	10.92655294
1.32524096	1.21045834	0.00000000	8.84070971
-0.09470669	0.70146698	0.00000000	-13.18991186
0.01112871	0.70146698	0.00000000	-14.80350697
-0.04178899	0.64854929	0.00000000	-0.42116791
-0.04178899	0.75438469	0.00000000	0.77252328
-1.21473924	-1.48163476	0.00000000	-1.96548816
-1.10890384	-1.48163476	0.00000000	-2.09800219
-1.16182154	-1.53455246	0.00000000	-0.12093908
-1.16182154	-1.42871706	0.00000000	-0.97392846
-2.43005336	-0.81881509	0.00000000	1.79374165
-2.32421796	-0.81881509	0.00000000	2.52864540
-2.37713566	-0.87173279	0.00000000	4.57762916
-2.37713566	-0.76589740	0.00000000	5.11814059
-2.49323545	0.59060577	0.00000000	1.43566336

-2.38740004 0.59060577 0.00000000 1.54219139
-2.44031774 0.53768808 0.00000000 1.16956318
-2.44031774 0.64352347 0.00000000 1.41247358
-1.33902835 1.35334636 0.00000000 -2.72897668
-1.23319294 1.35334636 0.00000000 -2.87227705
-1.28611065 1.30042866 0.00000000 5.53359564
-1.28611065 1.40626406 0.00000000 5.36293959
45 12979.0 # 770.5 nm, (r8r3v9) 45
-0.03093 -0.0418 0.00000000 1.0 # electr. mom.
0.00000000 0.00000000 0.1614 # magnet. mom.
1.31876649 -1.08237463 0.00000000 -0.96879287
0.00000000 -0.70208822 0.00000000 30.81943565
2.11091906 0.04406596 0.00000000 17.44134635
1.32524096 1.15754064 0.00000000 -6.83866136
-0.04178899 0.70146698 0.00000000 -19.33312620
-1.16182154 -1.48163476 0.00000000 -17.24565177
-2.37713566 -0.81881509 0.00000000 11.25305397
-2.44031774 0.59060577 0.00000000 1.18932199
-1.28611065 1.35334636 0.00000000 7.42273978
1.26584879 -1.08237463 0.00000000 1.83358237
1.37168419 -1.08237463 0.00000000 1.49207382
1.31876649 -1.13529233 0.00000000 -1.29317699
1.31876649 -1.02945693 0.00000000 -1.00007791
-0.05291770 -0.70208822 0.00000000 -11.81135911
0.05291770 -0.70208822 0.00000000 -11.36492850
0.00000000 -0.75500592 0.00000000 -3.85690290
0.00000000 -0.64917052 0.00000000 -4.04877530
2.05800137 0.04406596 0.00000000 -4.27933673
2.16383676 0.04406596 0.00000000 -3.93884283
2.11091906 -0.00885174 0.00000000 -4.61071766
2.11091906 0.09698366 0.00000000 -4.61769869
1.27232327 1.15754064 0.00000000 5.17877978
1.37815866 1.15754064 0.00000000 4.70380270
1.32524096 1.10462294 0.00000000 -1.78083221
1.32524096 1.21045834 0.00000000 -1.28812964
-0.09470669 0.70146698 0.00000000 7.42978609
0.01112871 0.70146698 0.00000000 7.83713605
-0.04178899 0.64854929 0.00000000 2.83392871
-0.04178899 0.75438469 0.00000000 1.34931874
-1.21473924 -1.48163476 0.00000000 3.07091704
-1.10890384 -1.48163476 0.00000000 3.13271283
-1.16182154 -1.53455246 0.00000000 4.54272875
-1.16182154 -1.42871706 0.00000000 6.73733357
-2.43005336 -0.81881509 0.00000000 -6.96159398
-2.32421796 -0.81881509 0.00000000 -8.53962431
-2.37713566 -0.87173279 0.00000000 1.56364656
-2.37713566 -0.76589740 0.00000000 2.64720766
-2.49323545 0.59060577 0.00000000 3.42169418
-2.38740004 0.59060577 0.00000000 4.02108115
-2.44031774 0.53768808 0.00000000 -4.65629740
-2.44031774 0.64352347 0.00000000 -4.03130983
-1.33902835 1.35334636 0.00000000 0.17842325
-1.23319294 1.35334636 0.00000000 -0.33570927
-1.28611065 1.30042866 0.00000000 -3.93727023
-1.28611065 1.40626406 0.00000000 -3.36123528
&TRANSITION 18->...
45 2081.0 # 4805.4 nm, (r8r7) 45
1.41269163 0.70347288 0.00000000 1.0 # electr. mom.
0.00000000 0.00000000 -0.3555450588E-01 # magnet. mom.
1.31876649 -1.08237463 0.00000000 28.27766678
0.00000000 -0.70208822 0.00000000 -44.77598434
2.11091906 0.04406596 0.00000000 291.43570502
1.32524096 1.15754064 0.00000000 250.46582554
-0.04178899 0.70146698 0.00000000 -284.70533261
-1.16182154 -1.48163476 0.00000000 49.11470827
-2.37713566 -0.81881509 0.00000000 17.32325856
-2.44031774 0.59060577 0.00000000 5.07931306
-1.28611065 1.35334636 0.00000000 250.31193922
1.26584879 -1.08237463 0.00000000 -17.73126048
1.37168419 -1.08237463 0.00000000 -21.08065387
1.31876649 -1.13529233 0.00000000 1.35266342
1.31876649 -1.02945693 0.00000000 9.92360659
-0.05291770 -0.70208822 0.00000000 -54.91585742
0.05291770 -0.70208822 0.00000000 -55.50295042
0.00000000 -0.75500592 0.00000000 69.43104130
0.00000000 -0.64917052 0.00000000 85.99201459
2.05800137 0.04406596 0.00000000 -128.15565874
2.16383676 0.04406596 0.00000000 -114.82075243
2.11091906 -0.00885174 0.00000000 -24.46431936
2.11091906 0.09698366 0.00000000 -24.40568309
1.27232327 1.15754064 0.00000000 -20.39239699
1.37815866 1.15754064 0.00000000 -15.60858571
1.32524096 1.10462294 0.00000000 -112.24425250
1.32524096 1.21045834 0.00000000 -102.84322262
-0.09470669 0.70146698 0.00000000 165.10645330
0.01112871 0.70146698 0.00000000 171.56927914
-0.04178899 0.64854929 0.00000000 -32.38792450

```
-0.04178899   0.75438469   0.00000000   -18.65421058
-1.21473924  -1.48163476   0.00000000    -0.49403299
-1.10890384  -1.48163476   0.00000000     0.61869638
-1.16182154  -1.53455246   0.00000000   -25.27994438
-1.16182154  -1.42871706   0.00000000   -23.85349363
-2.43005336  -0.81881509   0.00000000   -12.01953317
-2.32421796  -0.81881509   0.00000000   -12.96148906
-2.37713566  -0.87173279   0.00000000     3.85913549
-2.37713566  -0.76589740   0.00000000     3.66723806
-2.49323545   0.59060577   0.00000000    17.96788176
-2.38740004   0.59060577   0.00000000    26.43586075
-2.44031774   0.53768808   0.00000000   -26.38711123
-2.44031774   0.64352347   0.00000000   -22.48966738
-1.33902835   1.35334636   0.00000000   -66.65013235
-1.23319294   1.35334636   0.00000000   -65.80104547
-1.28611065   1.30042866   0.00000000   -65.07230630
-1.28611065   1.40626406   0.00000000   -54.23448562
&PERMANENT MOMENTS
45 0.0 # 0 nm, Grd. (r1r1) 45
 1.73104840  -1.92708772   0.00000000 1.0 # electr. mom.
 1.31876649  -1.08237463   0.00000000 -3391.09120171
 0.00000000  -0.70208822   0.00000000   468.91078354
 2.11091906   0.04406596   0.00000000   909.02213645
 1.32524096   1.15754064   0.00000000 -2704.30951382
-0.04178899   0.70146698   0.00000000   514.78806499
-1.16182154  -1.48163476   0.00000000   121.27062902
-2.37713566  -0.81881509   0.00000000 -1042.97614522
-2.44031774   0.59060577   0.00000000 -1948.63292904
-1.28611065   1.35334636   0.00000000   316.62313320
 1.26584879  -1.08237463   0.00000000   172.02859627
 1.37168419  -1.08237463   0.00000000   233.78238619
 1.31876649  -1.13529233   0.00000000  1408.19312971
 1.31876649  -1.02945693   0.00000000  1590.90062458
-0.05291770  -0.70208822   0.00000000   163.01598432
 0.05291770  -0.70208822   0.00000000    93.43527178
 0.00000000  -0.75500592   0.00000000  -279.81228538
 0.00000000  -0.64917052   0.00000000  -449.26786296
 2.05800137   0.04406596   0.00000000  -281.46868037
 2.16383676   0.04406596   0.00000000   -78.52429858
 2.11091906  -0.00885174   0.00000000  -279.86375899
 2.11091906   0.09698366   0.00000000  -291.75613877
 1.27232327   1.15754064   0.00000000   182.00207626
 1.37815866   1.15754064   0.00000000   233.68582594
 1.32524096   1.10462294   0.00000000  1244.81949494
 1.32524096   1.21045834   0.00000000  1058.20231882
-0.09470669   0.70146698   0.00000000  -990.36610550
 0.01112871   0.70146698   0.00000000 -1030.32224947
-0.04178899   0.64854929   0.00000000   756.10156478
-0.04178899   0.75438469   0.00000000   753.95467747
-1.21473924  -1.48163476   0.00000000  -313.59839688
-1.10890384  -1.48163476   0.00000000  -309.01455542
-1.16182154  -1.53455246   0.00000000   274.53066826
-1.16182154  -1.42871706   0.00000000   217.89279336
-2.43005336  -0.81881509   0.00000000   396.14576093
-2.32421796  -0.81881509   0.00000000   448.55416094
-2.37713566  -0.87173279   0.00000000   135.10028410
-2.37713566  -0.76589740   0.00000000    63.77974733
-2.49323545   0.59060577   0.00000000   226.44929184
-2.38740004   0.59060577   0.00000000   229.65384322
-2.44031774   0.53768808   0.00000000   764.74581840
-2.44031774   0.64352347   0.00000000   733.45554720
-1.33902835   1.35334636   0.00000000  -650.01981274
-1.23319294   1.35334636   0.00000000  -675.01151530
-1.28611065   1.30042866   0.00000000   514.53465653
-1.28611065   1.40626406   0.00000000   490.45617979
45 0.0 # 0 nm, 1st Exc. (r2v1r2v1) 45
 1.74098131  -1.46780024   0.00000000 1.0 # electr. mom.
 1.31876649  -1.08237463   0.00000000 -3637.42173509
 0.00000000  -0.70208822   0.00000000  1463.58358291
 2.11091906   0.04406596   0.00000000   827.34939854
 1.32524096   1.15754064   0.00000000 -2731.65780005
-0.04178899   0.70146698   0.00000000   740.99122666
-1.16182154  -1.48163476   0.00000000  -301.58102534
-2.37713566  -0.81881509   0.00000000 -1111.45560634
-2.44031774   0.59060577   0.00000000 -1930.01932901
-1.28611065   1.35334636   0.00000000   171.55611859
 1.26584879  -1.08237463   0.00000000   297.61113893
 1.37168419  -1.08237463   0.00000000   344.11727213
 1.31876649  -1.13529233   0.00000000  1409.80008139
 1.31876649  -1.02945693   0.00000000  1602.16176106
-0.05291770  -0.70208822   0.00000000  -157.40184773
 0.05291770  -0.70208822   0.00000000  -233.04201086
 0.00000000  -0.75500592   0.00000000  -450.63016843
 0.00000000  -0.64917052   0.00000000  -632.53874990
 2.05800137   0.04406596   0.00000000  -229.52602070
 2.16383676   0.04406596   0.00000000   -32.12191310
 2.11091906  -0.00885174   0.00000000  -291.64397170
```

2.11091906	0.09698366	0.00000000	-296.91813470
1.27232327	1.15754064	0.00000000	174.91054651
1.37815866	1.15754064	0.00000000	222.30167765
1.32524096	1.10462294	0.00000000	1270.48523702
1.32524096	1.21045834	0.00000000	1079.24918803
-0.09470669	0.70146698	0.00000000	-1250.82118382
0.01112871	0.70146698	0.00000000	-1293.61212259
-0.04178899	0.64854929	0.00000000	926.89080523
-0.04178899	0.75438469	0.00000000	881.53434384
-1.21473924	-1.48163476	0.00000000	-198.39718790
-1.10890384	-1.48163476	0.00000000	-188.34576802
-1.16182154	-1.53455246	0.00000000	358.63747334
-1.16182154	-1.42871706	0.00000000	322.44546130
-2.43005336	-0.81881509	0.00000000	424.97900290
-2.32421796	-0.81881509	0.00000000	483.69524501
-2.37713566	-0.87173279	0.00000000	139.56833405
-2.37713566	-0.76589740	0.00000000	63.18229838
-2.49323545	0.59060577	0.00000000	224.14676232
-2.38740004	0.59060577	0.00000000	231.94534584
-2.44031774	0.53768808	0.00000000	760.31130835
-2.44031774	0.64352347	0.00000000	719.80761187
-1.33902835	1.35334636	0.00000000	-580.14101088
-1.23319294	1.35334636	0.00000000	-606.45578904
-1.28611065	1.30042866	0.00000000	522.01681008
-1.28611065	1.40626406	0.00000000	490.45334328

45 0.0 # 0 nm, 1st Exc. (r2v2r2v2) 45

1.74098131	-1.46780024	0.00000000 1.0 # electr. mom.	
1.31876649	-1.08237463	0.00000000	-3637.42173509
0.00000000	-0.70208822	0.00000000	1463.58358291
2.11091906	0.04406596	0.00000000	827.34939854
1.32524096	1.15754064	0.00000000	-2731.65780005
-0.04178899	0.70146698	0.00000000	740.99122666
-1.16182154	-1.48163476	0.00000000	-301.58102534
-2.37713566	-0.81881509	0.00000000	-1111.45560634
-2.44031774	0.59060577	0.00000000	-1930.01932901
-1.28611065	1.35334636	0.00000000	171.55611859
1.26584879	-1.08237463	0.00000000	297.61113893
1.37168419	-1.08237463	0.00000000	344.11727213
1.31876649	-1.13529233	0.00000000	1409.80008139
1.31876649	-1.02945693	0.00000000	1602.16176106
-0.05291770	-0.70208822	0.00000000	-157.40184773
0.05291770	-0.70208822	0.00000000	-233.04201086
0.00000000	-0.75500592	0.00000000	-450.63016843
0.00000000	-0.64917052	0.00000000	-632.53874990
2.05800137	0.04406596	0.00000000	-229.52602070
2.16383676	0.04406596	0.00000000	-32.12191310
2.11091906	-0.00885174	0.00000000	-291.64397170
2.11091906	0.09698366	0.00000000	-296.91813470
1.27232327	1.15754064	0.00000000	174.91054651
1.37815866	1.15754064	0.00000000	222.30167765
1.32524096	1.10462294	0.00000000	1270.48523702
1.32524096	1.21045834	0.00000000	1079.24918803
-0.09470669	0.70146698	0.00000000	-1250.82118382
0.01112871	0.70146698	0.00000000	-1293.61212259
-0.04178899	0.64854929	0.00000000	926.89080523
-0.04178899	0.75438469	0.00000000	881.53434384
-1.21473924	-1.48163476	0.00000000	-198.39718790
-1.10890384	-1.48163476	0.00000000	-188.34576802
-1.16182154	-1.53455246	0.00000000	358.63747334
-1.16182154	-1.42871706	0.00000000	322.44546130
-2.43005336	-0.81881509	0.00000000	424.97900290
-2.32421796	-0.81881509	0.00000000	483.69524501
-2.37713566	-0.87173279	0.00000000	139.56833405
-2.37713566	-0.76589740	0.00000000	63.18229838
-2.49323545	0.59060577	0.00000000	224.14676232
-2.38740004	0.59060577	0.00000000	231.94534584
-2.44031774	0.53768808	0.00000000	760.31130835
-2.44031774	0.64352347	0.00000000	719.80761187
-1.33902835	1.35334636	0.00000000	-580.14101088
-1.23319294	1.35334636	0.00000000	-606.45578904
-1.28611065	1.30042866	0.00000000	522.01681008
-1.28611065	1.40626406	0.00000000	490.45334328

45 0.0 # 0 nm, 1st Exc. (r2v3r2v3) 45

1.74098131	-1.46780024	0.00000000 1.0 # electr. mom.	
1.31876649	-1.08237463	0.00000000	-3637.42173509
0.00000000	-0.70208822	0.00000000	1463.58358291
2.11091906	0.04406596	0.00000000	827.34939854
1.32524096	1.15754064	0.00000000	-2731.65780005
-0.04178899	0.70146698	0.00000000	740.99122666
-1.16182154	-1.48163476	0.00000000	-301.58102534
-2.37713566	-0.81881509	0.00000000	-1111.45560634
-2.44031774	0.59060577	0.00000000	-1930.01932901
-1.28611065	1.35334636	0.00000000	171.55611859
1.26584879	-1.08237463	0.00000000	297.61113893
1.37168419	-1.08237463	0.00000000	344.11727213
1.31876649	-1.13529233	0.00000000	1409.80008139
1.31876649	-1.02945693	0.00000000	1602.16176106

```
-0.05291770 -0.70208822 0.00000000 -157.40184773
0.05291770 -0.70208822 0.00000000 -233.04201086
0.00000000 -0.75500592 0.00000000 -450.63016843
0.00000000 -0.64917052 0.00000000 -632.53874990
2.05800137 0.04406596 0.00000000 -229.52602070
2.16383676 0.04406596 0.00000000 -32.12191310
2.11091906 -0.00885174 0.00000000 -291.64397170
2.11091906 0.09698366 0.00000000 -296.91813470
1.27232327 1.15754064 0.00000000 174.91054651
1.37815866 1.15754064 0.00000000 222.30167765
1.32524096 1.10462294 0.00000000 1270.48523702
1.32524096 1.21045834 0.00000000 1079.24918803
-0.09470669 0.70146698 0.00000000 -1250.82118382
0.01112871 0.70146698 0.00000000 -1293.61212259
-0.04178899 0.64854929 0.00000000 926.89080523
-0.04178899 0.75438469 0.00000000 881.53434384
-1.21473924 -1.48163476 0.00000000 -198.39718790
-1.10890384 -1.48163476 0.00000000 -188.34576802
-1.16182154 -1.53455246 0.00000000 358.63747334
-1.16182154 -1.42871706 0.00000000 322.44546130
-2.43005336 -0.81881509 0.00000000 424.97900290
-2.32421796 -0.81881509 0.00000000 483.69524501
-2.37713566 -0.87173279 0.00000000 139.56833405
-2.37713566 -0.76589740 0.00000000 63.18229838
-2.49323545 0.59060577 0.00000000 224.14676232
-2.38740004 0.59060577 0.00000000 231.94534584
-2.44031774 0.53768808 0.00000000 760.31130835
-2.44031774 0.64352347 0.00000000 719.80761187
-1.33902835 1.35334636 0.00000000 -580.14101088
-1.23319294 1.35334636 0.00000000 -606.45578904
-1.28611065 1.30042866 0.00000000 522.01681008
-1.28611065 1.40626406 0.00000000 490.45334328
45 0.0 # 0 nm, 1st Exc. (r2v4r2v4) 45
1.74098131 -1.46780024 0.00000000 1.0 # electr. mom.
1.31876649 -1.08237463 0.00000000 -3637.42173509
0.00000000 -0.70208822 0.00000000 1463.58358291
2.11091906 0.04406596 0.00000000 827.34939854
1.32524096 1.15754064 0.00000000 -2731.65780005
-0.04178899 0.70146698 0.00000000 740.99122666
-1.16182154 -1.48163476 0.00000000 -301.58102534
-2.37713566 -0.81881509 0.00000000 -1111.45560634
-2.44031774 0.59060577 0.00000000 -1930.01932901
-1.28611065 1.35334636 0.00000000 171.55611859
1.26584879 -1.08237463 0.00000000 297.61113893
1.37168419 -1.08237463 0.00000000 344.11727213
1.31876649 -1.13529233 0.00000000 1409.80008139
1.31876649 -1.02945693 0.00000000 1602.16176106
-0.05291770 -0.70208822 0.00000000 -157.40184773
0.05291770 -0.70208822 0.00000000 -233.04201086
0.00000000 -0.75500592 0.00000000 -450.63016843
0.00000000 -0.64917052 0.00000000 -632.53874990
2.05800137 0.04406596 0.00000000 -229.52602070
2.16383676 0.04406596 0.00000000 -32.12191310
2.11091906 -0.00885174 0.00000000 -291.64397170
2.11091906 0.09698366 0.00000000 -296.91813470
1.27232327 1.15754064 0.00000000 174.91054651
1.37815866 1.15754064 0.00000000 222.30167765
1.32524096 1.10462294 0.00000000 1270.48523702
1.32524096 1.21045834 0.00000000 1079.24918803
-0.09470669 0.70146698 0.00000000 -1250.82118382
0.01112871 0.70146698 0.00000000 -1293.61212259
-0.04178899 0.64854929 0.00000000 926.89080523
-0.04178899 0.75438469 0.00000000 881.53434384
-1.21473924 -1.48163476 0.00000000 -198.39718790
-1.10890384 -1.48163476 0.00000000 -188.34576802
-1.16182154 -1.53455246 0.00000000 358.63747334
-1.16182154 -1.42871706 0.00000000 322.44546130
-2.43005336 -0.81881509 0.00000000 424.97900290
-2.32421796 -0.81881509 0.00000000 483.69524501
-2.37713566 -0.87173279 0.00000000 139.56833405
-2.37713566 -0.76589740 0.00000000 63.18229838
-2.49323545 0.59060577 0.00000000 224.14676232
-2.38740004 0.59060577 0.00000000 231.94534584
-2.44031774 0.53768808 0.00000000 760.31130835
-2.44031774 0.64352347 0.00000000 719.80761187
-1.33902835 1.35334636 0.00000000 -580.14101088
-1.23319294 1.35334636 0.00000000 -606.45578904
-1.28611065 1.30042866 0.00000000 522.01681008
-1.28611065 1.40626406 0.00000000 490.45334328
45 0.0 # 0 nm, 1st Exc. (r2v5r2v5) 45
1.74098131 -1.46780024 0.00000000 1.0 # electr. mom.
1.31876649 -1.08237463 0.00000000 -3637.42173509
0.00000000 -0.70208822 0.00000000 1463.58358291
2.11091906 0.04406596 0.00000000 827.34939854
1.32524096 1.15754064 0.00000000 -2731.65780005
-0.04178899 0.70146698 0.00000000 740.99122666
-1.16182154 -1.48163476 0.00000000 -301.58102534
```

-2.37713566 -0.81881509 0.00000000 -1111.45560634
-2.44031774 0.59060577 0.00000000 -1930.01932901
-1.28611065 1.35334636 0.00000000 171.55611859
1.26584879 -1.08237463 0.00000000 297.61113893
1.37168419 -1.08237463 0.00000000 344.11727213
1.31876649 -1.13529233 0.00000000 1409.80008139
1.31876649 -1.02945693 0.00000000 1602.16176106
-0.05291770 -0.70208822 0.00000000 -157.40184773
0.05291770 -0.70208822 0.00000000 -233.04201086
0.00000000 -0.75500592 0.00000000 -450.63016843
0.00000000 -0.64917052 0.00000000 -632.53874990
2.05800137 0.04406596 0.00000000 -229.52602070
2.16383676 0.04406596 0.00000000 -32.12191310
2.11091906 -0.00885174 0.00000000 -291.64397170
2.11091906 0.09698366 0.00000000 -296.91813470
1.27232327 1.15754064 0.00000000 174.91054651
1.37815866 1.15754064 0.00000000 222.30167765
1.32524096 1.10462294 0.00000000 1270.48523702
1.32524096 1.21045834 0.00000000 1079.24918803
-0.09470669 0.70146698 0.00000000 -1250.82118382
0.01112871 0.70146698 0.00000000 -1293.61212259
-0.04178899 0.64854929 0.00000000 926.89080523
-0.04178899 0.75438469 0.00000000 881.53434384
-1.21473924 -1.48163476 0.00000000 -198.39718790
-1.10890384 -1.48163476 0.00000000 -188.34576802
-1.16182154 -1.53455246 0.00000000 358.63747334
-1.16182154 -1.42871706 0.00000000 322.44546130
-2.43005336 -0.81881509 0.00000000 424.97900290
-2.32421796 -0.81881509 0.00000000 483.69524501
-2.37713566 -0.87173279 0.00000000 139.56833405
-2.37713566 -0.76589740 0.00000000 63.18229838
-2.49323545 0.59060577 0.00000000 224.14676232
-2.38740004 0.59060577 0.00000000 231.94534584
-2.44031774 0.53768808 0.00000000 760.31130835
-2.44031774 0.64352347 0.00000000 719.80761187
-1.33902835 1.35334636 0.00000000 -580.14101088
-1.23319294 1.35334636 0.00000000 -606.45578904
-1.28611065 1.30042866 0.00000000 522.01681008
-1.28611065 1.40626406 0.00000000 490.45334328
45 0.0 # 0 nm, 1st Exc. (r2v6r2v6) 45
1.74098131 -1.46780024 0.00000000 1.0 # electr. mom.
1.31876649 -1.08237463 0.00000000 -3637.42173509
0.00000000 -0.70208822 0.00000000 1463.58358291
2.11091906 0.04406596 0.00000000 827.34939854
1.32524096 1.15754064 0.00000000 -2731.65780005
-0.04178899 0.70146698 0.00000000 740.99122666
-1.16182154 -1.48163476 0.00000000 -301.58102534
-2.37713566 -0.81881509 0.00000000 -1111.45560634
-2.44031774 0.59060577 0.00000000 -1930.01932901
-1.28611065 1.35334636 0.00000000 171.55611859
1.26584879 -1.08237463 0.00000000 297.61113893
1.37168419 -1.08237463 0.00000000 344.11727213
1.31876649 -1.13529233 0.00000000 1409.80008139
1.31876649 -1.02945693 0.00000000 1602.16176106
-0.05291770 -0.70208822 0.00000000 -157.40184773
0.05291770 -0.70208822 0.00000000 -233.04201086
0.00000000 -0.75500592 0.00000000 -450.63016843
0.00000000 -0.64917052 0.00000000 -632.53874990
2.05800137 0.04406596 0.00000000 -229.52602070
2.16383676 0.04406596 0.00000000 -32.12191310
2.11091906 -0.00885174 0.00000000 -291.64397170
2.11091906 0.09698366 0.00000000 -296.91813470
1.27232327 1.15754064 0.00000000 174.91054651
1.37815866 1.15754064 0.00000000 222.30167765
1.32524096 1.10462294 0.00000000 1270.48523702
1.32524096 1.21045834 0.00000000 1079.24918803
-0.09470669 0.70146698 0.00000000 -1250.82118382
0.01112871 0.70146698 0.00000000 -1293.61212259
-0.04178899 0.64854929 0.00000000 926.89080523
-0.04178899 0.75438469 0.00000000 881.53434384
-1.21473924 -1.48163476 0.00000000 -198.39718790
-1.10890384 -1.48163476 0.00000000 -188.34576802
-1.16182154 -1.53455246 0.00000000 358.63747334
-1.16182154 -1.42871706 0.00000000 322.44546130
-2.43005336 -0.81881509 0.00000000 424.97900290
-2.32421796 -0.81881509 0.00000000 483.69524501
-2.37713566 -0.87173279 0.00000000 139.56833405
-2.37713566 -0.76589740 0.00000000 63.18229838
-2.49323545 0.59060577 0.00000000 224.14676232
-2.38740004 0.59060577 0.00000000 231.94534584
-2.44031774 0.53768808 0.00000000 760.31130835
-2.44031774 0.64352347 0.00000000 719.80761187
-1.33902835 1.35334636 0.00000000 -580.14101088
-1.23319294 1.35334636 0.00000000 -606.45578904
-1.28611065 1.30042866 0.00000000 522.01681008
-1.28611065 1.40626406 0.00000000 490.45334328
45 0.0 # 0 nm, 1st Exc. (r2v7r2v7) 45

1.74098131	-1.46780024	0.00000000	1.0 # electr. mom.
1.31876649	-1.08237463	0.00000000	-3637.42173509
0.00000000	-0.70208822	0.00000000	1463.58358291
2.11091906	0.04406596	0.00000000	827.34939854
1.32524096	1.15754064	0.00000000	-2731.65780005
-0.04178899	0.70146698	0.00000000	740.99122666
-1.16182154	-1.48163476	0.00000000	-301.58102534
-2.37713566	-0.81881509	0.00000000	-1111.45560634
-2.44031774	0.59060577	0.00000000	-1930.01932901
-1.28611065	1.35334636	0.00000000	171.55611859
1.26584879	-1.08237463	0.00000000	297.61113893
1.37168419	-1.08237463	0.00000000	344.11727213
1.31876649	-1.13529233	0.00000000	1409.80008139
1.31876649	-1.02945693	0.00000000	1602.16176106
-0.05291770	-0.70208822	0.00000000	-157.40184773
0.05291770	-0.70208822	0.00000000	-233.04201086
0.00000000	-0.75500592	0.00000000	-450.63016843
0.00000000	-0.64917052	0.00000000	-632.53874990
2.05800137	0.04406596	0.00000000	-229.52602070
2.16383676	0.04406596	0.00000000	-32.12191310
2.11091906	-0.00885174	0.00000000	-291.64397170
2.11091906	0.09698366	0.00000000	-296.91813470
1.27232327	1.15754064	0.00000000	174.91054651
1.37815866	1.15754064	0.00000000	222.30167765
1.32524096	1.10462294	0.00000000	1270.48523702
1.32524096	1.21045834	0.00000000	1079.24918803
-0.09470669	0.70146698	0.00000000	-1250.82118382
0.01112871	0.70146698	0.00000000	-1293.61212259
-0.04178899	0.64854929	0.00000000	926.89080523
-0.04178899	0.75438469	0.00000000	881.53434384
-1.21473924	-1.48163476	0.00000000	-198.39718790
-1.10890384	-1.48163476	0.00000000	-188.34576802
-1.16182154	-1.53455246	0.00000000	358.63747334
-1.16182154	-1.42871706	0.00000000	322.44546130
-2.43005336	-0.81881509	0.00000000	424.97900290
-2.32421796	-0.81881509	0.00000000	483.69524501
-2.37713566	-0.87173279	0.00000000	139.56833405
-2.37713566	-0.76589740	0.00000000	63.18229838
-2.49323545	0.59060577	0.00000000	224.14676232
-2.38740004	0.59060577	0.00000000	231.94534584
-2.44031774	0.53768808	0.00000000	760.31130835
-2.44031774	0.64352347	0.00000000	719.80761187
-1.33902835	1.35334636	0.00000000	-580.14101088
-1.23319294	1.35334636	0.00000000	-606.45578904
-1.28611065	1.30042866	0.00000000	522.01681008
-1.28611065	1.40626406	0.00000000	490.45334328
45 0.0 # 0 nm, 2nd Exc. (r3v1r3v1) 45			
8.02920110	-2.39813299	0.00000000	1.0 # electr. mom.
1.31876649	-1.08237463	0.00000000	-3111.09173827
0.00000000	-0.70208822	0.00000000	479.26392014
2.11091906	0.04406596	0.00000000	877.98729350
1.32524096	1.15754064	0.00000000	-2295.52199851
-0.04178899	0.70146698	0.00000000	1036.04327866
-1.16182154	-1.48163476	0.00000000	-465.13678925
-2.37713566	-0.81881509	0.00000000	-1363.24957435
-2.44031774	0.59060577	0.00000000	-2009.00655979
-1.28611065	1.35334636	0.00000000	-544.75823597
1.26584879	-1.08237463	0.00000000	233.39872829
1.37168419	-1.08237463	0.00000000	295.86154570
1.31876649	-1.13529233	0.00000000	1218.70527062
1.31876649	-1.02945693	0.00000000	1377.45140990
-0.05291770	-0.70208822	0.00000000	550.90011254
0.05291770	-0.70208822	0.00000000	473.15773978
0.00000000	-0.75500592	0.00000000	-648.35457547
0.00000000	-0.64917052	0.00000000	-861.59159222
2.05800137	0.04406596	0.00000000	-190.40195750
2.16383676	0.04406596	0.00000000	-0.67199246
2.11091906	-0.00885174	0.00000000	-359.03366035
2.11091906	0.09698366	0.00000000	-349.48574362
1.27232327	1.15754064	0.00000000	-24.63705101
1.37815866	1.15754064	0.00000000	27.97454678
1.32524096	1.10462294	0.00000000	1250.73639888
1.32524096	1.21045834	0.00000000	1057.35734616
-0.09470669	0.70146698	0.00000000	-1494.89930760
0.01112871	0.70146698	0.00000000	-1533.08509838
-0.04178899	0.64854929	0.00000000	1026.80544518
-0.04178899	0.75438469	0.00000000	969.79281115
-1.21473924	-1.48163476	0.00000000	-30.60842189
-1.10890384	-1.48163476	0.00000000	-22.63615646
-1.16182154	-1.53455246	0.00000000	289.05083304
-1.16182154	-1.42871706	0.00000000	218.34096355
-2.43005336	-0.81881509	0.00000000	555.91725211
-2.32421796	-0.81881509	0.00000000	639.48838378
-2.37713566	-0.87173279	0.00000000	125.63083224
-2.37713566	-0.76589740	0.00000000	43.02867206
-2.49323545	0.59060577	0.00000000	243.28207517
-2.38740004	0.59060577	0.00000000	240.17051131

-2.44031774	0.53768808	0.00000000	789.74795342
-2.44031774	0.64352347	0.00000000	740.60692531
-1.33902835	1.35334636	0.00000000	-307.75011542
-1.23319294	1.35334636	0.00000000	-337.84948114
-1.28611065	1.30042866	0.00000000	619.45769813
-1.28611065	1.40626406	0.00000000	569.61210230
45 0.0 # 0 nm, 2nd Exc. (r3v2r3v2) 45			
8.02920110	-2.39813299	0.00000000 1.0 # electr. mom.	
1.31876649	-1.08237463	0.00000000	-3111.09173827
0.00000000	-0.70208822	0.00000000	479.26392014
2.11091906	0.04406596	0.00000000	877.98729350
1.32524096	1.15754064	0.00000000	-2295.52199851
-0.04178899	0.70146698	0.00000000	1036.04327866
-1.16182154	-1.48163476	0.00000000	-465.13678925
-2.37713566	-0.81881509	0.00000000	-1363.24957435
-2.44031774	0.59060577	0.00000000	-2009.00655979
-1.28611065	1.35334636	0.00000000	-544.75823597
1.26584879	-1.08237463	0.00000000	233.39872829
1.37168419	-1.08237463	0.00000000	295.86154570
1.31876649	-1.13529233	0.00000000	1218.70527062
1.31876649	-1.02945693	0.00000000	1377.45140990
-0.05291770	-0.70208822	0.00000000	550.90011254
0.05291770	-0.70208822	0.00000000	473.15773978
0.00000000	-0.75500592	0.00000000	-648.35457547
0.00000000	-0.64917052	0.00000000	-861.59159222
2.05800137	0.04406596	0.00000000	-190.40195750
2.16383676	0.04406596	0.00000000	-0.67199246
2.11091906	-0.00885174	0.00000000	-359.03366035
2.11091906	0.09698366	0.00000000	-349.48574362
1.27232327	1.15754064	0.00000000	-24.63705101
1.37815866	1.15754064	0.00000000	27.97454678
1.32524096	1.10462294	0.00000000	1250.73639888
1.32524096	1.21045834	0.00000000	1057.35734616
-0.09470669	0.70146698	0.00000000	-1494.89930760
0.01112871	0.70146698	0.00000000	-1533.08509838
-0.04178899	0.64854929	0.00000000	1026.80544518
-0.04178899	0.75438469	0.00000000	969.79281115
-1.21473924	-1.48163476	0.00000000	-30.60842189
-1.10890384	-1.48163476	0.00000000	-22.63615646
-1.16182154	-1.53455246	0.00000000	289.05083304
-1.16182154	-1.42871706	0.00000000	218.34096355
-2.43005336	-0.81881509	0.00000000	555.91725211
-2.32421796	-0.81881509	0.00000000	639.48838378
-2.37713566	-0.87173279	0.00000000	125.63083224
-2.37713566	-0.76589740	0.00000000	43.02867206
-2.49323545	0.59060577	0.00000000	243.28207517
-2.38740004	0.59060577	0.00000000	240.17051131
-2.44031774	0.53768808	0.00000000	789.74795342
-2.44031774	0.64352347	0.00000000	740.60692531
-1.33902835	1.35334636	0.00000000	-307.75011542
-1.23319294	1.35334636	0.00000000	-337.84948114
-1.28611065	1.30042866	0.00000000	619.45769813
-1.28611065	1.40626406	0.00000000	569.61210230
45 0.0 # 0 nm, 2nd Exc. (r3v3r3v3) 45			
8.02920110	-2.39813299	0.00000000 1.0 # electr. mom.	
1.31876649	-1.08237463	0.00000000	-3111.09173827
0.00000000	-0.70208822	0.00000000	479.26392014
2.11091906	0.04406596	0.00000000	877.98729350
1.32524096	1.15754064	0.00000000	-2295.52199851
-0.04178899	0.70146698	0.00000000	1036.04327866
-1.16182154	-1.48163476	0.00000000	-465.13678925
-2.37713566	-0.81881509	0.00000000	-1363.24957435
-2.44031774	0.59060577	0.00000000	-2009.00655979
-1.28611065	1.35334636	0.00000000	-544.75823597
1.26584879	-1.08237463	0.00000000	233.39872829
1.37168419	-1.08237463	0.00000000	295.86154570
1.31876649	-1.13529233	0.00000000	1218.70527062
1.31876649	-1.02945693	0.00000000	1377.45140990
-0.05291770	-0.70208822	0.00000000	550.90011254
0.05291770	-0.70208822	0.00000000	473.15773978
0.00000000	-0.75500592	0.00000000	-648.35457547
0.00000000	-0.64917052	0.00000000	-861.59159222
2.05800137	0.04406596	0.00000000	-190.40195750
2.16383676	0.04406596	0.00000000	-0.67199246
2.11091906	-0.00885174	0.00000000	-359.03366035
2.11091906	0.09698366	0.00000000	-349.48574362
1.27232327	1.15754064	0.00000000	-24.63705101
1.37815866	1.15754064	0.00000000	27.97454678
1.32524096	1.10462294	0.00000000	1250.73639888
1.32524096	1.21045834	0.00000000	1057.35734616
-0.09470669	0.70146698	0.00000000	-1494.89930760
0.01112871	0.70146698	0.00000000	-1533.08509838
-0.04178899	0.64854929	0.00000000	1026.80544518
-0.04178899	0.75438469	0.00000000	969.79281115
-1.21473924	-1.48163476	0.00000000	-30.60842189
-1.10890384	-1.48163476	0.00000000	-22.63615646
-1.16182154	-1.53455246	0.00000000	289.05083304

-1.16182154	-1.42871706	0.00000000	218.34096355
-2.43005336	-0.81881509	0.00000000	555.91725211
-2.32421796	-0.81881509	0.00000000	639.48838378
-2.37713566	-0.87173279	0.00000000	125.63083224
-2.37713566	-0.76589740	0.00000000	43.02867206
-2.49323545	0.59060577	0.00000000	243.28207517
-2.38740004	0.59060577	0.00000000	240.17051131
-2.44031774	0.53768808	0.00000000	789.74795342
-2.44031774	0.64352347	0.00000000	740.60692531
-1.33902835	1.35334636	0.00000000	-307.75011542
-1.23319294	1.35334636	0.00000000	-337.84948114
-1.28611065	1.30042866	0.00000000	619.45769813
-1.28611065	1.40626406	0.00000000	569.61210230
45 0.0 # 0 nm, 2nd Exc. (r3v4r3v4) 45			
8.02920110	-2.39813299	0.00000000	1.0 # electr. mom.
1.31876649	-1.08237463	0.00000000	-3111.09173827
0.00000000	-0.70208822	0.00000000	479.26392014
2.11091906	0.04406596	0.00000000	877.98729350
1.32524096	1.15754064	0.00000000	-2295.52199851
-0.04178899	0.70146698	0.00000000	1036.04327866
-1.16182154	-1.48163476	0.00000000	-465.13678925
-2.37713566	-0.81881509	0.00000000	-1363.24957435
-2.44031774	0.59060577	0.00000000	-2009.00655979
-1.28611065	1.35334636	0.00000000	-544.75823597
1.26584879	-1.08237463	0.00000000	233.39872829
1.37168419	-1.08237463	0.00000000	295.86154570
1.31876649	-1.13529233	0.00000000	1218.70527062
1.31876649	-1.02945693	0.00000000	1377.45140990
-0.05291770	-0.70208822	0.00000000	550.90011254
0.05291770	-0.70208822	0.00000000	473.15773978
0.00000000	-0.75500592	0.00000000	-648.35457547
0.00000000	-0.64917052	0.00000000	-861.59159222
2.05800137	0.04406596	0.00000000	-190.40195750
2.16383676	0.04406596	0.00000000	-0.67199246
2.11091906	-0.00885174	0.00000000	-359.03366035
2.11091906	0.09698366	0.00000000	-349.48574362
1.27232327	1.15754064	0.00000000	-24.63705101
1.37815866	1.15754064	0.00000000	27.97454678
1.32524096	1.10462294	0.00000000	1250.73639888
1.32524096	1.21045834	0.00000000	1057.35734616
-0.09470669	0.70146698	0.00000000	-1494.89930760
0.01112871	0.70146698	0.00000000	-1533.08509838
-0.04178899	0.64854929	0.00000000	1026.80544518
-0.04178899	0.75438469	0.00000000	969.79281115
-1.21473924	-1.48163476	0.00000000	-30.60842189
-1.10890384	-1.48163476	0.00000000	-22.63615646
-1.16182154	-1.53455246	0.00000000	289.05083304
-1.16182154	-1.42871706	0.00000000	218.34096355
-2.43005336	-0.81881509	0.00000000	555.91725211
-2.32421796	-0.81881509	0.00000000	639.48838378
-2.37713566	-0.87173279	0.00000000	125.63083224
-2.37713566	-0.76589740	0.00000000	43.02867206
-2.49323545	0.59060577	0.00000000	243.28207517
-2.38740004	0.59060577	0.00000000	240.17051131
-2.44031774	0.53768808	0.00000000	789.74795342
-2.44031774	0.64352347	0.00000000	740.60692531
-1.33902835	1.35334636	0.00000000	-307.75011542
-1.23319294	1.35334636	0.00000000	-337.84948114
-1.28611065	1.30042866	0.00000000	619.45769813
-1.28611065	1.40626406	0.00000000	569.61210230
45 0.0 # 0 nm, 2nd Exc. (r3v5r3v5) 45			
8.02920110	-2.39813299	0.00000000	1.0 # electr. mom.
1.31876649	-1.08237463	0.00000000	-3111.09173827
0.00000000	-0.70208822	0.00000000	479.26392014
2.11091906	0.04406596	0.00000000	877.98729350
1.32524096	1.15754064	0.00000000	-2295.52199851
-0.04178899	0.70146698	0.00000000	1036.04327866
-1.16182154	-1.48163476	0.00000000	-465.13678925
-2.37713566	-0.81881509	0.00000000	-1363.24957435
-2.44031774	0.59060577	0.00000000	-2009.00655979
-1.28611065	1.35334636	0.00000000	-544.75823597
1.26584879	-1.08237463	0.00000000	233.39872829
1.37168419	-1.08237463	0.00000000	295.86154570
1.31876649	-1.13529233	0.00000000	1218.70527062
1.31876649	-1.02945693	0.00000000	1377.45140990
-0.05291770	-0.70208822	0.00000000	550.90011254
0.05291770	-0.70208822	0.00000000	473.15773978
0.00000000	-0.75500592	0.00000000	-648.35457547
0.00000000	-0.64917052	0.00000000	-861.59159222
2.05800137	0.04406596	0.00000000	-190.40195750
2.16383676	0.04406596	0.00000000	-0.67199246
2.11091906	-0.00885174	0.00000000	-359.03366035
2.11091906	0.09698366	0.00000000	-349.48574362
1.27232327	1.15754064	0.00000000	-24.63705101
1.37815866	1.15754064	0.00000000	27.97454678
1.32524096	1.10462294	0.00000000	1250.73639888
1.32524096	1.21045834	0.00000000	1057.35734616

-0.09470669	0.70146698	0.00000000	-1494.89930760
0.01112871	0.70146698	0.00000000	-1533.08509838
-0.04178899	0.64854929	0.00000000	1026.80544518
-0.04178899	0.75438469	0.00000000	969.79281115
-1.21473924	-1.48163476	0.00000000	-30.60842189
-1.10890384	-1.48163476	0.00000000	-22.63615646
-1.16182154	-1.53455246	0.00000000	289.05083304
-1.16182154	-1.42871706	0.00000000	218.34096355
-2.43005336	-0.81881509	0.00000000	555.91725211
-2.32421796	-0.81881509	0.00000000	639.48838378
-2.37713566	-0.87173279	0.00000000	125.63083224
-2.37713566	-0.76589740	0.00000000	43.02867206
-2.49323545	0.59060577	0.00000000	243.28207517
-2.38740004	0.59060577	0.00000000	240.17051131
-2.44031774	0.53768808	0.00000000	789.74795342
-2.44031774	0.64352347	0.00000000	740.60692531
-1.33902835	1.35334636	0.00000000	-307.75011542
-1.23319294	1.35334636	0.00000000	-337.84948114
-1.28611065	1.30042866	0.00000000	619.45769813
-1.28611065	1.40626406	0.00000000	569.61210230
45 0.0 # 0 nm, 2nd Exc. (r3v6r3v6) 45			
8.02920110	-2.39813299	0.00000000 1.0 # electr. mom.	
1.31876649	-1.08237463	0.00000000	-3111.09173827
0.00000000	-0.70208822	0.00000000	479.26392014
2.11091906	0.04406596	0.00000000	877.98729350
1.32524096	1.15754064	0.00000000	-2295.52199851
-0.04178899	0.70146698	0.00000000	1036.04327866
-1.16182154	-1.48163476	0.00000000	-465.13678925
-2.37713566	-0.81881509	0.00000000	-1363.24957435
-2.44031774	0.59060577	0.00000000	-2009.00655979
-1.28611065	1.35334636	0.00000000	-544.75823597
1.26584879	-1.08237463	0.00000000	233.39872829
1.37168419	-1.08237463	0.00000000	295.86154570
1.31876649	-1.13529233	0.00000000	1218.70527062
1.31876649	-1.02945693	0.00000000	1377.45140990
-0.05291770	-0.70208822	0.00000000	550.90011254
0.05291770	-0.70208822	0.00000000	473.15773978
0.00000000	-0.75500592	0.00000000	-648.35457547
0.00000000	-0.64917052	0.00000000	-861.59159222
2.05800137	0.04406596	0.00000000	-190.40195750
2.16383676	0.04406596	0.00000000	-0.67199246
2.11091906	-0.00885174	0.00000000	-359.03366035
2.11091906	0.09698366	0.00000000	-349.48574362
1.27232327	1.15754064	0.00000000	-24.63705101
1.37815866	1.15754064	0.00000000	27.97454678
1.32524096	1.10462294	0.00000000	1250.73639888
1.32524096	1.21045834	0.00000000	1057.35734616
-0.09470669	0.70146698	0.00000000	-1494.89930760
0.01112871	0.70146698	0.00000000	-1533.08509838
-0.04178899	0.64854929	0.00000000	1026.80544518
-0.04178899	0.75438469	0.00000000	969.79281115
-1.21473924	-1.48163476	0.00000000	-30.60842189
-1.10890384	-1.48163476	0.00000000	-22.63615646
-1.16182154	-1.53455246	0.00000000	289.05083304
-1.16182154	-1.42871706	0.00000000	218.34096355
-2.43005336	-0.81881509	0.00000000	555.91725211
-2.32421796	-0.81881509	0.00000000	639.48838378
-2.37713566	-0.87173279	0.00000000	125.63083224
-2.37713566	-0.76589740	0.00000000	43.02867206
-2.49323545	0.59060577	0.00000000	243.28207517
-2.38740004	0.59060577	0.00000000	240.17051131
-2.44031774	0.53768808	0.00000000	789.74795342
-2.44031774	0.64352347	0.00000000	740.60692531
-1.33902835	1.35334636	0.00000000	-307.75011542
-1.23319294	1.35334636	0.00000000	-337.84948114
-1.28611065	1.30042866	0.00000000	619.45769813
-1.28611065	1.40626406	0.00000000	569.61210230
45 0.0 # 0 nm, 2nd Exc. (r3v7r3v7) 45			
8.02920110	-2.39813299	0.00000000 1.0 # electr. mom.	
1.31876649	-1.08237463	0.00000000	-3111.09173827
0.00000000	-0.70208822	0.00000000	479.26392014
2.11091906	0.04406596	0.00000000	877.98729350
1.32524096	1.15754064	0.00000000	-2295.52199851
-0.04178899	0.70146698	0.00000000	1036.04327866
-1.16182154	-1.48163476	0.00000000	-465.13678925
-2.37713566	-0.81881509	0.00000000	-1363.24957435
-2.44031774	0.59060577	0.00000000	-2009.00655979
-1.28611065	1.35334636	0.00000000	-544.75823597
1.26584879	-1.08237463	0.00000000	233.39872829
1.37168419	-1.08237463	0.00000000	295.86154570
1.31876649	-1.13529233	0.00000000	1218.70527062
1.31876649	-1.02945693	0.00000000	1377.45140990
-0.05291770	-0.70208822	0.00000000	550.90011254
0.05291770	-0.70208822	0.00000000	473.15773978
0.00000000	-0.75500592	0.00000000	-648.35457547
0.00000000	-0.64917052	0.00000000	-861.59159222
2.05800137	0.04406596	0.00000000	-190.40195750

2.16383676	0.04406596	0.00000000	-0.67199246
2.11091906	-0.00885174	0.00000000	-359.03366035
2.11091906	0.09698366	0.00000000	-349.48574362
1.27232327	1.15754064	0.00000000	-24.63705101
1.37815866	1.15754064	0.00000000	27.97454678
1.32524096	1.10462294	0.00000000	1250.73639888
1.32524096	1.21045834	0.00000000	1057.35734616
-0.09470669	0.70146698	0.00000000	-1494.89930760
0.01112871	0.70146698	0.00000000	-1533.08509838
-0.04178899	0.64854929	0.00000000	1026.80544518
-0.04178899	0.75438469	0.00000000	969.79281115
-1.21473924	-1.48163476	0.00000000	-30.60842189
-1.10890384	-1.48163476	0.00000000	-22.63615646
-1.16182154	-1.53455246	0.00000000	289.05083304
-1.16182154	-1.42871706	0.00000000	218.34096355
-2.43005336	-0.81881509	0.00000000	555.91725211
-2.32421796	-0.81881509	0.00000000	639.48838378
-2.37713566	-0.87173279	0.00000000	125.63083224
-2.37713566	-0.76589740	0.00000000	43.02867206
-2.49323545	0.59060577	0.00000000	243.28207517
-2.38740004	0.59060577	0.00000000	240.17051131
-2.44031774	0.53768808	0.00000000	789.74795342
-2.44031774	0.64352347	0.00000000	740.60692531
-1.33902835	1.35334636	0.00000000	-307.75011542
-1.23319294	1.35334636	0.00000000	-337.84948114
-1.28611065	1.30042866	0.00000000	619.45769813
-1.28611065	1.40626406	0.00000000	569.61210230

45 0.0 # 0 nm, 2nd Exc. (r3v8r3v8) 45

8.02920110	-2.39813299	0.00000000	1.0 # electr. mom.
1.31876649	-1.08237463	0.00000000	-3111.09173827
0.00000000	-0.70208822	0.00000000	479.26392014
2.11091906	0.04406596	0.00000000	877.98729350
1.32524096	1.15754064	0.00000000	-2295.52199851
-0.04178899	0.70146698	0.00000000	1036.04327866
-1.16182154	-1.48163476	0.00000000	-465.13678925
-2.37713566	-0.81881509	0.00000000	-1363.24957435
-2.44031774	0.59060577	0.00000000	-2009.00655979
-1.28611065	1.35334636	0.00000000	-544.75823597
1.26584879	-1.08237463	0.00000000	233.39872829
1.37168419	-1.08237463	0.00000000	295.86154570
1.31876649	-1.13529233	0.00000000	1218.70527062
1.31876649	-1.02945693	0.00000000	1377.45140990
-0.05291770	-0.70208822	0.00000000	550.90011254
0.05291770	-0.70208822	0.00000000	473.15773978
0.00000000	-0.75500592	0.00000000	-648.35457547
0.00000000	-0.64917052	0.00000000	-861.59159222
2.05800137	0.04406596	0.00000000	-190.40195750
2.16383676	0.04406596	0.00000000	-0.67199246
2.11091906	-0.00885174	0.00000000	-359.03366035
2.11091906	0.09698366	0.00000000	-349.48574362
1.27232327	1.15754064	0.00000000	-24.63705101
1.37815866	1.15754064	0.00000000	27.97454678
1.32524096	1.10462294	0.00000000	1250.73639888
1.32524096	1.21045834	0.00000000	1057.35734616
-0.09470669	0.70146698	0.00000000	-1494.89930760
0.01112871	0.70146698	0.00000000	-1533.08509838
-0.04178899	0.64854929	0.00000000	1026.80544518
-0.04178899	0.75438469	0.00000000	969.79281115
-1.21473924	-1.48163476	0.00000000	-30.60842189
-1.10890384	-1.48163476	0.00000000	-22.63615646
-1.16182154	-1.53455246	0.00000000	289.05083304
-1.16182154	-1.42871706	0.00000000	218.34096355
-2.43005336	-0.81881509	0.00000000	555.91725211
-2.32421796	-0.81881509	0.00000000	639.48838378
-2.37713566	-0.87173279	0.00000000	125.63083224
-2.37713566	-0.76589740	0.00000000	43.02867206
-2.49323545	0.59060577	0.00000000	243.28207517
-2.38740004	0.59060577	0.00000000	240.17051131
-2.44031774	0.53768808	0.00000000	789.74795342
-2.44031774	0.64352347	0.00000000	740.60692531
-1.33902835	1.35334636	0.00000000	-307.75011542
-1.23319294	1.35334636	0.00000000	-337.84948114
-1.28611065	1.30042866	0.00000000	619.45769813
-1.28611065	1.40626406	0.00000000	569.61210230

45 0.0 # 0 nm, 2nd Exc. (r3v9r3v9) 45

8.02920110	-2.39813299	0.00000000	1.0 # electr. mom.
1.31876649	-1.08237463	0.00000000	-3111.09173827
0.00000000	-0.70208822	0.00000000	479.26392014
2.11091906	0.04406596	0.00000000	877.98729350
1.32524096	1.15754064	0.00000000	-2295.52199851
-0.04178899	0.70146698	0.00000000	1036.04327866
-1.16182154	-1.48163476	0.00000000	-465.13678925
-2.37713566	-0.81881509	0.00000000	-1363.24957435
-2.44031774	0.59060577	0.00000000	-2009.00655979
-1.28611065	1.35334636	0.00000000	-544.75823597
1.26584879	-1.08237463	0.00000000	233.39872829
1.37168419	-1.08237463	0.00000000	295.86154570

1.31876649	-1.13529233	0.00000000	1218.70527062
1.31876649	-1.02945693	0.00000000	1377.45140990
-0.05291770	-0.70208822	0.00000000	550.90011254
0.05291770	-0.70208822	0.00000000	473.15773978
0.00000000	-0.75500592	0.00000000	-648.35457547
0.00000000	-0.64917052	0.00000000	-861.59159222
2.05800137	0.04406596	0.00000000	-190.40195750
2.16383676	0.04406596	0.00000000	-0.67199246
2.11091906	-0.00885174	0.00000000	-359.03366035
2.11091906	0.09698366	0.00000000	-349.48574362
1.27232327	1.15754064	0.00000000	-24.63705101
1.37815866	1.15754064	0.00000000	27.97454678
1.32524096	1.10462294	0.00000000	1250.73639888
1.32524096	1.21045834	0.00000000	1057.35734616
-0.09470669	0.70146698	0.00000000	-1494.89930760
0.01112871	0.70146698	0.00000000	-1533.08509838
-0.04178899	0.64854929	0.00000000	1026.80544518
-0.04178899	0.75438469	0.00000000	969.79281115
-1.21473924	-1.48163476	0.00000000	-30.60842189
-1.10890384	-1.48163476	0.00000000	-22.63615646
-1.16182154	-1.53455246	0.00000000	289.05083304
-1.16182154	-1.42871706	0.00000000	218.34096355
-2.43005336	-0.81881509	0.00000000	555.91725211
-2.32421796	-0.81881509	0.00000000	639.48838378
-2.37713566	-0.87173279	0.00000000	125.63083224
-2.37713566	-0.76589740	0.00000000	43.02867206
-2.49323545	0.59060577	0.00000000	243.28207517
-2.38740004	0.59060577	0.00000000	240.17051131
-2.44031774	0.53768808	0.00000000	789.74795342
-2.44031774	0.64352347	0.00000000	740.60692531
-1.33902835	1.35334636	0.00000000	-307.75011542
-1.23319294	1.35334636	0.00000000	-337.84948114
-1.28611065	1.30042866	0.00000000	619.45769813
-1.28611065	1.40626406	0.00000000	569.61210230
45 0.0 # 0 nm, 3rd Exc. (r7r7) 45			
4.06009902	-2.86027426	0.00000000	1.0 # electr. mom.
1.31876649	-1.08237463	0.00000000	-3735.26980776
0.00000000	-0.70208822	0.00000000	1000.21899619
2.11091906	0.04406596	0.00000000	824.03983440
1.32524096	1.15754064	0.00000000	-2885.77104710
-0.04178899	0.70146698	0.00000000	1820.91866554
-1.16182154	-1.48163476	0.00000000	612.51893496
-2.37713566	-0.81881509	0.00000000	-1372.98419107
-2.44031774	0.59060577	0.00000000	-2286.38162059
-1.28611065	1.35334636	0.00000000	-555.53556403
1.26584879	-1.08237463	0.00000000	235.86530256
1.37168419	-1.08237463	0.00000000	270.61231200
1.31876649	-1.13529233	0.00000000	1519.72118429
1.31876649	-1.02945693	0.00000000	1726.99447190
-0.05291770	-0.70208822	0.00000000	-91.96350739
0.05291770	-0.70208822	0.00000000	-248.87764190
0.00000000	-0.75500592	0.00000000	-246.97495622
0.00000000	-0.64917052	0.00000000	-415.62653885
2.05800137	0.04406596	0.00000000	-295.92775618
2.16383676	0.04406596	0.00000000	-84.65721523
2.11091906	-0.00885174	0.00000000	-225.16961506
2.11091906	0.09698366	0.00000000	-242.35538971
1.27232327	1.15754064	0.00000000	164.72294237
1.37815866	1.15754064	0.00000000	217.12547059
1.32524096	1.10462294	0.00000000	1368.29667826
1.32524096	1.21045834	0.00000000	1152.66508738
-0.09470669	0.70146698	0.00000000	-1708.54684272
0.01112871	0.70146698	0.00000000	-1699.99710446
-0.04178899	0.64854929	0.00000000	796.96449250
-0.04178899	0.75438469	0.00000000	788.06617502
-1.21473924	-1.48163476	0.00000000	-472.95457982
-1.10890384	-1.48163476	0.00000000	-479.89029490
-1.16182154	-1.53455246	0.00000000	214.61770075
-1.16182154	-1.42871706	0.00000000	110.72380555
-2.43005336	-0.81881509	0.00000000	640.52974651
-2.32421796	-0.81881509	0.00000000	753.91074782
-2.37713566	-0.87173279	0.00000000	42.49710883
-2.37713566	-0.76589740	0.00000000	-62.30006362
-2.49323545	0.59060577	0.00000000	104.16983366
-2.38740004	0.59060577	0.00000000	60.14338723
-2.44031774	0.53768808	0.00000000	1103.08729459
-2.44031774	0.64352347	0.00000000	1022.80817126
-1.33902835	1.35334636	0.00000000	-514.21888330
-1.23319294	1.35334636	0.00000000	-535.85580672
-1.28611065	1.30042866	0.00000000	858.20278666
-1.28611065	1.40626406	0.00000000	751.83729566
45 0.0 # 0 nm, 4th Exc. (r8r8) 45			
1.23654057	-2.24688502	0.00000000	1.0 # electr. mom.
1.31876649	-1.08237463	0.00000000	-3550.64407416
0.00000000	-0.70208822	0.00000000	1234.44807946
2.11091906	0.04406596	0.00000000	717.53197382
1.32524096	1.15754064	0.00000000	-3067.91716526

-0.04178899	0.70146698	0.00000000	1162.94488867
-1.16182154	-1.48163476	0.00000000	-34.40206075
-2.37713566	-0.81881509	0.00000000	-1345.43180938
-2.44031774	0.59060577	0.00000000	-2142.44037595
-1.28611065	1.35334636	0.00000000	210.36958426
1.26584879	-1.08237463	0.00000000	242.17297877
1.37168419	-1.08237463	0.00000000	296.95675973
1.31876649	-1.13529233	0.00000000	1417.38380640
1.31876649	-1.02945693	0.00000000	1609.56253411
-0.05291770	-0.70208822	0.00000000	111.90882499
0.05291770	-0.70208822	0.00000000	37.12837075
0.00000000	-0.75500592	0.00000000	-582.29201381
0.00000000	-0.64917052	0.00000000	-811.63249661
2.05800137	0.04406596	0.00000000	-246.28606858
2.16383676	0.04406596	0.00000000	-40.95144758
2.11091906	-0.00885174	0.00000000	-224.17681834
2.11091906	0.09698366	0.00000000	-230.18326795
1.27232327	1.15754064	0.00000000	160.42357920
1.37815866	1.15754064	0.00000000	211.92395875
1.32524096	1.10462294	0.00000000	1470.66927360
1.32524096	1.21045834	0.00000000	1242.41696155
-0.09470669	0.70146698	0.00000000	-1757.10079882
0.01112871	0.70146698	0.00000000	-1806.49369709
-0.04178899	0.64854929	0.00000000	1241.90016058
-0.04178899	0.75438469	0.00000000	1163.89848806
-1.21473924	-1.48163476	0.00000000	-272.36063506
-1.10890384	-1.48163476	0.00000000	-260.48221810
-1.16182154	-1.53455246	0.00000000	306.04728485
-1.16182154	-1.42871706	0.00000000	252.34295702
-2.43005336	-0.81881509	0.00000000	472.98802286
-2.32421796	-0.81881509	0.00000000	538.65442522
-2.37713566	-0.87173279	0.00000000	205.10762606
-2.37713566	-0.76589740	0.00000000	128.95936008
-2.49323545	0.59060577	0.00000000	193.34497531
-2.38740004	0.59060577	0.00000000	193.25080762
-2.44031774	0.53768808	0.00000000	904.14281366
-2.44031774	0.64352347	0.00000000	857.83232472
-1.33902835	1.35334636	0.00000000	-687.05049379
-1.23319294	1.35334636	0.00000000	-714.87745920
-1.28611065	1.30042866	0.00000000	622.11296040
-1.28611065	1.40626406	0.00000000	568.29911981
